# Supplementary material for: Global prevalence and trends in hypertension and type 2 diabetes mellitus among slum residents: a systematic review and meta-analysis
Source: BMJ Open. 2022 Feb 24;12(2):e052393. doi: 10.1136/bmjopen-2021-052393 (PMC8883228; doi:10.1136/bmjopen-2021-052393)
Supplement: Supplementary data [file bmjopen-2021-052393supp001.pdf]

Supplementary Digital Content

Table of Contents

eFigure 1: Study selection and inclusion flow chart 1

Box 1: Study selection and inclusion flow chart 2

eTable 1: List of Excluded Studies 3

eTable 2: Characteristics of included studies 11

eTable 3: Risk of bias of included studies 13

Annex 1: MEDLINE Search Strategy 15

eFigure 1: Study selection and inclusion flow chart

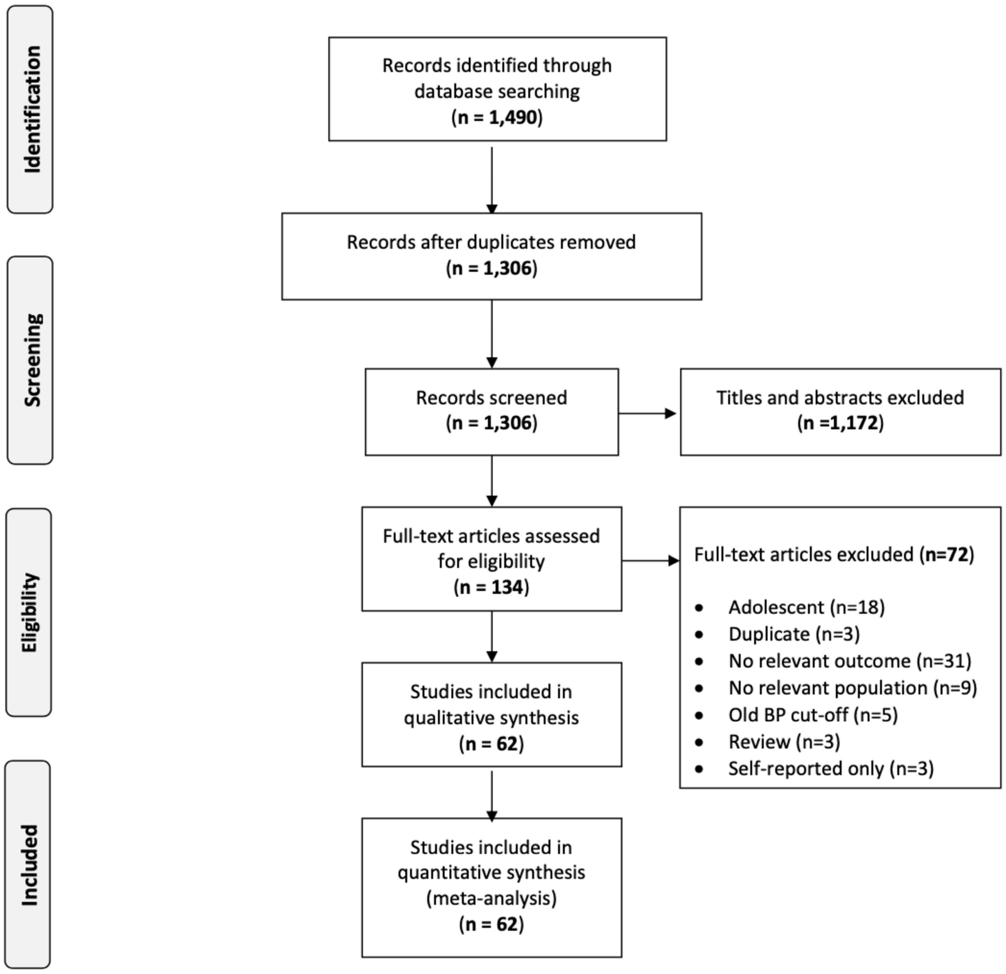

Uthman OA, *et al. BMJ Open* 2022; 12:e052393. doi: 10.1136/bmjopen-2021-052393

Box 1: Study selection and inclusion flow chart

**Domain**	**Details**	**Risk of bias**
Selection of participants	Selection bias caused by the inadequate selection of participants	- Low<br>- High<br>- Unclear
Confounding variables	Selection bias caused by the inadequate confirmation and consideration of confounding variable	- Low<br>- High<br>- Unclear
Measurement of exposure	Performance bias caused by the inadequate measurement of exposure	- Low<br>- High<br>- Unclear
Blinding of outcome assessments	Detection bias caused by the inadequate blinding of outcome assessments	- Low<br>- High<br>- Unclear
Incomplete outcome data	Attrition bias caused by the inadequate handling of incomplete outcome data	- Low<br>- High<br>- Unclear
Selective outcome reporting	Reporting bias caused by the selective reporting of outcomes	- Low<br>- High<br>- Unclear

Uthman OA, *et al. BMJ Open* 2022; 12:e052393. doi: 10.1136/bmjopen-2021-052393

eTable 1: List of Excluded Studies

s/n	Study	Reason
1	Maiti 2016[1]	Adolescent
2	Khopkar 2015[2]	Adolescent
3	Paul 2013[3]	Adolescent
4	Kamath 2012[4]	Adolescent
5	Simsek 2012[5]	Adolescent
6	Saha 2011[6]	Adolescent
7	Oria 2010[7]	Adolescent
8	Saha 2008[8]	Adolescent
9	Saha 2008[9]	Adolescent
10	Sesso 2004[10]	Adolescent
11	Fernandes 2003[11]	Adolescent
12	Zeelie 2010[12]	Adolescent
13	Soudrassanane 2008[13]	Adolescent
14	Werner 2015[14]	Duplicate
15	van de Vijver 2016[15]	Duplicate
16	Haregu 2016[16]	Duplicate
17	Ezenwaka 1997[17]	Old BP cut-off
18	Suriyawongpaisal 1993[18]	Old BP cut-off
19	Suriyawongpaisal 1991[19]	Old BP cut-off
20	Sitthi-Amornn 1989[20]	Old BP cut-off
21	Bunnag 1990[21]	Old BP cut-off
22	E. Sharmin Trisha 2016[22]	No relevant outcome
23	Bhandari 2015[23]	No relevant outcome
24	Oti 2014[24]	No relevant outcome
25	Hiremath 2014[25]	No relevant outcome
26	Joshi 2013[26]	No relevant outcome
27	van de Vijver 2013[27]	No relevant outcome
28	Itrat 2011[28]	No relevant outcome
29	Ahmed 2011[29]	No relevant outcome
30	Haregu 2015[30]	No relevant outcome
31	van de Vijver 2015[31]	No relevant outcome
32	Kohli 2016[32]	No relevant outcome
33	Mudgapalli 2016[33]	No relevant population
34	Natarajan 2014[34]	No relevant population
35	Kumaramanickavel 2014[35]	No relevant population
36	Kumaramanickavel 2015[36]	No relevant population
37	Hulzebosch 2015[37]	No relevant population
38	Madhu 2016[38]	No relevant population
39	Mugure 2014[39]	No relevant population
40	Mukhopadhyay 2012[40]	No relevant population
41	Khan 2010[41]	No relevant population
42	Etyang 2013[42]	Review
43	Dhar 2014[43]	Review
44	Bhargava 1991[44]	Review
46	Kien 2015[45]	Self-reported only
47	Sur 2007[46]	Self-reported only
48	Thakur 2013[47]	Self-reported only
49	Ahmedani 2019[48]	No relevant outcome
50	Ashe 2019[49]	No relevant outcome
51	Asiki 2018[50]	No relevant outcome
52	Bagdey 2019[51]	No relevant outcome
53	Cope 2020[52]	No relevant outcome
54	De Silva 2018[53]	No relevant outcome
55	Kapwata 2018[54]	No relevant outcome
56	Kawazoe 2018 [55]	No relevant outcome

Uthman OA, *et al. BMJ Open* 2022; 12:e052393. doi: 10.1136/bmjopen-2021-052393

57	Khanam 2019[56]	No relevant outcome
58	Kolak 2018[57]	No relevant outcome
59	Korn 2018[58]	No relevant outcome
60	Kotian 2019[59]	No relevant outcome
61	Kumar 2018[60]	No relevant outcome
62	Ma 2018[61]	No relevant outcome
63	Maharana 2019[62]	No relevant outcome
64	Nagarkar 2018[63]	No relevant outcome
65	Narendran 2018[64]	No relevant outcome
66	Rajapakshe 2018[65]	No relevant outcome
67	Sarkar 2019[66]	No relevant outcome
68	Scazufca 2019[67]	No relevant outcome
69	Wang 2018[68]	No relevant outcome
70	Wekasah 2020[69]	No relevant outcome
71	Wilson 2020[70]	No relevant outcome
72	Yadav 2018[71]	No relevant outcome
73	Zhang 2019[72]	No relevant outcome

Uthman OA, *et al. BMJ Open* 2022; 12:e052393. doi: 10.1136/bmjopen-2021-052393

List of excluded studies

1. Maiti M, Bandyopadhyay L. Variation in blood pressure among adolescent schoolchildren in an urban slum of Kolkata, West Bengal. *Postgraduate Medical Journal (no pagination), 2016* 2016;Date of Publication:July 25. doi: http://dx.doi.org/10.1136/postgradmedj-2016-134227
2. Khopkar SA, Virtanen SM, Kulathinal S. Mental health, anthropometry and blood pressure among adolescents living in slums of Nashik, India. *Tanzania Journal of Health Research* 2015;17(4) doi: http://dx.doi.org/10.4314/thrb.v17i4.6
3. Paul B, Saha I, Mukherjee A. Adolescent Hypertension and Family History. *Pakistan Paediatric Journal* 2013;37(3):177-79.
4. Kamath N, Goud BR, Phadke KD, et al. Use of oscillometric devices for the measurement of blood pressure-comparison with the gold standard. *Indian Journal of Pediatrics* 2012;79(9):1230-32. doi: http://dx.doi.org/10.1007/s12098-011-0600-0
5. Simsek E, Selver B, Dallar Y, et al. Obesity epidemiology in children living in the lower socio-economic status. *Hormone Research in Paediatrics* 2012;Conference:51st Annual Meeting of the European Society for Paediatric Endocrinology. doi: http://dx.doi.org/10.1159/000343184
6. Saha I, Paul B, Mukherjee A, et al. Validity of the WHO criteria for adolescent hypertension. *East African journal of public health* 2011;8(2):135-37.
7. Oria RB, Patrick PD, Oria MOB, et al. ApoE polymorphisms and diarrheal outcomes in Brazilian shanty town children. *Brazilian Journal of Medical and Biological Research* 2010;43(3):249-56.
8. Saha I, Paul B, Dasgupta A. Prevalence of hypertension and variation of blood pressure with age among adolescents in Chetla, India. *Tanzania journal of health research* 2008;10(2):108-11.
9. Saha I, Paul B, Dasgupta A, et al. Variations of adolescent blood pressure by multifactorial analysis in an urban slum of Kolkata. *Journal of the Indian Medical Association* 2008;106(9)
10. Sesso R, Barreto GP, Neves J, et al. Malnutrition is associated with increased blood pressure in childhood. *Nephron Clinical Practice* 2004;97(2):c61-c66. doi: http://dx.doi.org/10.1159/000078402
11. Fernandes MTB, Sesso R, Martins PA, et al. Increased blood pressure in adolescents of low socioeconomic status with short stature. *Pediatric Nephrology* 2003;18(5):435-39.
12. Zeelie A, Moss SJ, Kruger HS. The relationship between body composition and selected metabolic syndrome markers in black adolescents in South Africa: the PLAY study. *Nutrition* 2010;26(11-12):1059-64. doi: 10.1016/j.nut.2010.03.001 [published Online First: 2010/06/15]
13. Soudarssanane M, Mathanraj S, Sumanth M, et al. Tracking of blood pressure among adolescents and young adults in an urban slum of puducherry. *Indian journal of community medicine : official publication of Indian Association of Preventive & Social Medicine* 2008;33(2):107-12. doi: 10.4103/0970-0218.40879 [published Online First: 2008/04/01]
14. Werner ME, van de Vijver S, Adhiambo M, et al. Results of a hypertension and diabetes treatment program in the slums of Nairobi: a retrospective cohort study. *BMC health services research* 2015;15(pp 512) doi: http://dx.doi.org/10.1186/s12913-015-1167-7
15. van de Vijver S, Oti SO, Gomez GB, et al. Impact evaluation of a community-based intervention for prevention of cardiovascular diseases in the slums of Nairobi: the SCALE-UP study. *Glob Health Action* 2016;9(1):30922. doi: 10.3402/gha.v9.30922 [published Online First: 2017/02/06]

16. Haregu TN, Oti S, Egondi T, et al. Measurement of overweight and obesity an urban slum setting in sub-Saharan Africa: a comparison of four anthropometric indices. *BMC obesity* 2016;3:46. doi: 10.1186/s40608-016-0126-0 [published Online First: 2016/11/12]
17. Ezenwaka CE, Akanji AO, Akanji BO, et al. The prevalence of insulin resistance and other cardiovascular disease risk factors in healthy elderly southwestern Nigerians. *Atherosclerosis* 1997;128(2):201-11. doi: http://dx.doi.org/10.1016/S0021-9150%2896%2905991-6
18. Suriyawongpaisal P, Underwood P. Situation of hypertension in some Bangkok slums. *Journal of the Medical Association of Thailand = Chotmaihet thangphaet* 1993;76(3):123-28.
19. Suriyawongpaisal P, Underwood P, Rouse IL, et al. An investigation of hypertension in a slum of Nakhon Ratchasima. *The Southeast Asian journal of tropical medicine and public health* 1991;22(4):586-94.
20. Sitthi-Amorn C, Chandraprasert S, Bunnag SC, et al. The prevalence and risk factors of hypertension in Klong Toey Slum and Klong Toey government apartment houses. *International Journal of Epidemiology* 1989;18(1):89-94.
21. Bunnag SC, Sitthi-Amorn C, Chandraprasert S. The prevalence of obesity, risk factors and associated diseases in Klong Toey slum and Klong Toey government apartment houses. *Diabetes Res Clin Pract* 1990;10(1)
22. N EST, Jelinek HF, Tarvainen MP, et al. Socioeconomic status, age and heart rate variability in a Bangladeshi community. *Conference proceedings : Annual International Conference of the IEEE Engineering in Medicine and Biology Society IEEE Engineering in Medicine and Biology Society Annual Conference* 2016;01 doi: http://dx.doi.org/10.1109/EMBC.2016.7591919
23. Bhandari S, Sarma PS, Thankappan KR. Adherence to antihypertensive treatment and its determinants among urban slum dwellers in Kolkata, India. *Asia Pacific journal of public health / Asia Pacific Academic Consortium for Public Health* 2015;27(2) doi: http://dx.doi.org/10.1177/1010539511423568
24. Oti SO, van de Vijver S, Kyobutungi C. Trends in non-communicable disease mortality among adult residents in Nairobi's slums, 2003-2011: applying InterVA-4 to verbal autopsy data. *Global health action* 2014;7(pp 25533) doi: http://dx.doi.org/10.3402/gha.v7.25533
25. Hiremath RN, Venkatesh G, Sharvesh, et al. Hypertension status and awareness among geriatric population living in Urban slum. *Nepal Journal of Epidemiology* 2014;Conference:International Conference on Research Methodology and Scientific Writing.
26. Joshi A, Mehta S, Grover A, et al. Knowledge, attitude, and practices of individuals to prevent and manage metabolic syndrome in an Indian setting. *Diabetes Technology and Therapeutics* 2013;15(8):644-53. doi: http://dx.doi.org/10.1089/dia.2012.0309
27. van de Vijver SJ, Oti SO, Agyemang C, et al. Prevalence, awareness, treatment and control of hypertension among slum dwellers in Nairobi, Kenya. *Journal of hypertension* 2013;31(5):1018-24. doi: 10.1097/HJH.0b013e32835e3a56 [published Online First: 2013/02/22]
28. Itrat A, Ahmed B, Khan M, et al. Risk factor profiles of South Asians with cerebrovascular disease. *International Journal of Stroke* 2011;6(4):346-48. doi: http://dx.doi.org/10.1111/j.1747-4949.2011.00622.x
29. Ahmed B, Itrat A, Khan M, et al. Risk factor profiles of south asians with cerebrovascular disease: Findings from a community-based prevalence study in semiurban Pakistan. *Circulation: Cardiovascular Quality and Outcomes* 2011;Conference:Quality of Care

and Outcomes Research in Cardiovascular Disease and Stroke 2011 Scientific Sessions.
30. Haregu TN, Oti S, Egondi T, et al. Co-occurrence of behavioral risk factors of common non-communicable diseases among urban slum dwellers in Nairobi, Kenya. *Glob Health Action* 2015;8(28697) doi: https://dx.doi.org/10.3402/gha.v8.28697
31. van de Vijver S, Oti S, Moll van Charante E, et al. Cardiovascular prevention model from Kenyan slums to migrants in the Netherlands. *Global health* 2015;11(11):07. doi: https://dx.doi.org/10.1186/s12992-015-0095-y
32. Kohli C, Gupta K. LBOS 03-03 ECONOMIC IMPACT OF HYPERTENSION. *Journal of hypertension* 2016;34 Suppl 1 - ISH 2016 Abstract Book:e551-e52. doi: 10.1097/01.hjh.0000501509.98288.ad [published Online First: 2016/10/19]
33. Mudgapalli V, Sharan S, Amadi C, et al. Perception of receiving SMS based health messages among hypertensive individuals in urban slums. *Technology and Health Care* 2016;24(1):57-65. doi: http://dx.doi.org/10.3233/THC-151097
34. Natarajan S, Mohan S, Satagopan U, et al. Elderly patients with T2DM should be periodically screened for diabetic retinopathy and its complications to reduce visual morbidity - A study from slums of Western India. *Investigative Ophthalmology and Visual Science* 2014;Conference:2014 Annual Meeting of the Association for Research in Vision and Ophthalmology.
35. Kumaramanickavel G, Mohan S, Satagopan U, et al. Diabetic retinopathy in urban slums of Mumbai, India - Social, lifestyle, clinical and genetic risk factors. *Investigative Ophthalmology and Visual Science* 2014;Conference:2014 Annual Meeting of the Association for Research in Vision and Ophthalmology.
36. Kumaramanickavel G, Mohan S, Kumar Singh A, et al. AJDRUMSS-diabetic retinopathy prevalence study in Mumbai slums of India-association of demographic, genetic and medical risk factors. *Investigative Ophthalmology and Visual Science* 2015;Conference:2015 Annual Meeting of the Association for Research in Vision and Ophthalmology.
37. Hulzebosch A, van de Vijver S, Oti SO, et al. Profile of people with hypertension in Nairobi's slums: a descriptive study. *Globalization and health* 2015;11(pp 26) doi: http://dx.doi.org/10.1186/s12992-015-0112-1
38. Madhu B, Srinath KM, Chandresh S, et al. Quality of diabetic care in an urban slum area of Mysore: A community based study. *Diabetes and Metabolic Syndrome: Clinical Research and Reviews* 2016 doi: http://dx.doi.org/10.1016/j.dsx.2016.03.014
39. Mugure G, Karama M, Kyobutungi C, et al. Correlates for cardiovascular diseases among diabetic/hypertensive patients attending outreach clinics in two Nairobi slums, Kenya. *Pan African Medical Journal* 2014;19(no pagination) doi: http://dx.doi.org/10.11604/pamj.2014.19.261.5261
40. Mukhopadhyay A, Sundar U, Adwani S, et al. Prevalence of stroke and post-stroke cognitive impairment in the elderly in Dharavi, Mumbai. *Journal of Association of Physicians of India* 2012;60(10):29-32.
41. Khan RMA, Ahmad M. To assess the public awareness about obesity among adult populace of lahore. *Pakistan Journal of Medical and Health Sciences* 2010;4(4)
42. Etyang A, Harding S, Cruickshank JK. Slum living and hypertension in tropical settings: Neglected issue, statistical artifact or surprisingly slight? Insights amidst adversity. *Journal of Hypertension* 2013;31(5):877-79. doi: http://dx.doi.org/10.1097/HJH.0b013e32836103fb
43. Dhar L. Preventing coronary heart disease risk of slum dwelling residents in India. *Journal of family medicine and primary care* 2014;3(1):58-62. doi: 10.4103/2249-4863.130278 [published Online First: 2014/05/03]

44. Bhargava SK, Singh KK, Saxena BN. ICMR Task Force National Collaborative Study on Identification of High Risk Families, Mothers and Outcome of their Off-springs with particular reference to the problem of maternal nutrition, low birth weight, perinatal and infant morbidity and mortality in rural and urban slum communities. Summary, conclusions and recommendations. *Indian pediatrics* 1991;28(12):1473-80. [published Online First: 1991/12/01]
45. Kien VD, Van Minh H, Giang KB, et al. Socioeconomic inequalities in self-reported chronic non-communicable diseases in urban Hanoi, Vietnam. *Global Public Health* 2015 doi: http://dx.doi.org/10.1080/17441692.2015.1123282
46. Sur D, Mukhopadhyay SP. A study on smoking habits among slum dwellers and the impact on health and economics. *Journal of the Indian Medical Association* 2007;105(9):492-98.
47. Thakur R, Banerjee A, Nikumb V. Health problems among the elderly: a cross-sectional study. *Annals of medical and health sciences research* 2013;3(1):19-25. doi: 10.4103/2141-9248.109466 [published Online First: 2013/05/02]
48. Ahmedani MY, Fawwad A, Shaheen F, et al. Optimized health care for subjects with type 1 diabetes in a resource constraint society: A three-year follow-up study from Pakistan. *World J Diabetes* 2019;10(3):224-33. doi: 10.4239/wjd.v10.i3.224
49. Ashe S, Routray D. Prevalence, associated risk factors of depression and mental health needs among geriatric population of an urban slum, Cuttack, Odisha. *International Journal of Geriatric Psychiatry* 2019;34(12):1799-807. doi: 10.1002/gps.5195
50. Asiki G, Mohamed SF, Wambui D, et al. Sociodemographic and behavioural factors associated with body mass index among men and women in Nairobi slums: AWI-Gen Project. *Global health action* 2018;11(sup2):1470738-38. doi: 10.1080/16549716.2018.1470738
51. Bagdey PS, Ansari JA, Barnwal RK. Prevalence and epidemiological factors associated with hypertension among post-menopausal women in an urban area of central India. *Clinical Epidemiology and Global Health* 2019;7(1):111-14. doi: 10.1016/j.cegh.2018.02.008
52. Cope AB, Edmonds A, Ludema C, et al. Neighborhood Poverty and Control of HIV, Hypertension, and Diabetes in the Women's Interagency HIV Study. *AIDS Behav* 2020;24(7):2033-44. doi: 10.1007/s10461-019-02757-5
53. De Silva AP, De Silva SHP, Haniffa R, et al. Inequalities in the prevalence of diabetes mellitus and its risk factors in Sri Lanka: a lower middle income country. *Int J Equity Health* 2018;17(1):45-45. doi: 10.1186/s12939-018-0759-3
54. Kapwata T, Manda S. Geographic assessment of access to health care in patients with cardiovascular disease in South Africa. *BMC health services research* 2018;18(1):197-97. doi: 10.1186/s12913-018-3006-0
55. Kawazoe N, Zhang X, Chiang C, et al. Prevalence of hypertension and hypertension control rates among elderly adults during the cold season in rural Northeast China: a cross-sectional study. *J Rural Med* 2018;13(1):64-71. doi: 10.2185/jrm.2959 [published Online First: 2018/05/29]
56. Khanam F, Hossain MB, Mistry SK, et al. Prevalence and Risk Factors of Cardiovascular Diseases among Bangladeshi Adults: Findings from a Cross-sectional Study. *J Epidemiol Glob Health* 2019;9(3):176-84. doi: 10.2991/jegh.k.190531.001
57. Kolak M, Bradley M, Block DR, et al. Urban foodscape trends: Disparities in healthy food access in Chicago, 2007–2014. *Health & Place* 2018;52:231-39. doi: 10.1016/j.healthplace.2018.06.003

58. Korn A, Bolton SM, Spencer B, et al. Physical and Mental Health Impacts of Household Gardens in an Urban Slum in Lima, Peru. *Int J Environ Res Public Health* 2018;15(8):1751. doi: 10.3390/ijerph15081751
59. Kotian S, Waingankar P, Mahadik V. Assessment of compliance to treatment of hypertension and diabetes among previously diagnosed patients in urban slums of Belapur, Navi Mumbai, India. *Indian Journal of Public Health* 2019;63(4):348. doi: 10.4103/ijph.ijph_422_18
60. Kumar R, Kaur N, Pilania M. Morbidity Pattern of Patients Attending a Primary Healthcare Facility in an Urban Slum of Chandigarh, India. *JOURNAL OF CLINICAL AND DIAGNOSTIC RESEARCH* 2018 doi: 10.7860/jcdr/2018/31331.11297
61. Ma C. The prevalence of depressive symptoms and associated factors in countryside-dwelling older Chinese patients with hypertension. *Journal of Clinical Nursing* 2018;27(15-16):2933-41. doi: 10.1111/jocn.14349
62. Maharana S, Garg S, Dasgupta A, et al. A study on impact of oral health on general health among the elderly residing in a slum of Kolkata: A cross-sectional study. *Indian Journal of Dental Research* 2019;30(2):164. doi: 10.4103/ijdr.ijdr_491_17
63. Nagarkar AM, Kulkarni SS. Obesity and its Effects on Health in Middle-Aged Women from Slums of Pune. *J Midlife Health* 2018;9(2):79-84. doi: 10.4103/jmh.JMH_8_18
64. Narendran M, Rani BBS, Kulkarni P, et al. Interdependence of communicable and Non-Communicable diseases among elderly population in declared slum in Mysuru City, Karnataka. *Indian Journal of Public Health Research & Development* 2018;9(11):62. doi: 10.5958/0976-5506.2018.01426.2
65. Rajapakshe OBW, Sivayogan S, Kulatunga PM. Prevalence and correlates of depression among older urban community-dwelling adults in Sri Lanka. *Psychogeriatrics* 2018;19(3):202-11. doi: 10.1111/psyg.12389
66. Sarkar A, Roy D, Chauhan MM, et al. A lay epidemiological study on coexistent stress in hypertension: Its prevalence, risk factors, and implications in patients' lives. *Journal of family medicine and primary care* 2019;8(3):966-71. doi: 10.4103/jfmpc.jfmpc_60_19
67. Scazufca M, de Paula Couto MCP, Henrique MG, et al. Pilot study of a two-arm non-randomized controlled cluster trial of a psychosocial intervention to improve late life depression in socioeconomically deprived areas of São Paulo, Brazil (PROACTIVE): feasibility study of a psychosocial intervention for late life depression in São Paulo. *BMC public health* 2019;19(1):1152-52. doi: 10.1186/s12889-019-7495-5
68. Wang H, Su M, Fang P-q, et al. Analysis on Medical Expenses of Hypertensive Inpatients in Urban Areas from 2010 to 2013—Evidence from Two Provinces in South of China. *Current Medical Science* 2018;38(4):741-48. doi: 10.1007/s11596-018-1939-5
69. Wekesah FM, Klipstein-Grobusch K, Grobbee DE, et al. Determinants of Mortality from Cardiovascular Disease in the Slums of Nairobi, Kenya. *Glob Heart* 2020;15(1):33-33. doi: 10.5334/gh.787
70. Wilson V, Nittoori S. Risk of type 2 diabetes mellitus among urban slum population using Indian Diabetes Risk Score. *Indian Journal of Medical Research* 2020;152(3):308. doi: 10.4103/ijmr.ijmr_1597_18
71. Yadav S, Saraswat N, Saini AK, et al. A REVIEW ON THE PREVALENCE OF HYPERTENSION IN SIDE-LINED POPULATIONS; SLUM DWELLERS, SHIFT JOB WORKERS AND OCCUPATIONAL NOISE AFFECTED WORKERS: ATTRIBUTABLE TO LIFESTYLE AND ENVIRONMENTAL FACTOR. *Asian Journal of Pharmaceutical and Clinical Research* 2018;11(10):18. doi: 10.22159/ajpcr.2018.v11i10.27007

72. Zhang X, Chen X, Gong W. Type 2 diabetes mellitus and neighborhood deprivation index: A spatial analysis in Zhejiang, China. *J Diabetes Investig* 2019;10(2):272-82. doi: 10.1111/jdi.12899 [published Online First: 2018/08/28]

eTable 2: Characteristics of included studies

Study	Country	Slum	Sample size	Age group	% female
Acharyya (2014)	India	North-Parganas	1052	25-64	49.8
Ahmad (2014)	India	Meerut	196	>60	50
Akinwale (2013)	Nigeria	Ijora Oloye, Ajegunle & Makoko	2434		
Anand (2007)	India	Faridabad	2562	15+	50.9
Ayah (2013)	Kenya		2061	18-90	49.1
Banerjee (2016)	India	Kolkata	10167	>20 years	60
Chakerborty (2012)	India	Kolkata	470	18-60	0
Chaturvedi (2007)	India	Delhi	596	>20	
Daniel (2013)	Nigeria	Ajegunle	964	20-81	65.8
Dasappa (2015)	India	Bangalore	2013	35+	50.8
Deepa (2011)	India	Ballabgarh, Delhi, Chennai, Trivandrum , Dibrugarh and Nagpur	15763	15-64	
Edwards (2015)	Kenya	Kibera			
Ezeala-Adikaibe (2016)	Nigeria	Enugu	774	⩾ 20	64.7
Ferreira (2005)	Brazil	Maceio	223	18-65	100
Florencio (2004)	Brazil	Maceio	416	18-60	57
Haregu (2016)	Kenya	Nairobi	5190	18+	46.2
Heitzinger (2014)	Peru	Lima	142	18-81	69.7
Huda (2012)	Bangladesh	Mirpur, Dhaka	1000	15-65	33.4
Jalil (2008)	Pakistan	Lahore	695		43.6
Joshi (2013)	India	Rourkela & Bhubaneswar	100	>18	69
Joshi (2014)	Kenya	Kibera	2045	18-90	49.1
Kar (2008)	India	Chandigarh & Haryana	1010	>30	58.9
Kar (2010)	India	Chandigarh & Haryana	150	>30	62
Khalequzzaman (2017)	Bangladesh	Dhakar	2551	18+	46.7
Kumari (2014)	India	Hyderabad	250		78
Lubree (2002)	India	Pune	150	30-50	100
Marins (2007)	Brazil	Rio-de-Janeiro	3279	>20	56.9
Misra (2001)	India	Gautam-Nagar, Delhi	532		68
Nirmala (2014)	India	Hyderabad, Telangana	700	>20	50.8
Olack (2015)	Kenya	Kibera	1528	35-64	58.1
Oli (2013)	Nepal	Kathmandu	689	15-64	58.9
Ongeti (2013)	Kenya	Kibera	400	14-75	70.3
Oti (2013)	Kenya	Viwandani & Korogocho		18+	46
Patil (2016)	India	Pune, Maharashtra	425	20+	
Rahim (2004)	Bangladesh	Dhakar	1555	20+	52.99
Rawal (2017)	Bangladesh	Dhaka	507		50
Sayeed (2007)	Bangladesh	Dhakar			59.2
Singh (b) (2012)	India	Delhi	474	60+	48
Singh (2012)	India	Patna	3118	>30	56.5
Sinha (2010)	India	Gokulpuri	275	18-40	100
Sithi-Amorn (1989)	Thailand	Klong-Toey	976		54.7

Uthman OA, *et al. BMJ Open* 2022; 12:e052393. doi: 10.1136/bmjopen-2021-052393

Snyder (2017)	Brazil		792		64.5
Sowemimo (2015)	Nigeria	Yemetu, Ibadan	806	18-90	
Sunita (2017)	India	Mumbai	6464	>40	
Unger (2015)	Brazil	Salvador	5649	>18	58.3
Uthakalla (2012)	India	Hyderabad		20-60	56
Vigneswari (2014)	India	Chennai	529	18+	77.3
Vigneswari (2015)	India		529	18+	77.3
Vikram (2003)	India	New-Delhi	639		73.4
Wasir (2007)	India	Delhi	278		
Yajnik (2008)	India		142	30-50	0
van de Vijver (2013)	Kenya	Viwandani & Korogocho	5190	>18	46.2
Bawah (2019)	Ghana	Accra	2009		
Chiang (2019)	Bangladesh	Dhaka	423		
Choudhury (2018)	Bangladesh	Dhaka	984	43.4	73
Dwivedi (2018)	India	Bangalore			
Gadallah (2018)	Egypt	West Delhi			
George (2019)	India	Bangalore		57.6	
Gonmei (2018)	India	Delhi			
Jain (2019)	India	Delhi	984	43.4	73
Tymejczyk (2019)	Haiti	Gurugram	420		
Vusirikala (2019)	Kenya	Nairobi		57.6	

eTable 3: Risk of bias of included studies

Study	Selection of participants	Confounding variables	Measurement of exposure	Blinding of outcome assessments	Incomplete outcome data	Selective outcome reporting
Acharyya (2014)	Low risk	Low risk	Low risk	Low risk	Low risk	Low risk
Ahmad (2014)	Low risk	High risk	Low risk	Low risk	Unclear risk	Low risk
Akinwale (2013)	Low risk	High risk	Low risk	Low risk	Low risk	Low risk
Anand (2007)	Low risk	Low risk	Low risk	Low risk	Low risk	Low risk
Ayah (2013)	Low risk	Low risk	Low risk	Low risk	Low risk	Low risk
Banerjee (2016)	Low risk	Low risk	Low risk	Low risk	Unclear risk	Low risk
Chakerborty (2012)	High risk	High risk	Low risk	Low risk	Low risk	Low risk
Chaturvedi (2007)	Low risk	Low risk	Low risk	Low risk	Low risk	Low risk
Daniel (2013)	Low risk	Low risk	Low risk	Low risk	Low risk	Low risk
Dasappa (2015)	Low risk	High risk	Low risk	Low risk	Low risk	Low risk
Deepa (2011)	Low risk	High risk	Low risk	Low risk	Low risk	Low risk
Edwards (2015)	Low risk	High risk	Low risk	Low risk	Low risk	Low risk
Ezeala-Adikaibe (2016)	High risk	Low risk	Low risk	Low risk	High risk	Low risk
Ferreira (2005)	Low risk	Low risk	Low risk	Low risk	Low risk	Low risk
Florencio (2004)	Low risk	Low risk	Low risk	Low risk	Low risk	Low risk
Haregu (2016)	Unclear risk	Low risk	Low risk	Low risk	Unclear risk	Low risk
Heitzinger (2014)	Low risk	Low risk	Low risk	Low risk	Low risk	Low risk
Huda (2012)	Low risk	High risk	Low risk	Low risk	Low risk	Low risk
Jalil (2008)	Low risk	Low risk	Low risk	Low risk	Low risk	Low risk
Joshi (2013)	High risk	Low risk	Low risk	Low risk	Low risk	Low risk
Joshi (2014)	Low risk	Low risk	Low risk	Low risk	Low risk	Low risk
Kar (2008)	Low risk	Low risk	Low risk	Low risk	Low risk	Low risk
Kar (2010)	Low risk	Low risk	Low risk	Low risk	Low risk	Low risk
Khalequzzaman (2017)	Low risk	Low risk	Low risk	Low risk	Low risk	Low risk
Kumari (2014)	Low risk	High risk	Low risk	Low risk	Low risk	Low risk
Lubree (2002)	Low risk	High risk	Low risk	Low risk	Low risk	Low risk
Marins (2007)	Low risk	High risk	Low risk	Low risk	Low risk	Low risk
Misra (2001)	Low risk	High risk	Low risk	Low risk	Low risk	Low risk
Nirmala (2014)	Low risk	High risk	Low risk	Low risk	Low risk	Low risk
Olack (2015)	Low risk	Low risk	Low risk	Low risk	Low risk	Low risk
Oli (2013)	Low risk	Low risk	Low risk	Low risk	Low risk	Low risk
Ongeti (2013)	Low risk	Low risk	Low risk	Low risk	Low risk	Low risk
Oti (2013)	Low risk	Low risk	Low risk	Low risk	Low risk	Low risk
Patil (2016)	Low risk	High risk	Low risk	Low risk	Low risk	Low risk
Rahim (2004)	Low risk	High risk	Low risk	Low risk	Low risk	Low risk
Rawal (2017)	Low risk	Low risk	Low risk	Low risk	Low risk	Low risk
Sayeed (2007)	Low risk	High risk	Low risk	Low risk	Low risk	Low risk
Singh (b) (2012)	Low risk	Low risk	Low risk	Low risk	Low risk	Low risk
Singh (2012)	Low risk	Low risk	Low risk	Low risk	Low risk	Low risk

Study	Selection of participants	Confounding variables	Measurement of exposure	Blinding of outcome assessments	Incomplete outcome data	Selective outcome reporting
Sinha (2010)	Low risk	Low risk	Low risk	Low risk	Low risk	Low risk
Sithi-Amorn (1989)	Low risk	High risk	Low risk	Low risk	Low risk	Low risk
Snyder (2017)	Low risk	Low risk	Low risk	Low risk	Low risk	Low risk
Sowemimo (2015)	Low risk	Low risk	Low risk	Low risk	Unclear risk	Low risk
Sunita (2017)	Low risk	High risk	Low risk	Low risk	Low risk	Low risk
Unger (2015)	Low risk	Low risk	Low risk	Low risk	Low risk	Low risk
Uthakalla (2012)	Low risk	High risk	Low risk	Low risk	Low risk	Low risk
Vigneswari (2014)	Low risk	High risk	Low risk	Low risk	Low risk	Low risk
Vigneswari (2015)	Low risk	High risk	Low risk	Low risk	Low risk	Low risk
Vikram (2003)	Low risk	Low risk	Low risk	Low risk	Low risk	Low risk
Wasir (2007)	Low risk	High risk	Low risk	Low risk	High risk	Low risk
Yajnik (2008)	Low risk	High risk	Low risk	Low risk	Low risk	Low risk
van de Vijver (2013)	Low risk	Low risk	Low risk	Low risk	Low risk	Low risk
Bawah (2019)	Unclear risk	Low risk	Low risk	Low risk	Unclear risk	Low risk
Chiang (2019)	Low risk	Low risk	Low risk	Low risk	Low risk	Low risk
Choudhury (2018)	Low risk	Low risk	Low risk	Low risk	Low risk	Low risk
Dwivedi (2018)	Low risk	Low risk	Low risk	Low risk	Low risk	Low risk
Gadallah (2018)	Low risk	Low risk	Low risk	Low risk	Low risk	Low risk
George (2019)	Low risk	Low risk	Low risk	Low risk	Low risk	Low risk
Gonmei (2018)	Unclear risk	Unclear risk	Low risk	Low risk	Unclear risk	Low risk
Jain (2019)	Low risk	Low risk	Low risk	Low risk	Low risk	Low risk
Tymejczyk (2019)	Low risk	Low risk	Low risk	Low risk	Low risk	Low risk
Vusirikala (2019)	Low risk	Low risk	Low risk	Low risk	Low risk	Low risk

Uthman OA, *et al. BMJ Open* 2022; 12:e052393. doi: 10.1136/bmjopen-2021-052393

Annex 1: MEDLINE Search Strategy

1 exp hypertension/
2 hypertens$.mp.
3 exp blood pressure/
4 (blood pressure or bloodpressure).mp.
5 (essential adj3 hypertension).ti,ab.
6 (isolat* adj3 hypertension).ti,ab.
7 (elevat* adj3 blood adj pressur*).ti,ab.
8 (high adj3 blood adj pressur*).ti,ab.
9 (increase* adj3 blood pressur*).ti,ab.
10 ((systolic or diastolic or arterial) adj3 pressur*).ti,ab.
11 essential hypertension.mp.
12 isolated hypertension.mp.
13 elevated blood pressure.mp.
14 high blood pressure.mp.
15 increase blood pressure.mp.
16 diastolic pressure.mp.
17 pre-hypertension.mp.
18 pre-hypertensive.mp.
19 prehypertension.mp.
20 prehypertensive.mp.
21 arterial pressure.mp.
22 cardiovascular diseases/
23 exp coronary disease/
24 cardiovascular risk factor$.tw.
25 (cardiovascular adj3 disease$).tw.
26 (Coronary adj3 disease$).tw.
27 heart disease$.tw.
28 coronary risk factor$.tw.
29 or/1-28

1 exp Diabetes Mellitus, Type 2/
2 exp DIABETES MELLITUS/
3 T2DM.ti,ab.
4 (Type* adj3 ("2" or "II" or two*) adj3 (diabete* or diabetic*)).tw.
5 ((Maturit* or adult* or slow*) adj3 onset* adj3 (diabete* or diabetic*)).tw.
6 ((Ketosis-resistant* or stable*) adj3 (diabete* or diabetic*)).tw.
7 ((Non-insulin* or Non insulin* or Noninsulin*) adj3 depend* adj3 (diabete* or diabetic*)).tw.
8 IDDM.ti,ab.
9 diabet$.ti.
10 PREDIABETIC STATE/
11 prediabet$.ti,ab.
12 impaired glucose tolerance.ti,ab.
13 IGT.ti,ab.
14 Impaired fasting glucose.ti,ab.
15 IFG.ti,ab.
16 Impaired glucose regulation.ti,ab. 1
17 IGR.ti,ab.
18 GLUCOSE INTOLERANCE/
19 (diabet* or glucose or hyperglycaemia or hyperglycaemia or postprandial or post-prandial or insulin or hypoglycemia or hypoglycaemia or IGT or OGTT or CGMS).tw.
20 (subclinical diabetes" or "subclinical diabetic" or "sub-clinical diabetes" or "sub-clinical diabetic").tw.
21 or/1-20

22 (baladi or bandas de miseria or barraca or barrio marginal or barrio or bidonville or brarek or bustee or chalis or chereka bete or dagatan or estero or favela or galoos or gecekondu or hrushebi).mp.
23 (ishash or karyan or katras or looban or loteamento or medina achouaia or morro or mudun safi or musseque or solares or tanake or taudis or township or tugurio or udukku or umjondolo or watta or zopadpattis).mp.
24 (slum or slums or ghetto or ghettos or informal settlement$ or shantytown$ or shanty town$).mp.
25 slum/
26 ghetto/
27 or/22-26

Uthman OA, *et al. BMJ Open* 2022; 12:e052393. doi: 10.1136/bmjopen-2021-052393
