# Supplementary material for: Global prevalence and trends in hypertension and type 2 diabetes mellitus among slum residents: a systematic review and meta-analysis
Source: BMJ Open. 2022 Feb 24;12(2):e052393. doi: 10.1136/bmjopen-2021-052393 (PMC8883228; doi:10.1136/bmjopen-2021-052393)
Supplement: Author's manuscript [file bmjopen-2021-052393.draft_revisions.pdf]

BMJ Open

BMJ Open is committed to open peer review. As part of this commitment we make the peer review history of every article we publish publicly available.

When an article is published we post the peer reviewers' comments and the authors' responses online. We also post the versions of the paper that were used during peer review. These are the versions that the peer review comments apply to.

The versions of the paper that follow are the versions that were submitted during the peer review process. They are not the versions of record or the final published versions. They should not be cited or distributed as the published version of this manuscript.

BMJ Open is an open access journal and the full, final, typeset and author-corrected version of record of the manuscript is available on our site with no access controls, subscription charges or pay-per-view fees (http://bmjopen.bmj.com).

If you have any questions on BMJ Open's open peer review process please email info.bmjopen@bmj.com

BMJ Open

Global Prevalence and Trends in Hypertension and Type 2 Diabetes Mellitus among Slum Residents: A Systematic Review and Meta-analysis

Journal:	*BMJ Open*
Manuscript ID	bmjopen-2021-052393
Article Type:	Original research
Date Submitted by the Author:	15-Apr-2021
Complete List of Authors:	Uthman, Olalekan; University of Warwick, Warwick Centre for Global Health, Warwick Medical School<br>Ayorinde, Abimbola; University of Warwick, Warwick Centre for Global Health, Warwick Medical School<br>Oyebode, Oyinlola; University of Warwick Warwick Medical School, Warwick Centre for Global Health, Warwick Medical School<br>Sartori, Jo; University of Birmingham, Institute of Applied Health Research<br>Gill, Paramjit ; University of Warwick, Warwick Centre for Global Health, Warwick Medical School,<br>Lilford, RJ; University of Birmingham, Institute of Applied Health Research
Keywords:	Hypertension < CARDIOLOGY, DIABETES & ENDOCRINOLOGY, Public health < INFECTIOUS DISEASES

SCHOLARONE™
Manuscripts

I, the Submitting Author has the right to grant and does grant on behalf of all authors of the Work (as defined in the below author licence), an exclusive licence and/or a non-exclusive licence for contributions from authors who are: i) UK Crown employees; ii) where BMJ has agreed a CC-BY licence shall apply, and/or iii) in accordance with the terms applicable for US Federal Government officers or employees acting as part of their official duties; on a worldwide, perpetual, irrevocable, royalty-free basis to BMJ Publishing Group Ltd ("BMJ") its licensees and where the relevant Journal is co-owned by BMJ to the co-owners of the Journal, to publish the Work in this journal and any other BMJ products and to exploit all rights, as set out in our licence.

The Submitting Author accepts and understands that any supply made under these terms is made by BMJ to the Submitting Author unless you are acting as an employee on behalf of your employer or a postgraduate student of an affiliated institution which is paying any applicable article publishing charge ("APC") for Open Access articles. Where the Submitting Author wishes to make the Work available on an Open Access basis (and intends to pay the relevant APC), the terms of reuse of such Open Access shall be governed by a Creative Commons licence – details of these licences and which Creative Commons licence will apply to this Work are set out in our licence referred to above.

Other than as permitted in any relevant BMJ Author's Self Archiving Policies, I confirm this Work has not been accepted for publication elsewhere, is not being considered for publication elsewhere and does not duplicate material already published. I confirm all authors consent to publication of this Work and authorise the granting of this licence.

For peer review only

Global Prevalence and Trends in Hypertension and Type 2 Diabetes Mellitus among Slum Residents: A Systematic Review and Meta-analysis

For peer review only

Olalekan A. Uthman[1*], Ayorinde A. Abimbola[1], Oyinlola Oyebode[1], Jo Sartori[2], Paramjit Gill[1,3], RJ. Lilford[2]

[1]Warwick Centre for Global Health, Warwick Medical School, University of Warwick, Coventry, UK

[2]Institute of Applied Health Research, University of Birmingham, Birmingham, UK

[3]Academic Unit of Primary Care, Warwick Medical School, University of Warwick, Coventry, UK

***Correspondence author:**

Professor Olalekan A. Uthman, Warwick Centre for Global Health, Division of Health Sciences, Warwick Medical School, The University of Warwick, Coventry, CV4 7AL, United Kingdom. Email: olalekan.uthman@warwick.ac.uk

ABSTRACT

Objective: To obtain regional estimates of prevalence of hypertension and Type 2 diabetes in urban slums, and secondly to compare these with those in urban and rural areas.

Design: Systematic review and meta-analysis

Data sources: Ovid MEDLINE, Cochrane CENTRAL and EMBASE from inception to December 2020

Eligibility criteria: Studies that reported hypertension prevalence using the definition of blood pressure ≥140/90 mm Hg and/or prevalence of type 2 diabetes.

Data extraction and synthesis: Two authors extracted relevant data and assessed risk of bias independently. We used random-effects meta-analyses to pool prevalence estimates.

Results: A total 61 studies involving 105,559 participants met the inclusion criteria. Prevalence of hypertension and type 2 diabetes in slum populations ranged from 4.2% to 52.5% and 0.9% to 25.0%, respectively. The pooled prevalence of hypertension tended to be higher among studies from South Asia (25.3%, 95% CI 21.3 to 29.6, 26 studies) and sub-Saharan Africa (24.4%, 95% CI 17.7 to 31.9, 10 studies) than those from Latin America and Caribbean (18.3%, 95% CI 13.4 to 23.9, 6 studies). However, the pooled prevalence of type 2 tended to be higher among studies from South Asia (11.6%, 95% CI 9.1 to 21.9, 18 studies) than those from sub-Saharan Africa (4.5%, 95% CI 2.4 to 7.2, 8 studies). In six studies presenting comparator data, all from the Indian sub-continent, slum residents were 35% more likely to be hypertensive than those living in comparator rural areas and 30% less likely to be hypertensive than those from comparator non-slum urban areas. Four studies from India (n=3) and Bangladesh reported prevalence of type 2 diabetes by place of residence and the pooled prevalence of type 2 diabetes was highest among those residing in non-slum urban areas, followed by urban slum residents and was lowest among rural residents.

Conclusion: The burden of hypertension and type 2 diabetes varied widely between countries and regions and, to some degree, also within countries. In addition, many hypertensive individuals are not aware of their condition, not on treatment and control of hypertension is poor. The burden of hypertension and type 2 diabetes was higher among non-slum urban residents than their counterparts living in urban slums and rural areas.

PROSPERO registration number: CRD42017077381

For peer review only - http://bmjopen.bmj.com/site/about/guidelines.xhtml

Strengths and limitations of this study

- The prevalence of hypertension and type 2 diabetes differed greatly across countries and regions, and to some extent within countries.
- Many hypertensive people are unaware of their disease, are not on medication, and their hypertension control is weak.
- The prevalence of hypertension and type 2 diabetes was higher in non-slum urban residents than in urban slums and rural areas.
- This meta-analysis pooled prevalence estimates from various regions and reported over an 11-year period, and as predicted, high heterogeneity between studies was found in the meta-analyses.

For peer review only - http://bmjopen.bmj.com/site/about/guidelines.xhtml

INTRODUCTION

Noncommunicable diseases (NCDs) are currently the leading cause of death globally, even in low- and middle-income countries (LMICs) the burden of disease is shifting from infectious diseases to NCDs[1]. NCDs now account for about 41 million deaths annually, corresponding to nearly 7 in 10 of all deaths worldwide. Every year, 15 million people of ages of 30 to 69 years die from these diseases, more than 85% of which are people living in LMICs. Most of the deaths from NCDs are caused by cardiovascular diseases, followed by cancer and respiratory diseases. NCDs affect people in all age groups, countries and geographic regions. The leading causes of these diseases include increased consumption of unhealthy foods, increased physical inactivity and population ageing[2-4]. These factors are mediated through metabolic risk factors for NCDs the most common of which include hypertension and type 2 diabetes[2-4]

Urbanization is a global phenomenon that is occurring at a fast pace in most LMICs[5 6]. For more than 20 years, urban settlements have been increasing in population size because of fast growth in urban births, significant movement of people from rural areas and sustained integration of the global economy [5 6]. The United Nations defines slums as urban areas with overcrowding, poor sanitation infrastructure, limited access to safe water, and/or poor structural quality of housing[7 8]. Slums are now an important component of today's urban settlements and likely continue to be for the foreseeable future [7 8].

For peer review only - http://bmjopen.bmj.com/site/about/guidelines.xhtml

Despite increased global awareness about the presence and persistence of slums, and evidence that their popualtions are affected by different health problems and needs to other urban inhabitants, the health of their inhabitants is under researched[7-10]. The health of the urban poor, people with low socioeconomic status living in urban areas, is usually conflated with that of slum residents. Although there is substantial overlap between these groups, there are also richer residents within slum neighbourhoods, as well as urban poverty occuring in non-slum urban areas. Health outcomes for these two groups may differ depending on whether deprivation is at the individual (urban poverty) or neighbourhood level (slum resident) due to neighbourhood effects [7 8 11 12]. For exampe, with respect to NCD risk-factors, those resident in slums, whatever their personal socio-economic status, may be more exposed to a common physical environmental risk factors (for example: air pollution increasing risk of hypertension), social environmental risk factors (for example: crime rates which may increase stress and drive metabolic risk) or institutional risk-factors (for example: stigma on the basis of their address reducing access to appropriate medical care). Many existing studies of NCDs risk factors done in urban areas do not disaggregate the population's health data by slum and non-slums status to allow for the detection of intra-urban health disparities that are due to neighbourhood effects rather than individual socio-economic status[13-22].

Understanding how the global challenges of hypertension, type 2 diabetes and rapid unplanned urbanisation intersect, by investigating whether the up to 1 billion people residing in slums[23]are succumbing to these important metabolic risk factors for non-communicable disease, will inform priorities for health services and health policy in LMICs. To fill this research gap, we therefore systematically gathered all the publications that relate to the burden of

For peer review only - http://bmjopen.bmj.com/site/about/guidelines.xhtml

105 hypertension among slum residents to (1) assess the contemporary prevalence esimates of
106 hypertension among slum residents (2) compare the prevalence of hypertension and Type 2
107 diabetes in slums with those in two other types of settlement i.e. non-slum urban and rural
108 areas; and (3) assess the proportion of those with hypertension who were aware of their
109 hypertensive status, those on treatment and those with blood pressure under control.

110

111

For peer review only

For peer review only - http://bmjopen.bmj.com/site/about/guidelines.xhtml

METHODS

Protocol and registration

The study background, rationale, and methods were specified in advance and documented in a protocol that was published in the PROSPERO register (CRD42017077381).

Search and information sources:

We searched Ovid MEDLINE, Cochrane CENTRAL and EMBASE from inception to December 2020 using the following keywords: slum, shanty town, ghetto, hypertension and type 2 diabetes. The search strategy for Medline is shown in **Annex 1**.

Eligibility criteria:

We evaluated each identified study against the following pre-defined selection criteria:

- *Types of studies:* We included all studies (cross-sectional studies, retrospective or prospective cohort studies) that reported prevalence of hypertension among slum residents as a primary or secondary outcome. No language, publication date or publication status restrictions were imposed.
- *Types of participants:* adult population (18 years and above) living in slum (as defined by the authors of the original studies included).
- *Types of Interventions:* Not applicable.
- *Types of outcomes:* Essential hypertension (also called primary or idiopathic hypertension), defined as persistent (seated) systolic blood pressure (SBP) of 140 mmHg or greater or had diastolic blood pressure 90mmHg or greater regardless of age and sex. We excluded studies that included subjects with pregnancy-induced, pre-

For peer review only - http://bmjopen.bmj.com/site/about/guidelines.xhtml

eclampsia, malignant, portal, pulmonary, renal, intracranial or ocular hypertension. We also excluded studies used only self-reported measure, i.e. deducible from the use of antihypertensive drugs or self-reported physician-diagnosed cases. If data were available, we noted (1) the percentage of those aware of their hypertension status (2) on any anti-hypertensive treatment, and (3) blood pressure controlled to a target level. Awareness of hypertension was defined as self-reporting of any prior diagnosis of hypertension by a healthcare professional. Treatment of hypertension was defined as receiving prescribed antihypertensive medication for management of high BP at some time in the 1 year preceding the survey. Control of hypertension was defined as the proportion of patients reporting antihypertensive therapy with SBP of less than 140 mmHg and DBP of less than 90 mmHg.

Type 2 diabetes was defined based on measured fasting plasma glucose, or oral glucose tolerance test. Type 2 diabetes was diagnosed if the fasting blood glucose was ≥126 mg/dL (≥7.0 mmol/L) after an overnight fast for at least 8 hours, or random capillary blood glucose of >= 11.1 mmol/L or if the participant was taking treatment for type 2 diabetes.

Study selection

In pairs, three reviewers (OAU, AAA, OO) independently evaluated the eligibility and methodological quality of the studies obtained from the literature searches. All articles yielded by the database search were initially screened by their titles and abstracts to obtain studies that met inclusion criteria. In cases of discrepancies, agreement was reached by discussion with a third reviewer.

For peer review only - http://bmjopen.bmj.com/site/about/guidelines.xhtml

Data collection process and data items

OAU extracted data and AAA and OO checked the extracted data. For each study that met the selection criteria, details extracted included on year of publication, country of origin, study design, sample size, sampling strategy, study period, setting (rural/urban/slum), socio-demographic variables, prevalence estimates; etc.

Risk of bias (quality) assessment

We used the Risk of Bias Assessment tool for Non-randomized Studies (RoBANS)[24] to assessed the risk of bias of included studies (see Box 1). The risk of bias in a study was graded as low, high or unclear on the basis of study features including the selection of participants (selection bias), participation rate (selection bias), outcome measurement (detection bias), consideration of confounding variables (analytical methods to control for bias), and other form of bias.

For each included study, we estimated the precision (C) or margin of error, considering the sample size (SS) and the observed prevalence (p) of hypertension among slum dwellers from the formula:

$$SS = Z^2 * p * (1-p) / C^2 \qquad (1)$$

where Z was the z-value fixed at 1.96 across studies (corresponding to 95% confidence interval). The desirable margin of error is 5% (0.05) or lower.

Box 1: Risk of bias assessment

Bias type	Low-risk of bias	High-risk of bias	Unclear risk of bias
Selection (sample population)	participants selected randomly	Sample selection ambiguous and sample unlikely to be representative	Insufficient information
Selection (participation rate)	High participation rate (>70-85%)	Low participation rate (<70%)	Insufficient information

For peer review only - http://bmjopen.bmj.com/site/about/guidelines.xhtml

Performance bias (outcome assessment)	Objective measures of hypertension	Self-reported measure of hypertension	Insufficient information
Performance bias (analytical methods to control for bias)	Analysis appropriate for type of sample (unadjusted, univariable analyses etc.)	Analysis does not account for common adjustment (adjusted, multivariable analyses)	Insufficient information
Other form of bias	There is no evidence of bias from other sources.	There is potential bias present from other sources	Insufficient information

Synthesis of results

For the meta-analysis, we used DerSimonian-Laird random effects model[25] due to anticipated variations in study population, health care delivery systems and stage of epidemic transition to pool the hypertension and type 2 diabetes prevalence estimates. We performed leave-one-study-out sensitivity analysis to determine the stability of the results[26]. This analysis evaluated the influence of individual studies by estimating the pooled prevalence estimates in the absence of each study[26]. We assessed heterogeneity among studies by inspecting the forest plots and using the chi-squared test for heterogeneity with a 10% level of statistical significance and using the I^2 statistic where we interpret a value of 50% as representing moderate heterogeneity[27 28]. We assessed the possibility of publication bias by evaluating a funnel plot for asymmetry. Because graphical evaluation can be subjective, we also conducted a Egger's regression asymmetry test as formal statistical tests for publication bias[29].

Following the overall analyses, we performed the following sub-group analyses: place of residence (rural versus urban slum versus non-slum urban); participants risk factors, including socioeconomic position; study design (cross-sectional, cohort); study location (low- and middle income versus high-income countries); and study precision.

For peer review only - http://bmjopen.bmj.com/site/about/guidelines.xhtml

For peer review only

We examined time trends in the hypertension prevalence estimates using meta-regression regression models with the prevalence estimates as the outcome variable and the calendar year of the publication as the predictor. In order to measure secular patterns in prevalence figures, we use the annual average percentages change (AAPC). We fitted a regression line to the natural logarithm of the prevalence estimates, i.e., $y = \alpha + \beta x + \varepsilon$, where $y = \ln(\text{Prevalence})$, and x = calendar year. The AAPC was calculated as $100 \times (\exp(\beta)-1)$. The 95% confidence interval (CI) of the AAPC was also computed from the regression model. [30] The prevalence calculations indicated an upward trend when both the AAPC estimate and the lower limit of its 95% CI were > 0. However, they indicated a downward trend when both the AAPC and its upper limits were less than 0. The prevalence estimates were otherwise considered stable over time[30]. This systematic review was reported according to the Preferred Reporting Items for Systematic Reviews and Meta-analyses (PRISMA) guideline (**Annex 2**)[31].

Patient and public involvement

The design of this review meant it was not appropriate or possible to involve patients or the public in the design, or conduct, or reporting, or dissemination plans of our research.

Results

Study selection and characteristics

The literature search yielded 1490 articles. **eFigure 1** shows the study selection flow diagram. After review, 134 articles were selected for critical reading. Seventy-three studies did not meet the inclusion criteria and were excluded (see **eTable 1** for list of excluded studies). The other 61 studies involving 105,559 participants met the inclusion criteria and were included in the meta-analysis[13-22 32-80]. Forty-three studies reported only hypertension prevalence

For peer review only - http://bmjopen.bmj.com/site/about/guidelines.xhtml

estimates, 29 studies reported only type 2 diabetes prevalence estimates and seven reported both. **Table 1 and eTable 2** presents the characteristics of the included studies. The studies were reported between 1989 and 2019. Studies were reported as full-text journal articles (n=50, **98%);** except for one which was reported as a conference abstract. The number of participants included in the studies ranged from 100 to 15,763. When reported, the mean age of participants ranged from 32 years to 47 years. Most of the studies were carried out in South Asia: India (n=30); Bangladesh (n=7) and Nepal (n=1) and Pakistan (n=1); followed by sub-Saharan Africa: Kenya (n=9) and Nigeria (n=4); Latin America and Caribbean: Brazil (n=5) and Peru (n=1) and East Asia and Pacific: Thailand (n=1). Most of the studies were conducted in the following urban slums: Kibera (n=4), Delhi (n=3), Hyderabad (n=3), Ajegunle (n=2), Chandigarh (n=2), Chennai (n=2), Dhaka (n=2), Haryana (n=2), and Maceio (n=2).

Risk of bias of included studies

Summary of risk of bias assessment for each study is shown in **eTable 3.** The risk of bias in the selection of participants was low in most studies (n=57, 93%), high in three studies (5%) and unclear in one study. The risk of selection bias due to participate rate was low in most studies (n=55, 90%), unclear in four (7%) and high in two study (3%). The performance bias due to outcome assessment was low in all the 61 studies as we included all studies that used objective measure of hypertension and type 2 diabetes. The performance bias due to analytical methods was low in 39 studies (64%) and high in 22 studies (36%). The risk of other biases was low in most studies (n=44, 72%), unclear in 16 studies (26%) and high in one study (2%).

Variations in prevalence of hypertension and type 2 diabetes by geographical regions

For peer review only - http://bmjopen.bmj.com/site/about/guidelines.xhtml

Prevalence of hypertension and type 2 diabetes from individuals are shown in **Figure 1 and Figure 2** respectively.

East Asia and Pacific

Thailand: One study from Klong-Toey slum found that 77 of the 976 respondents had type 2 diabetes in 1989 (7.9%, 95% CI 6.3 to 9.8).

Latin America and Caribbean

Brazil: Four studies reported the prevalence of hypertension from three different slums: Maceio (n=2), Rio de Janeiro (n=1) and Salvador (n=1). Florencio et al. found that almost one third of the Maceio slum dweller were hypertensive in 2004 (29.8%, 95% CI 24.8 to 35.2), while Ferriera et al estimated prevalence of hypertenssion among Maceio slum residents to be 14.8% (95% 10.4 to 20.2) in 2005. The reported prevalence of hypertension in other slums was 11.3% (95% CI10.2 to 12.4) in Rio de Janerio in 2007 and 20.6% (95% CI 19.5 to 21.7) in Salvador in 2015. The pooled prevalence ('annualised year average') of hypertension for the four studies yielded an estimate of 18.4% (95% CI 12.0% to 26.2%). One study from Brazil found that one in ten had type 2 diabetes in 2017.

Peru: One study from a Lima slum conducted in 2014 found that 21 of the 142 respondents were hypertensive (14.8%, 95% CI 9.4 to 21.7).

South Asia

Bangladesh: Three studies from Dhakan slum reported prevalence of hypertension. The reported prevalence of hyertension ranged from 11.6% (95% CI 9.7 to 13.8) in 2012 to 19.56%

For peer review only - http://bmjopen.bmj.com/site/about/guidelines.xhtml

(95% CI 17.85 to 21.37) in 2018. Four studies from Dhakan slum reported prevalence of type 2 diabetes. The pooled prevalence ('annualised year average') of hypertension for the three studies yielded an estimate of 14.9% (95% CI 9.9% to 20.6%). The reported prevalence of type 2 diabetes in these slums ranged from 8.1% (95% CI 6.8 to 9.6) in 2004 to 18.12% (95% CI 16.46 to 19.87) in 2019.

India: Twenty-two studies from India reported prevalence of hypertension from more than 15 difference slums. The reported prevalence varied across and within the slums. For example, Kar and colleagues estimated the prevalence of hypertension of 27.6% (95% 21.4 to 34.4) among 196 Chandigarh and Haryana slum residents in 2008; however they estimated the prevalence of hypertension of 16.5% (95% CI 15.1 to 18.0) among 2,562 196 Chandigarh and Haryana slum residents in 2010. Prevalence of type diabetes also varied across slums in India. The pooled prevalence ('annualised year average') of hypertension for the 22 studies yielded an estimate of 26.8% (95% CI 22.5% to 31.3%). In Delhi, the reported prevalence of type 2 diabetes ranged from 12.7% (95% CI 11.3 to 14.2) in 2007 to 31.5% (95% CI 27.8 to 35.4) in 2012. The pooled prevalence ('annualised year average') of type 2 for the 13studies yielded an estimate of 12.2% (95% CI 9.2% to 15.6%).

Nepal: One study from a Kathmandu slum conducted in 2013 found that 193 of the 689 respondents were hypertensive (28.0%, 95% CI 24.7 to 31.5).

Pakistan: One study from a Lahore slum found that 22 of the 695 respondents had type 2 diabetes in 2008 (3.2%, 95% CI 2.0 to 4.8).

For peer review only - http://bmjopen.bmj.com/site/about/guidelines.xhtml

Sub-Saharan Africa. *Kenya:* Six studies reported the prevalence of hypertension from three different slums: Kibera (n=4) and Viwandani and Korogocho (n=2). The reported prevalence among Kibera slum residents ranged from 13.0% (95% CI9.9 to 16.7) in 2013 to 27.8% (95% CI 25.9 to 29.7) in 2015. van de Vijver found that 640 of the 5,190 respondents from Viwandani and Korogocho slum residents were hypertensive (12.3%, 95% CI 11.5 to 13.3). The pooled prevalence ('annualised year average') of hypertension for the six studies yielded an estimate of 19.2% (95% CI 13.2% to 26.0%). The reported prevalence of type 2 diabetes ranged from 0.9% (95% CI 0.7 to 1.2 in Nairobi slum in 2016 to 4.4% (95% CI 3.8 to 5.0) in Viwandani and Korogocho in 2013. The pooled prevalence ('annualised year average') of type 2 diabetes for the six studies yielded an estimate of 4.5% (95% CI 2.0% to 7.9%).

Nigeria: Four studies from five different slums reported prevalence of hypertension. The reported prevalence varied across and within the slums. Ezeala-Adikaibe found that half of the respondents from Enugu slum were hypertensive in 2016 (52.5%, 95% CI 48.9 to 56.0). While Daniel et al. and Sowemimo et al. found that almost one-third of the Ajegule (38.2%, 95% CI 35.1 to 41.3, 2013) and Yemetu (33.1%, 95% CI 30.0 to 36.5, 2015) slum residents were hypertensive. However, Akinwale found that only 12.8% of the respondents from Ijora Oloye, Ajegunle and Makoko were hypertensive in 2013. The pooled prevalence ('annualised year average') of hypertension for the four studies yielded an estimate of 33.2% (95% CI 15.6% to 53.5%). Akinwale found that only 3.3% of the respondents from Ijora Oloye, Ajegunle and Makoko had type 2 diabetes in 2013.

Secular trends in hypertension and Type 2 diabetes prevalence estimates

For peer review only - http://bmjopen.bmj.com/site/about/guidelines.xhtml

Secular trends in hypertension, in 5 countries for which there were data across multiple time points, and type 2 diabetes, in 3 countries in which we had data across multiple time points, among slum residents are shown in **Figures 3 and 4**. We observed a continuous increase in prevalence of hypertension among slum residents in four out of five countries. The increase is more pronounced in India, followed by Kenya and Bangladesh. The prevalence of hypertension increased by 204.6% from 11.7% in 2001 to 35.5% in 2019 in India. The prevalence of hypertension increased by 98.8% from 12.3% in 2013 to 24.5% in 2019 in Kenya. However, the results of the trend analysis showed statistically significant upward trends only in India, such that the prevalence of hypertension increased +6.9% (95% CI +2.0% to +12.0%) per year between 2001 and 2019. There was no statistically significant trend was observed in Brazil using trend analyses (trend =-0.0%, 95% CI −22.7% to +29.2%). We also observed a continuous increase in prevalence of type 2 diabetes among slum residents in India and Bangladesh. The prevalence of type 2 diabetes increased by 123.6% from 8.1% in 2004 to 18.1% in 2019 in Bangladesh. The prevalence of type 2 diabetes increased by 95.8% from 10.3% in 2001 to 20.2% in 2019 in India. However, the results of the trend analysis showed statistically significant upward trends only in Bangladesh such that the prevalence of type 2 diabetes increased +5.9% (95% CI +1.1% to +10.8%) per year between 2004 and 2019. A non-statistically significant downward trends in type 2 diabetes prevalence was also observed in Kenya (trend =-11.1%, 95% CI −45.7% to +45.6%).

Prevalence of hypertension by different hypertension and type 2 diabetes subgroups

Study characteristics: As shown in **Table 1**, the pooled prevalence of hypertension was highest in studies conducted in lower-middle income countries (23.2%, 95% CI 21.5 to 29.0,

For peer review only - http://bmjopen.bmj.com/site/about/guidelines.xhtml

36 studies) than those from upper-middle income countries (17.9%, 95% CI 12.1 to 24.6, 5 studies). The pooled prevalence of hypertension tended to be higher among studies from South Asia (25.3%, 95% CI 21.3 to 29.6, 26 studies) and sub-Saharan Africa (24.4%, 95% CI 17.7 to 31.9, 10 studies) than those from Latin America and Carribean (18.3%, 95% CI 13.4 to 23.9, 6 studies). The pooled prevalence tended to higher among imprecise studies (33.4%, 95% CI 25.7 to 41.7, 8 studies) than those from precise studies (22.4%, 95% CI 18.9 to 26.1%, 35 studies). The pattern was similar for type 2 diabetes prevalence estimates.

Socio-demographic characteristics: As shown in **Table 1**, the pooled prevalence of hypertension was similar among males (22.5%, 95% CI 16.0 to 29.7, 24 studies) and females (23.5%, 95% CI 18.6 to 28.1, 24 studies). The pooled prevalence of hypertension tended to be higher among older adults (49.6%, 95% CI 36.7 to 62.6, 9 studies) than middle-age (35.0%, 95% CI 45.6, 9 studies) and young adults (15.7%, 95% CI 10.1 to 22.1, 8 studies). Similarly, the pooled prevalence of hypertension tended to be higher obese (45.4%, 95% CI 34.5 to 56.5, 6 studies) and overweight (32.9%, 95% CI 21.2 to 45.8, 6 studies) participants than participants with normal (21.9%, 95% CI 11.8 to 34.2, 6 studies) and under-weight (21.8%, 95% CI 11.4 to 34.4, 5 studies). The pooled prevalence of hypertension tended to be higher among those never studied (39.1%, 95% CI 27.5 to 51.3) than those with less than primary (18.3%, 95% CI 13.9 to 23.1, 4 studies), primary (24.8%, 95% CI 12.0 to 40.4, 6 studies) or secondary/higher education attainment (22.4%, 95% CI 11.2 to 36.2, 7 studies). The pooled prevalence of hypertension tended to be higher among least poor (29.2%, 95% CI 13.1 to 48.5, 5 studies) than those with middle- (25.3%, 10.6 to 43.8, 5 studies) and poorest-income (20.9%, 95% CI 10.4 to 33.8, 5 studies). The pattern was similar for type 2 diabetes prevalence estimates.

For peer review only - http://bmjopen.bmj.com/site/about/guidelines.xhtml

Lifestyle factors: The pooled prevalence of hypertension tended to be higher among smokers (38.0%, 95% CI 19.1 to 59.0, 5 studies) than those not smoking (30.5%, 95% CI 17.6 to 45.2, 5 studies). We found that the pooled prevalence of hypertension tended to be higher those not physically active (30.8%, 95% CI 7.7 to 60.9, 3 studies) than those physical active (28.8%, 95% CI 11.1 to 50.8); tended to be higher among with no history of alcohol consumption (29.1%, 95% CI 9.3 to 54.3, 3 studies) than those reported alcohol consumption (26.5%, 95% CI 18.0 to 35.9, 3 studies).

Comparative prevalence by place of residence

Six studies from India included non-slum populations alongside data from the slum population, and reported prevalence of hypertension by place of residence[36 38 46 48 49 51]. As shown in **Figure 5**, the pooled prevalence of hypertension was highest among those residing in non-slum urban areas (33.5%, 95% CI 26.0 to 42.0, 6 studies), followed by urban slum residents (28.8%, 95% CI 23.7 to 34.4%, 6 studies) and was lowest among rural residents (24.4%, 95% 18.4 to 31.5, 5 studies). Slum residents were 35% more likely to be hypertensive than those living in rural areas (OR = 1.35, 95% 1.29 to 1.42) and 30% less likely to be hypertensive than those living in other urban areas (OR = 0.70, 95% CI 0.51 to 0.96).

Four studies from India (n=3) and Bangladesh reported prevalence of Type 2 diabetes by place of residence[46 51 59 71]. As shown in **Figure 6**, the pooled prevalence of type 2 diabetes was highest among those residing in non-slum urban areas (13.06%, 95% CI 6.53 to 24.43, 4 studies; 2813 participants), followed by urban slum residents (7.88%, 95% CI 3.32 to 17.55; 4 studies; 1811 participants) and was lowest among rural residents (1.64%; 95% CI 0.06 to

For peer review only - http://bmjopen.bmj.com/site/about/guidelines.xhtml

32.21; 3 studies; 405 participants). Such that prevalence of type 2 diabetes tended to be higher among urban slum residents than those living in rural areas (OR = 3.78, 95% 0.75 to 18.93). Urban slum residents were 46% less likely to be diabetic than those from other urban areas (OR = 0.54, 95% CI 0.44 to 0.66).

Treatment cascade

Among those diagnosed with hypertension, only one-third were aware of their hypertensive status (33.6%, 95% CI 19.1 to 50.0%, 12 studies) (**Table 1)**. Among those aware of their high blood pressure, half of them were on antihypertensive medications (51.9%, 95% CI 35.2 to 68.3, 9 studies). Among those on treatment, only one-quarter had good blood pressure control (25.2, 95% CI 18.4 to 34.3, 8 studies). Among those diagnosed with type 2 diabetes, 57.4% were aware of their type 2 diabetes status (95% CI 18.2 to 91.8%, 2 studies).

Discussion

Main Findings

This systematic review and meta-analysis summarises available evidence on the global prevalence of hypertension and type 2 diabetes among slum residents. There were several key findings: firstly, the burden of hypertension and type 2 diabetes among slum dweller is high and may be rising globally, with wide variation between countries and regions and, to some degree, also within countries. Using data from within study comparator populations when presented, the pooled prevalence of hypertension and Type 2 diabetes was highest among those residing in non-slum urban areas, followed by slum residents and was lowest

For peer review only - http://bmjopen.bmj.com/site/about/guidelines.xhtml

For peer review only

among rural residents. This finding corroborates those of previous reviews that observed higher prevalence of hypertension among urban residents than those living in rural areas[81 82]. This high prevalence may be due to rapid urbanization, lifestyle changes, dietary changes and increased life expectancy[83 84] or a combination of these factors[85 86]. In addition, the observed difference could be due to other factors including but not limited to lack of access to testing and care of NCDs risk factors in rural areas and urban areas.

The observed gradient in burden of hypertension and Type 2 diabetes among rural, slum and urban residents is consistent with the effects of urbanization and wealth, as residents experience an economic transition when moving from one area to the next[87-92]. LMICs are now undergoing epidemiological transition, the change from a burden of infectious diseases to chronic diseases [93]. In addition, it could be due to increase in awareness in (non-slum) urban areas and recent availability of testing in some places. Recent systematic reviews of dietary risk-behaviour in Sub-Saharan Africa have found that urban populations tended to consume more salt than rural populations [94] and consume fewer portions of vegetables[12]. The rapid pace of urbanisation and economic growth is accelerating the rate of this epidemiologic transition; as such LMICs are at great risk for an explosive growth in the burden of NCDs, including hypertension and type 2 diabetes [87 88].

We found evidence of significant unmet need for hypertension care among urban slum residents. Significant proportion of the urban slum residents were unscreened, undiagnosed, untreated or uncontrolled. This huge unmet need has been documented in previous studies from low- and middle-income settings[95-101]. We also found that control of hypertension among slum residents was poor, such that only one in four slum residents on treatment, had

For peer review only - http://bmjopen.bmj.com/site/about/guidelines.xhtml

their blood pressure controlled. The poor control of BP noted in our study, despite the fact the one half of those that were unaware of high blood pressure being on antihypertensive medications, needs further exploration. One possible explanation is availability and affordability of the medications and there could be minimal additional contact with a health professional[15]. It has been documented that the control of BP was related to the frequency of follow-up visits[96]. Another possible explanation could be low adherence to prescribed medications, as they may not be able to afford the medications.

As expected, we found that the burden of hypertension increased with the particpants' age, which may be attributed to age-related structural changes in blood vessels which potentially cause narrowing of the vascular lumen, and consequently increasing blood pressure, as have been reported in previous studies[102 103]. The association between combined overweight/obesity and hypertension shown in our results exemplify the role of excess body weight in hypertension prevalence, which has been long recognized and consistent across numerous observational and trial data[104-106]. We found evidence of significantly high prevalence of hypertension among smokers compared to the non-smokers. Direct relation of chronic tobacco consumption with hypertension however is not yet well established[107 108] although tobacco consumption has been shown to cause an acute elevation of BP[109].

Study Limitations and Strengths

To the best of our knowledge, this paper is the first systematic reviews that summarises data about prevalence of hypertension and type 2 diabetes among slum residents. Strengths of this study include the use of a predefined and published protocol, a comprehensive search strategy, and involvement of two independent reviewers in the review process. Nevertheless,

For peer review only

For peer review only - http://bmjopen.bmj.com/site/about/guidelines.xhtml

the findings of this study should be interpreted with caution. Prevalence estimates from different regions and published over the course of 11 years were pooled in this meta-analysis, and as expected, high heterogeneity between studies was found in the meta-analyses. Nonetheless, as affirmed by previous evidence, meta-analyses are the preferred options to narrative syntheses for interpreting the results in a review, even in spite of the presence of a considerable amount of heterogeneity[110]. Heterogeneity appeared to be the norm rather than exception in published meta-analyses of observational studies[111].

In conclusion, the burden of hypertension and type 2 diabetes varied widely between countries and regions and, to some degree, also within countries. In addition, many hypertensive individuals are not aware of their condition, not on treatment and control of hypertension is poor. The burden of hypertension and type 2 diabetes was higher among urban residents than their counterparts living in urban slums and rural areas. There is a need for public health strategies to improve the awareness, control and overall management of hypertension and type 2 diabetes in urban areas.

For peer review only

For peer review only - http://bmjopen.bmj.com/site/about/guidelines.xhtml

Funding

This research was funded by the National Institute for Health Research (NIHR) Global Health Research Unit on Improving Health in Slums using UK aid from the UK Government to support global health research (Award ID: 16/136/87). The views expressed in this publication are those of the author(s) and not necessarily those of the NIHR or the UK Department of Health and Social Care.

Ethics approval and consent to participate

Not applicable.

Consent for publication

Not applicable.

Data sharing statement:

No additional data available

Competing interests

The authors declare that they have no competing interests.

Authors' contribution

OAU, AAA, OO and RL conceived the study. OAU, AAA and OO collected and analysed initial data. OAU, AAA, OO, JO, PG and RL participated contributed in refining the data analysis. OAU wrote the first manuscript. OAU, AAA, OO, JS, PG and RL contributed to further

For peer review only

For peer review only - http://bmjopen.bmj.com/site/about/guidelines.xhtml

analysis, interpreting and shaping of the argument of the manuscript and participated in writing the final draft of the manuscript. All the authors read and approved the final manuscript.

References

1. Global, regional, and national comparative risk assessment of 84 behavioural, environmental and occupational, and metabolic risks or clusters of risks, 1990-2016: a systematic analysis for the Global Burden of Disease Study 2016. *Lancet (London, England)* 2017;390(10100):1345-422. doi: 10.1016/s0140-6736(17)32366-8 [published Online First: 2017/09/19]

2. Bickler SW, Wang A, Amin S, et al. Urbanization in Sub-Saharan Africa: Declining Rates of Chronic and Recurrent Infection and Their Possible Role in the Origins of Non-communicable Diseases. *World journal of surgery* 2018;42(6):1617-28. doi: 10.1007/s00268-017-4389-5 [published Online First: 2017/12/14]

3. Goryakin Y, Rocco L, Suhrcke M. The contribution of urbanization to non-communicable diseases: Evidence from 173 countries from 1980 to 2008. *Economics and human biology* 2017;26:151-63. doi: 10.1016/j.ehb.2017.03.004 [published Online First: 2017/04/15]

4. Khorrami Z, Etemad K, Yarahmadi S, et al. Urbanization and noncommunicable disease (NCD) risk factors: WHO STEPwise Iranian NCD risk factors surveillance in 2011. *Eastern Mediterranean health journal = La revue de sante de la Mediterranee orientale = al-Majallah al-sihhiyah li-sharq al-mutawassit* 2017;23(7):469-79. [published Online First: 2017/08/31]

5. Cohen B. Urban Growth in Developing Countries: A Review of Current Trends and a Caution Regarding Existing Forecasts. *World Development* 2004;32(1):23-51. doi: https://doi.org/10.1016/j.worlddev.2003.04.008

6. Cohen B. Urbanization in developing countries: Current trends, future projections, and key challenges for sustainability. *Technology in Society* 2006;28(1):63-80. doi: https://doi.org/10.1016/j.techsoc.2005.10.005

7. Ezeh A, Oyebode O, Satterthwaite D, et al. The history, geography, and sociology of slums and the health problems of people who live in slums. *Lancet (London, England)* 2017;389(10068):547-58. doi: 10.1016/s0140-6736(16)31650-6 [published Online First: 2016/10/21]

8. Lilford RJ, Oyebode O, Satterthwaite D, et al. Improving the health and welfare of people who live in slums. *Lancet (London, England)* 2017;389(10068):559-70. doi: 10.1016/s0140-6736(16)31848-7 [published Online First: 2016/10/21]

For peer review only - http://bmjopen.bmj.com/site/about/guidelines.xhtml

9. Riley LW, Ko AI, Unger A, et al. Slum health: diseases of neglected populations. *BMC Int Health Hum Rights* 2007;7:2. doi: 10.1186/1472-698x-7-2 [published Online First: 2007/03/09]

10. Unger A, Riley LW. Slum health: from understanding to action. *PLoS medicine* 2007;4(10):1561-6. doi: 10.1371/journal.pmed.0040295 [published Online First: 2007/10/26]

11. Lilford R, Kyobutungi C, Ndugwa R, et al. Because space matters: conceptual framework to help distinguish slum from non-slum urban areas. *BMJ Glob Health* 2019;4(2):e001267. doi: 10.1136/bmjgh-2018-001267 [published Online First: 2019/05/30]

12. Mensah DO, Nunes AR, Bockarie T, et al. Meat, fruit, and vegetable consumption in sub-Saharan Africa: a systematic review and meta-regression analysis. *Nutr Rev* 2020 doi: 10.1093/nutrit/nuaa032 [published Online First: 2020/06/20]

13. Ahmad S, Goel K, Parashar P, et al. A community based cross sectional study on life style & morbidity status of elderly in urban slums of meerut. *Indian Journal of Public Health Research and Development* 2014;5(1):153-57.

14. Anand K, Shah B, Yadav K, et al. Are the urban poor vulnerable to non-communicable diseases? A survey of risk factors for non-communicable diseases in urban slums of Faridabad. *National Medical Journal of India* 2007;20(3):115-20.

15. Banerjee S, Mukherjee TK, Basu S. Prevalence, awareness, and control of hypertension in the slums of Kolkata. *Indian Heart Journal* 2016;68(3):286-94. doi: http://dx.doi.org/10.1016/j.ihj.2015.09.029

16. Daniel OJ, Adejumo OA, Adejumo EN, et al. Prevalence of hypertension among urban slum dwellers in Lagos, Nigeria. *Journal of urban health : bulletin of the New York Academy of Medicine* 2013;90(6):1016-25. doi: 10.1007/s11524-013-9795-x [published Online First: 2013/02/27]

17. Heitzinger K, Montano SM, Hawes SE, et al. A community-based cluster randomized survey of noncommunicable disease and risk factors in a peri-urban shantytown in Lima, Peru. *BMC International Health and Human Rights* 2014;14(1) doi: http://dx.doi.org/10.1186/1472-698X-14-19

18. Nirmala DB, Vijay KM, Sreedhar M. Prevalence of risk factors for Non Communicable Diseases in urban slums of Hyderabad, Telangana *Indian Journal of Basic and Applied Medical Research* 2014;4(1):487-93.

19. Oli N, Vaidya A, Thapa G. Behavioural risk factors of noncommunicable diseases among nepalese urban poor: A descriptive study from a slum area of Kathmandu. *Epidemiology Research International* 2013(pagination) doi: http://dx.doi.org/10.1155/2013/329156

20. Rawal LB, Biswas T, Khandker NN, et al. Non-communicable disease (NCD) risk factors and diabetes among adults living in slum areas of Dhaka, Bangladesh. *PLoS ONE* 2017;12(10) doi: http://dx.doi.org/10.1371/journal.pone.0184967

21. Singh R, Mukherjee M, Kumar R, et al. Study of Risk factors of Coronary Heart Disease in Urban Slums of Patna. *2012* 2012;2(3):-192. doi: 10.3126/nje.v2i3.6902 [published Online First: 2012-10-02]

For peer review only - http://bmjopen.bmj.com/site/about/guidelines.xhtml

22. Vigneswari A, Manikandan R, Satyavani K, et al. Prevalence of Risk Factors of Diabetes Among Urban Poor South Indian Population. *The Journal of the Association of Physicians of India* 2015;63(10):32-4. [published Online First: 2016/09/10]

23. UN-Habitat. UN-Habitat Urbanization and development: emerging futures. world cities report 2016. Nairobi Kenya, 2016.

24. Kim SY, Park JE, Lee YJ, et al. Testing a tool for assessing the risk of bias for nonrandomized studies showed moderate reliability and promising validity. *Journal of clinical epidemiology* 2013;66(4):408-14. doi: 10.1016/j.jclinepi.2012.09.016 [published Online First: 2013/01/23]

25. DerSimonian R, Laird N. Meta-analysis in clinical trials. *Controlled clinical trials* 1986;7(3):177-88. [published Online First: 1986/09/01]

26. Normand SL. Meta-analysis: formulating, evaluating, combining, and reporting. *Statistics in medicine* 1999;18(3):321-59. [published Online First: 1999/03/10]

27. Higgins JP, Thompson SG. Quantifying heterogeneity in a meta-analysis. *Statistics in medicine* 2002;21(11):1539-58. doi: 10.1002/sim.1186 [published Online First: 2002/07/12]

28. Higgins JP, Thompson SG, Deeks JJ, et al. Measuring inconsistency in meta-analyses. *BMJ (Clinical research ed)* 2003;327(7414):557-60. doi: 10.1136/bmj.327.7414.557 [published Online First: 2003/09/06]

29. Egger M, Davey Smith G, Schneider M, et al. Bias in meta-analysis detected by a simple, graphical test. *BMJ (Clinical research ed)* 1997;315(7109):629-34. [published Online First: 1997/10/06]

30. Clegg LX, Hankey BF, Tiwari R, et al. Estimating average annual per cent change in trend analysis. *Statistics in medicine* 2009;28(29):3670-82. doi: 10.1002/sim.3733 [published Online First: 2009/10/27]

31. Liberati A, Altman DG, Tetzlaff J, et al. The PRISMA statement for reporting systematic reviews and meta-analyses of studies that evaluate healthcare interventions: explanation and elaboration. *BMJ (Clinical research ed)* 2009;339:b2700. doi: 10.1136/bmj.b2700 [published Online First: 2009/07/23]

32. Acharyya T, Kaur P, Murhekar MV. Prevalence of behavioral risk factors, overweight and hypertension in the urban slums of North 24 Parganas District, West Bengal, India, 2010. *Indian journal of public health* 2014;58(3):195-98.

33. Akinwale O, Oyefara J, Adejoh P, et al. The benefits of using a community-engaged research approach to promote a healthy lifestyle in three Nigerian urban slums. *Southern African Journal of Epidemiology and Infection* 2014;29(1):48-50.

34. Ayah R, Joshi MD, Wanjiru R, et al. A population-based survey of prevalence of diabetes and correlates in an urban slum community in Nairobi, Kenya. *BMC public health* 2013;13(371):20. doi: https://dx.doi.org/10.1186/1471-2458-13-371

For peer review only

For peer review only - http://bmjopen.bmj.com/site/about/guidelines.xhtml

35. Chakraborty R, Bose K. Comparison of body adiposity indices in predicting blood pressure and hypertension among slum-dwelling men in Kolkata, India. *Malaysian Journal of Nutrition* 2012;18(3):319-28.

36. Chaturvedi S, Pant M, Yadav G. Hypertension in Delhi: prevalence, awareness, treatment and control. *Tropical doctor* 2007;37(3):142-5. doi: 10.1258/004947507781524593 [published Online First: 2007/08/25]

37. Dasappa H, Fathima FN, Prabhakar R, et al. Prevalence of diabetes and pre-diabetes and assessments of their risk factors in urban slums of Bangalore. *Journal of family medicine and primary care* 2015;4(3):399-404. doi: 10.4103/2249-4863.161336 [published Online First: 2015/08/20]

38. Deepa M, Pradeepa R, Anjana R, et al. Noncommunicable diseases risk factor surveillance: experience and challenge from India. *Indian journal of community medicine : official publication of Indian Association of Preventive & Social Medicine* 2011;36(Suppl 1):S50-6. doi: 10.4103/0970-0218.94709 [published Online First: 2012/05/26]

39. Edwards JK, Bygrave H, Van den Bergh R, et al. HIV with non-communicable diseases in primary care in Kibera, Nairobi, Kenya: characteristics and outcomes 2010-2013. *Trans R Soc Trop Med Hyg* 2015;109(7):440-6. doi: https://dx.doi.org/10.1093/trstmh/trv038

40. Ezeala-Adikaibe BA, Orjioke C, Ekenze OS, et al. Population-based prevalence of high blood pressure among adults in an urban slum in Enugu, South East Nigeria. *Journal of Human Hypertension* 2016;30(4):285-91. doi: http://dx.doi.org/10.1038/jhh.2015.49

41. Ferreira HDS, Florencio TMTDM, Fragoso MDAC, et al. Hypertension, abdominal obesity and short stature: Aspects of nutritional transition within a shantytown in the city of Maceio (Northeastern Brazil). *Revista de Nutricao* 2005;18(2):209-18.

42. Florencio TT, Ferreira HS, Cavalcante JC, et al. Short stature, obesity and arterial hypertension in a very low income population in North-eastern Brazil. *Nutrition, Metabolism and Cardiovascular Diseases* 2004;14(1):26-33. doi: http://dx.doi.org/10.1016/S0939-4753%2804%2980044-9

43. Haregu TN, Oti S, Ngomi N, et al. Interlinkage among cardio-metabolic disease markers in an urban poor setting in Nairobi, Kenya. *Global health action* 2016;9(pp 30626) doi: http://dx.doi.org/10.3402/gha.v9.30626

44. Huda MN, Alam KS, Harun Ur R. Prevalence of chronic kidney disease and its association with risk factors in disadvantageous population. *International journal of nephrology* 2012;2012:267329. doi: 10.1155/2012/267329 [published Online First: 2012/08/01]

45. Jalil F, Moore SE, Butt NS, et al. Early-life risk factors for adult chronic disease: Follow-up of a cohort born during 1964-1978 in an urban slum of Lahore, Pakistan. *Journal of Health, Population and Nutrition* 2008;26(1):12-21.

46. Joshi A, Puricelli Perin DM, Arora M. Using Portable Health Information Kiosk to assess chronic disease burden in remote settings. *Rural and remote health* 2013;13(2):2279. [published Online First: 2013/03/29]

For peer review only - http://bmjopen.bmj.com/site/about/guidelines.xhtml

47. Joshi MD, Ayah R, Njau EK, et al. Prevalence of hypertension and associated cardiovascular risk factors in an urban slum in Nairobi, Kenya: a population-based survey. *BMC public health* 2014;14:1177. doi: 10.1186/1471-2458-14-1177 [published Online First: 2014/11/20]

48. Kar SS, Thakur JS, Jain S, et al. Cardiovascular disease risk management in a primary health care setting of North India. *Indian Heart Journal* 2008;60(1):19-25.

49. Kar SS, Thakur JS, Virdi NK, et al. Risk factors for cardiovascular diseases: Is the social gradient reversing in northern India? *National Medical Journal of India* 2010;23(4):206-09.

50. Kumari SMV, Humaira B, Sreedhar M. A study on prevalence of hypertension in urban slum field practice area of osmania medical college – Hyderabad *Indian Journal of Basic and Applied Medical Research* 2014;4(1):462-70.

51. Lubree HG, Rege SS, Bhat DS, et al. Body fat and cardiovascular risk factors in Indian men in three geographical locations. *Food and Nutrition Bulletin* 2002;23(3 SUPP):146-49.

52. Marins VM, Almeida RM, Pereira RA, et al. The association between socioeconomic indicators and cardiovascular disease risk factors in Rio de Janeiro, Brazil. *J Biosoc Sci* 2007;39(2):221-9. doi: https://dx.doi.org/10.1017/S0021932006001246

53. Misra A, Pandey RM, Devi JR, et al. High prevalence of diabetes, obesity and dyslipidaemia in urban slum population in northern India. *International journal of obesity and related metabolic disorders : journal of the International Association for the Study of Obesity* 2001;25(11):1722-9. doi: 10.1038/sj.ijo.0801748 [published Online First: 2001/12/26]

54. Olack B, Wabwire-Mangen F, Smeeth L, et al. Risk factors of hypertension among adults aged 35-64 years living in an urban slum Nairobi, Kenya. *BMC public health* 2015;15:1251. doi: 10.1186/s12889-015-2610-8 [published Online First: 2015/12/19]

55. Ongeti K, Ogeng'o J, Pulei A, et al. Blood pressure characteristics among slum dwellers in Kenya. *Global Advanced Research* 2013;2(4):80-85.

56. Oti SO, van de Vijver SJ, Agyemang C, et al. The magnitude of diabetes and its association with obesity in the slums of Nairobi, Kenya: results from a cross-sectional survey. *Tropical medicine & international health : TM & IH* 2013;18(12):1520-30. doi: 10.1111/tmi.12200 [published Online First: 2013/10/15]

57. Patil RS, Gothankar JS. Assessment of risk of type 2 diabetes using the Indian Diabetes Risk Score in an urban slum of Pune, Maharashtra, India: a cross-sectional study. *WHO South-East Asia journal of public health* 2016;5(1):53-61. doi: 10.4103/2224-3151.206555 [published Online First: 2016/04/01]

58. Rahim MA, Vaaler S, Keramat Ali SM, et al. Prevalence of type 2 diabetes in urban slums of Dhaka, Bangladesh. *Bangladesh Medical Research Council Bulletin* 2004;30(2):60-70.

59. Sayeed MA, Mahtab H, Khanam PA, et al. Prevalence of diabetes and impaired fasting glucose in urban population of Bangladesh. *Bangladesh Medical Research Council Bulletin* 2007;33(1):1-12.

For peer review only - http://bmjopen.bmj.com/site/about/guidelines.xhtml

60. Singh AK, Mani K, Krishnan A, et al. Prevalence, awareness, treatment and control of diabetes among elderly persons in an urban slum of delhi. *Indian journal of community medicine : official publication of Indian Association of Preventive & Social Medicine* 2012;37(4):236-9. doi: 10.4103/0970-0218.103472 [published Online First: 2013/01/08]

61. Sinha P, Taneja DK, Singh NP, et al. Seasonal variation in prevalence of hypertension: Implications for interpretation. *Indian journal of public health* 2010;54(1):7-10.

62. Sitthi-Amorn C, Chandraprasert S, Bunnag SC, et al. The prevalence and risk factors of hypertension in Klong Toey Slum and Klong Toey government apartment houses. *International Journal of Epidemiology* 1989;18(1):89-94.

63. Snyder RE, Lopes LA, Tavares LCC, et al. O Dia de Dona Maria-Using technology and community based participatory research to improve healthcare delivery in a Brazilian urban slum. *Annals of Global Health* 2016;Conference:7th Annual CUGH Conference: Bridging to a Sustainable Future in Global Health. United States. 82 (3) (pp 599).

64. Sowemimo I, Ajayi I, Akpa O, et al. Prevalence of hypertension and associated factors among residents of Ibadan-north local government area of Oyo State, Nigeria. *Journal of Hypertension* 2015;Conference:25th European Meeting on Hypertension and Cardiovascular Protection. doi: http://dx.doi.org/10.1097/01.hjh.0000467432.10548.8c

65. Sunita M, Singh AK, Rogye A, et al. Prevalence of Diabetic Retinopathy in Urban Slums: The Aditya Jyot Diabetic Retinopathy in Urban Mumbai Slums Study-Report 2. *Ophthalmic Epidemiology* 2017;24(5):303-10. doi: http://dx.doi.org/10.1080/09286586.2017.1290258

66. Unger A, Felzemburgh RD, Snyder RE, et al. Hypertension in a Brazilian urban slum population. *J Urban Health* 2015;92(3):446-59. doi: https://dx.doi.org/10.1007/s11524-015-9956-1

67. Uthakalla VK, Kishore Kumar KJ, Jena SK, et al. Prevalence study of overweight/obesity among adults (20-60yrs) of urban field practice area of osmania medical college, Hyderabad. *Indian Journal of Public Health Research and Development* 2012;3(3):250-53.

68. van de Vijver S, Oti S, Tervaert TC, et al. Introducing a model of cardiovascular prevention in Nairobi's slums by integrating a public health and private-sector approach: the SCALE-UP study. *Global health action* 2013;6(pp 22510)

69. Vikram NK, Pandey RM, Misra A, et al. Non-obese (body mass index < 25 kg/m²) Asian Indians with normal waist circumference have high cardiovascular risk. *Nutrition* 2003;19(6):503-09. doi: http://dx.doi.org/10.1016/S0899-9007%2802%2901083-3

70. Wasir JS, Misra A, Vikram NK, et al. C-reactive protein, obesity, and insulin resistance in postmenopausal women in urban slums of North India. *Diabetes and Metabolic Syndrome: Clinical Research and Reviews* 2007;1(2):83-89. doi: http://dx.doi.org/10.1016/j.dsx.2007.02.001

71. Yajnik CS, Joglekar CV, Lubree HG, et al. Adiposity, inflammation and hyperglycaemia in rural and urban Indian men: Coronary Risk of Insulin Sensitivity in Indian Subjects (CRISIS) Study. *Diabetologia* 2008;51(1):39-46. doi: https://dx.doi.org/10.1007/s00125-007-0847-1

For peer review only - http://bmjopen.bmj.com/site/about/guidelines.xhtml

72. Hypertension detection, treatment and control rates in urban slum population in bangladesh. *Journal of Hypertension* 2018;Conference:27th Scientific Meeting of the International Society of Hypertension, ISH 2018. China. 36 (Supplement 3) (pp e337-e338).

73. Assessment of risk factors of hypertension among adults residing in urban slum of Delhi. *Asian Journal of Pharmaceutical and Clinical Research* 2018;11(1):405-07. doi: http://dx.doi.org/10.22159/ajpcr.2018.v11i1.23755

74. Prevalence of hypertension among elderly residing in slums of west Delhi. *Asian Journal of Pharmaceutical and Clinical Research* 2018;11(4):337-39. doi: http://dx.doi.org/10.22159/ajpcr.2018.v11i4.23414

75. Abhinav Jain BKAMPSMSKSACSJ. A Study of Prevalence of Diabetes Mellitus and its Risk Factors in the Urban Slum Population of Gurugram. *Indian Journal of Public Health Research & Development* 2019;10(4):141-45. doi: 10.37506/ijphrd.v10i4.6613

76. Bawah AT, Abaka-Yawson A, Seini MM, et al. Prevalence of diabetes among homeless and slum dwellers in Accra, Ghana: a survey study. *BMC Res Notes* 2019;12(1):572. doi: 10.1186/s13104-019-4613-5 [published Online First: 2019/09/13]

77. Gadallah M, Megid SA, Mohsen A, et al. Hypertension and associated cardiovascular risk factors among urban slum dwellers in Egypt: a population-based survey. *Eastern Mediterranean health journal = La revue de sante de la Mediterranee orientale = al-Majallah al-sihhiyah li-sharq al-mutawassit* 2018;24(5):435-42. doi: 10.26719/2018.24.5.435 [published Online First: 2018/07/26]

78. George CE, Norman G, Wadugodapitya A, et al. Health issues in a Bangalore slum: findings from a household survey using a mobile screening toolkit in Devarajeevanahalli. *BMC public health* 2019;19(1):456. doi: 10.1186/s12889-019-6756-7 [published Online First: 2019/05/01]

79. Tymejczyk O, McNairy ML, Petion JS, et al. Hypertension prevalence and risk factors among residents of four slum communities: population-representative findings from Port-au-Prince, Haiti. *Journal of hypertension* 2019;37(4):685-95. doi: 10.1097/hjh.0000000000001966 [published Online First: 2019/03/01]

80. Vusirikala A, Wekesah F, Kyobutungi C, et al. Assessment of cardiovascular risk in a slum population in Kenya: use of World Health Organisation/International Society of Hypertension (WHO/ISH) risk prediction charts - secondary analyses of a household survey. *BMJ open* 2019;9(9):e029304. doi: 10.1136/bmjopen-2019-029304 [published Online First: 2019/09/07]

81. Addo J, Smeeth L, Leon DA. Hypertension in sub-saharan Africa: a systematic review. *Hypertension (Dallas, Tex : 1979)* 2007;50(6):1012-8. doi: 10.1161/hypertensionaha.107.093336 [published Online First: 2007/10/24]

82. Pereira M, Lunet N, Azevedo A, et al. Differences in prevalence, awareness, treatment and control of hypertension between developing and developed countries. *Journal of hypertension* 2009;27(5):963-75. [published Online First: 2009/04/30]

For peer review only

For peer review only - http://bmjopen.bmj.com/site/about/guidelines.xhtml

83. Gupta R, al-Odat NA, Gupta VP. Hypertension epidemiology in India: meta-analysis of 50 year prevalence rates and blood pressure trends. *J Hum Hypertens* 1996;10(7):465-72. [published Online First: 1996/07/01]

84. Mahmood SE, Prakash D, Srivastava JP, et al. Prevalence of Hypertension Amongst Adult Patients Attending Out Patient Department of Urban Health Training Centre, Department of Community Medicine, Era's Lucknow Medical College and Hospital, Lucknow. *Journal of clinical and diagnostic research : JCDR* 2013;7(4):652-6. doi: 10.7860/jcdr/2013/4707.2874 [published Online First: 2013/06/05]

85. Amuna P, Zotor FB. Epidemiological and nutrition transition in developing countries: impact on human health and development. *The Proceedings of the Nutrition Society* 2008;67(1):82-90. doi: 10.1017/s0029665108006058 [published Online First: 2008/02/01]

86. Kroll M, Bharucha E, Kraas F. Does rapid urbanization aggravate health disparities? Reflections on the epidemiological transition in Pune, India. *Glob Health Action* 2014;7:23447. doi: 10.3402/gha.v7.23447 [published Online First: 2014/09/13]

87. Angkurawaranon C, Jiraporncharoen W, Chenthanakij B, et al. Urbanization and non-communicable disease in Southeast Asia: a review of current evidence. *Public health* 2014;128(10):886-95. doi: 10.1016/j.puhe.2014.08.003 [published Online First: 2014/11/05]

88. Cheema A, Adeloye D, Sidhu S, et al. Urbanization and prevalence of type 2 diabetes in Southern Asia: A systematic analysis. *Journal of global health* 2014;4(1):010404. doi: 10.7189/jogh.04.010404 [published Online First: 2014/07/01]

89. Low WY, Lee YK, Samy AL. Non-communicable diseases in the Asia-Pacific region: Prevalence, risk factors and community-based prevention. *International journal of occupational medicine and environmental health* 2015;28(1):20-6. doi: 10.2478/s13382-014-0326-0 [published Online First: 2015/07/15]

90. Phipps ME, Chan KK, Naidu R, et al. Cardio-metabolic health risks in indigenous populations of Southeast Asia and the influence of urbanization. *BMC public health* 2015;15:47. doi: 10.1186/s12889-015-1384-3 [published Online First: 2015/02/01]

91. Siegel KR, Patel SA, Ali MK. Non-communicable diseases in South Asia: contemporary perspectives. *British medical bulletin* 2014;111(1):31-44. doi: 10.1093/bmb/ldu018 [published Online First: 2014/09/06]

92. Streatfield PK, Khan WA, Bhuiya A, et al. Adult non-communicable disease mortality in Africa and Asia: evidence from INDEPTH Health and Demographic Surveillance System sites. *Glob Health Action* 2014;7:25365. doi: 10.3402/gha.v7.25365 [published Online First: 2014/11/08]

93. Gaziano TA, Bitton A, Anand S, et al. Growing epidemic of coronary heart disease in low- and middle-income countries. *Current problems in cardiology* 2010;35(2):72-115. doi: 10.1016/j.cpcardiol.2009.10.002 [published Online First: 2010/01/30]

94. Oyebode O, Oti S, Chen YF, et al. Salt intakes in sub-Saharan Africa: a systematic review and meta-regression. *Population health metrics* 2016;14:1. doi: 10.1186/s12963-015-0068-7 [published Online First: 2016/01/14]

For peer review only - http://bmjopen.bmj.com/site/about/guidelines.xhtml

95. Adeloye D, Basquill C. Estimating the prevalence and awareness rates of hypertension in Africa: a systematic analysis. *PLoS One* 2014;9(8):e104300. doi: 10.1371/journal.pone.0104300 [published Online First: 2014/08/05]

96. Macia E, Duboz P, Gueye L. Prevalence, awareness, treatment and control of hypertension among adults 50 years and older in Dakar, Senegal. *Cardiovascular journal of Africa* 2012;23(5):265-9. doi: 10.5830/cvja-2011-039 [published Online First: 2011/10/18]

97. Mohan V, Deepa M, Farooq S, et al. Prevalence, awareness and control of hypertension in Chennai--The Chennai Urban Rural Epidemiology Study (CURES-52). *The Journal of the Association of Physicians of India* 2007;55:326-32. [published Online First: 2007/09/12]

98. Pilav A, Doder V, Brankovic S. Awareness, Treatment, and control of Hypertension among Adult Population in the Federation of Bosnia and Herzegovina over the Past Decade. *Journal of public health research* 2014;3(3):323. doi: 10.4081/jphr.2014.323 [published Online First: 2015/01/02]

99. Supiyev A, Kossumov A, Utepova L, et al. Prevalence, awareness, treatment and control of arterial hypertension in Astana, Kazakhstan. A cross-sectional study. *Public health* 2015;129(7):948-53. doi: 10.1016/j.puhe.2015.02.020 [published Online First: 2015/03/31]

100. Tailakh A, Evangelista LS, Mentes JC, et al. Hypertension prevalence, awareness, and control in Arab countries: a systematic review. *Nursing & health sciences* 2014;16(1):126-30. doi: 10.1111/nhs.12060 [published Online First: 2013/10/15]

101. Yazdanpanah L, Shahbazian H, Shahbazian H, et al. Prevalence, awareness and risk factors of hypertension in southwest of Iran. *Journal of renal injury prevention* 2015;4(2):51-6. doi: 10.12861/jrip.2015.11 [published Online First: 2015/06/11]

102. Landahl S, Bengtsson C, Sigurdsson JA, et al. Age-related changes in blood pressure. *Hypertension (Dallas, Tex : 1979)* 1986;8(11):1044-9. [published Online First: 1986/11/01]

103. Pinto E. Blood pressure and ageing. *Postgraduate medical journal* 2007;83(976):109-14. doi: 10.1136/pgmj.2006.048371 [published Online First: 2007/02/20]

104. Dyer AR, Elliott P, Shipley M. Body mass index versus height and weight in relation to blood pressure. Findings for the 10,079 persons in the INTERSALT Study. *American journal of epidemiology* 1990;131(4):589-96. [published Online First: 1990/04/01]

105. Folsom AR, Kushi LH, Anderson KE, et al. Associations of general and abdominal obesity with multiple health outcomes in older women: the Iowa Women's Health Study. *Archives of internal medicine* 2000;160(14):2117-28. [published Online First: 2000/07/25]

106. Hu G, Barengo NC, Tuomilehto J, et al. Relationship of physical activity and body mass index to the risk of hypertension: a prospective study in Finland. *Hypertension (Dallas, Tex : 1979)* 2004;43(1):25-30. doi: 10.1161/01.Hyp.0000107400.72456.19 [published Online First: 2003/12/06]

107. Abtahi F, Kianpour Z, Zibaeenezhad MJ. Correlation between cigarette smoking and blood pressure and pulse pressure among teachers residing in Shiraz, Southern Iran. *Iran Cardiovasc Res J* 2011;5:97-102.

For peer review only

For peer review only - http://bmjopen.bmj.com/site/about/guidelines.xhtml

108. Primatesta P, Falaschetti E, Gupta S, et al. Association between smoking and blood pressure: evidence from the health survey for England. *Hypertension (Dallas, Tex : 1979)* 2001;37(2):187-93. [published Online First: 2001/03/07]

109. Westman EC. Does smokeless tobacco cause hypertension? *Southern medical journal* 1995;88(7):716-20. [published Online First: 1995/07/01]

110. Ioannidis JP, Patsopoulos NA, Rothstein HR. Reasons or excuses for avoiding meta-analysis in forest plots. *BMJ (Clinical research ed)* 2008;336(7658):1413-5. doi: 10.1136/bmj.a117 [published Online First: 2008/06/21]

111. Higgins JP. Commentary: Heterogeneity in meta-analysis should be expected and appropriately quantified. *Int J Epidemiol* 2008;37(5):1158-60. doi: 10.1093/ije/dyn204 [published Online First: 2008/10/04]

For peer review only

For peer review only - http://bmjopen.bmj.com/site/about/guidelines.xhtml

TABLES

Table 1: Pooled prevalence by difference subgroup

Subgroup		Hypertension				Type 2 Diabetes		
		n	%	I^2		n	%	I^2
Sample size	Smaller studies (<1000)	27	25.9 (21.6 to 30.6)	97.1		15	11.0 (8.2 to 14.2)	93.9
Sample size	Larger studies (1000+)	16	21.6 (16.6 to 27.0)	99.6		14	7.2 (4.6 to 10.3)	99.3
Study precision	Imprecise studies	8	33.4 (25.7 to 41.7)	91.2		1	25.2 (17.3 to 34.2)	
Study precision	Precise studies	35	22.4 (18.9 to 26.1)	99.3		28	8.6 (6.6 to 11.0)	98.8
Publication year	2001 to 2005	5	15.6 (9.0 to 23.8)	94.7		4	8.2 (6.7 to 9.8)	53.6
Publication year	2006 to 2010	6	28.6 (18.9 to 39.4)	98.7		4	6.3 (3.3 to 10.3)	90.6
Publication year	2011 to 2020	32	24.9 (21.2 to 28.8)	99.2		21	9.9 (7.2 to 12.9)	99.1
Region	South Asia	26	25.3 (21.3 to 29.6)	98.9		18	11.6 (9.1 to 14.3)	97.3
Region	Sub-Saharan Africa	10	24.4 (17.7 to 31.9)	99.2		8	4.5 (2.4 to 7.2)	98.8
Region	Latin America and Caribbean	6	18.3 (13.4 to 23.9)	97.1		1	10.2 (8.1 to 12.3)	
Region	Middle East and North Africa	1	31.2 (28.4 to 34.1)			1	8.8 (7.1 to 10.6)	
						1	7.9 (6.3 to 9.7)	
Income category	Lower Middle Income	36	25.2 (21.5 to 29.0)	99.1		27	9.0 (6.8 to 11.5)	98.8
Income category	Upper Middle Income	5	17.9 (12.1 to 24.6)	97.6		2	9.0 (6.9 to 11.3)	62
Income category	Low Income	2	24.0 (16.9 to 32.0)	92.2				
Sex	Male	24	22.5 (16.0 to 29.7)	99.2		11	8.1 (5.1 to 11.6)	97.6
Sex	Female	24	23.2 (18.6 to 28.1)	98.7		11	7.3 (4.6 to 10.6)	97.5
Age	Young adult	8	15.7 (10.1 to 22.1)	97.8		2	2.1 (0.3 to 5.4)	96.7
Age	Middle-age adult	9	35.0 (25.0 to 45.6)	99.2		2	5.6 (4.5 to 6.8)	0
Age	Older adult	9	49.6 (36.7 to 62.6)	98.3		2	9.1 (7.0 to 11.4)	0
Body mass index	Under weight	5	21.8 (11.4 to 34.4)	87.3				
Body mass index	Normal weight	6	21.9 (11.8 to 34.2)	98.6		2	2.3 (1.8 to 2.8)	0
Body mass index	Overweight	6	32.9 (21.2 to 45.8)	97.4		2	4.2 (1.2 to 8.8)	50
Body mass index	Obese	6	45.4 (34.5 to 56.6)	93.3		2	6.4 (4.0 to 9.3)	0
Education Status	Never studied	7	39.1 (27.5 to 51.3)	98		1	5.1 (3.0 to 7.8)	
Education Status	Less than primary	4	18.3 (13.9 to 23.1)	87.1		1	4.6 (3.4 to 6.1)	
Education Status	Primary	6	24.8 (12.0 to 40.4)	99.4		1	4.4 (3.6 to 5.2)	
Education Status	Secondary or higher	7	22.4 (11.1 to 36.2)	99.3		1	4.1 (3.2 to 5.2)	
Income	Poorest	5	20.9 (10.4 to 33.8)	98.9				
Income	Middle	5	25.3 (10.6 to 43.8)	99.5				
Income	Least poor	5	29.2 (13.1 to 48.5)	98.3				
Smoking status	Yes	5	38.0 (19.1 to 59.0)	99.1				
Smoking status	No	5	30.5 (17.6 to 45.2)	99.6				
Alcohol consumption	Yes	3	26.5 (18.0 to 35.9)	83.4				
Alcohol consumption	No	3	29.1 (9.3 to 54.3)	99.7				
Physically active	Yes	3	28.8 (11.1 to 50.8)	99.6				
Physically active	No	3	30.8 (7.7 to 60.9)	98.4				
Treatment cascade	Aware of HBP	12	33.6 (19.1 to 50.0)	99.7				
Treatment cascade	On treatment	9	51.9 (35.2 to 68.3)	98.6				
Treatment cascade	BP controlled	8	25.9 (18.4 to 34.3)	87.8				

* World Bank Country Income Groups, 2018

For peer review only

For peer review only - http://bmjopen.bmj.com/site/about/guidelines.xhtml

Participants were divided into age groups that, broadly defined, covered young adulthood (18 to 35 years), middle age (36 to 55 years), and older adulthood (56 years and older).

Underweight - BMI under 18.5 kg/m^2

Normal weight - BMI greater than or equal to 18.5 to 24.9 kg/m^2

Overweight – BMI greater than or equal to 25 to 29.9 kg/m^2

Obesity – BMI greater than or equal to 30 kg/m^2

Physical activity as defined by the authors

Alcohol consumption as defined by authors

Smoking status as defined by authors

Income status as reported by authors

FIGURE LEGENDS

Figure 1: Hypertension prevalence estimates among slum residents and 95% confidence intervals from individual studies and pooled data

Figure 2: Type 2 diabetes mellitus prevalence estimates among slum residents and 95% confidence intervals from individual studies and pooled data

Figure 3: Secular trends in hypertension prevalence estimates among slum residents across different regions

Figure 4: Secular trends in Type 2 diabetes mellitus prevalence estimates among slum residents across different regions

For peer review only - http://bmjopen.bmj.com/site/about/guidelines.xhtml

For peer review only

Figure 5: Hypertension prevalence estimates by place of residence: urban versus rural versus slum

Figure 6: Type 2 diabetes mellitus prevalence estimates by place of residence: urban versus rural versus slum

For peer review only

For peer review only - http://bmjopen.bmj.com/site/about/guidelines.xhtml

ONLINE ONLY SUPPLEMENTS

eFigure 1: Study selection and inclusion flow chart

eTable 1: List of Excluded Studies

eTable 2: Characteristics of included studies

eTable 3: Risk of bias of included studies

Annex 1: MEDLINE Search Strategy

Annex 2: PRISMA Checklist

For peer review only

For peer review only - http://bmjopen.bmj.com/site/about/guidelines.xhtml

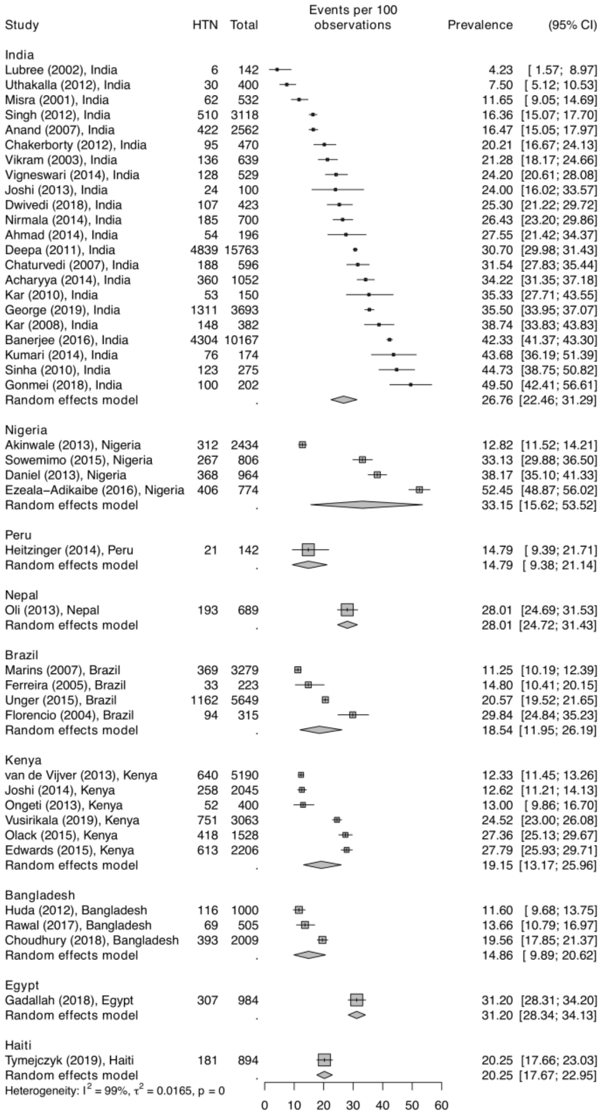

Figure 1

228x406mm (300 x 300 DPI)

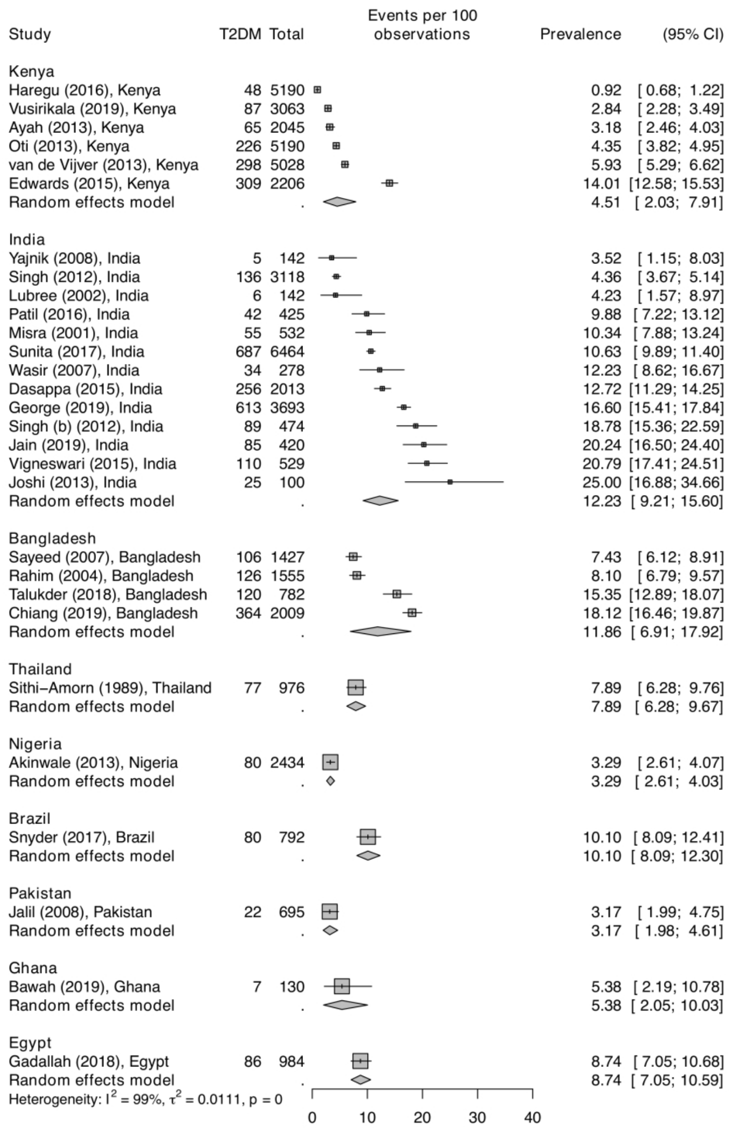

Figure 2

228x326mm (300 x 300 DPI)

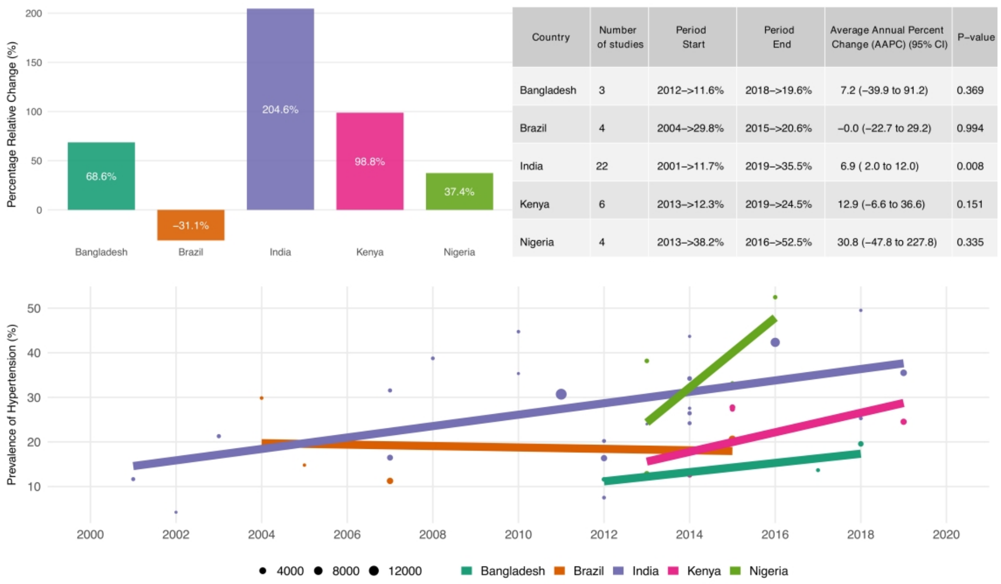

Country	Number of studies	Period Start	Period End	Average Annual Percent Change (AAPC) (95% CI)	P−value
Bangladesh	3	2012−>11.6%	2018−>19.6%	7.2 (−39.9 to 91.2)	0.369
Brazil	4	2004−>29.8%	2015−>20.6%	−0.0 (−22.7 to 29.2)	0.994
India	22	2001−>11.7%	2019−>35.5%	6.9 (2.0 to 12.0)	0.008
Kenya	6	2013−>12.3%	2019−>24.5%	12.9 (−6.6 to 36.6)	0.151
Nigeria	4	2013−>38.2%	2016−>52.5%	30.8 (−47.8 to 227.8)	0.335

Figure 3

462x274mm (300 x 300 DPI)

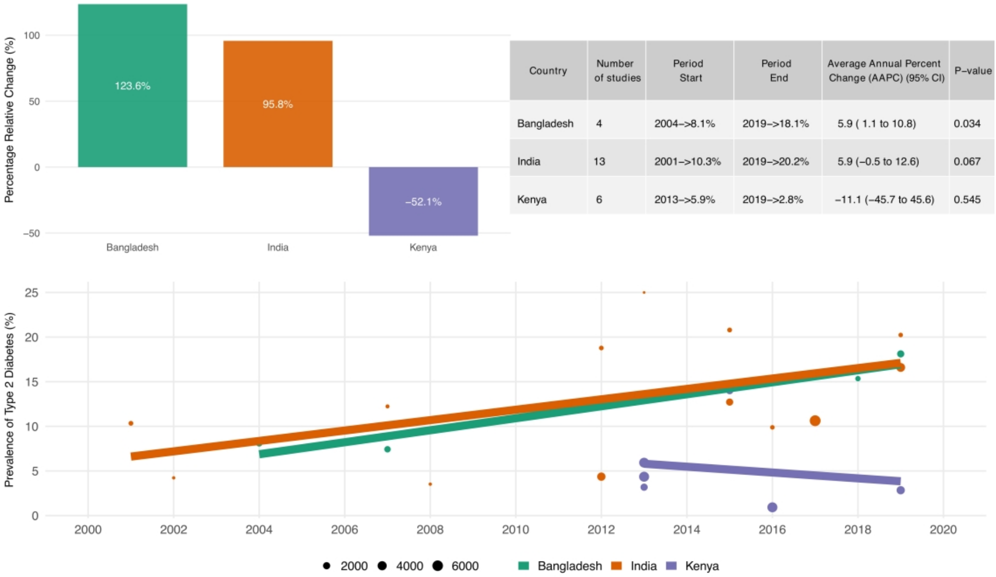

Country	Number of studies	Period Start	Period End	Average Annual Percent Change (AAPC) (95% CI)	P–value
Bangladesh	4	2004–>8.1%	2019–>18.1%	5.9 (1.1 to 10.8)	0.034
India	13	2001–>10.3%	2019–>20.2%	5.9 (−0.5 to 12.6)	0.067
Kenya	6	2013–>5.9%	2019–>2.8%	−11.1 (−45.7 to 45.6)	0.545

Figure 4

462x274mm (300 x 300 DPI)

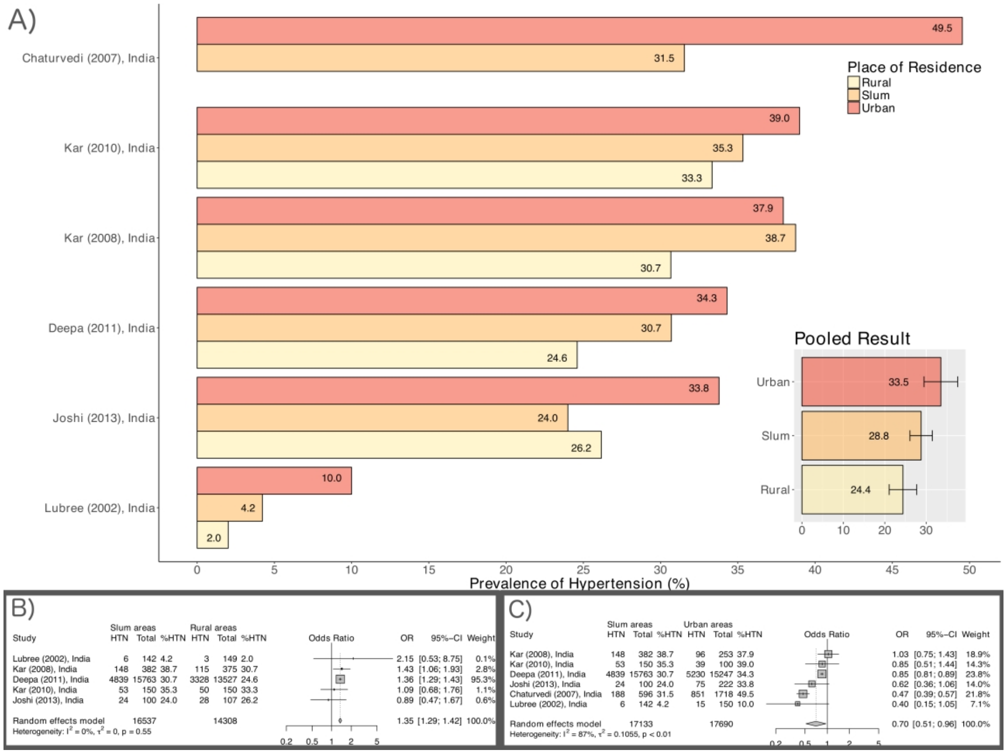

Figure 5

478x357mm (300 x 300 DPI)

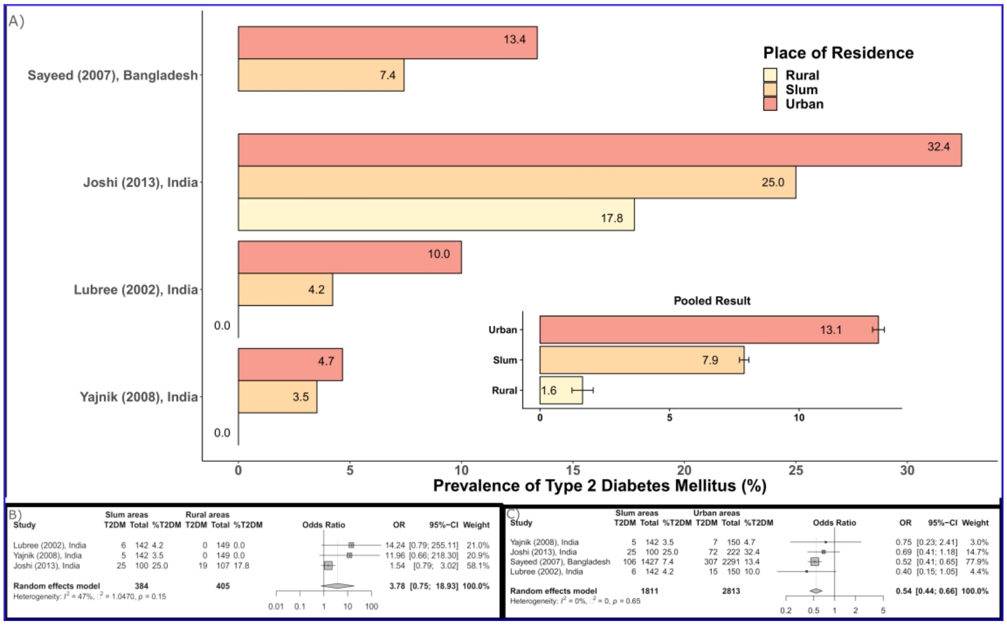

Figure 6

425x261mm (300 x 300 DPI)

Supplementary Digital Content

Table of Contents

eFigure 1: Study selection and inclusion flow chart 1

eTable 1: List of Excluded Studies 2

eTable 2: Characteristics of included studies 10

eTable 3: Risk of bias of included studies 12

Annex 1: MEDLINE Search Strategy 14

Annex 2: PRISMA Checklist 15

For peer review only

eFigure 1: Study selection and inclusion flow chart

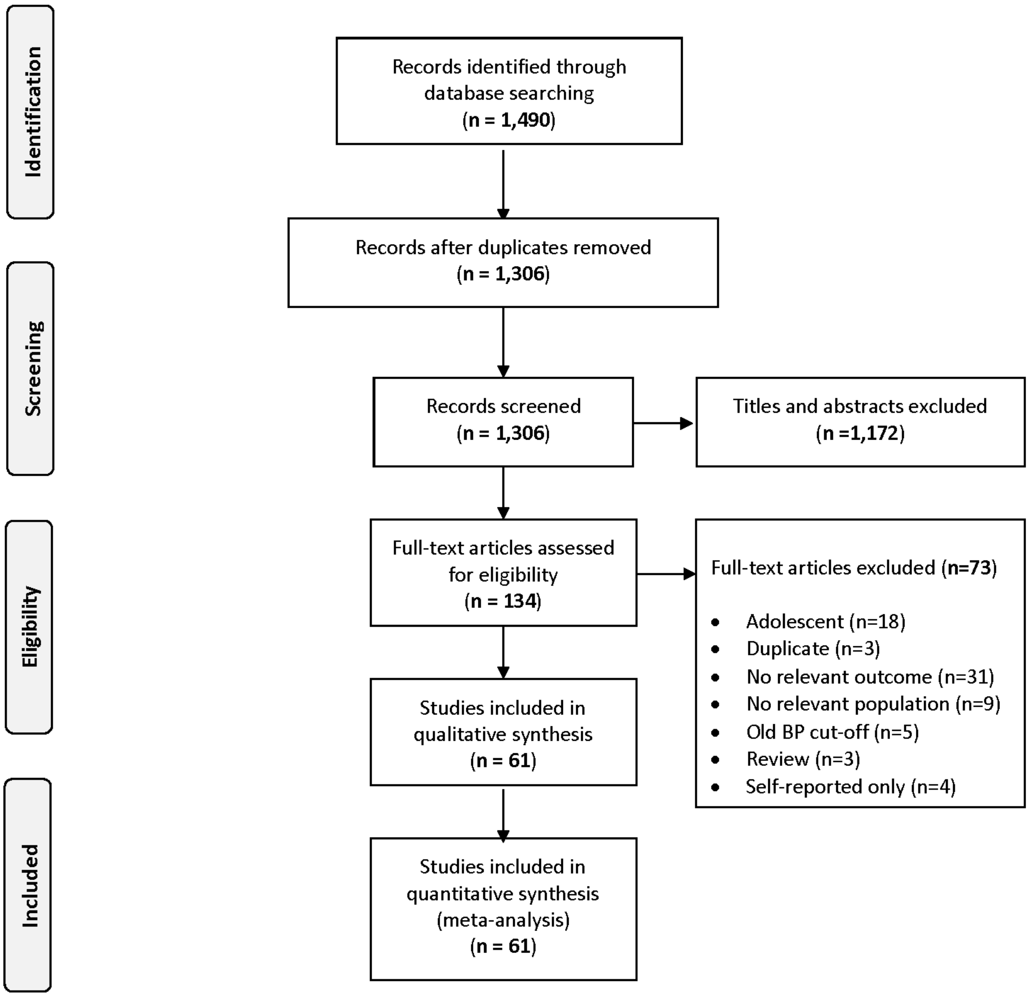

For peer review only - http://bmjopen.bmj.com/site/about/guidelines.xhtml

eTable 1: List of Excluded Studies

s/n	Study	Reason
1	Maiti 2016[1]	Adolescent
2	Khopkar 2015[2]	Adolescent
3	Paul 2013[3]	Adolescent
4	Kamath 2012[4]	Adolescent
5	Simsek 2012[5]	Adolescent
6	Saha 2011[6]	Adolescent
7	Oria 2010[7]	Adolescent
8	Saha 2008[8]	Adolescent
9	Saha 2008[9]	Adolescent
10	Sesso 2004[10]	Adolescent
11	Fernandes 2003[11]	Adolescent
12	Zeelie 2010[12]	Adolescent
13	Soudrassanane 2008[13]	Adolescent
14	Werner 2015[14]	Duplicate
15	van de Vijver 2016[15]	Duplicate
16	Haregu 2016[16]	Duplicate
17	Ezenwaka 1997[17]	Old BP cut-off
18	Suriyawongpaisal 1993[18]	Old BP cut-off
19	Suriyawongpaisal 1991[19]	Old BP cut-off
20	Sitthi-Amornn 1989[20]	Old BP cut-off
21	Bunnag 1990[21]	Old BP cut-off
22	E. Sharmin Trisha 2016[22]	No relevant outcome
23	Bhandari 2015[23]	No relevant outcome
24	Oti 2014[24]	No relevant outcome
25	Hiremath 2014[25]	No relevant outcome
26	Joshi 2013[26]	No relevant outcome
27	van de Vijver 2013[27]	No relevant outcome
28	Itrat 2011[28]	No relevant outcome
29	Ahmed 2011[29]	No relevant outcome
30	Haregu 2015[30]	No relevant outcome
31	van de Vijver 2015[31]	No relevant outcome
32	Kohli 2016[32]	No relevant outcome
33	Mudgapalli 2016[33]	No relevant population
34	Natarajan 2014[34]	No relevant population
35	Kumaramanickavel 2014[35]	No relevant population
36	Kumaramanickavel 2015[36]	No relevant population
37	Hulzebosch 2015[37]	No relevant population
38	Madhu 2016[38]	No relevant population
39	Mugure 2014[39]	No relevant population
40	Mukhopadhyay 2012[40]	No relevant population
41	Khan 2010[41]	No relevant population
42	Etyang 2013[42]	Review
43	Dhar 2014[43]	Review
44	Bhargava 1991[44]	Review
45	Khalequzzaman 2017[45]	Self-reported only
46	Kien 2015[46]	Self-reported only
47	Sur 2007[47]	Self-reported only
48	Thakur 2013[48]	Self-reported only
49	Ahmedani 2019[49]	No relevant outcome
50	Ashe 2019[50]	No relevant outcome
51	Asiki 2018[51]	No relevant outcome
52	Bagdey 2019[52]	No relevant outcome
53	Cope 2020[53]	No relevant outcome
54	De Silva 2018[54]	No relevant outcome
55	Kapwata 2018[55]	No relevant outcome

For peer review only - http://bmjopen.bmj.com/site/about/guidelines.xhtml

56	Kawazoe 2018 [56]	No relevant outcome
57	Khanam 2019[57]	No relevant outcome
58	Kolak 2018[58]	No relevant outcome
59	Korn 2018[59]	No relevant outcome
60	Kotian 2019[60]	No relevant outcome
61	Kumar 2018[61]	No relevant outcome
62	Ma 2018[62]	No relevant outcome
63	Maharana 2019[63]	No relevant outcome
64	Nagarkar 2018[64]	No relevant outcome
65	Narendran 2018[65]	No relevant outcome
66	Rajapakshe 2018[66]	No relevant outcome
67	Sarkar 2019[67]	No relevant outcome
68	Scazufca 2019[68]	No relevant outcome
69	Wang 2018[69]	No relevant outcome
70	Wekasah 2020[70]	No relevant outcome
71	Wilson 2020[71]	No relevant outcome
72	Yadav 2018[72]	No relevant outcome
73	Zhang 2019[73]	No relevant outcome

For peer review only

For peer review only - http://bmjopen.bmj.com/site/about/guidelines.xhtml

List of excluded studies

1. Maiti M, Bandyopadhyay L. Variation in blood pressure among adolescent schoolchildren in an urban slum of Kolkata, West Bengal. *Postgraduate Medical Journal (no pagination), 2016* 2016;Date of Publication:July 25. doi: http://dx.doi.org/10.1136/postgradmedj-2016-134227
2. Khopkar SA, Virtanen SM, Kulathinal S. Mental health, anthropometry and blood pressure among adolescents living in slums of Nashik, India. *Tanzania Journal of Health Research* 2015;17(4) doi: http://dx.doi.org/10.4314/thrb.v17i4.6
3. Paul B, Saha I, Mukherjee A. Adolescent Hypertension and Family History. *Pakistan Paediatric Journal* 2013;37(3):177-79.
4. Kamath N, Goud BR, Phadke KD, et al. Use of oscillometric devices for the measurement of blood pressure-comparison with the gold standard. *Indian Journal of Pediatrics* 2012;79(9):1230-32. doi: http://dx.doi.org/10.1007/s12098-011-0600-0
5. Simsek E, Selver B, Dallar Y, et al. Obesity epidemiology in children living in the lower socio-economic status. *Hormone Research in Paediatrics* 2012;Conference:51st Annual Meeting of the European Society for Paediatric Endocrinology. doi: http://dx.doi.org/10.1159/000343184
6. Saha I, Paul B, Mukherjee A, et al. Validity of the WHO criteria for adolescent hypertension. *East African journal of public health* 2011;8(2):135-37.
7. Oria RB, Patrick PD, Oria MOB, et al. ApoE polymorphisms and diarrheal outcomes in Brazilian shanty town children. *Brazilian Journal of Medical and Biological Research* 2010;43(3):249-56.
8. Saha I, Paul B, Dasgupta A. Prevalence of hypertension and variation of blood pressure with age among adolescents in Chetla, India. *Tanzania journal of health research* 2008;10(2):108-11.
9. Saha I, Paul B, Dasgupta A, et al. Variations of adolescent blood pressure by multifactorial analysis in an urban slum of Kolkata. *Journal of the Indian Medical Association* 2008;106(9)
10. Sesso R, Barreto GP, Neves J, et al. Malnutrition is associated with increased blood pressure in childhood. *Nephron Clinical Practice* 2004;97(2):c61-c66. doi: http://dx.doi.org/10.1159/000078402
11. Fernandes MTB, Sesso R, Martins PA, et al. Increased blood pressure in adolescents of low socioeconomic status with short stature. *Pediatric Nephrology* 2003;18(5):435-39.
12. Zeelie A, Moss SJ, Kruger HS. The relationship between body composition and selected metabolic syndrome markers in black adolescents in South Africa: the PLAY study. *Nutrition* 2010;26(11-12):1059-64. doi: 10.1016/j.nut.2010.03.001 [published Online First: 2010/06/15]
13. Soudarssanane M, Mathanraj S, Sumanth M, et al. Tracking of blood pressure among adolescents and young adults in an urban slum of puducherry. *Indian journal of community medicine : official publication of Indian Association of Preventive & Social Medicine* 2008;33(2):107-12. doi: 10.4103/0970-0218.40879 [published Online First: 2008/04/01]
14. Werner ME, van de Vijver S, Adhiambo M, et al. Results of a hypertension and diabetes treatment program in the slums of Nairobi: a retrospective cohort study. *BMC health services research* 2015;15(pp 512) doi: http://dx.doi.org/10.1186/s12913-015-1167-7
15. van de Vijver S, Oti SO, Gomez GB, et al. Impact evaluation of a community-based intervention for prevention of cardiovascular diseases in the slums of Nairobi: the SCALE-UP study. *Glob Health Action* 2016;9(1):30922. doi: 10.3402/gha.v9.30922 [published Online First: 2017/02/06]

For peer review only - http://bmjopen.bmj.com/site/about/guidelines.xhtml

16. Haregu TN, Oti S, Egondi T, et al. Measurement of overweight and obesity an urban slum setting in sub-Saharan Africa: a comparison of four anthropometric indices. *BMC obesity* 2016;3:46. doi: 10.1186/s40608-016-0126-0 [published Online First: 2016/11/12]
17. Ezenwaka CE, Akanji AO, Akanji BO, et al. The prevalence of insulin resistance and other cardiovascular disease risk factors in healthy elderly southwestern Nigerians. *Atherosclerosis* 1997;128(2):201-11. doi: http://dx.doi.org/10.1016/S0021-9150%2896%2905991-6
18. Suriyawongpaisal P, Underwood P. Situation of hypertension in some Bangkok slums. *Journal of the Medical Association of Thailand = Chotmaihet thangphaet* 1993;76(3):123-28.
19. Suriyawongpaisal P, Underwood P, Rouse IL, et al. An investigation of hypertension in a slum of Nakhon Ratchasima. *The Southeast Asian journal of tropical medicine and public health* 1991;22(4):586-94.
20. Sitthi-Amorn C, Chandraprasert S, Bunnag SC, et al. The prevalence and risk factors of hypertension in Klong Toey Slum and Klong Toey government apartment houses. *International Journal of Epidemiology* 1989;18(1):89-94.
21. Bunnag SC, Sitthi-Amorn C, Chandraprasert S. The prevalence of obesity, risk factors and associated diseases in Klong Toey slum and Klong Toey government apartment houses. *Diabetes Res Clin Pract* 1990;10(1)
22. N EST, Jelinek HF, Tarvainen MP, et al. Socioeconomic status, age and heart rate variability in a Bangladeshi community. *Conference proceedings : Annual International Conference of the IEEE Engineering in Medicine and Biology Society IEEE Engineering in Medicine and Biology Society Annual Conference* 2016;01 doi: http://dx.doi.org/10.1109/EMBC.2016.7591919
23. Bhandari S, Sarma PS, Thankappan KR. Adherence to antihypertensive treatment and its determinants among urban slum dwellers in Kolkata, India. *Asia Pacific journal of public health / Asia Pacific Academic Consortium for Public Health* 2015;27(2) doi: http://dx.doi.org/10.1177/1010539511423568
24. Oti SO, van de Vijver S, Kyobutungi C. Trends in non-communicable disease mortality among adult residents in Nairobi's slums, 2003-2011: applying InterVA-4 to verbal autopsy data. *Global health action* 2014;7(pp 25533) doi: http://dx.doi.org/10.3402/gha.v7.25533
25. Hiremath RN, Venkatesh G, Sharvesh, et al. Hypertension status and awareness among geriatric population living in Urban slum. *Nepal Journal of Epidemiology* 2014;Conference:International Conference on Research Methodology and Scientific Writing.
26. Joshi A, Mehta S, Grover A, et al. Knowledge, attitude, and practices of individuals to prevent and manage metabolic syndrome in an Indian setting. *Diabetes Technology and Therapeutics* 2013;15(8):644-53. doi: http://dx.doi.org/10.1089/dia.2012.0309
27. van de Vijver SJ, Oti SO, Agyemang C, et al. Prevalence, awareness, treatment and control of hypertension among slum dwellers in Nairobi, Kenya. *Journal of hypertension* 2013;31(5):1018-24. doi: 10.1097/HJH.0b013e32835e3a56 [published Online First: 2013/02/22]
28. Itrat A, Ahmed B, Khan M, et al. Risk factor profiles of South Asians with cerebrovascular disease. *International Journal of Stroke* 2011;6(4):346-48. doi: http://dx.doi.org/10.1111/j.1747-4949.2011.00622.x
29. Ahmed B, Itrat A, Khan M, et al. Risk factor profiles of south asians with cerebrovascular disease: Findings from a community-based prevalence study in semiurban Pakistan. *Circulation: Cardiovascular Quality and Outcomes* 2011;Conference:Quality of Care

For peer review only - http://bmjopen.bmj.com/site/about/guidelines.xhtml

and Outcomes Research in Cardiovascular Disease and Stroke 2011 Scientific Sessions.
30. Haregu TN, Oti S, Egondi T, et al. Co-occurrence of behavioral risk factors of common non-communicable diseases among urban slum dwellers in Nairobi, Kenya. *Glob Health Action* 2015;8(28697) doi: https://dx.doi.org/10.3402/gha.v8.28697
31. van de Vijver S, Oti S, Moll van Charante E, et al. Cardiovascular prevention model from Kenyan slums to migrants in the Netherlands. *Global health* 2015;11(11):07. doi: https://dx.doi.org/10.1186/s12992-015-0095-y
32. Kohli C, Gupta K. LBOS 03-03 ECONOMIC IMPACT OF HYPERTENSION. *Journal of hypertension* 2016;34 Suppl 1 - ISH 2016 Abstract Book:e551-e52. doi: 10.1097/01.hjh.0000501509.98288.ad [published Online First: 2016/10/19]
33. Mudgapalli V, Sharan S, Amadi C, et al. Perception of receiving SMS based health messages among hypertensive individuals in urban slums. *Technology and Health Care* 2016;24(1):57-65. doi: http://dx.doi.org/10.3233/THC-151097
34. Natarajan S, Mohan S, Satagopan U, et al. Elderly patients with T2DM should be periodically screened for diabetic retinopathy and its complications to reduce visual morbidity - A study from slums of Western India. *Investigative Ophthalmology and Visual Science* 2014;Conference:2014 Annual Meeting of the Association for Research in Vision and Ophthalmology.
35. Kumaramanickavel G, Mohan S, Satagopan U, et al. Diabetic retinopathy in urban slums of Mumbai, India - Social, lifestyle, clinical and genetic risk factors. *Investigative Ophthalmology and Visual Science* 2014;Conference:2014 Annual Meeting of the Association for Research in Vision and Ophthalmology.
36. Kumaramanickavel G, Mohan S, Kumar Singh A, et al. AJDRUMSS-diabetic retinopathy prevalence study in Mumbai slums of India-association of demographic, genetic and medical risk factors. *Investigative Ophthalmology and Visual Science* 2015;Conference:2015 Annual Meeting of the Association for Research in Vision and Ophthalmology.
37. Hulzebosch A, van de Vijver S, Oti SO, et al. Profile of people with hypertension in Nairobi's slums: a descriptive study. *Globalization and health* 2015;11(pp 26) doi: http://dx.doi.org/10.1186/s12992-015-0112-1
38. Madhu B, Srinath KM, Chandresh S, et al. Quality of diabetic care in an urban slum area of Mysore: A community based study. *Diabetes and Metabolic Syndrome: Clinical Research and Reviews* 2016 doi: http://dx.doi.org/10.1016/j.dsx.2016.03.014
39. Mugure G, Karama M, Kyobutungi C, et al. Correlates for cardiovascular diseases among diabetic/hypertensive patients attending outreach clinics in two Nairobi slums, Kenya. *Pan African Medical Journal* 2014;19(no pagination) doi: http://dx.doi.org/10.11604/pamj.2014.19.261.5261
40. Mukhopadhyay A, Sundar U, Adwani S, et al. Prevalence of stroke and post-stroke cognitive impairment in the elderly in Dharavi, Mumbai. *Journal of Association of Physicians of India* 2012;60(10):29-32.
41. Khan RMA, Ahmad M. To assess the public awareness about obesity among adult populace of lahore. *Pakistan Journal of Medical and Health Sciences* 2010;4(4)
42. Etyang A, Harding S, Cruickshank JK. Slum living and hypertension in tropical settings: Neglected issue, statistical artifact or surprisingly slight? Insights amidst adversity. *Journal of Hypertension* 2013;31(5):877-79. doi: http://dx.doi.org/10.1097/HJH.0b013e32836103fb
43. Dhar L. Preventing coronary heart disease risk of slum dwelling residents in India. *Journal of family medicine and primary care* 2014;3(1):58-62. doi: 10.4103/2249-4863.130278 [published Online First: 2014/05/03]

For peer review only - http://bmjopen.bmj.com/site/about/guidelines.xhtml

44. Bhargava SK, Singh KK, Saxena BN. ICMR Task Force National Collaborative Study on Identification of High Risk Families, Mothers and Outcome of their Off-springs with particular reference to the problem of maternal nutrition, low birth weight, perinatal and infant morbidity and mortality in rural and urban slum communities. Summary, conclusions and recommendations. *Indian pediatrics* 1991;28(12):1473-80. [published Online First: 1991/12/01]
45. Khalequzzaman M, Chiang C, Hoque BA, et al. Population profile and residential environment of an urban poor community in Dhaka, Bangladesh. *Environmental Health and Preventive Medicine* 2017;22(1) doi: http://dx.doi.org/10.1186/s12199-017-0610-2
46. Kien VD, Van Minh H, Giang KB, et al. Socioeconomic inequalities in self-reported chronic non-communicable diseases in urban Hanoi, Vietnam. *Global Public Health* 2015 doi: http://dx.doi.org/10.1080/17441692.2015.1123282
47. Sur D, Mukhopadhyay SP. A study on smoking habits among slum dwellers and the impact on health and economics. *Journal of the Indian Medical Association* 2007;105(9):492-98.
48. Thakur R, Banerjee A, Nikumb V. Health problems among the elderly: a cross-sectional study. *Annals of medical and health sciences research* 2013;3(1):19-25. doi: 10.4103/2141-9248.109466 [published Online First: 2013/05/02]
49. Ahmedani MY, Fawwad A, Shaheen F, et al. Optimized health care for subjects with type 1 diabetes in a resource constraint society: A three-year follow-up study from Pakistan. *World J Diabetes* 2019;10(3):224-33. doi: 10.4239/wjd.v10.i3.224
50. Ashe S, Routray D. Prevalence, associated risk factors of depression and mental health needs among geriatric population of an urban slum, Cuttack, Odisha. *International Journal of Geriatric Psychiatry* 2019;34(12):1799-807. doi: 10.1002/gps.5195
51. Asiki G, Mohamed SF, Wambui D, et al. Sociodemographic and behavioural factors associated with body mass index among men and women in Nairobi slums: AWI-Gen Project. *Global health action* 2018;11(sup2):1470738-38. doi: 10.1080/16549716.2018.1470738
52. Bagdey PS, Ansari JA, Barnwal RK. Prevalence and epidemiological factors associated with hypertension among post-menopausal women in an urban area of central India. *Clinical Epidemiology and Global Health* 2019;7(1):111-14. doi: 10.1016/j.cegh.2018.02.008
53. Cope AB, Edmonds A, Ludema C, et al. Neighborhood Poverty and Control of HIV, Hypertension, and Diabetes in the Women's Interagency HIV Study. *AIDS Behav* 2020;24(7):2033-44. doi: 10.1007/s10461-019-02757-5
54. De Silva AP, De Silva SHP, Haniffa R, et al. Inequalities in the prevalence of diabetes mellitus and its risk factors in Sri Lanka: a lower middle income country. *Int J Equity Health* 2018;17(1):45-45. doi: 10.1186/s12939-018-0759-3
55. Kapwata T, Manda S. Geographic assessment of access to health care in patients with cardiovascular disease in South Africa. *BMC health services research* 2018;18(1):197-97. doi: 10.1186/s12913-018-3006-0
56. Kawazoe N, Zhang X, Chiang C, et al. Prevalence of hypertension and hypertension control rates among elderly adults during the cold season in rural Northeast China: a cross-sectional study. *J Rural Med* 2018;13(1):64-71. doi: 10.2185/jrm.2959 [published Online First: 2018/05/29]
57. Khanam F, Hossain MB, Mistry SK, et al. Prevalence and Risk Factors of Cardiovascular Diseases among Bangladeshi Adults: Findings from a Cross-sectional Study. *J Epidemiol Glob Health* 2019;9(3):176-84. doi: 10.2991/jegh.k.190531.001

For peer review only - http://bmjopen.bmj.com/site/about/guidelines.xhtml

58. Kolak M, Bradley M, Block DR, et al. Urban foodscape trends: Disparities in healthy food access in Chicago, 2007–2014. *Health & Place* 2018;52:231-39. doi: 10.1016/j.healthplace.2018.06.003
59. Korn A, Bolton SM, Spencer B, et al. Physical and Mental Health Impacts of Household Gardens in an Urban Slum in Lima, Peru. *Int J Environ Res Public Health* 2018;15(8):1751. doi: 10.3390/ijerph15081751
60. Kotian S, Waingankar P, Mahadik V. Assessment of compliance to treatment of hypertension and diabetes among previously diagnosed patients in urban slums of Belapur, Navi Mumbai, India. *Indian Journal of Public Health* 2019;63(4):348. doi: 10.4103/ijph.ijph_422_18
61. Kumar R, Kaur N, Pilania M. Morbidity Pattern of Patients Attending a Primary Healthcare Facility in an Urban Slum of Chandigarh, India. *JOURNAL OF CLINICAL AND DIAGNOSTIC RESEARCH* 2018 doi: 10.7860/jcdr/2018/31331.11297
62. Ma C. The prevalence of depressive symptoms and associated factors in countryside-dwelling older Chinese patients with hypertension. *Journal of Clinical Nursing* 2018;27(15-16):2933-41. doi: 10.1111/jocn.14349
63. Maharana S, Garg S, Dasgupta A, et al. A study on impact of oral health on general health among the elderly residing in a slum of Kolkata: A cross-sectional study. *Indian Journal of Dental Research* 2019;30(2):164. doi: 10.4103/ijdr.ijdr_491_17
64. Nagarkar AM, Kulkarni SS. Obesity and its Effects on Health in Middle-Aged Women from Slums of Pune. *J Midlife Health* 2018;9(2):79-84. doi: 10.4103/jmh.JMH_8_18
65. Narendran M, Rani BBS, Kulkarni P, et al. Interdependence of communicable and Non-Communicable diseases among elderly population in declared slum in Mysuru City, Karnataka. *Indian Journal of Public Health Research & Development* 2018;9(11):62. doi: 10.5958/0976-5506.2018.01426.2
66. Rajapakshe OBW, Sivayogan S, Kulatunga PM. Prevalence and correlates of depression among older urban community-dwelling adults in Sri Lanka. *Psychogeriatrics* 2018;19(3):202-11. doi: 10.1111/psyg.12389
67. Sarkar A, Roy D, Chauhan MM, et al. A lay epidemiological study on coexistent stress in hypertension: Its prevalence, risk factors, and implications in patients' lives. *Journal of family medicine and primary care* 2019;8(3):966-71. doi: 10.4103/jfmpc.jfmpc_60_19
68. Scazufca M, de Paula Couto MCP, Henrique MG, et al. Pilot study of a two-arm non-randomized controlled cluster trial of a psychosocial intervention to improve late life depression in socioeconomically deprived areas of São Paulo, Brazil (PROACTIVE): feasibility study of a psychosocial intervention for late life depression in São Paulo. *BMC public health* 2019;19(1):1152-52. doi: 10.1186/s12889-019-7495-5
69. Wang H, Su M, Fang P-q, et al. Analysis on Medical Expenses of Hypertensive Inpatients in Urban Areas from 2010 to 2013—Evidence from Two Provinces in South of China. *Current Medical Science* 2018;38(4):741-48. doi: 10.1007/s11596-018-1939-5
70. Wekesah FM, Klipstein-Grobusch K, Grobbee DE, et al. Determinants of Mortality from Cardiovascular Disease in the Slums of Nairobi, Kenya. *Glob Heart* 2020;15(1):33-33. doi: 10.5334/gh.787
71. Wilson V, Nittoori S. Risk of type 2 diabetes mellitus among urban slum population using Indian Diabetes Risk Score. *Indian Journal of Medical Research* 2020;152(3):308. doi: 10.4103/ijmr.ijmr_1597_18
72. Yadav S, Saraswat N, Saini AK, et al. A REVIEW ON THE PREVALENCE OF HYPERTENSION IN SIDE-LINED POPULATIONS; SLUM DWELLERS, SHIFT JOB WORKERS AND OCCUPATIONAL NOISE AFFECTED WORKERS: ATTRIBUTABLE TO LIFESTYLE AND ENVIRONMENTAL FACTOR. *Asian*

For peer review only - http://bmjopen.bmj.com/site/about/guidelines.xhtml

Journal of Pharmaceutical and Clinical Research 2018;11(10):18. doi: 10.22159/ajpcr.2018.v11i10.27007
73. Zhang X, Chen X, Gong W. Type 2 diabetes mellitus and neighborhood deprivation index: A spatial analysis in Zhejiang, China. *J Diabetes Investig* 2019;10(2):272-82. doi: 10.1111/jdi.12899 [published Online First: 2018/08/28]

For peer review only

For peer review only - http://bmjopen.bmj.com/site/about/guidelines.xhtml

eTable 2: Characteristics of included studies

Study	Country	Slum	Sample size	Age group	% female
Acharyya (2014)	India	North-Parganas	1052	25-64	49.8
Ahmad (2014)	India	Meerut	196	>60	50
Akinwale (2013)	Nigeria	Ijora Oloye, Ajegunle & Makoko	2434		
Anand (2007)	India	Faridabad	2562	15+	50.9
Ayah (2013)	Kenya		2061	18-90	49.1
Banerjee (2016)	India	Kolkata	10167	>20 years	60
Chakerborty (2012)	India	Kolkata	470	18-60	0
Chaturvedi (2007)	India	Delhi	596	>20	
Daniel (2013)	Nigeria	Ajegunle	964	20-81	65.8
Dasappa (2015)	India	Bangalore	2013	35+	50.8
Deepa (2011)	India	Ballabgarh, Delhi, Chennai, Trivandrum , Dibrugarh and Nagpur	15763	15-64	
Edwards (2015)	Kenya	Kibera			
Ezeala-Adikaibe (2016)	Nigeria	Enugu	774	⩾ 20	64.7
Ferreira (2005)	Brazil	Maceio	223	18-65	100
Florencio (2004)	Brazil	Maceio	416	18-60	57
Haregu (2016)	Kenya	Nairobi	5190	18+	46.2
Heitzinger (2014)	Peru	Lima	142	18-81	69.7
Huda (2012)	Bangladesh	Mirpur, Dhaka	1000	15-65	33.4
Jalil (2008)	Pakistan	Lahore	695		43.6
Joshi (2013)	India	Rourkela & Bhubaneswar	100	>18	69
Joshi (2014)	Kenya	Kibera	2045	18-90	49.1
Kar (2008)	India	Chandigarh & Haryana	1010	>30	58.9
Kar (2010)	India	Chandigarh & Haryana	150	>30	62
Kumari (2014)	India	Hyderabad	250		78
Lubree (2002)	India	Pune	150	30-50	100
Marins (2007)	Brazil	Rio-de-Janeiro	3279	>20	56.9
Misra (2001)	India	Gautam-Nagar, Delhi	532		68
Nirmala (2014)	India	Hyderabad, Telangana	700	>20	50.8
Olack (2015)	Kenya	Kibera	1528	35-64	58.1
Oli (2013)	Nepal	Kathmandu	689	15-64	58.9
Ongeti (2013)	Kenya	Kibera	400	14-75	70.3
Oti (2013)	Kenya	Viwandani & Korogocho		18+	46
Patil (2016)	India	Pune, Maharashtra	425	20+	
Rahim (2004)	Bangladesh	Dhakar	1555	20+	52.99
Rawal (2017)	Bangladesh	Dhaka	507		50
Sayeed (2007)	Bangladesh	Dhakar			59.2
Singh (b) (2012)	India	Delhi	474	60+	48
Singh (2012)	India	Patna	3118	>30	56.5
Sinha (2010)	India	Gokulpuri	275	18-40	100
Sithi-Amorn (1989)	Thailand	Klong-Toey	976		54.7
Snyder (2017)	Brazil		792		64.5

For peer review only - http://bmjopen.bmj.com/site/about/guidelines.xhtml

Sowemimo (2015)	Nigeria	Yemetu, Ibadan	806	18-90	
Sunita (2017)	India	Mumbai	6464	>40	
Unger (2015)	Brazil	Salvador	5649	>18	58.3
Uthakalla (2012)	India	Hyderabad		20-60	56
Vigneswari (2014)	India	Chennai	529	18+	77.3
Vigneswari (2015)	India		529	18+	77.3
Vikram (2003)	India	New-Delhi	639		73.4
Wasir (2007)	India	Delhi	278		
Yajnik (2008)	India		142	30-50	0
van de Vijver (2013)	Kenya	Viwandani & Korogocho	5190	>18	46.2
Bawah (2019)	Ghana	Accra	2009		
Chiang (2019)	Bangladesh	Dhaka	423		
Choudhury (2018)	Bangladesh	Dhaka	984	43.4	73
Dwivedi (2018)	India	Bangalore			
Gadallah (2018)	Egypt	West Delhi			
George (2019)	India	Bangalore		57.6	
Gonmei (2018)	India	Delhi			
Jain (2019)	India	Delhi	984	43.4	73
Tymejczyk (2019)	Haiti	Gurugram	420		
Vusirikala (2019)	Kenya	Nairobi		57.6	

For peer review only - http://bmjopen.bmj.com/site/about/guidelines.xhtml

eTable 3: Risk of bias of included studies

Study	Selection (sample population)	Selection (participation rate)	Performance bias (analytical methods to control for bias)	Other form of bias
Acharyya (2014)	Low risk	Low risk	Low risk	Low risk
Ahmad (2014)	Low risk	Unclear risk	High risk	Unclear risk
Akinwale (2013)	Low risk	Low risk	High risk	Unclear risk
Anand (2007)	Low risk	Low risk	Low risk	Low risk
Ayah (2013)	Low risk	Low risk	Low risk	Low risk
Banerjee (2016)	Low risk	Unclear risk	Low risk	Low risk
Chakerborty (2012)	High risk	Low risk	High risk	Low risk
Chaturvedi (2007)	Low risk	Low risk	Low risk	Low risk
Daniel (2013)	Low risk	Low risk	Low risk	Low risk
Dasappa (2015)	Low risk	Low risk	High risk	Low risk
Deepa (2011)	Low risk	Low risk	High risk	Low risk
Edwards (2015)	Low risk	Low risk	High risk	Unclear risk
Ezeala-Adikaibe (2016)	High risk	High risk	Low risk	Low risk
Ferreira (2005)	Low risk	Low risk	Low risk	Low risk
Florencio (2004)	Low risk	Low risk	Low risk	Low risk
Haregu (2016)	Unclear risk	Unclear risk	Low risk	Low risk
Heitzinger (2014)	Low risk	Low risk	Low risk	Low risk
Huda (2012)	Low risk	Low risk	High risk	Unclear risk
Jalil (2008)	Low risk	Low risk	Low risk	Unclear risk
Joshi (2013)	High risk	Low risk	Low risk	High risk
Joshi (2014)	Low risk	Low risk	Low risk	Low risk
Kar (2008)	Low risk	Low risk	Low risk	Low risk
Kar (2010)	Low risk	Low risk	Low risk	Low risk
Kumari (2014)	Low risk	Low risk	High risk	Low risk
Lubree (2002)	Low risk	Low risk	High risk	Low risk
Marins (2007)	Low risk	Low risk	High risk	Unclear risk
Misra (2001)	Low risk	Low risk	High risk	Low risk
Nirmala (2014)	Low risk	Low risk	High risk	Low risk
Olack (2015)	Low risk	Low risk	Low risk	Low risk
Oli (2013)	Low risk	Low risk	Low risk	Low risk
Ongeti (2013)	Low risk	Low risk	Low risk	Low risk
Oti (2013)	Low risk	Low risk	Low risk	Low risk
Patil (2016)	Low risk	Low risk	High risk	Unclear risk
Rahim (2004)	Low risk	Low risk	High risk	Unclear risk
Rawal (2017)	Low risk	Low risk	Low risk	Low risk
Sayeed (2007)	Low risk	Low risk	High risk	Unclear risk
Singh (b) (2012)	Low risk	Low risk	Low risk	Unclear risk
Singh (2012)	Low risk	Low risk	Low risk	Low risk
Sinha (2010)	Low risk	Low risk	Low risk	Low risk
Sithi-Amorn (1989)	Low risk	Low risk	High risk	Unclear risk
Snyder (2017)	Low risk	Low risk	Low risk	Low risk
Sowemimo (2015)	Low risk	Unclear risk	Low risk	Unclear risk

For peer review only

Sunita (2017)	Low risk	Low risk	High risk	Unclear risk
Unger (2015)	Low risk	Low risk	Low risk	Low risk
Uthakalla (2012)	Low risk	Low risk	High risk	Unclear risk
Vigneswari (2014)	Low risk	Low risk	High risk	Low risk
Vigneswari (2015)	Low risk	Low risk	High risk	Low risk
Vikram (2003)	Low risk	Low risk	Low risk	Low risk
Wasir (2007)	Low risk	High risk	High risk	Unclear risk
Yajnik (2008)	Low risk	Low risk	High risk	Unclear risk
van de Vijver (2013)	Low risk	Low risk	Low risk	Low risk
Bawah (2019)	Unclear risk	Unclear risk	Low risk	Unclear risk
Chiang (2019)	Low risk	Low risk	Low risk	Low risk
Choudhury (2018)	Low risk	Low risk	Low risk	Low risk
Dwivedi (2018)	Low risk	Low risk	Low risk	Low risk
Gadallah (2018)	Low risk	Low risk	Low risk	Low risk
George (2019)	Low risk	Low risk	Low risk	Low risk
Gonmei (2018)	Unclear risk	Unclear risk	Unclear risk	Unclear risk
Jain (2019)	Low risk	Low risk	Low risk	Low risk
Tymejczyk (2019)	Low risk	Low risk	Low risk	Low risk
Vusirikala (2019)	Low risk	Low risk	Low risk	Low risk

For peer review only

For peer review only - http://bmjopen.bmj.com/site/about/guidelines.xhtml

Annex 1: MEDLINE Search Strategy

1 exp hypertension/
2 hypertens$.mp.
3 exp blood pressure/
4 (blood pressure or bloodpressure).mp.
5 (essential adj3 hypertension).ti,ab.
6 (isolat* adj3 hypertension).ti,ab.
7 (elevat* adj3 blood adj pressur*).ti,ab.
8 (high adj3 blood adj pressur*).ti,ab.
9 (increase* adj3 blood pressur*).ti,ab.
10 ((systolic or diastolic or arterial) adj3 pressur*).ti,ab.
11 essential hypertension.mp.
12 isolated hypertension.mp.
13 elevated blood pressure.mp.
14 high blood pressure.mp.
15 increase blood pressure.mp.
16 diastolic pressure.mp.
17 pre-hypertension.mp.
18 pre-hypertensive.mp.
19 prehypertension.mp.
20 prehypertensive.mp.
21 arterial pressure.mp.
22 cardiovascular diseases/
23 exp coronary disease/
24 cardiovascular risk factor$.tw.
25 (cardiovascular adj3 disease$).tw.
26 (Coronary adj3 disease$).tw.
27 heart disease$.tw.
28 coronary risk factor$.tw.
29 or/1-28

1 exp Diabetes Mellitus, Type 2/
2 exp DIABETES MELLITUS/
3 T2DM.ti,ab.
4 (Type* adj3 ("2" or "II" or two*) adj3 (diabete* or diabetic*)).tw.
5 ((Maturit* or adult* or slow*) adj3 onset* adj3 (diabete* or diabetic*)).tw.
6 ((Ketosis-resistant* or stable*) adj3 (diabete* or diabetic*)).tw.
7 ((Non-insulin* or Non insulin* or Noninsulin*) adj3 depend* adj3 (diabete* or diabetic*)).tw.
8 IDDM.ti,ab.
9 diabet$.ti.
10 PREDIABETIC STATE/
11 prediabet$.ti,ab.
12 impaired glucose tolerance.ti,ab.
13 IGT.ti,ab.
14 Impaired fasting glucose.ti,ab.
15 IFG.ti,ab.
16 Impaired glucose regulation.ti,ab. 1
17 IGR.ti,ab.
18 GLUCOSE INTOLERANCE/
19 (diabet* or glucose or hyperglycaemia or hyperglycaemia or postprandial or post-prandial or insulin or hypoglycemia or hypoglycaemia or IGT or OGTT or CGMS).tw.
20 (subclinical diabetes" or "subclinical diabetic" or "sub-clinical diabetes" or "sub-clinical diabetic").tw.
21 or/1-20

22 (baladi or bandas de miseria or barraca or barrio marginal or barrio or bidonville or brarek or bustee or chalis or chereka bete or dagatan or estero or favela or galoos or gecekondu or hrushebi).mp.
23 (ishash or karyan or katras or looban or loteamento or medina achouaia or morro or mudun safi or musseque or solares or tanake or taudis or township or tugurio or udukku or umjondolo or watta or zopadpattis).mp.
24 (slum or slums or ghetto or ghettos or informal settlement$ or shantytown$ or shanty town$).mp.
25 slum/
26 ghetto/
27 or/22-26

For peer review only

For peer review only - http://bmjopen.bmj.com/site/about/guidelines.xhtml

Annex 2: PRISMA Checklist

Section/topic	#	Checklist item	Reported on page #
**TITLE**			
Title	1	Identify the report as a systematic review, meta-analysis, or both.	1
**ABSTRACT**			
Structured summary	2	Provide a structured summary including, as applicable: background; objectives; data sources; study eligibility criteria, participants, and interventions; study appraisal and synthesis methods; results; limitations; conclusions and implications of key findings; systematic review registration number.	2
**INTRODUCTION**			
Rationale	3	Describe the rationale for the review in the context of what is already known.	4
Objectives	4	Provide an explicit statement of questions being addressed with reference to participants, interventions, comparisons, outcomes, and study design (PICOS).	5
**METHODS**			
Protocol and registration	5	Indicate if a review protocol exists, if and where it can be accessed (e.g., Web address), and, if available, provide registration information including registration number.	6
Eligibility criteria	6	Specify study characteristics (e.g., PICOS, length of follow-up) and report characteristics (e.g., years considered, language, publication status) used as criteria for eligibility, giving rationale.	6-7
Information sources	7	Describe all information sources (e.g., databases with dates of coverage, contact with study authors to identify additional studies) in the search and date last searched.	6
Search	8	Present full electronic search strategy for at least one database, including any limits used, such that it could be repeated.	6
Study selection	9	State the process for selecting studies (i.e., screening, eligibility, included in systematic review, and, if applicable, included in the meta-analysis).	7
Data collection process	10	Describe method of data extraction from reports (e.g., piloted forms, independently, in duplicate) and any processes for obtaining and confirming data from investigators.	7
Data items	11	List and define all variables for which data were sought (e.g., PICOS, funding sources) and any assumptions and simplifications made.	7
Risk of bias in individual studies	12	Describe methods used for assessing risk of bias of individual studies (including specification of whether this was done at the study or outcome level), and how this information is to be used in any data synthesis.	8
Summary measures	13	State the principal summary measures (e.g., risk ratio, difference in means).	9
Synthesis of results	14	Describe the methods of handling data and combining results of studies, if done, including measures of consistency (e.g., I^2) for each meta-analysis.	9
Risk of bias across studies	15	Specify any assessment of risk of bias that may affect the cumulative evidence (e.g., publication bias, selective reporting within studies).	8

For peer review only - http://bmjopen.bmj.com/site/about/guidelines.xhtml

Section/topic	#	Checklist item	Reported on page #
Additional analyses	16	Describe methods of additional analyses (e.g., sensitivity or subgroup analyses, meta-regression), if done, indicating which were pre-specified.	9
**RESULTS**			
Study selection	17	Give numbers of studies screened, assessed for eligibility, and included in the review, with reasons for exclusions at each stage, ideally with a flow diagram.	10
Study characteristics	18	For each study, present characteristics for which data were extracted (e.g., study size, PICOS, follow-up period) and provide the citations.	10
Risk of bias within studies	19	Present data on risk of bias of each study and, if available, any outcome level assessment (see item 12).	11
Results of individual studies	20	For all outcomes considered (benefits or harms), present, for each study: (a) simple summary data for each intervention group (b) effect estimates and confidence intervals, ideally with a forest plot.	11-12
Synthesis of results	21	Present results of each meta-analysis done, including confidence intervals and measures of consistency.	12-15
Risk of bias across studies	22	Present results of any assessment of risk of bias across studies (see Item 15).	11
Additional analysis	23	Give results of additional analyses, if done (e.g., sensitivity or subgroup analyses, meta-regression [see Item 16]).	12-15
**DISCUSSION**			
Summary of evidence	24	Summarize the main findings including the strength of evidence for each main outcome; consider their relevance to key groups (e.g., healthcare providers, users, and policy makers).	15-17
Limitations	25	Discuss limitations at study and outcome level (e.g., risk of bias), and at review-level (e.g., incomplete retrieval of identified research, reporting bias).	18-19
Conclusions	26	Provide a general interpretation of the results in the context of other evidence, and implications for future research.	18-19
**FUNDING**			
Funding	27	Describe sources of funding for the systematic review and other support (e.g., supply of data); role of funders for the systematic review.	20

For peer review only - http://bmjopen.bmj.com/site/about/guidelines.xhtml

BMJ Open

Global Prevalence and Trends in Hypertension and Type 2 Diabetes Mellitus among Slum Residents: A Systematic Review and Meta-analysis

Journal:	*BMJ Open*
Manuscript ID	bmjopen-2021-052393.R1
Article Type:	Original research
Date Submitted by the Author:	17-Nov-2021
Complete List of Authors:	Uthman, Olalekan; University of Warwick, Warwick Centre for Global Health, Warwick Medical School<br>Ayorinde, Abimbola; University of Warwick, Warwick Centre for Global Health, Warwick Medical School<br>Oyebode, Oyinlola; University of Warwick Warwick Medical School, Warwick Centre for Global Health, Warwick Medical School<br>Sartori, Jo; University of Birmingham, Institute of Applied Health Research<br>Gill, Paramjit ; University of Warwick, Warwick Centre for Global Health, Warwick Medical School,<br>Lilford, RJ; University of Birmingham, Institute of Applied Health Research
**Primary Subject Heading**:	Global health
Secondary Subject Heading:	Global health
Keywords:	Hypertension < CARDIOLOGY, DIABETES & ENDOCRINOLOGY, Public health < INFECTIOUS DISEASES

SCHOLARONE™
Manuscripts

I, the Submitting Author has the right to grant and does grant on behalf of all authors of the Work (as defined in the below author licence), an exclusive licence and/or a non-exclusive licence for contributions from authors who are: i) UK Crown employees; ii) where BMJ has agreed a CC-BY licence shall apply, and/or iii) in accordance with the terms applicable for US Federal Government officers or employees acting as part of their official duties; on a worldwide, perpetual, irrevocable, royalty-free basis to BMJ Publishing Group Ltd ("BMJ") its licensees and where the relevant Journal is co-owned by BMJ to the co-owners of the Journal, to publish the Work in this journal and any other BMJ products and to exploit all rights, as set out in our licence.

The Submitting Author accepts and understands that any supply made under these terms is made by BMJ to the Submitting Author unless you are acting as an employee on behalf of your employer or a postgraduate student of an affiliated institution which is paying any applicable article publishing charge ("APC") for Open Access articles. Where the Submitting Author wishes to make the Work available on an Open Access basis (and intends to pay the relevant APC), the terms of reuse of such Open Access shall be governed by a Creative Commons licence – details of these licences and which Creative Commons licence will apply to this Work are set out in our licence referred to above.

Other than as permitted in any relevant BMJ Author's Self Archiving Policies, I confirm this Work has not been accepted for publication elsewhere, is not being considered for publication elsewhere and does not duplicate material already published. I confirm all authors consent to publication of this Work and authorise the granting of this licence.

For peer review only

Global Prevalence and Trends in Hypertension and Type 2 Diabetes Mellitus among Slum Residents: A Systematic Review and Meta-analysis

For peer review only

Olalekan A. Uthman[1*], Ayorinde A. Abimbola[1], Oyinlola Oyebode[1], Jo Sartori[2], Paramjit Gill[1,3], RJ. Lilford[2]

[1]Warwick Centre for Global Health, Warwick Medical School, University of Warwick, Coventry, UK

[2]Institute of Applied Health Research, University of Birmingham, Birmingham, UK

[3]Academic Unit of Primary Care, Warwick Medical School, University of Warwick, Coventry, UK

***Correspondence author:**

Professor Olalekan A. Uthman, Warwick Centre for Global Health, Division of Health Sciences, Warwick Medical School, The University of Warwick, Coventry, CV4 7AL, United Kingdom. Email: olalekan.uthman@warwick.ac.uk

For peer review only - http://bmjopen.bmj.com/site/about/guidelines.xhtml

ABSTRACT

Objective: To obtain regional estimates of prevalence of hypertension and Type 2 diabetes in urban slums, and secondly to compare these with those in urban and rural areas.

Design: Systematic review and meta-analysis

Eligibility criteria: Studies that reported hypertension prevalence using the definition of blood pressure ≥140/90 mm Hg and/or prevalence of type 2 diabetes.

Information sources: Ovid MEDLINE, Cochrane CENTRAL and EMBASE from inception to December 2020

Risk of bias: Two authors extracted relevant data and assessed risk of bias independently using the Strengthening the Reporting of Observational Studies in Epidemiology (STROBE) guideline.

Synthesis of results: We used random-effects meta-analyses to pool prevalence estimates. We examined time trends in the prevalence estimates using meta-regression regression models with the prevalence estimates as the outcome variable and the calendar year of the publication as the predictor.

Results: A total 62 studies involving 108,110 participants met the inclusion criteria. Prevalence of hypertension and type 2 diabetes in slum populations ranged from 4.2% to 52.5% and 0.9% to 25.0%, respectively. In six studies presenting comparator data, all from the Indian sub-continent, slum residents were 35% more likely to be hypertensive than those living in comparator rural areas and 30% less likely to be hypertensive than those from comparator non-slum urban areas.

Limitations of evidence: Of the included studies, only few studies from India compared the slum prevalence estimates with those living on non-slum urban and rural areas, this limits the generalisability of the finding.

Interpretation: The burden of hypertension and type 2 diabetes varied widely between countries and regions and, to some degree, also within countries.

Funding: This research was funded by the National Institute for Health Research (NIHR) (16/136/87) using UK aid from the UK Government to support global health research.

PROSPERO registration number: CRD42017077381

For peer review only - http://bmjopen.bmj.com/site/about/guidelines.xhtml

Strengths and limitations of this study

- To reduce the chance of missing relevant studies, no language constraints were applied during the literature search.
- The data was extracted by two independent reviewers, reducing the possibility of bias.
- We analysed trends over time, and between geographical regions.
- The substantial between studies heterogeneity is an important limitation.
- Of the included studies, only few studies from India compared the slum prevalence estimates with those living on non-slum urban and rural areas, this limits the generalisability of the finding.

For peer review only

For peer review only - http://bmjopen.bmj.com/site/about/guidelines.xhtml

INTRODUCTION

Noncommunicable diseases (NCDs) are currently the leading cause of death globally, even in low- and middle-income countries (LMICs) the burden of disease is shifting from infectious diseases to NCDs[1]. NCDs now account for about 41 million deaths annually, corresponding to nearly 7 in 10 of all deaths worldwide. Every year, 15 million people of ages of 30 to 69 years die from these diseases, more than 85% of which are people living in LMICs. Most of the deaths from NCDs are caused by cardiovascular diseases, followed by cancer and respiratory diseases. NCDs affect people in all age groups, countries and geographic regions. The leading causes of these diseases include increased consumption of unhealthy foods, increased physical inactivity and population ageing[2-4]. These factors are mediated through metabolic risk factors for NCDs the most common of which include hypertension and type 2 diabetes[2-4]

Urbanization is a global phenomenon that is occurring at a fast pace in most LMICs[5 6]. For more than 20 years, urban settlements have been increasing in population size because of fast growth in urban births, significant movement of people from rural areas and sustained integration of the global economy [5 6]. The United Nations defines slums as urban areas with overcrowding, poor sanitation infrastructure, limited access to safe water, and/or poor structural quality of housing[7 8]. Slums are now an important component of today's urban settlements and likely continue to be for the foreseeable future [7 8].

For peer review only - http://bmjopen.bmj.com/site/about/guidelines.xhtml

Despite increased global awareness about the presence and persistence of slums, and evidence that their popualtions are affected by different health problems and needs to other urban inhabitants, the health of their inhabitants is under researched[7-10]. The health of the urban poor, people with low socioeconomic status living in urban areas, is usually conflated with that of slum residents. Although there is substantial overlap between these groups, there are also richer residents within slum neighbourhoods, as well as urban poverty occuring in non-slum urban areas. Health outcomes for these two groups may differ depending on whether deprivation is at the individual (urban poverty) or neighbourhood level (slum resident) due to neighbourhood effects [7 8 11 12]. For exampe, with respect to NCD risk-factors, those resident in slums, whatever their personal socio-economic status, may be more exposed to a common physical environmental risk factors (for example: air pollution increasing risk of hypertension), social environmental risk factors (for example: crime rates which may increase stress and drive metabolic risk) or institutional risk-factors (for example: stigma on the basis of their address reducing access to appropriate medical care). Many existing studies of NCDs risk factors done in urban areas do not disaggregate the population's health data by slum and non-slums status to allow for the detection of intra-urban health disparities that are due to neighbourhood effects rather than individual socio-economic status[13-22].

Understanding how the global challenges of hypertension, type 2 diabetes and rapid unplanned urbanisation intersect, by investigating whether the up to 1 billion people residing in slums[23]are succumbing to these important metabolic risk factors for non-communicable disease, will inform priorities for health services and health policy in LMICs. To fill this research gap, we therefore systematically gathered all the publications that relate to the burden of

For peer review only - http://bmjopen.bmj.com/site/about/guidelines.xhtml

110 hypertension among slum residents to (1) assess the contemporary prevalence esimates of hypertension among slum residents (2) compare the prevalence of hypertension and Type 2 diabetes in slums with those in two other types of settlement i.e. non-slum urban and rural areas; and (3) assess the proportion of those with hypertension who were aware of their hypertensive status, those on treatment and those with blood pressure under control.

For peer review only

For peer review only - http://bmjopen.bmj.com/site/about/guidelines.xhtml

METHODS

Protocol and registration

The study background, rationale, and methods were specified in advance and documented in a protocol that was published in the PROSPERO register (CRD42017077381).

Search and information sources:

We searched Ovid MEDLINE, Cochrane CENTRAL and EMBASE from inception to December 2020 using the following keywords: slum, shanty town, ghetto, hypertension and type 2 diabetes. The search strategy for Medline is shown in **Annex 1**.

Eligibility criteria:

We evaluated each identified study against the following pre-defined selection criteria:

- *Types of studies:* We included all studies (cross-sectional studies, retrospective or prospective cohort studies) that reported prevalence of hypertension and type 2 diabetes mellitus among slum residents as a primary or secondary outcome. No language, publication date or publication status restrictions were imposed.
- *Types of participants:* adult population (18 years and above) living in slum (as defined by the authors of the original studies included).
- *Types of Interventions:* Not applicable.
- *Types of outcomes:* Essential hypertension (also called primary or idiopathic hypertension), defined as persistent (seated) systolic blood pressure (SBP) of 140 mmHg or greater or had diastolic blood pressure 90mmHg or greater regardless of age and sex. We excluded studies that included subjects with pregnancy-induced, pre-

For peer review only - http://bmjopen.bmj.com/site/about/guidelines.xhtml

eclampsia, malignant, portal, pulmonary, renal, intracranial or ocular hypertension. We also excluded studies used only self-reported measure, i.e. deducible from the use of antihypertensive drugs or self-reported physician-diagnosed cases. If data were available, we noted (1) the percentage of those aware of their hypertension status (2) on any anti-hypertensive treatment, and (3) blood pressure controlled to a target level. Awareness of hypertension was defined as self-reporting of any prior diagnosis of hypertension by a healthcare professional. Treatment of hypertension was defined as receiving prescribed antihypertensive medication for management of high BP at some time in the 1 year preceding the survey. Control of hypertension was defined as the proportion of patients reporting antihypertensive therapy with SBP of less than 140 mmHg and DBP of less than 90 mmHg.

Type 2 diabetes was defined based on measured fasting plasma glucose, or oral glucose tolerance test. Type 2 diabetes was diagnosed if the fasting blood glucose was ≥126 mg/dL (≥7.0 mmol/L) after an overnight fast for at least 8 hours, or random capillary blood glucose of >= 11.1 mmol/L or if the participant was taking treatment for type 2 diabetes.

Study selection

In pairs, three reviewers (OAU, AAA, OO) independently evaluated the eligibility and methodological quality of the studies obtained from the literature searches. All articles yielded by the database search were initially screened by their titles and abstracts to obtain studies that met inclusion criteria. In cases of discrepancies, agreement was reached by discussion with a third reviewer. In pairs, three reviewers (OAU, AAA, OO) independently

For peer review only - http://bmjopen.bmj.com/site/about/guidelines.xhtml

then independently evaluated the full-text articles of all identified citations to establish relevance of the article according to the pre-specified criteria. In cases of discrepancies, agreement was reached by discussion with a third reviewer.

Data collection process and data items

OAU extracted data and AAA and OO checked the extracted data. For each study that met the selection criteria, details extracted included on year of publication, country of origin, study design, sample size, sampling strategy, study period, setting (rural/urban/slum), socio-demographic variables, prevalence estimates; etc.

Risk of bias (quality) assessment

The risk of bias of included studies will be assessed by using the Strengthening the Reporting of Observational Studies in Epidemiology (STROBE)[24 25](see Box 1). The risk of bias in a study was graded as low, high or unclear on the basis of study features including the selection of participants (selection bias), participation rate (selection bias), outcome measurement (detection bias), consideration of confounding variables (analytical methods to control for bias), and other form of bias.

For each included study, we estimated the precision (C) or margin of error, considering the sample size (SS) and the observed prevalence (p) of hypertension among slum dwellers from the formula:

$$SS = Z^2 * p * (1-p)/C^2 \qquad (1)$$

where Z was the z-value fixed at 1.96 across studies (corresponding to 95% confidence interval). The desirable margin of error is 5% (0.05) or lower.

For peer review only - http://bmjopen.bmj.com/site/about/guidelines.xhtml

Box 1: Risk of bias assessment

Bias type	Low-risk of bias	High-risk of bias	Unclear risk of bias
Selection (sample population)	participants selected randomly	Sample selection ambiguous and sample unlikely to be representative	Insufficient information
Selection (participation rate)	High participation rate (>70-85%)	Low participation rate (<70%)	Insufficient information
Performance bias (outcome assessment)	Objective measures of hypertension	Self-reported measure of hypertension	Insufficient information
Performance bias (analytical methods to control for bias)	Analysis appropriate for type of sample (unadjusted, univariable analyses etc.)	Analysis does not account for common adjustment (adjusted, multivariable analyses)	Insufficient information
Other form of bias	There is no evidence of bias from other sources.	There is potential bias present from other sources	Insufficient information

For peer review only

Synthesis of results

For the meta-analysis, we used DerSimonian-Laird random effects model[26] due to anticipated variations in study population, health care delivery systems and stage of epidemic transition to pool the hypertension and type 2 diabetes prevalence estimates. We performed leave-one-study-out sensitivity analysis to determine the stability of the results[27]. This analysis evaluated the influence of individual studies by estimating the pooled prevalence estimates in the absence of each study[27]. We assessed heterogeneity among studies by inspecting the forest plots and using the chi-squared test for heterogeneity with a 10% level of statistical significance and using the I^2 statistic where we interpret a value of 50% as representing moderate heterogeneity[28] [29]. We assessed the possibility of publication bias by evaluating a funnel plot for asymmetry. Because graphical evaluation can be subjective, we also conducted a Egger's regression asymmetry test as formal statistical tests for publication bias[30].

For peer review only - http://bmjopen.bmj.com/site/about/guidelines.xhtml

Following the overall analyses, we performed the following sub-group analyses: place of residence (rural versus urban slum versus non-slum urban); participants risk factors, including socioeconomic position; study design (cross-sectional, cohort); study location (low- and middle income versus high-income countries); and study precision.

We examined time trends in the prevalence estimates using meta-regression regression models with the prevalence estimates as the outcome variable and the calendar year of the publication as the predictor. In order to measure secular patterns in prevalence figures, we use the annual average percentages change (AAPC). We fitted a regression line to the natural logarithm of the prevalence estimates, i.e., $y = \alpha + \beta x + \varepsilon$, where y = ln(Prevalence), and x = calendar year. The AAPC was calculated as $100 \times (\exp(\beta)-1)$. The 95% confidence interval (CI) of the AAPC was also computed from the regression model. [31] The prevalence calculations indicated an upward trend when both the AAPC estimate and the lower limit of its 95% CI were > 0. However, they indicated a downward trend when both the AAPC and its upper limits were less than 0. The prevalence estimates were otherwise considered stable over time[31]. This systematic review was reported according to the Preferred Reporting Items for Systematic Reviews and Meta-analyses (PRISMA) guideline (**Annex 2**)[32].

Patient and public involvement

No patient was involved.

For peer review only - http://bmjopen.bmj.com/site/about/guidelines.xhtml

Results

Study selection and characteristics

The literature search yielded 1490 articles. **eFigure 1** shows the study selection flow diagram. After review, 135 articles were selected for critical reading. Seventy-two studies did not meet the inclusion criteria and were excluded (see **eTable 1** for list of excluded studies). The other 62 studies involving 108,110 participants met the inclusion criteria and were included in the meta-analysis[13-22 33-81]. Forty-three studies reported only hypertension prevalence estimates, 29 studies reported only type 2 diabetes prevalence estimates and eight reported both. **Table 1 and eTable 2** presents the characteristics of the included studies. The studies were reported between 1989 and 2019. Studies were reported as full-text journal articles (n=61, **98%);** except for one which was reported as a conference abstract. The number of participants included in the studies ranged from 100 to 15,763. When reported, the mean age of participants ranged from 32 years to 47 years. Most of the studies were carried out in South Asia: India (n=30); Bangladesh (n=8) and Nepal (n=1) and Pakistan (n=1); followed by sub-Saharan Africa: Kenya (n=9) and Nigeria (n=4); Latin America and Caribbean: Brazil (n=5) and Peru (n=1) and East Asia and Pacific: Thailand (n=1). Most of the studies were conducted in the following urban slums: Kibera (n=4), Delhi (n=3), Hyderabad (n=3), Ajegunle (n=2), Chandigarh (n=2), Chennai (n=2), Dhaka (n=2), Haryana (n=2), and Maceio (n=2).

Risk of bias of included studies

Summary of risk of bias assessment for each study is shown in **eTable 3.** The risk of bias in the selection of participants was low in most studies (n=58, 94%), high in three studies (5%) and unclear in one study. The risk of selection bias due to participate rate was low in most studies

For peer review only - http://bmjopen.bmj.com/site/about/guidelines.xhtml

(n=56, 90%), unclear in four (7%) and high in two study (3%). The performance bias due to outcome assessment was low in all the 62 studies as we included all studies that used objective measure of hypertension and type 2 diabetes. The performance bias due to analytical methods was low in 40 studies (64%) and high in 22 studies (35%). The risk of other biases was low in most studies (n=45, 73%), unclear in 16 studies (26%) and high in one study (2%).

For peer review only

Variations in prevalence of hypertension and type 2 diabetes by geographical regions

Prevalence of hypertension and type 2 diabetes from individuals are shown in **Figure 1 and Figure 2** respectively.

East Asia and Pacific

Thailand: One study from Klong-Toey slum found that 77 of the 976 respondents had type 2 diabetes in 1989 (7.9%, 95% CI 6.3 to 9.8).

Latin America and Caribbean

Brazil: Four studies reported the prevalence of hypertension from three different slums: Maceio (n=2), Rio de Janeiro (n=1) and Salvador (n=1). Florencio et al. found that almost one third of the Maceio slum dweller were hypertensive in 2004 (29.8%, 95% CI 24.8 to 35.2), while Ferriera et al estimated prevalence of hypertenssion among Maceio slum residents to be 14.8% (95% 10.4 to 20.2) in 2005. The reported prevalence of hypertension in other slums was 11.3% (95% CI10.2 to 12.4) in Rio de Janerio in 2007 and 20.6% (95% CI 19.5 to 21.7) in Salvador in 2015. The pooled prevalence (‘annualised year average’) of hypertension for the

For peer review only - http://bmjopen.bmj.com/site/about/guidelines.xhtml

four studies yielded an estimate of 18.4% (95% CI 12.0% to 26.2%). One study from Brazil found that one in ten had type 2 diabetes in 2017.

Peru: One study from a Lima slum conducted in 2014 found that 21 of the 142 respondents were hypertensive (14.8%, 95% CI 9.4 to 21.7).

South Asia

Bangladesh: Four studies from Dhakan slum reported prevalence of hypertension. The reported prevalence of hypertension ranged from 11.6% (95% CI 9.7 to 13.8) in 2012 to 19.56% (95% CI 17.85 to 21.37) in 2018. Fivestudies from Dhakan slum reported prevalence of type 2 diabetes. The pooled prevalence ('annualised year average') of hypertension for the three studies yielded an estimate of 16.1% (95% CI 12.2% to 20.3%). The reported prevalence of type 2 diabetes in these slums ranged from 8.1% (95% CI 6.8 to 9.6) in 2004 to 18.12% (95% CI 16.46 to 19.87) in 2019.

India: Twenty-two studies from India reported prevalence of hypertension from more than 15 difference slums. The reported prevalence varied across and within the slums. For example, Kar and colleagues estimated the prevalence of hypertension of 27.6% (95% 21.4 to 34.4) among 196 Chandigarh and Haryana slum residents in 2008; however they estimated the prevalence of hypertension of 16.5% (95% CI 15.1 to 18.0) among 2,562 196 Chandigarh and Haryana slum residents in 2010. Prevalence of type diabetes also varied across slums in India. The pooled prevalence ('annualised year average') of hypertension for the 22 studies yielded an estimate of 26.8% (95% CI 22.5% to 31.3%). In Delhi, the reported prevalence of type 2 diabetes ranged from 12.7% (95% CI 11.3 to 14.2) in 2007 to 31.5% (95% CI 27.8 to

For peer review only - http://bmjopen.bmj.com/site/about/guidelines.xhtml

35.4) in 2012. The pooled prevalence ('annualised year average') of type 2 for the 13studies yielded an estimate of 12.2% (95% CI 9.2% to 15.6%).

Nepal: One study from a Kathmandu slum conducted in 2013 found that 193 of the 689 respondents were hypertensive (28.0%, 95% CI 24.7 to 31.5).

Pakistan: One study from a Lahore slum found that 22 of the 695 respondents had type 2 diabetes in 2008 (3.2%, 95% CI 2.0 to 4.8).

Sub-Saharan Africa. *Kenya:* Six studies reported the prevalence of hypertension from three different slums: Kibera (n=4) and Viwandani and Korogocho (n=2). The reported prevalence among Kibera slum residents ranged from 13.0% (95% CI9.9 to 16.7) in 2013 to 27.8% (95% CI 25.9 to 29.7) in 2015. van de Vijver found that 640 of the 5,190 respondents from Viwandani and Korogocho slum residents were hypertensive (12.3%, 95% CI 11.5 to 13.3). The pooled prevalence ('annualised year average') of hypertension for the six studies yielded an estimate of 19.2% (95% CI 13.2% to 26.0%). The reported prevalence of type 2 diabetes ranged from 0.9% (95% CI 0.7 to 1.2 in Nairobi slum in 2016 to 4.4% (95% CI 3.8 to 5.0) in Viwandani and Korogocho in 2013. The pooled prevalence ('annualised year average') of type 2 diabetes for the six studies yielded an estimate of 4.5% (95% CI 2.0% to 7.9%).

Nigeria: Four studies from five different slums reported prevalence of hypertension. The reported prevalence varied across and within the slums. Ezeala-Adikaibe found that half of the respondents from Enugu slum were hypertensive in 2016 (52.5%, 95% CI 48.9 to 56.0). While Daniel et al. and Sowemimo et al. found that almost one-third of the Ajegule (38.2%,

For peer review only - http://bmjopen.bmj.com/site/about/guidelines.xhtml

95% CI 35.1 to 41.3, 2013) and Yemetu (33.1%, 95% CI 30.0 to 36.5, 2015) slum residents were hypertensive. However, Akinwale found that only 12.8% of the respondents from Ijora Oloye, Ajegunle and Makoko were hypertensive in 2013. The pooled prevalence ('annualised year average') of hypertension for the four studies yielded an estimate of 33.2% (95% CI 15.6% to 53.5%). Akinwale found that only 3.3% of the respondents from Ijora Oloye, Ajegunle and Makoko had type 2 diabetes in 2013.

Secular trends in hypertension and Type 2 diabetes prevalence estimates

Secular trends in hypertension, in 5 countries for which there were data across multiple time points, and type 2 diabetes, in 3 countries in which we had data across multiple time points, among slum residents are shown in **Figures 3 and 4**. We observed a continuous increase in prevalence of hypertension among slum residents in four out of five countries. The increase is more pronounced in India, followed by Kenya and Bangladesh. The prevalence of hypertension increased by 204.6% from 11.7% in 2001 to 35.5% in 2019 in India. The prevalence of hypertension increased by 98.8% from 12.3% in 2013 to 24.5% in 2019 in Kenya. However, the results of the trend analysis showed statistically significant upward trends only in India, such that the prevalence of hypertension increased +6.9% (95% CI +2.0% to +12.0%) per year between 2001 and 2019. There was no statistically significant trend was observed in Brazil using trend analyses (trend =-0.0%, 95% CI −22.7% to +29.2%). We also observed a continuous increase in prevalence of type 2 diabetes among slum residents in India and Bangladesh. The prevalence of type 2 diabetes increased by 123.6% from 8.1% in 2004 to 18.1% in 2019 in Bangladesh. The prevalence of type 2 diabetes increased by 95.8% from 10.3% in 2001 to 20.2% in 2019 in India. However, the results of the trend analysis showed statistically significant upward trends only in Bangladesh such that the prevalence of type 2

For peer review only - http://bmjopen.bmj.com/site/about/guidelines.xhtml

diabetes increased +5.9% (95% CI +1.1% to +10.8%) per year between 2004 and 2019. A non-statistically significant downward trends in type 2 diabetes prevalence was also observed in Kenya (trend =-11.1%, 95% CI −45.7% to +45.6%).

Prevalence of hypertension by different hypertension and type 2 diabetes subgroups

Study characteristics: As shown in **Table 1**, the pooled prevalence of hypertension was highest in studies conducted in lower-middle income countries (23.2%, 95% CI 21.5 to 29.0, 36 studies) than those from upper-middle income countries (17.9%, 95% CI 12.1 to 24.6, 5 studies). The pooled prevalence of hypertension tended to be higher among studies from South Asia (25.3%, 95% CI 21.3 to 29.6, 26 studies) and sub-Saharan Africa (24.4%, 95% CI 17.7 to 31.9, 10 studies) than those from Latin America and Carribean (18.3%, 95% CI 13.4 to 23.9, 6 studies). The pooled prevalence tended to higher among imprecise studies (33.4%, 95% CI 25.7 to 41.7, 8 studies) than those from precise studies (22.4%, 95% CI 18.9 to 26.1%, 35 studies). The pattern was similar for type 2 diabetes prevalence estimates.

Socio-demographic characteristics: As shown in **Table 1**, the pooled prevalence of hypertension was similar among males (22.5%, 95% CI 16.0 to 29.7, 24 studies) and females (23.5%, 95% CI 18.6 to 28.1, 24 studies). The pooled prevalence of hypertension tended to be higher among older adults (49.6%, 95% CI 36.7 to 62.6, 9 studies) than middle-age (35.0%, 95% CI 45.6, 9 studies) and young adults (15.7%, 95% CI 10.1 to 22.1, 8 studies). Similarly, the pooled prevalence of hypertension tended to be higher obese (45.4%, 95% CI 34.5 to 56.5, 6 studies) and overweight (32.9%, 95% CI 21.2 to 45.8, 6 studies) participants than participants with normal (21.9%, 95% CI 11.8 to 34.2, 6 studies) and under-weight (21.8%,

For peer review only - http://bmjopen.bmj.com/site/about/guidelines.xhtml

95% CI 11.4 to 34.4, 5 studies). The pooled prevalence of hypertension tended to be higher among those never studied (39.1%, 95% CI 27.5 to 51.3) than those with less than primary (18.3%, 95% CI 13.9 to 23.1, 4 studies), primary (24.8%, 95% CI 12.0 to 40.4, 6 studies) or secondary/higher education attainment (22.4%, 95% CI 11.2 to 36.2, 7 studies). The pooled prevalence of hypertension tended to be higher among least poor (29.2%, 95% CI 13.1 to 48.5, 5 studies) than those with middle- (25.3%, 10.6 to 43.8, 5 studies) and poorest-income (20.9%, 95% CI 10.4 to 33.8, 5 studies). The pattern was similar for type 2 diabetes prevalence estimates.

Lifestyle factors: The pooled prevalence of hypertension tended to be higher among smokers (38.0%, 95% CI 19.1 to 59.0, 5 studies) than those not smoking (30.5%, 95% CI 17.6 to 45.2, 5 studies). We found that the pooled prevalence of hypertension tended to be higher those not physically active (30.8%, 95% CI 7.7 to 60.9, 3 studies) than those physical active (28.8%, 95% CI 11.1 to 50.8); tended to be higher among with no history of alcohol consumption (29.1%, 95% CI 9.3 to 54.3, 3 studies) than those reported alcohol consumption (26.5%, 95% CI 18.0 to 35.9, 3 studies).

Comparative prevalence by place of residence

Six studies from India included non-slum populations alongside data from the slum population, and reported prevalence of hypertension by place of residence[37 39 47 49 50 52]. As shown in **Figure 5**, the pooled prevalence of hypertension was highest among those residing in non-slum urban areas (33.5%, 95% CI 26.0 to 42.0, 6 studies), followed by urban slum residents (28.8%, 95% CI 23.7 to 34.4%, 6 studies) and was lowest among rural residents (24.4%, 95% 18.4 to 31.5, 5 studies). Slum residents were 35% more likely to be hypertensive

For peer review only - http://bmjopen.bmj.com/site/about/guidelines.xhtml

than those living in rural areas (OR = 1.35, 95% 1.29 to 1.42) and 30% less likely to be hypertensive than those living in other urban areas (OR = 0.70, 95% CI 0.51 to 0.96).

Four studies from India (n=3) and Bangladesh reported prevalence of Type 2 diabetes by place of residence[47 52 60 72]. As shown in **Figure 6**, the pooled prevalence of type 2 diabetes was highest among those residing in non-slum urban areas (13.06%, 95% CI 6.53 to 24.43, 4 studies; 2813 participants), followed by urban slum residents (7.88%, 95% CI 3.32 to 17.55; 4 studies; 1811 participants) and was lowest among rural residents (1.64%; 95% CI 0.06 to 32.21; 3 studies; 405 participants). Such that prevalence of type 2 diabetes tended to be higher among urban slum residents than those living in rural areas (OR = 3.78, 95% 0.75 to 18.93). Urban slum residents were 46% less likely to be diabetic than those from other urban areas (OR = 0.54, 95% CI 0.44 to 0.66).

Treatment cascade

Among those diagnosed with hypertension, only one-third were aware of their hypertensive status (33.6%, 95% CI 19.1 to 50.0%, 12 studies) (**Table 1)**. Among those aware of their high blood pressure, half of them were on antihypertensive medications (51.9%, 95% CI 35.2 to 68.3, 9 studies). Among those on treatment, only one-quarter had good blood pressure control (25.2, 95% CI 18.4 to 34.3, 8 studies). Among those diagnosed with type 2 diabetes, 57.4% were aware of their type 2 diabetes status (95% CI 18.2 to 91.8%, 2 studies).

For peer review only - http://bmjopen.bmj.com/site/about/guidelines.xhtml

Discussion

Main Findings

This systematic review and meta-analysis summarises available evidence on the global prevalence of hypertension and type 2 diabetes among slum residents. There were several key findings: firstly, the burden of hypertension and type 2 diabetes among slum dweller is high and may be rising globally, with wide variation between countries and regions and, to some degree, also within countries. Using data from within study comparator populations when presented, the pooled prevalence of hypertension and Type 2 diabetes was highest among those residing in non-slum urban areas, followed by slum residents and was lowest among rural residents. This finding corroborates those of previous reviews that observed higher prevalence of hypertension among urban residents than those living in rural areas[82 83]. This high prevalence may be due to rapid urbanization, lifestyle changes, dietary changes and increased life expectancy[84 85] or a combination of these factors[86 87]. In addition, the observed difference could be due to other factors including but not limited to lack of access to testing and care of NCDs risk factors in rural areas and urban areas.

The observed gradient in burden of hypertension and Type 2 diabetes among rural, slum and urban residents is consistent with the effects of urbanization and wealth, as residents experience an economic transition when moving from one area to the next[88-93]. LMICs are now undergoing epidemiological transition, the change from a burden of infectious diseases to chronic diseases [94]. In addition, it could be due to increase in awareness in (non-slum) urban areas and recent availability of testing in some places. Recent systematic reviews of dietary risk-behaviour in Sub-Saharan Africa have found that urban populations tended to consume

For peer review only - http://bmjopen.bmj.com/site/about/guidelines.xhtml

more salt than rural populations [95] and consume fewer portions of vegetables[12]. The rapid pace of urbanisation and economic growth is accelerating the rate of this epidemiologic transition; as such LMICs are at great risk for an explosive growth in the burden of NCDs, including hypertension and type 2 diabetes [88 89].

We found evidence of significant unmet need for hypertension care among urban slum residents. Significant proportion of the urban slum residents were unscreened, undiagnosed, untreated or uncontrolled. This huge unmet need has been documented in previous studies from low- and middle-income settings[96-102]. We also found that control of hypertension among slum residents was poor, such that only one in four slum residents on treatment, had their blood pressure controlled. The poor control of BP noted in our study, despite the fact the one half of those that were unaware of high blood pressure being on antihypertensive medications, needs further exploration. One possible explanation is availability and affordability of the medications and there could be minimal additional contact with a health professional[15]. It has been documented that the control of BP was related to the frequency of follow-up visits[97]. Another possible explanation could be low adherence to prescribed medications, as they may not be able to afford the medications.

As expected, we found that the burden of hypertension increased with the particpants' age, which may be attributed to age-related structural changes in blood vessels which potentially cause narrowing of the vascular lumen, and consequently increasing blood pressure, as have been reported in previous studies[103 104]. The association between combined overweight/obesity and hypertension shown in our results exemplify the role of excess body weight in hypertension prevalence, which has been long recognized and consistent across numerous observational and trial data[105-107]. We found evidence of significantly high

For peer review only - http://bmjopen.bmj.com/site/about/guidelines.xhtml

prevalence of hypertension among smokers compared to the non-smokers. Direct relation of chronic tobacco consumption with hypertension however is not yet well established[108 109] although tobacco consumption has been shown to cause an acute elevation of BP[110].

Study Limitations and Strengths

To the best of our knowledge, this paper is the first systematic reviews that summarises data about prevalence of hypertension and type 2 diabetes among slum residents. Strengths of this study include the use of a predefined and published protocol, a comprehensive search strategy, and involvement of two independent reviewers in the review process. Nevertheless, the findings of this study should be interpreted with caution. Prevalence estimates from different regions and published over the course of 11 years were pooled in this meta-analysis, and as expected, high heterogeneity between studies was found in the meta-analyses. Nonetheless, as affirmed by previous evidence, meta-analyses are the preferred options to narrative syntheses for interpreting the results in a review, even in spite of the presence of a considerable amount of heterogeneity[111]. Heterogeneity appeared to be the norm rather than exception in published meta-analyses of observational studies[112].

In conclusion, the burden of hypertension and type 2 diabetes varied widely between countries and regions and, to some degree, also within countries. In addition, many hypertensive individuals are not aware of their condition, not on treatment and control of hypertension is poor. The burden of hypertension and type 2 diabetes was higher among urban residents than their counterparts living in urban slums and rural areas. There is a need for public health strategies to improve the awareness, control and overall management of hypertension and type 2 diabetes in urban areas.

For peer review only - http://bmjopen.bmj.com/site/about/guidelines.xhtml

Funding

This research was funded by the National Institute for Health Research (NIHR) Global Health Research Unit on Improving Health in Slums using UK aid from the UK Government to support global health research (Award ID: 16/136/87). The views expressed in this publication are those of the author(s) and not necessarily those of the NIHR or the UK Department of Health and Social Care.

Ethics approval and consent to participate

Not applicable.

Consent for publication: Not applicable.

Data sharing statement: No additional data available

Competing interests

The authors declare that they have no competing interests.

Authors' contribution

OAU, AAA, OO and RL conceived the study. OAU, AAA and OO collected and analysed initial data. OAU, AAA, OO, JO, PG and RL participated contributed in refining the data analysis. OAU wrote the first manuscript. OAU, AAA, OO, JS, PG and RL contributed to further analysis, interpreting and shaping of the argument of the manuscript and participated in writing the final draft of the manuscript. All the authors read and approved the final manuscript.

For peer review only - http://bmjopen.bmj.com/site/about/guidelines.xhtml

References

1. Global, regional, and national comparative risk assessment of 84 behavioural, environmental and occupational, and metabolic risks or clusters of risks, 1990-2016: a systematic analysis for the Global Burden of Disease Study 2016. *Lancet (London, England)* 2017;390(10100):1345-422. doi: 10.1016/s0140-6736(17)32366-8 [published Online First: 2017/09/19]

2. Bickler SW, Wang A, Amin S, et al. Urbanization in Sub-Saharan Africa: Declining Rates of Chronic and Recurrent Infection and Their Possible Role in the Origins of Non-communicable Diseases. *World journal of surgery* 2018;42(6):1617-28. doi: 10.1007/s00268-017-4389-5 [published Online First: 2017/12/14]

3. Goryakin Y, Rocco L, Suhrcke M. The contribution of urbanization to non-communicable diseases: Evidence from 173 countries from 1980 to 2008. *Economics and human biology* 2017;26:151-63. doi: 10.1016/j.ehb.2017.03.004 [published Online First: 2017/04/15]

4. Khorrami Z, Etemad K, Yarahmadi S, et al. Urbanization and noncommunicable disease (NCD) risk factors: WHO STEPwise Iranian NCD risk factors surveillance in 2011. *Eastern Mediterranean health journal = La revue de sante de la Mediterranee orientale = al-Majallah al-sihhiyah li-sharq al-mutawassit* 2017;23(7):469-79. [published Online First: 2017/08/31]

5. Cohen B. Urban Growth in Developing Countries: A Review of Current Trends and a Caution Regarding Existing Forecasts. *World Development* 2004;32(1):23-51. doi: https://doi.org/10.1016/j.worlddev.2003.04.008

6. Cohen B. Urbanization in developing countries: Current trends, future projections, and key challenges for sustainability. *Technology in Society* 2006;28(1):63-80. doi: https://doi.org/10.1016/j.techsoc.2005.10.005

7. Ezeh A, Oyebode O, Satterthwaite D, et al. The history, geography, and sociology of slums and the health problems of people who live in slums. *Lancet (London, England)* 2017;389(10068):547-58. doi: 10.1016/s0140-6736(16)31650-6 [published Online First: 2016/10/21]

8. Lilford RJ, Oyebode O, Satterthwaite D, et al. Improving the health and welfare of people who live in slums. *Lancet (London, England)* 2017;389(10068):559-70. doi: 10.1016/s0140-6736(16)31848-7 [published Online First: 2016/10/21]

9. Riley LW, Ko AI, Unger A, et al. Slum health: diseases of neglected populations. *BMC Int Health Hum Rights* 2007;7:2. doi: 10.1186/1472-698x-7-2 [published Online First: 2007/03/09]

10. Unger A, Riley LW. Slum health: from understanding to action. *PLoS medicine* 2007;4(10):1561-6. doi: 10.1371/journal.pmed.0040295 [published Online First: 2007/10/26]

11. Lilford R, Kyobutungi C, Ndugwa R, et al. Because space matters: conceptual framework to help distinguish slum from non-slum urban areas. *BMJ Glob Health* 2019;4(2):e001267. doi: 10.1136/bmjgh-2018-001267 [published Online First: 2019/05/30]

For peer review only - http://bmjopen.bmj.com/site/about/guidelines.xhtml

12. Mensah DO, Nunes AR, Bockarie T, et al. Meat, fruit, and vegetable consumption in sub-Saharan Africa: a systematic review and meta-regression analysis. *Nutr Rev* 2020 doi: 10.1093/nutrit/nuaa032 [published Online First: 2020/06/20]

13. Ahmad S, Goel K, Parashar P, et al. A community based cross sectional study on life style & morbidity status of elderly in urban slums of meerut. *Indian Journal of Public Health Research and Development* 2014;5(1):153-57.

14. Anand K, Shah B, Yadav K, et al. Are the urban poor vulnerable to non-communicable diseases? A survey of risk factors for non-communicable diseases in urban slums of Faridabad. *National Medical Journal of India* 2007;20(3):115-20.

15. Banerjee S, Mukherjee TK, Basu S. Prevalence, awareness, and control of hypertension in the slums of Kolkata. *Indian Heart Journal* 2016;68(3):286-94. doi: http://dx.doi.org/10.1016/j.ihj.2015.09.029

16. Daniel OJ, Adejumo OA, Adejumo EN, et al. Prevalence of hypertension among urban slum dwellers in Lagos, Nigeria. *Journal of urban health : bulletin of the New York Academy of Medicine* 2013;90(6):1016-25. doi: 10.1007/s11524-013-9795-x [published Online First: 2013/02/27]

17. Heitzinger K, Montano SM, Hawes SE, et al. A community-based cluster randomized survey of noncommunicable disease and risk factors in a peri-urban shantytown in Lima, Peru. *BMC International Health and Human Rights* 2014;14(1) doi: http://dx.doi.org/10.1186/1472-698X-14-19

18. Nirmala DB, Vijay KM, Sreedhar M. Prevalence of risk factors for Non Communicable Diseases in urban slums of Hyderabad, Telangana *Indian Journal of Basic and Applied Medical Research* 2014;4(1):487-93.

19. Oli N, Vaidya A, Thapa G. Behavioural risk factors of noncommunicable diseases among nepalese urban poor: A descriptive study from a slum area of Kathmandu. *Epidemiology Research International* 2013(pagination) doi: http://dx.doi.org/10.1155/2013/329156

20. Rawal LB, Biswas T, Khandker NN, et al. Non-communicable disease (NCD) risk factors and diabetes among adults living in slum areas of Dhaka, Bangladesh. *PLoS ONE* 2017;12(10) doi: http://dx.doi.org/10.1371/journal.pone.0184967

21. Singh R, Mukherjee M, Kumar R, et al. Study of Risk factors of Coronary Heart Disease in Urban Slums of Patna. *2012* 2012;2(3):-192. doi: 10.3126/nje.v2i3.6902 [published Online First: 2012-10-02]

22. Vigneswari A, Manikandan R, Satyavani K, et al. Prevalence of Risk Factors of Diabetes Among Urban Poor South Indian Population. *The Journal of the Association of Physicians of India* 2015;63(10):32-4. [published Online First: 2016/09/10]

23. UN-Habitat. UN-Habitat Urbanization and development: emerging futures. world cities report 2016. Nairobi Kenya, 2016.

24. Chen G, Lin L, Yan-Lin Y, et al. The prevalence and incidence of community-acquired pressure injury: A protocol for systematic review and meta-analysis. *Medicine*

For peer review only - http://bmjopen.bmj.com/site/about/guidelines.xhtml

2020;99(48):e22348. doi: 10.1097/md.0000000000022348 [published Online First: 2020/11/26]

25. Vandenbroucke JP, von Elm E, Altman DG, et al. Strengthening the Reporting of Observational Studies in Epidemiology (STROBE): explanation and elaboration. *Epidemiology* 2007;18(6):805-35. doi: 10.1097/EDE.0b013e3181577511 [published Online First: 2007/12/01]

26. DerSimonian R, Laird N. Meta-analysis in clinical trials. *Controlled clinical trials* 1986;7(3):177-88. [published Online First: 1986/09/01]

27. Normand SL. Meta-analysis: formulating, evaluating, combining, and reporting. *Statistics in medicine* 1999;18(3):321-59. [published Online First: 1999/03/10]

28. Higgins JP, Thompson SG. Quantifying heterogeneity in a meta-analysis. *Statistics in medicine* 2002;21(11):1539-58. doi: 10.1002/sim.1186 [published Online First: 2002/07/12]

29. Higgins JP, Thompson SG, Deeks JJ, et al. Measuring inconsistency in meta-analyses. *BMJ (Clinical research ed)* 2003;327(7414):557-60. doi: 10.1136/bmj.327.7414.557 [published Online First: 2003/09/06]

30. Egger M, Davey Smith G, Schneider M, et al. Bias in meta-analysis detected by a simple, graphical test. *BMJ (Clinical research ed)* 1997;315(7109):629-34. [published Online First: 1997/10/06]

31. Clegg LX, Hankey BF, Tiwari R, et al. Estimating average annual per cent change in trend analysis. *Statistics in medicine* 2009;28(29):3670-82. doi: 10.1002/sim.3733 [published Online First: 2009/10/27]

32. Liberati A, Altman DG, Tetzlaff J, et al. The PRISMA statement for reporting systematic reviews and meta-analyses of studies that evaluate healthcare interventions: explanation and elaboration. *BMJ (Clinical research ed)* 2009;339:b2700. doi: 10.1136/bmj.b2700 [published Online First: 2009/07/23]

33. Acharyya T, Kaur P, Murhekar MV. Prevalence of behavioral risk factors, overweight and hypertension in the urban slums of North 24 Parganas District, West Bengal, India, 2010. *Indian journal of public health* 2014;58(3):195-98.

34. Akinwale O, Oyefara J, Adejoh P, et al. The benefits of using a community-engaged research approach to promote a healthy lifestyle in three Nigerian urban slums. *Southern African Journal of Epidemiology and Infection* 2014;29(1):48-50.

35. Ayah R, Joshi MD, Wanjiru R, et al. A population-based survey of prevalence of diabetes and correlates in an urban slum community in Nairobi, Kenya. *BMC public health* 2013;13(371):20. doi: https://dx.doi.org/10.1186/1471-2458-13-371

36. Chakraborty R, Bose K. Comparison of body adiposity indices in predicting blood pressure and hypertension among slum-dwelling men in Kolkata, India. *Malaysian Journal of Nutrition* 2012;18(3):319-28.

For peer review only

For peer review only - http://bmjopen.bmj.com/site/about/guidelines.xhtml

37. Chaturvedi S, Pant M, Yadav G. Hypertension in Delhi: prevalence, awareness, treatment and control. *Tropical doctor* 2007;37(3):142-5. doi: 10.1258/004947507781524593 [published Online First: 2007/08/25]

38. Dasappa H, Fathima FN, Prabhakar R, et al. Prevalence of diabetes and pre-diabetes and assessments of their risk factors in urban slums of Bangalore. *Journal of family medicine and primary care* 2015;4(3):399-404. doi: 10.4103/2249-4863.161336 [published Online First: 2015/08/20]

39. Deepa M, Pradeepa R, Anjana R, et al. Noncommunicable diseases risk factor surveillance: experience and challenge from India. *Indian journal of community medicine : official publication of Indian Association of Preventive & Social Medicine* 2011;36(Suppl 1):S50-6. doi: 10.4103/0970-0218.94709 [published Online First: 2012/05/26]

40. Edwards JK, Bygrave H, Van den Bergh R, et al. HIV with non-communicable diseases in primary care in Kibera, Nairobi, Kenya: characteristics and outcomes 2010-2013. *Trans R Soc Trop Med Hyg* 2015;109(7):440-6. doi: https://dx.doi.org/10.1093/trstmh/trv038

41. Ezeala-Adikaibe BA, Orjioke C, Ekenze OS, et al. Population-based prevalence of high blood pressure among adults in an urban slum in Enugu, South East Nigeria. *Journal of Human Hypertension* 2016;30(4):285-91. doi: http://dx.doi.org/10.1038/jhh.2015.49

42. Ferreira HDS, Florencio TMTDM, Fragoso MDAC, et al. Hypertension, abdominal obesity and short stature: Aspects of nutritional transition within a shantytown in the city of Maceio (Northeastern Brazil). *Revista de Nutricao* 2005;18(2):209-18.

43. Florencio TT, Ferreira HS, Cavalcante JC, et al. Short stature, obesity and arterial hypertension in a very low income population in North-eastern Brazil. *Nutrition, Metabolism and Cardiovascular Diseases* 2004;14(1):26-33. doi: http://dx.doi.org/10.1016/S0939-4753%2804%2980044-9

44. Haregu TN, Oti S, Ngomi N, et al. Interlinkage among cardio-metabolic disease markers in an urban poor setting in Nairobi, Kenya. *Global health action* 2016;9(pp 30626) doi: http://dx.doi.org/10.3402/gha.v9.30626

45. Huda MN, Alam KS, Harun Ur R. Prevalence of chronic kidney disease and its association with risk factors in disadvantageous population. *International journal of nephrology* 2012;2012:267329. doi: 10.1155/2012/267329 [published Online First: 2012/08/01]

46. Jalil F, Moore SE, Butt NS, et al. Early-life risk factors for adult chronic disease: Follow-up of a cohort born during 1964-1978 in an urban slum of Lahore, Pakistan. *Journal of Health, Population and Nutrition* 2008;26(1):12-21.

47. Joshi A, Puricelli Perin DM, Arora M. Using Portable Health Information Kiosk to assess chronic disease burden in remote settings. *Rural and remote health* 2013;13(2):2279. [published Online First: 2013/03/29]

48. Joshi MD, Ayah R, Njau EK, et al. Prevalence of hypertension and associated cardiovascular risk factors in an urban slum in Nairobi, Kenya: a population-based survey. *BMC public health* 2014;14:1177. doi: 10.1186/1471-2458-14-1177 [published Online First: 2014/11/20]

For peer review only - http://bmjopen.bmj.com/site/about/guidelines.xhtml

49. Kar SS, Thakur JS, Jain S, et al. Cardiovascular disease risk management in a primary health care setting of North India. *Indian Heart Journal* 2008;60(1):19-25.

50. Kar SS, Thakur JS, Virdi NK, et al. Risk factors for cardiovascular diseases: Is the social gradient reversing in northern India? *National Medical Journal of India* 2010;23(4):206-09.

51. Kumari SMV, Humaira B, Sreedhar M. A study on prevalence of hypertension in urban slum field practice area of osmania medical college – Hyderabad *Indian Journal of Basic and Applied Medical Research* 2014;4(1):462-70.

52. Lubree HG, Rege SS, Bhat DS, et al. Body fat and cardiovascular risk factors in Indian men in three geographical locations. *Food and Nutrition Bulletin* 2002;23(3 SUPP):146-49.

53. Marins VM, Almeida RM, Pereira RA, et al. The association between socioeconomic indicators and cardiovascular disease risk factors in Rio de Janeiro, Brazil. *J Biosoc Sci* 2007;39(2):221-9. doi: https://dx.doi.org/10.1017/S0021932006001246

54. Misra A, Pandey RM, Devi JR, et al. High prevalence of diabetes, obesity and dyslipidaemia in urban slum population in northern India. *International journal of obesity and related metabolic disorders : journal of the International Association for the Study of Obesity* 2001;25(11):1722-9. doi: 10.1038/sj.ijo.0801748 [published Online First: 2001/12/26]

55. Olack B, Wabwire-Mangen F, Smeeth L, et al. Risk factors of hypertension among adults aged 35-64 years living in an urban slum Nairobi, Kenya. *BMC public health* 2015;15:1251. doi: 10.1186/s12889-015-2610-8 [published Online First: 2015/12/19]

56. Ongeti K, Ogeng'o J, Pulei A, et al. Blood pressure characteristics among slum dwellers in Kenya. *Global Advanced Research* 2013;2(4):80-85.

57. Oti SO, van de Vijver SJ, Agyemang C, et al. The magnitude of diabetes and its association with obesity in the slums of Nairobi, Kenya: results from a cross-sectional survey. *Tropical medicine & international health : TM & IH* 2013;18(12):1520-30. doi: 10.1111/tmi.12200 [published Online First: 2013/10/15]

58. Patil RS, Gothankar JS. Assessment of risk of type 2 diabetes using the Indian Diabetes Risk Score in an urban slum of Pune, Maharashtra, India: a cross-sectional study. *WHO South-East Asia journal of public health* 2016;5(1):53-61. doi: 10.4103/2224-3151.206555 [published Online First: 2016/04/01]

59. Rahim MA, Vaaler S, Keramat Ali SM, et al. Prevalence of type 2 diabetes in urban slums of Dhaka, Bangladesh. *Bangladesh Medical Research Council Bulletin* 2004;30(2):60-70.

60. Sayeed MA, Mahtab H, Khanam PA, et al. Prevalence of diabetes and impaired fasting glucose in urban population of Bangladesh. *Bangladesh Medical Research Council Bulletin* 2007;33(1):1-12.

61. Singh AK, Mani K, Krishnan A, et al. Prevalence, awareness, treatment and control of diabetes among elderly persons in an urban slum of delhi. *Indian journal of community medicine : official publication of Indian Association of Preventive & Social Medicine* 2012;37(4):236-9. doi: 10.4103/0970-0218.103472 [published Online First: 2013/01/08]

For peer review only - http://bmjopen.bmj.com/site/about/guidelines.xhtml

62. Sinha P, Taneja DK, Singh NP, et al. Seasonal variation in prevalence of hypertension: Implications for interpretation. *Indian journal of public health* 2010;54(1):7-10.

63. Sitthi-Amorn C, Chandraprasert S, Bunnag SC, et al. The prevalence and risk factors of hypertension in Klong Toey Slum and Klong Toey government apartment houses. *International Journal of Epidemiology* 1989;18(1):89-94.

64. Snyder RE, Lopes LA, Tavares LCC, et al. O Dia de Dona Maria-Using technology and community based participatory research to improve healthcare delivery in a Brazilian urban slum. *Annals of Global Health* 2016;Conference:7th Annual CUGH Conference: Bridging to a Sustainable Future in Global Health. United States. 82 (3) (pp 599).

65. Sowemimo I, Ajayi I, Akpa O, et al. Prevalence of hypertension and associated factors among residents of Ibadan-north local government area of Oyo State, Nigeria. *Journal of Hypertension* 2015;Conference:25th European Meeting on Hypertension and Cardiovascular Protection. doi: http://dx.doi.org/10.1097/01.hjh.0000467432.10548.8c

66. Sunita M, Singh AK, Rogye A, et al. Prevalence of Diabetic Retinopathy in Urban Slums: The Aditya Jyot Diabetic Retinopathy in Urban Mumbai Slums Study-Report 2. *Ophthalmic Epidemiology* 2017;24(5):303-10. doi: http://dx.doi.org/10.1080/09286586.2017.1290258

67. Unger A, Felzemburgh RD, Snyder RE, et al. Hypertension in a Brazilian urban slum population. *J Urban Health* 2015;92(3):446-59. doi: https://dx.doi.org/10.1007/s11524-015-9956-1

68. Uthakalla VK, Kishore Kumar KJ, Jena SK, et al. Prevalence study of overweight/obesity among adults (20-60yrs) of urban field practice area of osmania medical college, Hyderabad. *Indian Journal of Public Health Research and Development* 2012;3(3):250-53.

69. van de Vijver S, Oti S, Tervaert TC, et al. Introducing a model of cardiovascular prevention in Nairobi's slums by integrating a public health and private-sector approach: the SCALE-UP study. *Global health action* 2013;6(pp 22510)

70. Vikram NK, Pandey RM, Misra A, et al. Non-obese (body mass index < 25 kg/m²) Asian Indians with normal waist circumference have high cardiovascular risk. *Nutrition* 2003;19(6):503-09. doi: http://dx.doi.org/10.1016/S0899-9007%2802%2901083-3

71. Wasir JS, Misra A, Vikram NK, et al. C-reactive protein, obesity, and insulin resistance in postmenopausal women in urban slums of North India. *Diabetes and Metabolic Syndrome: Clinical Research and Reviews* 2007;1(2):83-89. doi: http://dx.doi.org/10.1016/j.dsx.2007.02.001

72. Yajnik CS, Joglekar CV, Lubree HG, et al. Adiposity, inflammation and hyperglycaemia in rural and urban Indian men: Coronary Risk of Insulin Sensitivity in Indian Subjects (CRISIS) Study. *Diabetologia* 2008;51(1):39-46. doi: https://dx.doi.org/10.1007/s00125-007-0847-1

73. Hypertension detection, treatment and control rates in urban slum population in bangladesh. *Journal of Hypertension* 2018;Conference:27th Scientific Meeting of the International Society of Hypertension, ISH 2018. China. 36 (Supplement 3) (pp e337-e338).

For peer review only

For peer review only - http://bmjopen.bmj.com/site/about/guidelines.xhtml

74. Assessment of risk factors of hypertension among adults residing in urban slum of Delhi. *Asian Journal of Pharmaceutical and Clinical Research* 2018;11(1):405-07. doi: http://dx.doi.org/10.22159/ajpcr.2018.v11i1.23755

75. Prevalence of hypertension among elderly residing in slums of west Delhi. *Asian Journal of Pharmaceutical and Clinical Research* 2018;11(4):337-39. doi: http://dx.doi.org/10.22159/ajpcr.2018.v11i4.23414

76. Abhinav Jain BKAMPSMSKSACSJ. A Study of Prevalence of Diabetes Mellitus and its Risk Factors in the Urban Slum Population of Gurugram. *Indian Journal of Public Health Research & Development* 2019;10(4):141-45. doi: 10.37506/ijphrd.v10i4.6613

77. Bawah AT, Abaka-Yawson A, Seini MM, et al. Prevalence of diabetes among homeless and slum dwellers in Accra, Ghana: a survey study. *BMC Res Notes* 2019;12(1):572. doi: 10.1186/s13104-019-4613-5 [published Online First: 2019/09/13]

78. Gadallah M, Megid SA, Mohsen A, et al. Hypertension and associated cardiovascular risk factors among urban slum dwellers in Egypt: a population-based survey. *Eastern Mediterranean health journal = La revue de sante de la Mediterranee orientale = al-Majallah al-sihhiyah li-sharq al-mutawassit* 2018;24(5):435-42. doi: 10.26719/2018.24.5.435 [published Online First: 2018/07/26]

79. George CE, Norman G, Wadugodapitya A, et al. Health issues in a Bangalore slum: findings from a household survey using a mobile screening toolkit in Devarajeevanahalli. *BMC public health* 2019;19(1):456. doi: 10.1186/s12889-019-6756-7 [published Online First: 2019/05/01]

80. Tymejczyk O, McNairy ML, Petion JS, et al. Hypertension prevalence and risk factors among residents of four slum communities: population-representative findings from Port-au-Prince, Haiti. *Journal of hypertension* 2019;37(4):685-95. doi: 10.1097/hjh.0000000000001966 [published Online First: 2019/03/01]

81. Vusirikala A, Wekesah F, Kyobutungi C, et al. Assessment of cardiovascular risk in a slum population in Kenya: use of World Health Organisation/International Society of Hypertension (WHO/ISH) risk prediction charts - secondary analyses of a household survey. *BMJ open* 2019;9(9):e029304. doi: 10.1136/bmjopen-2019-029304 [published Online First: 2019/09/07]

82. Addo J, Smeeth L, Leon DA. Hypertension in sub-saharan Africa: a systematic review. *Hypertension (Dallas, Tex : 1979)* 2007;50(6):1012-8. doi: 10.1161/hypertensionaha.107.093336 [published Online First: 2007/10/24]

83. Pereira M, Lunet N, Azevedo A, et al. Differences in prevalence, awareness, treatment and control of hypertension between developing and developed countries. *Journal of hypertension* 2009;27(5):963-75. [published Online First: 2009/04/30]

84. Gupta R, al-Odat NA, Gupta VP. Hypertension epidemiology in India: meta-analysis of 50 year prevalence rates and blood pressure trends. *J Hum Hypertens* 1996;10(7):465-72. [published Online First: 1996/07/01]

85. Mahmood SE, Prakash D, Srivastava JP, et al. Prevalence of Hypertension Amongst Adult Patients Attending Out Patient Department of Urban Health Training Centre,

For peer review only - http://bmjopen.bmj.com/site/about/guidelines.xhtml

Department of Community Medicine, Era's Lucknow Medical College and Hospital, Lucknow. *Journal of clinical and diagnostic research : JCDR* 2013;7(4):652-6. doi: 10.7860/jcdr/2013/4707.2874 [published Online First: 2013/06/05]

86. Amuna P, Zotor FB. Epidemiological and nutrition transition in developing countries: impact on human health and development. *The Proceedings of the Nutrition Society* 2008;67(1):82-90. doi: 10.1017/s0029665108006058 [published Online First: 2008/02/01]

87. Kroll M, Bharucha E, Kraas F. Does rapid urbanization aggravate health disparities? Reflections on the epidemiological transition in Pune, India. *Glob Health Action* 2014;7:23447. doi: 10.3402/gha.v7.23447 [published Online First: 2014/09/13]

88. Angkurawaranon C, Jiraporncharoen W, Chenthanakij B, et al. Urbanization and non-communicable disease in Southeast Asia: a review of current evidence. *Public health* 2014;128(10):886-95. doi: 10.1016/j.puhe.2014.08.003 [published Online First: 2014/11/05]

89. Cheema A, Adeloye D, Sidhu S, et al. Urbanization and prevalence of type 2 diabetes in Southern Asia: A systematic analysis. *Journal of global health* 2014;4(1):010404. doi: 10.7189/jogh.04.010404 [published Online First: 2014/07/01]

90. Low WY, Lee YK, Samy AL. Non-communicable diseases in the Asia-Pacific region: Prevalence, risk factors and community-based prevention. *International journal of occupational medicine and environmental health* 2015;28(1):20-6. doi: 10.2478/s13382-014-0326-0 [published Online First: 2015/07/15]

91. Phipps ME, Chan KK, Naidu R, et al. Cardio-metabolic health risks in indigenous populations of Southeast Asia and the influence of urbanization. *BMC public health* 2015;15:47. doi: 10.1186/s12889-015-1384-3 [published Online First: 2015/02/01]

92. Siegel KR, Patel SA, Ali MK. Non-communicable diseases in South Asia: contemporary perspectives. *British medical bulletin* 2014;111(1):31-44. doi: 10.1093/bmb/ldu018 [published Online First: 2014/09/06]

93. Streatfield PK, Khan WA, Bhuiya A, et al. Adult non-communicable disease mortality in Africa and Asia: evidence from INDEPTH Health and Demographic Surveillance System sites. *Glob Health Action* 2014;7:25365. doi: 10.3402/gha.v7.25365 [published Online First: 2014/11/08]

94. Gaziano TA, Bitton A, Anand S, et al. Growing epidemic of coronary heart disease in low- and middle-income countries. *Current problems in cardiology* 2010;35(2):72-115. doi: 10.1016/j.cpcardiol.2009.10.002 [published Online First: 2010/01/30]

95. Oyebode O, Oti S, Chen YF, et al. Salt intakes in sub-Saharan Africa: a systematic review and meta-regression. *Population health metrics* 2016;14:1. doi: 10.1186/s12963-015-0068-7 [published Online First: 2016/01/14]

96. Adeloye D, Basquill C. Estimating the prevalence and awareness rates of hypertension in Africa: a systematic analysis. *PLoS One* 2014;9(8):e104300. doi: 10.1371/journal.pone.0104300 [published Online First: 2014/08/05]

For peer review only - http://bmjopen.bmj.com/site/about/guidelines.xhtml

97. Macia E, Duboz P, Gueye L. Prevalence, awareness, treatment and control of hypertension among adults 50 years and older in Dakar, Senegal. *Cardiovascular journal of Africa* 2012;23(5):265-9. doi: 10.5830/cvja-2011-039 [published Online First: 2011/10/18]

98. Mohan V, Deepa M, Farooq S, et al. Prevalence, awareness and control of hypertension in Chennai--The Chennai Urban Rural Epidemiology Study (CURES-52). *The Journal of the Association of Physicians of India* 2007;55:326-32. [published Online First: 2007/09/12]

99. Pilav A, Doder V, Brankovic S. Awareness, Treatment, and control of Hypertension among Adult Population in the Federation of Bosnia and Herzegovina over the Past Decade. *Journal of public health research* 2014;3(3):323. doi: 10.4081/jphr.2014.323 [published Online First: 2015/01/02]

100. Supiyev A, Kossumov A, Utepova L, et al. Prevalence, awareness, treatment and control of arterial hypertension in Astana, Kazakhstan. A cross-sectional study. *Public health* 2015;129(7):948-53. doi: 10.1016/j.puhe.2015.02.020 [published Online First: 2015/03/31]

101. Tailakh A, Evangelista LS, Mentes JC, et al. Hypertension prevalence, awareness, and control in Arab countries: a systematic review. *Nursing & health sciences* 2014;16(1):126-30. doi: 10.1111/nhs.12060 [published Online First: 2013/10/15]

102. Yazdanpanah L, Shahbazian H, Shahbazian H, et al. Prevalence, awareness and risk factors of hypertension in southwest of Iran. *Journal of renal injury prevention* 2015;4(2):51-6. doi: 10.12861/jrip.2015.11 [published Online First: 2015/06/11]

103. Landahl S, Bengtsson C, Sigurdsson JA, et al. Age-related changes in blood pressure. *Hypertension (Dallas, Tex : 1979)* 1986;8(11):1044-9. [published Online First: 1986/11/01]

104. Pinto E. Blood pressure and ageing. *Postgraduate medical journal* 2007;83(976):109-14. doi: 10.1136/pgmj.2006.048371 [published Online First: 2007/02/20]

105. Dyer AR, Elliott P, Shipley M. Body mass index versus height and weight in relation to blood pressure. Findings for the 10,079 persons in the INTERSALT Study. *American journal of epidemiology* 1990;131(4):589-96. [published Online First: 1990/04/01]

106. Folsom AR, Kushi LH, Anderson KE, et al. Associations of general and abdominal obesity with multiple health outcomes in older women: the Iowa Women's Health Study. *Archives of internal medicine* 2000;160(14):2117-28. [published Online First: 2000/07/25]

107. Hu G, Barengo NC, Tuomilehto J, et al. Relationship of physical activity and body mass index to the risk of hypertension: a prospective study in Finland. *Hypertension (Dallas, Tex : 1979)* 2004;43(1):25-30. doi: 10.1161/01.Hyp.0000107400.72456.19 [published Online First: 2003/12/06]

108. Abtahi F, Kianpour Z, Zibaeenezhad MJ. Correlation between cigarette smoking and blood pressure and pulse pressure among teachers residing in Shiraz, Southern Iran. *Iran Cardiovasc Res J* 2011;5:97-102.

109. Primatesta P, Falaschetti E, Gupta S, et al. Association between smoking and blood pressure: evidence from the health survey for England. *Hypertension (Dallas, Tex : 1979)* 2001;37(2):187-93. [published Online First: 2001/03/07]

For peer review only - http://bmjopen.bmj.com/site/about/guidelines.xhtml

110. Westman EC. Does smokeless tobacco cause hypertension? *Southern medical journal* 1995;88(7):716-20. [published Online First: 1995/07/01]

111. Ioannidis JP, Patsopoulos NA, Rothstein HR. Reasons or excuses for avoiding meta-analysis in forest plots. *BMJ (Clinical research ed)* 2008;336(7658):1413-5. doi: 10.1136/bmj.a117 [published Online First: 2008/06/21]

112. Higgins JP. Commentary: Heterogeneity in meta-analysis should be expected and appropriately quantified. *Int J Epidemiol* 2008;37(5):1158-60. doi: 10.1093/ije/dyn204 [published Online First: 2008/10/04]

For peer review only

For peer review only - http://bmjopen.bmj.com/site/about/guidelines.xhtml

TABLES

Table 1: Pooled prevalence by difference subgroup

Subgroup		Hypertension			Type 2 Diabetes		
		n	%	I^2	n	%	I^2
Sample size	Smaller studies (<1000)	27	25.9 (21.6 to 30.6)	97.1	15	11.0 (8.2 to 14.2)	93.9
Sample size	Larger studies (1000+)	17	21.4 (17.2 ro 26.1)	99.6	15	7.8 (5.1 to 11.1)	99.4
Study precision	Imprecise studies	8	33.4 (25.7 to 41.7)	91.2	1	25.2 (17.3 to 34.2)	-
Study precision	Precise studies	36	22.3 (18.9 to 25.9)	99.2	29	8.9 (6.9 to 11.2)	98.9
Publication year	2001 to 2005	5	15.6 (9.0 to 23.8)	94.7	4	8.2 (6.7 to 9.8)	53.6
Publication year	2006 to 2010	6	28.6 (18.9 to 39.4)	98.7	4	6.3 (3.3 to 10.3)	90.6
Publication year	2011 to 2020	33	24.7 (21.0 to 28.6)	99.2	22	10.2 (7.4 to 13.4)	99.2
Region	South Asia	27	25.1 (20.7 to 29.8)	98.9	19	11.9 (9.1 to 15.1)	97.6
Region	Sub-Saharan Africa	10	24.4 (17.7 to 31.9)	99.2	8	4.5 (2.4 to 7.2)	98.8
Region	Latin America and Caribbean	6	18.3 (13.4 to 23.9)	97.1	1	10.2 (8.1 to 12.3)	-
Region	Middle East and North Africa	1	31.2 (28.4 to 34.1)	-	1	8.8 (7.1 to 10.6)	-
Region	East Asia and Pacific	-	-	-	1	7.9 (6.3 to 9.7)	
Income category	Lower Middle Income	36	25.2 (21.2 to 29.4)	99.1	28	9.3 (7.0 to 11.92)	98.9
Income category	Upper Middle Income	5	17.9 (12.1 to 24.6)	97.6	2	9.0 (6.9 to 11.3)	62
Income category	Low Income	2	24.0 (16.9 to 32.0)	92.2			
Sex	Male	24	22.5 (16.0 to 29.7)	99.2	11	8.1 (5.1 to 11.6)	97.6
Sex	Female	24	23.2 (18.6 to 28.1)	98.7	11	7.3 (4.6 to 10.6)	97.5
Age	Young adult	8	15.7 (10.1 to 22.1)	97.8	2	2.1 (0.3 to 5.4)	96.7
Age	Middle-age adult	9	35.0 (25.0 to 45.6)	99.2	2	5.6 (4.5 to 6.8)	0
Age	Older adult	9	49.6 (36.7 to 62.6)	98.3	2	9.1 (7.0 to 11.4)	0
Body mass index	Under weight	5	21.8 (11.4 to 34.4)	87.3			
Body mass index	Normal weight	6	21.9 (11.8 to 34.2)	98.6	2	2.3 (1.8 to 2.8)	0
Body mass index	Overweight	6	32.9 (21.2 to 45.8)	97.4	2	4.2 (1.2 to 8.8)	50
Body mass index	Obese	6	45.4 (34.5 to 56.6)	93.3	2	6.4 (4.0 to 9.3)	0
Education Status	Never studied	7	39.1 (27.5 to 51.3)	98	1	5.1 (3.0 to 7.8)	-
Education Status	Less than primary	4	18.3 (13.9 to 23.1)	87.1	1	4.6 (3.4 to 6.1)	-
Education Status	Primary	6	24.8 (12.0 to 40.4)	99.4	1	4.4 (3.6 to 5.2)	-
Education Status	Secondary or higher	7	22.4 (11.1 to 36.2)	99.3	1	4.1 (3.2 to 5.2)	-
Income	Poorest	5	20.9 (10.4 to 33.8)	98.9			
Income	Middle	5	25.3 (10.6 to 43.8)	99.5			
Income	Least poor	5	29.2 (13.1 to 48.5)	98.3			
Smoking status	Yes	5	38.0 (19.1 to 59.0)	99.1			
Smoking status	No	5	30.5 (17.6 to 45.2)	99.6			
Alcohol consumption	Yes	3	26.5 (18.0 to 35.9)	83.4			
Alcohol consumption	No	3	29.1 (9.3 to 54.3)	99.7			
Physically active	Yes	3	28.8 (11.1 to 50.8)	99.6			
Physically active	No	3	30.8 (7.7 to 60.9)	98.4			
Treatment cascade	Aware of HBP	12	33.6 (19.1 to 50.0)	99.7			
Treatment cascade	On treatment	9	51.9 (35.2 to 68.3)	98.6			
Treatment cascade	BP controlled	8	25.9 (18.4 to 34.3)	87.8			

* World Bank Country Income Groups, 2018

For peer review only

For peer review only - http://bmjopen.bmj.com/site/about/guidelines.xhtml

Participants were divided into age groups that, broadly defined, covered young adulthood (18 to 35 years), middle age (36 to 55 years), and older adulthood (56 years and older).

Underweight - BMI under 18.5 kg/m^2

Normal weight - BMI greater than or equal to 18.5 to 24.9 kg/m^2

Overweight – BMI greater than or equal to 25 to 29.9 kg/m^2

Obesity – BMI greater than or equal to 30 kg/m^2

Physical activity as defined by the authors

Alcohol consumption as defined by authors

Smoking status as defined by authors

Income status as reported by authors

For peer review only

FIGURE LEGENDS

Figure 1: Hypertension prevalence estimates among slum residents and 95% confidence intervals from individual studies and pooled data

Figure 2: Type 2 diabetes mellitus prevalence estimates among slum residents and 95% confidence intervals from individual studies and pooled data

Figure 3: Secular trends in hypertension prevalence estimates among slum residents across different regions

Figure 4: Secular trends in Type 2 diabetes mellitus prevalence estimates among slum residents across different regions

Figure 5: Hypertension prevalence estimates by place of residence: urban versus rural versus slum

Figure 6: Type 2 diabetes mellitus prevalence estimates by place of residence: urban versus rural versus slum

For peer review only

For peer review only - http://bmjopen.bmj.com/site/about/guidelines.xhtml

ONLINE ONLY SUPPLEMENTS

eFigure 1: Study selection and inclusion flow chart

eTable 1: List of Excluded Studies

eTable 2: Characteristics of included studies

eTable 3: Risk of bias of included studies

Annex 1: MEDLINE Search Strategy

Annex 2: PRISMA Checklist

For peer review only

For peer review only - http://bmjopen.bmj.com/site/about/guidelines.xhtml

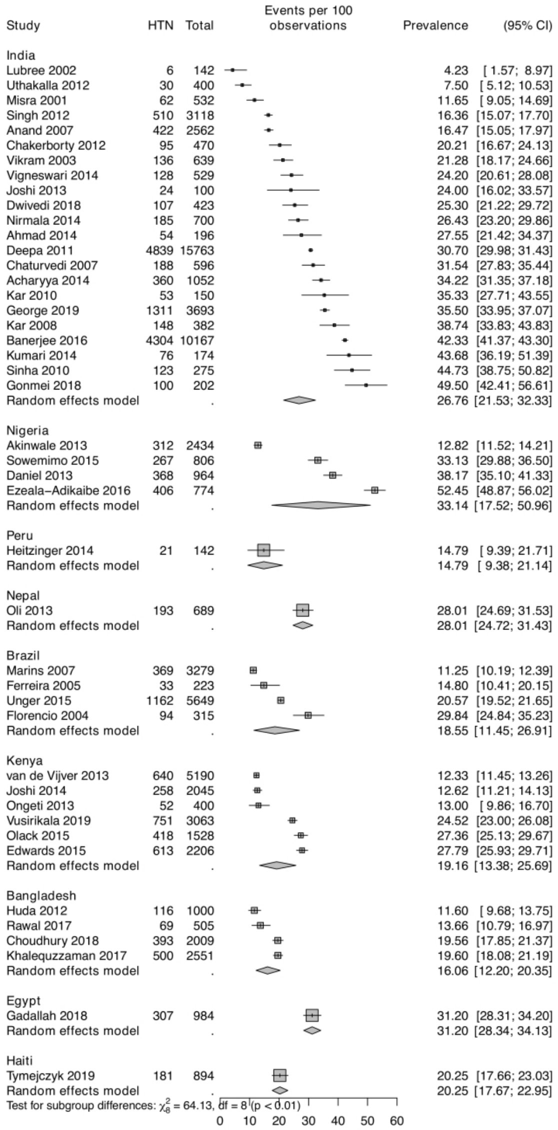

Figure 1

228x406mm (300 x 300 DPI)

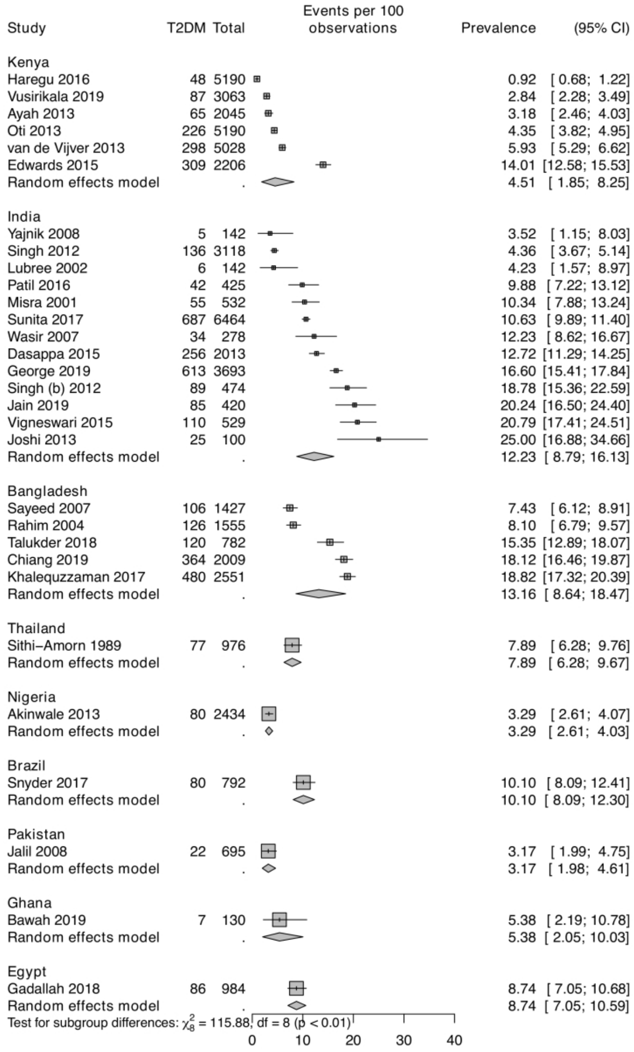

Figure 2

228x355mm (300 x 300 DPI)

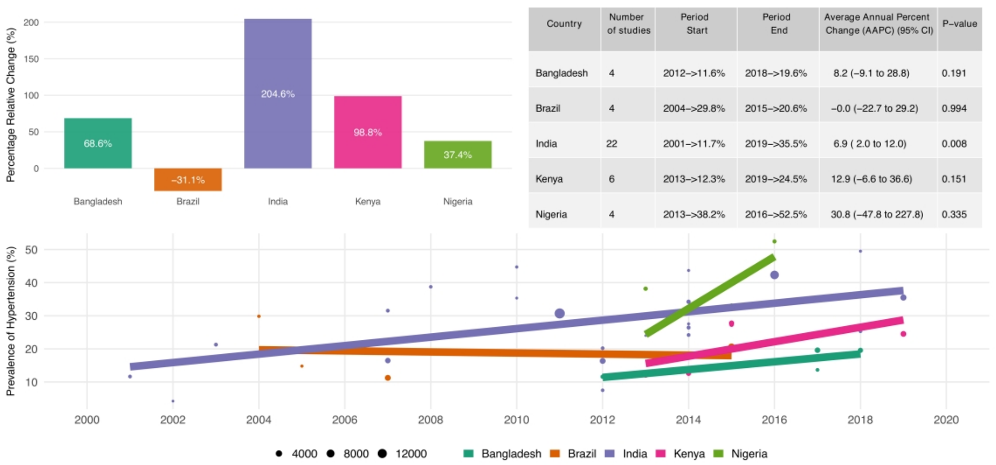

Country	Number of studies	Period Start	Period End	Average Annual Percent Change (AAPC) (95% CI)	P−value
Bangladesh	4	2012−>11.6%	2018−>19.6%	8.2 (−9.1 to 28.8)	0.191
Brazil	4	2004−>29.8%	2015−>20.6%	−0.0 (−22.7 to 29.2)	0.994
India	22	2001−>11.7%	2019−>35.5%	6.9 (2.0 to 12.0)	0.008
Kenya	6	2013−>12.3%	2019−>24.5%	12.9 (−6.6 to 36.6)	0.151
Nigeria	4	2013−>38.2%	2016−>52.5%	30.8 (−47.8 to 227.8)	0.335

Figure 3

496x229mm (300 x 300 DPI)

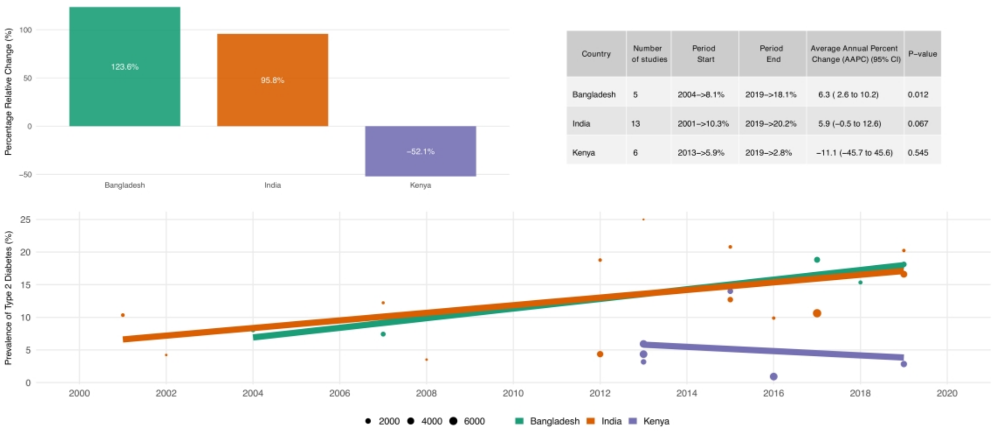

Country	Number of studies	Period Start	Period End	Average Annual Percent Change (AAPC) (95% CI)	P–value
Bangladesh	5	2004–>8.1%	2019–>18.1%	6.3 (2.6 to 10.2)	0.012
India	13	2001–>10.3%	2019–>20.2%	5.9 (–0.5 to 12.6)	0.067
Kenya	6	2013–>5.9%	2019–>2.8%	–11.1 (–45.7 to 45.6)	0.545

Figure 4

602x263mm (300 x 300 DPI)

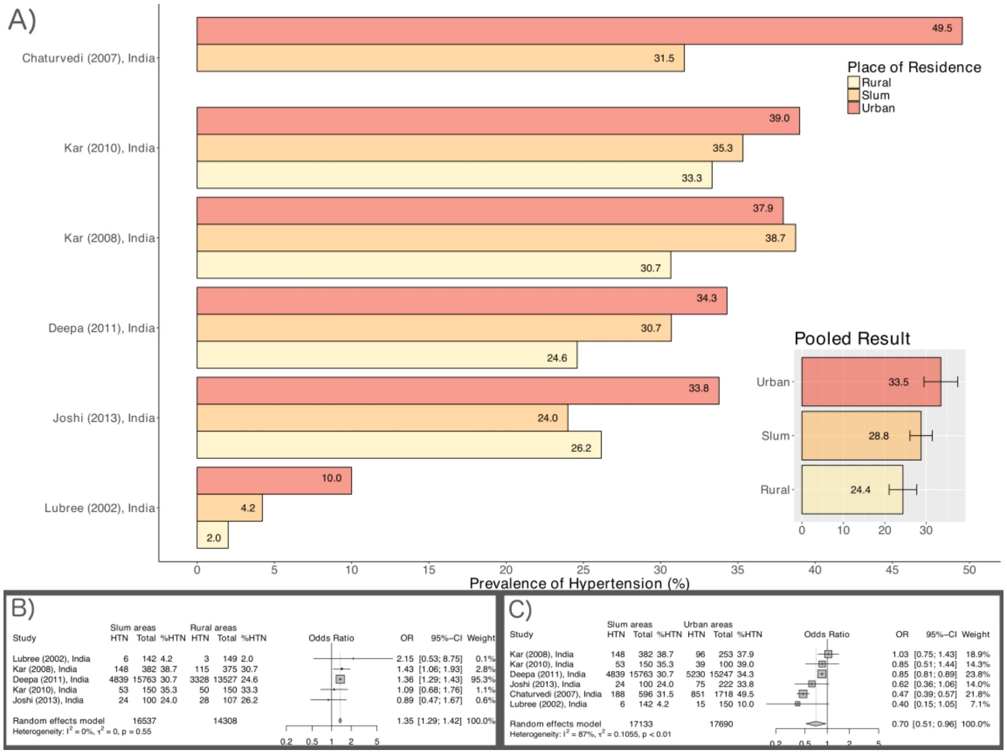

Figure 5

478x357mm (300 x 300 DPI)

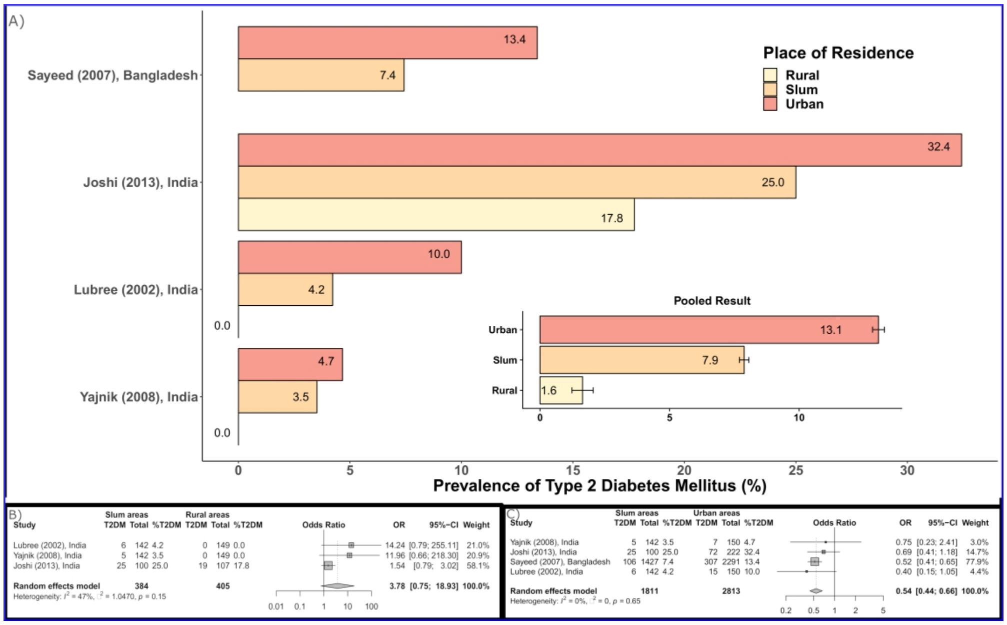

Figure 6

425x261mm (300 x 300 DPI)

Supplementary Digital Content

Table of Contents

eFigure 1: Study selection and inclusion flow chart 1

eTable 1: List of Excluded Studies 2

eTable 2: Characteristics of included studies 10

eTable 3: Risk of bias of included studies 12

Annex 1: MEDLINE Search Strategy 14

For peer review only

eFigure 1: Study selection and inclusion flow chart

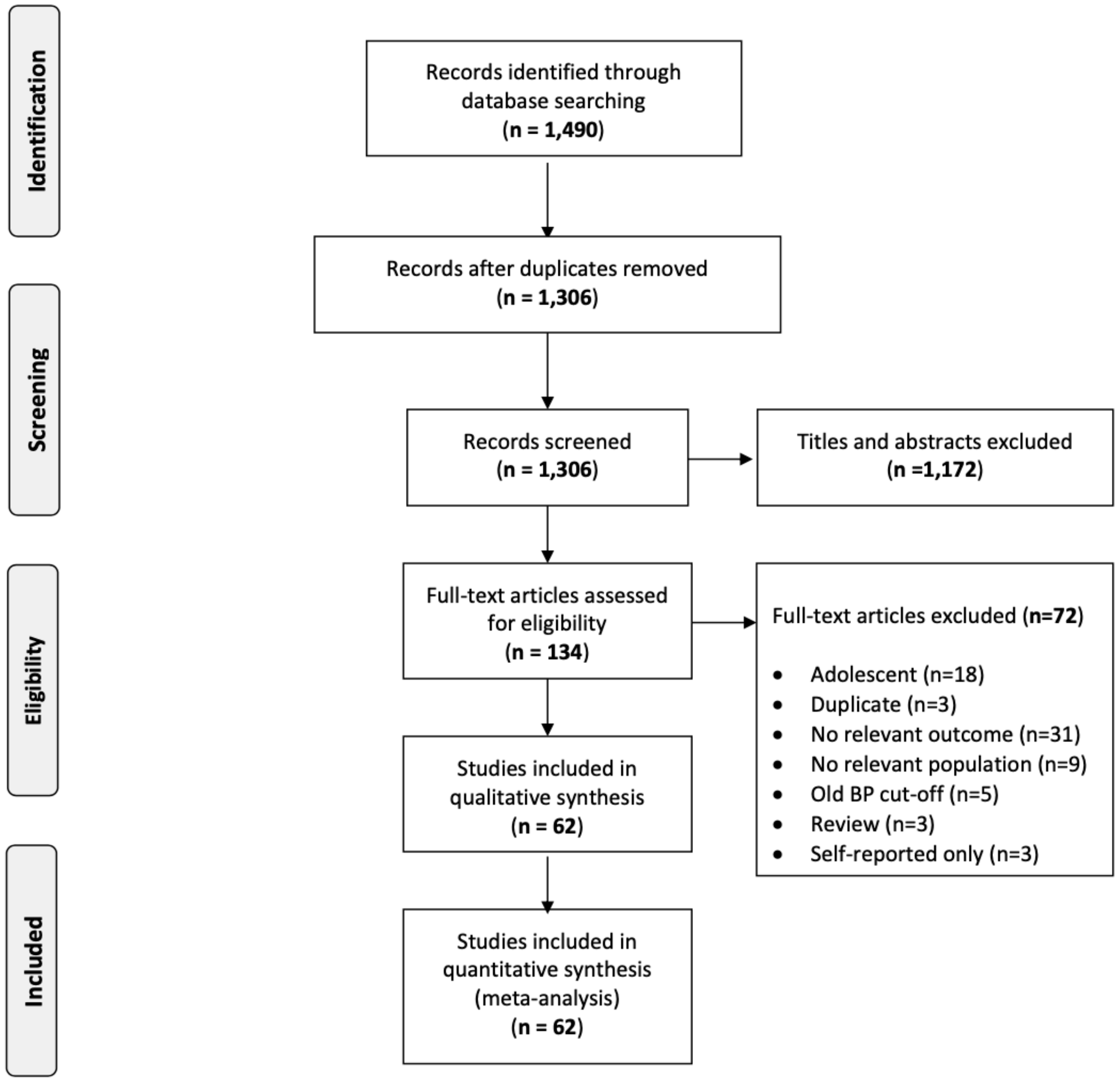

eTable 1: List of Excluded Studies

s/n	Study	Reason
1	Maiti 2016[1]	Adolescent
2	Khopkar 2015[2]	Adolescent
3	Paul 2013[3]	Adolescent
4	Kamath 2012[4]	Adolescent
5	Simsek 2012[5]	Adolescent
6	Saha 2011[6]	Adolescent
7	Oria 2010[7]	Adolescent
8	Saha 2008[8]	Adolescent
9	Saha 2008[9]	Adolescent
10	Sesso 2004[10]	Adolescent
11	Fernandes 2003[11]	Adolescent
12	Zeelie 2010[12]	Adolescent
13	Soudrassanane 2008[13]	Adolescent
14	Werner 2015[14]	Duplicate
15	van de Vijver 2016[15]	Duplicate
16	Haregu 2016[16]	Duplicate
17	Ezenwaka 1997[17]	Old BP cut-off
18	Suriyawongpaisal 1993[18]	Old BP cut-off
19	Suriyawongpaisal 1991[19]	Old BP cut-off
20	Sitthi-Amornn 1989[20]	Old BP cut-off
21	Bunnag 1990[21]	Old BP cut-off
22	E. Sharmin Trisha 2016[22]	No relevant outcome
23	Bhandari 2015[23]	No relevant outcome
24	Oti 2014[24]	No relevant outcome
25	Hiremath 2014[25]	No relevant outcome
26	Joshi 2013[26]	No relevant outcome
27	van de Vijver 2013[27]	No relevant outcome
28	Itrat 2011[28]	No relevant outcome
29	Ahmed 2011[29]	No relevant outcome
30	Haregu 2015[30]	No relevant outcome
31	van de Vijver 2015[31]	No relevant outcome
32	Kohli 2016[32]	No relevant outcome
33	Mudgapalli 2016[33]	No relevant population
34	Natarajan 2014[34]	No relevant population
35	Kumaramanickavel 2014[35]	No relevant population
36	Kumaramanickavel 2015[36]	No relevant population
37	Hulzebosch 2015[37]	No relevant population
38	Madhu 2016[38]	No relevant population
39	Mugure 2014[39]	No relevant population
40	Mukhopadhyay 2012[40]	No relevant population
41	Khan 2010[41]	No relevant population
42	Etyang 2013[42]	Review
43	Dhar 2014[43]	Review
44	Bhargava 1991[44]	Review
46	Kien 2015[45]	Self-reported only
47	Sur 2007[46]	Self-reported only
48	Thakur 2013[47]	Self-reported only
49	Ahmedani 2019[48]	No relevant outcome
50	Ashe 2019[49]	No relevant outcome
51	Asiki 2018[50]	No relevant outcome
52	Bagdey 2019[51]	No relevant outcome
53	Cope 2020[52]	No relevant outcome
54	De Silva 2018[53]	No relevant outcome
55	Kapwata 2018[54]	No relevant outcome
56	Kawazoe 2018 [55]	No relevant outcome

For peer review only - http://bmjopen.bmj.com/site/about/guidelines.xhtml

57	Khanam 2019[56]	No relevant outcome
58	Kolak 2018[57]	No relevant outcome
59	Korn 2018[58]	No relevant outcome
60	Kotian 2019[59]	No relevant outcome
61	Kumar 2018[60]	No relevant outcome
62	Ma 2018[61]	No relevant outcome
63	Maharana 2019[62]	No relevant outcome
64	Nagarkar 2018[63]	No relevant outcome
65	Narendran 2018[64]	No relevant outcome
66	Rajapakshe 2018[65]	No relevant outcome
67	Sarkar 2019[66]	No relevant outcome
68	Scazufca 2019[67]	No relevant outcome
69	Wang 2018[68]	No relevant outcome
70	Wekasah 2020[69]	No relevant outcome
71	Wilson 2020[70]	No relevant outcome
72	Yadav 2018[71]	No relevant outcome
73	Zhang 2019[72]	No relevant outcome

For peer review only

For peer review only - http://bmjopen.bmj.com/site/about/guidelines.xhtml

List of excluded studies

1. Maiti M, Bandyopadhyay L. Variation in blood pressure among adolescent schoolchildren in an urban slum of Kolkata, West Bengal. *Postgraduate Medical Journal (no pagination), 2016* 2016;Date of Publication:July 25. doi: http://dx.doi.org/10.1136/postgradmedj-2016-134227
2. Khopkar SA, Virtanen SM, Kulathinal S. Mental health, anthropometry and blood pressure among adolescents living in slums of Nashik, India. *Tanzania Journal of Health Research* 2015;17(4) doi: http://dx.doi.org/10.4314/thrb.v17i4.6
3. Paul B, Saha I, Mukherjee A. Adolescent Hypertension and Family History. *Pakistan Paediatric Journal* 2013;37(3):177-79.
4. Kamath N, Goud BR, Phadke KD, et al. Use of oscillometric devices for the measurement of blood pressure-comparison with the gold standard. *Indian Journal of Pediatrics* 2012;79(9):1230-32. doi: http://dx.doi.org/10.1007/s12098-011-0600-0
5. Simsek E, Selver B, Dallar Y, et al. Obesity epidemiology in children living in the lower socio-economic status. *Hormone Research in Paediatrics* 2012;Conference:51st Annual Meeting of the European Society for Paediatric Endocrinology. doi: http://dx.doi.org/10.1159/000343184
6. Saha I, Paul B, Mukherjee A, et al. Validity of the WHO criteria for adolescent hypertension. *East African journal of public health* 2011;8(2):135-37.
7. Oria RB, Patrick PD, Oria MOB, et al. ApoE polymorphisms and diarrheal outcomes in Brazilian shanty town children. *Brazilian Journal of Medical and Biological Research* 2010;43(3):249-56.
8. Saha I, Paul B, Dasgupta A. Prevalence of hypertension and variation of blood pressure with age among adolescents in Chetla, India. *Tanzania journal of health research* 2008;10(2):108-11.
9. Saha I, Paul B, Dasgupta A, et al. Variations of adolescent blood pressure by multifactorial analysis in an urban slum of Kolkata. *Journal of the Indian Medical Association* 2008;106(9)
10. Sesso R, Barreto GP, Neves J, et al. Malnutrition is associated with increased blood pressure in childhood. *Nephron Clinical Practice* 2004;97(2):c61-c66. doi: http://dx.doi.org/10.1159/000078402
11. Fernandes MTB, Sesso R, Martins PA, et al. Increased blood pressure in adolescents of low socioeconomic status with short stature. *Pediatric Nephrology* 2003;18(5):435-39.
12. Zeelie A, Moss SJ, Kruger HS. The relationship between body composition and selected metabolic syndrome markers in black adolescents in South Africa: the PLAY study. *Nutrition* 2010;26(11-12):1059-64. doi: 10.1016/j.nut.2010.03.001 [published Online First: 2010/06/15]
13. Soudarssanane M, Mathanraj S, Sumanth M, et al. Tracking of blood pressure among adolescents and young adults in an urban slum of puducherry. *Indian journal of community medicine : official publication of Indian Association of Preventive & Social Medicine* 2008;33(2):107-12. doi: 10.4103/0970-0218.40879 [published Online First: 2008/04/01]
14. Werner ME, van de Vijver S, Adhiambo M, et al. Results of a hypertension and diabetes treatment program in the slums of Nairobi: a retrospective cohort study. *BMC health services research* 2015;15(pp 512) doi: http://dx.doi.org/10.1186/s12913-015-1167-7
15. van de Vijver S, Oti SO, Gomez GB, et al. Impact evaluation of a community-based intervention for prevention of cardiovascular diseases in the slums of Nairobi: the SCALE-UP study. *Glob Health Action* 2016;9(1):30922. doi: 10.3402/gha.v9.30922 [published Online First: 2017/02/06]

For peer review only - http://bmjopen.bmj.com/site/about/guidelines.xhtml

16. Haregu TN, Oti S, Egondi T, et al. Measurement of overweight and obesity an urban slum setting in sub-Saharan Africa: a comparison of four anthropometric indices. *BMC obesity* 2016;3:46. doi: 10.1186/s40608-016-0126-0 [published Online First: 2016/11/12]
17. Ezenwaka CE, Akanji AO, Akanji BO, et al. The prevalence of insulin resistance and other cardiovascular disease risk factors in healthy elderly southwestern Nigerians. *Atherosclerosis* 1997;128(2):201-11. doi: http://dx.doi.org/10.1016/S0021-9150%2896%2905991-6
18. Suriyawongpaisal P, Underwood P. Situation of hypertension in some Bangkok slums. *Journal of the Medical Association of Thailand = Chotmaihet thangphaet* 1993;76(3):123-28.
19. Suriyawongpaisal P, Underwood P, Rouse IL, et al. An investigation of hypertension in a slum of Nakhon Ratchasima. *The Southeast Asian journal of tropical medicine and public health* 1991;22(4):586-94.
20. Sitthi-Amorn C, Chandraprasert S, Bunnag SC, et al. The prevalence and risk factors of hypertension in Klong Toey Slum and Klong Toey government apartment houses. *International Journal of Epidemiology* 1989;18(1):89-94.
21. Bunnag SC, Sitthi-Amorn C, Chandraprasert S. The prevalence of obesity, risk factors and associated diseases in Klong Toey slum and Klong Toey government apartment houses. *Diabetes Res Clin Pract* 1990;10(1)
22. N EST, Jelinek HF, Tarvainen MP, et al. Socioeconomic status, age and heart rate variability in a Bangladeshi community. *Conference proceedings : Annual International Conference of the IEEE Engineering in Medicine and Biology Society IEEE Engineering in Medicine and Biology Society Annual Conference* 2016;01 doi: http://dx.doi.org/10.1109/EMBC.2016.7591919
23. Bhandari S, Sarma PS, Thankappan KR. Adherence to antihypertensive treatment and its determinants among urban slum dwellers in Kolkata, India. *Asia Pacific journal of public health / Asia Pacific Academic Consortium for Public Health* 2015;27(2) doi: http://dx.doi.org/10.1177/1010539511423568
24. Oti SO, van de Vijver S, Kyobutungi C. Trends in non-communicable disease mortality among adult residents in Nairobi's slums, 2003-2011: applying InterVA-4 to verbal autopsy data. *Global health action* 2014;7(pp 25533) doi: http://dx.doi.org/10.3402/gha.v7.25533
25. Hiremath RN, Venkatesh G, Sharvesh, et al. Hypertension status and awareness among geriatric population living in Urban slum. *Nepal Journal of Epidemiology* 2014;Conference:International Conference on Research Methodology and Scientific Writing.
26. Joshi A, Mehta S, Grover A, et al. Knowledge, attitude, and practices of individuals to prevent and manage metabolic syndrome in an Indian setting. *Diabetes Technology and Therapeutics* 2013;15(8):644-53. doi: http://dx.doi.org/10.1089/dia.2012.0309
27. van de Vijver SJ, Oti SO, Agyemang C, et al. Prevalence, awareness, treatment and control of hypertension among slum dwellers in Nairobi, Kenya. *Journal of hypertension* 2013;31(5):1018-24. doi: 10.1097/HJH.0b013e32835e3a56 [published Online First: 2013/02/22]
28. Itrat A, Ahmed B, Khan M, et al. Risk factor profiles of South Asians with cerebrovascular disease. *International Journal of Stroke* 2011;6(4):346-48. doi: http://dx.doi.org/10.1111/j.1747-4949.2011.00622.x
29. Ahmed B, Itrat A, Khan M, et al. Risk factor profiles of south asians with cerebrovascular disease: Findings from a community-based prevalence study in semiurban Pakistan. *Circulation: Cardiovascular Quality and Outcomes* 2011;Conference:Quality of Care

For peer review only - http://bmjopen.bmj.com/site/about/guidelines.xhtml

and Outcomes Research in Cardiovascular Disease and Stroke 2011 Scientific Sessions.

30. Haregu TN, Oti S, Egondi T, et al. Co-occurrence of behavioral risk factors of common non-communicable diseases among urban slum dwellers in Nairobi, Kenya. *Glob Health Action* 2015;8(28697) doi: https://dx.doi.org/10.3402/gha.v8.28697
31. van de Vijver S, Oti S, Moll van Charante E, et al. Cardiovascular prevention model from Kenyan slums to migrants in the Netherlands. *Global health* 2015;11(11):07. doi: https://dx.doi.org/10.1186/s12992-015-0095-y
32. Kohli C, Gupta K. LBOS 03-03 ECONOMIC IMPACT OF HYPERTENSION. *Journal of hypertension* 2016;34 Suppl 1 - ISH 2016 Abstract Book:e551-e52. doi: 10.1097/01.hjh.0000501509.98288.ad [published Online First: 2016/10/19]
33. Mudgapalli V, Sharan S, Amadi C, et al. Perception of receiving SMS based health messages among hypertensive individuals in urban slums. *Technology and Health Care* 2016;24(1):57-65. doi: http://dx.doi.org/10.3233/THC-151097
34. Natarajan S, Mohan S, Satagopan U, et al. Elderly patients with T2DM should be periodically screened for diabetic retinopathy and its complications to reduce visual morbidity - A study from slums of Western India. *Investigative Ophthalmology and Visual Science* 2014;Conference:2014 Annual Meeting of the Association for Research in Vision and Ophthalmology.
35. Kumaramanickavel G, Mohan S, Satagopan U, et al. Diabetic retinopathy in urban slums of Mumbai, India - Social, lifestyle, clinical and genetic risk factors. *Investigative Ophthalmology and Visual Science* 2014;Conference:2014 Annual Meeting of the Association for Research in Vision and Ophthalmology.
36. Kumaramanickavel G, Mohan S, Kumar Singh A, et al. AJDRUMSS-diabetic retinopathy prevalence study in Mumbai slums of India-association of demographic, genetic and medical risk factors. *Investigative Ophthalmology and Visual Science* 2015;Conference:2015 Annual Meeting of the Association for Research in Vision and Ophthalmology.
37. Hulzebosch A, van de Vijver S, Oti SO, et al. Profile of people with hypertension in Nairobi's slums: a descriptive study. *Globalization and health* 2015;11(pp 26) doi: http://dx.doi.org/10.1186/s12992-015-0112-1
38. Madhu B, Srinath KM, Chandresh S, et al. Quality of diabetic care in an urban slum area of Mysore: A community based study. *Diabetes and Metabolic Syndrome: Clinical Research and Reviews* 2016 doi: http://dx.doi.org/10.1016/j.dsx.2016.03.014
39. Mugure G, Karama M, Kyobutungi C, et al. Correlates for cardiovascular diseases among diabetic/hypertensive patients attending outreach clinics in two Nairobi slums, Kenya. *Pan African Medical Journal* 2014;19(no pagination) doi: http://dx.doi.org/10.11604/pamj.2014.19.261.5261
40. Mukhopadhyay A, Sundar U, Adwani S, et al. Prevalence of stroke and post-stroke cognitive impairment in the elderly in Dharavi, Mumbai. *Journal of Association of Physicians of India* 2012;60(10):29-32.
41. Khan RMA, Ahmad M. To assess the public awareness about obesity among adult populace of lahore. *Pakistan Journal of Medical and Health Sciences* 2010;4(4)
42. Etyang A, Harding S, Cruickshank JK. Slum living and hypertension in tropical settings: Neglected issue, statistical artifact or surprisingly slight? Insights amidst adversity. *Journal of Hypertension* 2013;31(5):877-79. doi: http://dx.doi.org/10.1097/HJH.0b013e32836103fb
43. Dhar L. Preventing coronary heart disease risk of slum dwelling residents in India. *Journal of family medicine and primary care* 2014;3(1):58-62. doi: 10.4103/2249-4863.130278 [published Online First: 2014/05/03]

For peer review only - http://bmjopen.bmj.com/site/about/guidelines.xhtml

44. Bhargava SK, Singh KK, Saxena BN. ICMR Task Force National Collaborative Study on Identification of High Risk Families, Mothers and Outcome of their Off-springs with particular reference to the problem of maternal nutrition, low birth weight, perinatal and infant morbidity and mortality in rural and urban slum communities. Summary, conclusions and recommendations. *Indian pediatrics* 1991;28(12):1473-80. [published Online First: 1991/12/01]
45. Kien VD, Van Minh H, Giang KB, et al. Socioeconomic inequalities in self-reported chronic non-communicable diseases in urban Hanoi, Vietnam. *Global Public Health* 2015 doi: http://dx.doi.org/10.1080/17441692.2015.1123282
46. Sur D, Mukhopadhyay SP. A study on smoking habits among slum dwellers and the impact on health and economics. *Journal of the Indian Medical Association* 2007;105(9):492-98.
47. Thakur R, Banerjee A, Nikumb V. Health problems among the elderly: a cross-sectional study. *Annals of medical and health sciences research* 2013;3(1):19-25. doi: 10.4103/2141-9248.109466 [published Online First: 2013/05/02]
48. Ahmedani MY, Fawwad A, Shaheen F, et al. Optimized health care for subjects with type 1 diabetes in a resource constraint society: A three-year follow-up study from Pakistan. *World J Diabetes* 2019;10(3):224-33. doi: 10.4239/wjd.v10.i3.224
49. Ashe S, Routray D. Prevalence, associated risk factors of depression and mental health needs among geriatric population of an urban slum, Cuttack, Odisha. *International Journal of Geriatric Psychiatry* 2019;34(12):1799-807. doi: 10.1002/gps.5195
50. Asiki G, Mohamed SF, Wambui D, et al. Sociodemographic and behavioural factors associated with body mass index among men and women in Nairobi slums: AWI-Gen Project. *Global health action* 2018;11(sup2):1470738-38. doi: 10.1080/16549716.2018.1470738
51. Bagdey PS, Ansari JA, Barnwal RK. Prevalence and epidemiological factors associated with hypertension among post-menopausal women in an urban area of central India. *Clinical Epidemiology and Global Health* 2019;7(1):111-14. doi: 10.1016/j.cegh.2018.02.008
52. Cope AB, Edmonds A, Ludema C, et al. Neighborhood Poverty and Control of HIV, Hypertension, and Diabetes in the Women's Interagency HIV Study. *AIDS Behav* 2020;24(7):2033-44. doi: 10.1007/s10461-019-02757-5
53. De Silva AP, De Silva SHP, Haniffa R, et al. Inequalities in the prevalence of diabetes mellitus and its risk factors in Sri Lanka: a lower middle income country. *Int J Equity Health* 2018;17(1):45-45. doi: 10.1186/s12939-018-0759-3
54. Kapwata T, Manda S. Geographic assessment of access to health care in patients with cardiovascular disease in South Africa. *BMC health services research* 2018;18(1):197-97. doi: 10.1186/s12913-018-3006-0
55. Kawazoe N, Zhang X, Chiang C, et al. Prevalence of hypertension and hypertension control rates among elderly adults during the cold season in rural Northeast China: a cross-sectional study. *J Rural Med* 2018;13(1):64-71. doi: 10.2185/jrm.2959 [published Online First: 2018/05/29]
56. Khanam F, Hossain MB, Mistry SK, et al. Prevalence and Risk Factors of Cardiovascular Diseases among Bangladeshi Adults: Findings from a Cross-sectional Study. *J Epidemiol Glob Health* 2019;9(3):176-84. doi: 10.2991/jegh.k.190531.001
57. Kolak M, Bradley M, Block DR, et al. Urban foodscape trends: Disparities in healthy food access in Chicago, 2007–2014. *Health & Place* 2018;52:231-39. doi: 10.1016/j.healthplace.2018.06.003

For peer review only - http://bmjopen.bmj.com/site/about/guidelines.xhtml

58. Korn A, Bolton SM, Spencer B, et al. Physical and Mental Health Impacts of Household Gardens in an Urban Slum in Lima, Peru. *Int J Environ Res Public Health* 2018;15(8):1751. doi: 10.3390/ijerph15081751
59. Kotian S, Waingankar P, Mahadik V. Assessment of compliance to treatment of hypertension and diabetes among previously diagnosed patients in urban slums of Belapur, Navi Mumbai, India. *Indian Journal of Public Health* 2019;63(4):348. doi: 10.4103/ijph.ijph_422_18
60. Kumar R, Kaur N, Pilania M. Morbidity Pattern of Patients Attending a Primary Healthcare Facility in an Urban Slum of Chandigarh, India. *JOURNAL OF CLINICAL AND DIAGNOSTIC RESEARCH* 2018 doi: 10.7860/jcdr/2018/31331.11297
61. Ma C. The prevalence of depressive symptoms and associated factors in countryside-dwelling older Chinese patients with hypertension. *Journal of Clinical Nursing* 2018;27(15-16):2933-41. doi: 10.1111/jocn.14349
62. Maharana S, Garg S, Dasgupta A, et al. A study on impact of oral health on general health among the elderly residing in a slum of Kolkata: A cross-sectional study. *Indian Journal of Dental Research* 2019;30(2):164. doi: 10.4103/ijdr.ijdr_491_17
63. Nagarkar AM, Kulkarni SS. Obesity and its Effects on Health in Middle-Aged Women from Slums of Pune. *J Midlife Health* 2018;9(2):79-84. doi: 10.4103/jmh.JMH_8_18
64. Narendran M, Rani BBS, Kulkarni P, et al. Interdependence of communicable and Non-Communicable diseases among elderly population in declared slum in Mysuru City, Karnataka. *Indian Journal of Public Health Research & Development* 2018;9(11):62. doi: 10.5958/0976-5506.2018.01426.2
65. Rajapakshe OBW, Sivayogan S, Kulatunga PM. Prevalence and correlates of depression among older urban community-dwelling adults in Sri Lanka. *Psychogeriatrics* 2018;19(3):202-11. doi: 10.1111/psyg.12389
66. Sarkar A, Roy D, Chauhan MM, et al. A lay epidemiological study on coexistent stress in hypertension: Its prevalence, risk factors, and implications in patients' lives. *Journal of family medicine and primary care* 2019;8(3):966-71. doi: 10.4103/jfmpc.jfmpc_60_19
67. Scazufca M, de Paula Couto MCP, Henrique MG, et al. Pilot study of a two-arm non-randomized controlled cluster trial of a psychosocial intervention to improve late life depression in socioeconomically deprived areas of São Paulo, Brazil (PROACTIVE): feasibility study of a psychosocial intervention for late life depression in São Paulo. *BMC public health* 2019;19(1):1152-52. doi: 10.1186/s12889-019-7495-5
68. Wang H, Su M, Fang P-q, et al. Analysis on Medical Expenses of Hypertensive Inpatients in Urban Areas from 2010 to 2013—Evidence from Two Provinces in South of China. *Current Medical Science* 2018;38(4):741-48. doi: 10.1007/s11596-018-1939-5
69. Wekesah FM, Klipstein-Grobusch K, Grobbee DE, et al. Determinants of Mortality from Cardiovascular Disease in the Slums of Nairobi, Kenya. *Glob Heart* 2020;15(1):33-33. doi: 10.5334/gh.787
70. Wilson V, Nittoori S. Risk of type 2 diabetes mellitus among urban slum population using Indian Diabetes Risk Score. *Indian Journal of Medical Research* 2020;152(3):308. doi: 10.4103/ijmr.ijmr_1597_18
71. Yadav S, Saraswat N, Saini AK, et al. A REVIEW ON THE PREVALENCE OF HYPERTENSION IN SIDE-LINED POPULATIONS; SLUM DWELLERS, SHIFT JOB WORKERS AND OCCUPATIONAL NOISE AFFECTED WORKERS: ATTRIBUTABLE TO LIFESTYLE AND ENVIRONMENTAL FACTOR. *Asian Journal of Pharmaceutical and Clinical Research* 2018;11(10):18. doi: 10.22159/ajpcr.2018.v11i10.27007

For peer review only - http://bmjopen.bmj.com/site/about/guidelines.xhtml

72. Zhang X, Chen X, Gong W. Type 2 diabetes mellitus and neighborhood deprivation index: A spatial analysis in Zhejiang, China. *J Diabetes Investig* 2019;10(2):272-82. doi: 10.1111/jdi.12899 [published Online First: 2018/08/28]

For peer review only

For peer review only - http://bmjopen.bmj.com/site/about/guidelines.xhtml

eTable 2: Characteristics of included studies

Study	Country	Slum	Sample size	Age group	% female
Acharyya (2014)	India	North-Parganas	1052	25-64	49.8
Ahmad (2014)	India	Meerut	196	>60	50
Akinwale (2013)	Nigeria	Ijora Oloye, Ajegunle & Makoko	2434		
Anand (2007)	India	Faridabad	2562	15+	50.9
Ayah (2013)	Kenya		2061	18-90	49.1
Banerjee (2016)	India	Kolkata	10167	>20 years	60
Chakerborty (2012)	India	Kolkata	470	18-60	0
Chaturvedi (2007)	India	Delhi	596	>20	
Daniel (2013)	Nigeria	Ajegunle	964	20-81	65.8
Dasappa (2015)	India	Bangalore	2013	35+	50.8
Deepa (2011)	India	Ballabgarh, Delhi, Chennai, Trivandrum , Dibrugarh and Nagpur	15763	15-64	
Edwards (2015)	Kenya	Kibera			
Ezeala-Adikaibe (2016)	Nigeria	Enugu	774	⩾ 20	64.7
Ferreira (2005)	Brazil	Maceio	223	18-65	100
Florencio (2004)	Brazil	Maceio	416	18-60	57
Haregu (2016)	Kenya	Nairobi	5190	18+	46.2
Heitzinger (2014)	Peru	Lima	142	18-81	69.7
Huda (2012)	Bangladesh	Mirpur, Dhaka	1000	15-65	33.4
Jalil (2008)	Pakistan	Lahore	695		43.6
Joshi (2013)	India	Rourkela & Bhubaneswar	100	>18	69
Joshi (2014)	Kenya	Kibera	2045	18-90	49.1
Kar (2008)	India	Chandigarh & Haryana	1010	>30	58.9
Kar (2010)	India	Chandigarh & Haryana	150	>30	62
Khalequzzaman (2017)	Bangladesh	Dhakar	2551	18+	46.7
Kumari (2014)	India	Hyderabad	250		78
Lubree (2002)	India	Pune	150	30-50	100
Marins (2007)	Brazil	Rio-de-Janeiro	3279	>20	56.9
Misra (2001)	India	Gautam-Nagar, Delhi	532		68
Nirmala (2014)	India	Hyderabad, Telangana	700	>20	50.8
Olack (2015)	Kenya	Kibera	1528	35-64	58.1
Oli (2013)	Nepal	Kathmandu	689	15-64	58.9
Ongeti (2013)	Kenya	Kibera	400	14-75	70.3
Oti (2013)	Kenya	Viwandani & Korogocho		18+	46
Patil (2016)	India	Pune, Maharashtra	425	20+	
Rahim (2004)	Bangladesh	Dhakar	1555	20+	52.99
Rawal (2017)	Bangladesh	Dhaka	507		50
Sayeed (2007)	Bangladesh	Dhakar			59.2
Singh (b) (2012)	India	Delhi	474	60+	48
Singh (2012)	India	Patna	3118	>30	56.5
Sinha (2010)	India	Gokulpuri	275	18-40	100
Sithi-Amorn (1989)	Thailand	Klong-Toey	976		54.7

For peer review only - http://bmjopen.bmj.com/site/about/guidelines.xhtml

Snyder (2017)	Brazil		792		64.5
Sowemimo (2015)	Nigeria	Yemetu, Ibadan	806	18-90	
Sunita (2017)	India	Mumbai	6464	>40	
Unger (2015)	Brazil	Salvador	5649	>18	58.3
Uthakalla (2012)	India	Hyderabad		20-60	56
Vigneswari (2014)	India	Chennai	529	18+	77.3
Vigneswari (2015)	India		529	18+	77.3
Vikram (2003)	India	New-Delhi	639		73.4
Wasir (2007)	India	Delhi	278		
Yajnik (2008)	India		142	30-50	0
van de Vijver (2013)	Kenya	Viwandani & Korogocho	5190	>18	46.2
Bawah (2019)	Ghana	Accra	2009		
Chiang (2019)	Bangladesh	Dhaka	423		
Choudhury (2018)	Bangladesh	Dhaka	984	43.4	73
Dwivedi (2018)	India	Bangalore			
Gadallah (2018)	Egypt	West Delhi			
George (2019)	India	Bangalore		57.6	
Gonmei (2018)	India	Delhi			
Jain (2019)	India	Delhi	984	43.4	73
Tymejczyk (2019)	Haiti	Gurugram	420		
Vusirikala (2019)	Kenya	Nairobi		57.6	

For peer review only - http://bmjopen.bmj.com/site/about/guidelines.xhtml

eTable 3: Risk of bias of included studies

Study	Selection (sample population)	Selection (participation rate)	Performance bias (analytical methods to control for bias)	Other form of bias
Acharyya (2014)	Low risk	Low risk	Low risk	Low risk
Ahmad (2014)	Low risk	Unclear risk	High risk	Unclear risk
Akinwale (2013)	Low risk	Low risk	High risk	Unclear risk
Anand (2007)	Low risk	Low risk	Low risk	Low risk
Ayah (2013)	Low risk	Low risk	Low risk	Low risk
Banerjee (2016)	Low risk	Unclear risk	Low risk	Low risk
Chakerborty (2012)	High risk	Low risk	High risk	Low risk
Chaturvedi (2007)	Low risk	Low risk	Low risk	Low risk
Daniel (2013)	Low risk	Low risk	Low risk	Low risk
Dasappa (2015)	Low risk	Low risk	High risk	Low risk
Deepa (2011)	Low risk	Low risk	High risk	Low risk
Edwards (2015)	Low risk	Low risk	High risk	Unclear risk
Ezeala-Adikaibe (2016)	High risk	High risk	Low risk	Low risk
Ferreira (2005)	Low risk	Low risk	Low risk	Low risk
Florencio (2004)	Low risk	Low risk	Low risk	Low risk
Haregu (2016)	Unclear risk	Unclear risk	Low risk	Low risk
Heitzinger (2014)	Low risk	Low risk	Low risk	Low risk
Huda (2012)	Low risk	Low risk	High risk	Unclear risk
Jalil (2008)	Low risk	Low risk	Low risk	Unclear risk
Joshi (2013)	High risk	Low risk	Low risk	High risk
Joshi (2014)	Low risk	Low risk	Low risk	Low risk
Kar (2008)	Low risk	Low risk	Low risk	Low risk
Kar (2010)	Low risk	Low risk	Low risk	Low risk
Khalequzzaman (2017)	Low risk	Low risk	Low risk	Low risk
Kumari (2014)	Low risk	Low risk	High risk	Low risk
Lubree (2002)	Low risk	Low risk	High risk	Low risk
Marins (2007)	Low risk	Low risk	High risk	Unclear risk
Misra (2001)	Low risk	Low risk	High risk	Low risk
Nirmala (2014)	Low risk	Low risk	High risk	Low risk
Olack (2015)	Low risk	Low risk	Low risk	Low risk
Oli (2013)	Low risk	Low risk	Low risk	Low risk
Ongeti (2013)	Low risk	Low risk	Low risk	Low risk
Oti (2013)	Low risk	Low risk	Low risk	Low risk
Patil (2016)	Low risk	Low risk	High risk	Unclear risk
Rahim (2004)	Low risk	Low risk	High risk	Unclear risk
Rawal (2017)	Low risk	Low risk	Low risk	Low risk
Sayeed (2007)	Low risk	Low risk	High risk	Unclear risk
Singh (b) (2012)	Low risk	Low risk	Low risk	Unclear risk
Singh (2012)	Low risk	Low risk	Low risk	Low risk
Sinha (2010)	Low risk	Low risk	Low risk	Low risk
Sithi-Amorn (1989)	Low risk	Low risk	High risk	Unclear risk
Snyder (2017)	Low risk	Low risk	Low risk	Low risk

For peer review only - http://bmjopen.bmj.com/site/about/guidelines.xhtml

Sowemimo (2015)	Low risk	Unclear risk	Low risk	Unclear risk
Sunita (2017)	Low risk	Low risk	High risk	Unclear risk
Unger (2015)	Low risk	Low risk	Low risk	Low risk
Uthakalla (2012)	Low risk	Low risk	High risk	Unclear risk
Vigneswari (2014)	Low risk	Low risk	High risk	Low risk
Vigneswari (2015)	Low risk	Low risk	High risk	Low risk
Vikram (2003)	Low risk	Low risk	Low risk	Low risk
Wasir (2007)	Low risk	High risk	High risk	Unclear risk
Yajnik (2008)	Low risk	Low risk	High risk	Unclear risk
van de Vijver (2013)	Low risk	Low risk	Low risk	Low risk
Bawah (2019)	Unclear risk	Unclear risk	Low risk	Unclear risk
Chiang (2019)	Low risk	Low risk	Low risk	Low risk
Choudhury (2018)	Low risk	Low risk	Low risk	Low risk
Dwivedi (2018)	Low risk	Low risk	Low risk	Low risk
Gadallah (2018)	Low risk	Low risk	Low risk	Low risk
George (2019)	Low risk	Low risk	Low risk	Low risk
Gonmei (2018)	Unclear risk	Unclear risk	Unclear risk	Unclear risk
Jain (2019)	Low risk	Low risk	Low risk	Low risk
Tymejczyk (2019)	Low risk	Low risk	Low risk	Low risk
Vusirikala (2019)	Low risk	Low risk	Low risk	Low risk

For peer review only

Annex 1: MEDLINE Search Strategy

1	exp hypertension/
2	hypertens$.mp.
3	exp blood pressure/
4	(blood pressure or bloodpressure).mp.
5	(essential adj3 hypertension).ti,ab.
6	(isolat* adj3 hypertension).ti,ab.
7	(elevat* adj3 blood adj pressur*).ti,ab.
8	(high adj3 blood adj pressur*).ti,ab.
9	(increase* adj3 blood pressur*).ti,ab.
10	((systolic or diastolic or arterial) adj3 pressur*).ti,ab.
11	essential hypertension.mp.
12	isolated hypertension.mp.
13	elevated blood pressure.mp.
14	high blood pressure.mp.
15	increase blood pressure.mp.
16	diastolic pressure.mp.
17	pre-hypertension.mp.
18	pre-hypertensive.mp.
19	prehypertension.mp.
20	prehypertensive.mp.
21	arterial pressure.mp.
22	cardiovascular diseases/
23	exp coronary disease/
24	cardiovascular risk factor$.tw.
25	(cardiovascular adj3 disease$).tw.
26	(Coronary adj3 disease$).tw.
27	heart disease$.tw.
28	coronary risk factor$.tw.
29	or/1-28

1 exp Diabetes Mellitus, Type 2/
2 exp DIABETES MELLITUS/
3 T2DM.ti,ab.
4 (Type* adj3 ("2" or "II" or two*) adj3 (diabete* or diabetic*)).tw.
5 ((Maturit* or adult* or slow*) adj3 onset* adj3 (diabete* or diabetic*)).tw.
6 ((Ketosis-resistant* or stable*) adj3 (diabete* or diabetic*)).tw.
7 ((Non-insulin* or Non insulin* or Noninsulin*) adj3 depend* adj3 (diabete* or diabetic*)).tw.
8 IDDM.ti,ab.
9 diabet$.ti.
10 PREDIABETIC STATE/
11 prediabet$.ti,ab.
12 impaired glucose tolerance.ti,ab.
13 IGT.ti,ab.
14 Impaired fasting glucose.ti,ab.
15 IFG.ti,ab.
16 Impaired glucose regulation.ti,ab. 1
17 IGR.ti,ab.
18 GLUCOSE INTOLERANCE/
19 (diabet* or glucose or hyperglycaemia or hyperglycaemia or postprandial or post-prandial or insulin or hypoglycemia or hypoglycaemia or IGT or OGTT or CGMS).tw.
20 (subclinical diabetes" or "subclinical diabetic" or "sub-clinical diabetes" or "sub-clinical diabetic").tw.
21 or/1-20

22 (baladi or bandas de miseria or barraca or barrio marginal or barrio or bidonville or brarek or bustee or chalis or chereka bete or dagatan or estero or favela or galoos or gecekondu or hrushebi).mp.
23 (ishash or karyan or katras or looban or loteamento or medina achouaia or morro or mudun safi or musseque or solares or tanake or taudis or township or tugurio or udukku or umjondolo or watta or zopadpattis).mp.
24 (slum or slums or ghetto or ghettos or informal settlement$ or shantytown$ or shanty town$).mp.
25 slum/
26 ghetto/
27 or/22-26

For peer review only - http://bmjopen.bmj.com/site/about/guidelines.xhtml

Annex 2: PRISMA Checklist

Section/topic	#	Checklist item	Reported on page #
**TITLE**			
Title	1	Identify the report as a systematic review, meta-analysis, or both.	1
**ABSTRACT**			
Structured summary	2	Provide a structured summary including, as applicable: background; objectives; data sources; study eligibility criteria, participants, and interventions; study appraisal and synthesis methods; results; limitations; conclusions and implications of key findings; systematic review registration number.	2 -3
**INTRODUCTION**			
Rationale	3	Describe the rationale for the review in the context of what is already known.	6
Objectives	4	Provide an explicit statement of questions being addressed with reference to participants, interventions, comparisons, outcomes, and study design (PICOS).	6-7
**METHODS**			
Protocol and registration	5	Indicate if a review protocol exists, if and where it can be accessed (e.g., Web address), and, if available, provide registration information including registration number.	8
Eligibility criteria	6	Specify study characteristics (e.g., PICOS, length of follow-up) and report characteristics (e.g., years considered, language, publication status) used as criteria for eligibility, giving rationale.	8-9
Information sources	7	Describe all information sources (e.g., databases with dates of coverage, contact with study authors to identify additional studies) in the search and date last searched.	8
Search	8	Present full electronic search strategy for at least one database, including any limits used, such that it could be repeated.	8
Study selection	9	State the process for selecting studies (i.e., screening, eligibility, included in systematic review, and, if applicable, included in the meta-analysis).	9
Data collection process	10	Describe method of data extraction from reports (e.g., piloted forms, independently, in duplicate) and any processes for obtaining and confirming data from investigators.	9
Data items	11	List and define all variables for which data were sought (e.g., PICOS, funding sources) and any assumptions and simplifications made.	9
Risk of bias in individual studies	12	Describe methods used for assessing risk of bias of individual studies (including specification of whether this was done at the study or outcome level), and how this information is to be used in any data synthesis.	10-11
Summary measures	13	State the principal summary measures (e.g., risk ratio, difference in means).	11-12
Synthesis of results	14	Describe the methods of handling data and combining results of studies, if done, including measures of consistency (e.g., I^2) for each meta-analysis.	11-12

For peer review only - http://bmjopen.bmj.com/site/about/guidelines.xhtml

Section/topic	#	Checklist item	Reported on page #
Risk of bias across studies	15	Specify any assessment of risk of bias that may affect the cumulative evidence (e.g., publication bias, selective reporting within studies).	10-11
Additional analyses	16	Describe methods of additional analyses (e.g., sensitivity or subgroup analyses, meta-regression), if done, indicating which were pre-specified.	12
**RESULTS**			
Study selection	17	Give numbers of studies screened, assessed for eligibility, and included in the review, with reasons for exclusions at each stage, ideally with a flow diagram.	13
Study characteristics	18	For each study, present characteristics for which data were extracted (e.g., study size, PICOS, follow-up period) and provide the citations.	13
Risk of bias within studies	19	Present data on risk of bias of each study and, if available, any outcome level assessment (see item 12).	13-14
Results of individual studies	20	For all outcomes considered (benefits or harms), present, for each study: (a) simple summary data for each intervention group (b) effect estimates and confidence intervals, ideally with a forest plot.	14-18
Synthesis of results	21	Present results of each meta-analysis done, including confidence intervals and measures of consistency.	14-20
Risk of bias across studies	22	Present results of any assessment of risk of bias across studies (see Item 15).	13-14
Additional analysis	23	Give results of additional analyses, if done (e.g., sensitivity or subgroup analyses, meta-regression [see Item 16]).	19-20
**DISCUSSION**			
Summary of evidence	24	Summarize the main findings including the strength of evidence for each main outcome; consider their relevance to key groups (e.g., healthcare providers, users, and policy makers).	21-23
Limitations	25	Discuss limitations at study and outcome level (e.g., risk of bias), and at review-level (e.g., incomplete retrieval of identified research, reporting bias).	23
Conclusions	26	Provide a general interpretation of the results in the context of other evidence, and implications for future research.	23
**FUNDING**			
Funding	27	Describe sources of funding for the systematic review and other support (e.g., supply of data); role of funders for the systematic review.	24

For peer review only - http://bmjopen.bmj.com/site/about/guidelines.xhtml

BMJ Open

Global Prevalence and Trends in Hypertension and Type 2 Diabetes Mellitus among Slum Residents: A Systematic Review and Meta-analysis

Journal:	*BMJ Open*
Manuscript ID	bmjopen-2021-052393.R2
Article Type:	Original research
Date Submitted by the Author:	08-Dec-2021

Complete List of Authors:	Uthman, Olalekan; University of Warwick,<br>Ayorinde, Abimbola; University of Warwick, Warwick Centre for Global Health, Warwick Medical School<br>Oyebode, Oyinlola; University of Warwick Warwick Medical School, Warwick Centre for Global Health, Warwick Medical School<br>Sartori, Jo; University of Birmingham, Institute of Applied Health Research<br>Gill, Paramjit ; University of Warwick, Warwick Centre for Global Health, Warwick Medical School,<br>Lilford, RJ; University of Birmingham, Institute of Applied Health Research

**Primary Subject Heading**:	Global health
Secondary Subject Heading:	Global health
Keywords:	Hypertension < CARDIOLOGY, DIABETES & ENDOCRINOLOGY, Public health < INFECTIOUS DISEASES

SCHOLARONE™
Manuscripts

I, the Submitting Author has the right to grant and does grant on behalf of all authors of the Work (as defined in the below author licence), an exclusive licence and/or a non-exclusive licence for contributions from authors who are: i) UK Crown employees; ii) where BMJ has agreed a CC-BY licence shall apply, and/or iii) in accordance with the terms applicable for US Federal Government officers or employees acting as part of their official duties; on a worldwide, perpetual, irrevocable, royalty-free basis to BMJ Publishing Group Ltd ("BMJ") its licensees and where the relevant Journal is co-owned by BMJ to the co-owners of the Journal, to publish the Work in this journal and any other BMJ products and to exploit all rights, as set out in our licence.

The Submitting Author accepts and understands that any supply made under these terms is made by BMJ to the Submitting Author unless you are acting as an employee on behalf of your employer or a postgraduate student of an affiliated institution which is paying any applicable article publishing charge ("APC") for Open Access articles. Where the Submitting Author wishes to make the Work available on an Open Access basis (and intends to pay the relevant APC), the terms of reuse of such Open Access shall be governed by a Creative Commons licence – details of these licences and which Creative Commons licence will apply to this Work are set out in our licence referred to above.

Other than as permitted in any relevant BMJ Author's Self Archiving Policies, I confirm this Work has not been accepted for publication elsewhere, is not being considered for publication elsewhere and does not duplicate material already published. I confirm all authors consent to publication of this Work and authorise the granting of this licence.

For peer review only

Global Prevalence and Trends in Hypertension and Type 2 Diabetes Mellitus among Slum Residents: A Systematic Review and Meta-analysis

Olalekan A. Uthman[1*], Ayorinde A. Abimbola[1], Oyinlola Oyebode[1], Jo Sartori[2], Paramjit Gill[1,3], RJ. Lilford[2]

[1]Warwick Centre for Global Health, Warwick Medical School, University of Warwick, Coventry, UK

[2]Institute of Applied Health Research, University of Birmingham, Birmingham, UK

[3]Academic Unit of Primary Care, Warwick Medical School, University of Warwick, Coventry, UK

***Correspondence author:**

Professor Olalekan A. Uthman, Warwick Centre for Global Health, Division of Health Sciences, Warwick Medical School, The University of Warwick, Coventry, CV4 7AL, United Kingdom. Email: olalekan.uthman@warwick.ac.uk

For peer review only

ABSTRACT

Objective: To obtain regional estimates of prevalence of hypertension and Type 2 diabetes in urban slums, and secondly to compare these with those in urban and rural areas.

Design: Systematic review and meta-analysis

Eligibility criteria: Studies that reported hypertension prevalence using the definition of blood pressure ≥140/90 mm Hg and/or prevalence of type 2 diabetes.

Information sources: Ovid MEDLINE, Cochrane CENTRAL and EMBASE from inception to December 2020

Risk of bias: Two authors extracted relevant data and assessed risk of bias independently using the Strengthening the Reporting of Observational Studies in Epidemiology (STROBE) guideline.

Synthesis of results: We used random-effects meta-analyses to pool prevalence estimates. We examined time trends in the prevalence estimates using meta-regression regression models with the prevalence estimates as the outcome variable and the calendar year of the publication as the predictor.

Results: A total 62 studies involving 108,110 participants met the inclusion criteria. Prevalence of hypertension and type 2 diabetes in slum populations ranged from 4.2% to 52.5% and 0.9% to 25.0%, respectively. In six studies presenting comparator data, all from the Indian sub-continent, slum residents were 35% more likely to be hypertensive than those living in comparator rural areas and 30% less likely to be hypertensive than those from comparator non-slum urban areas.

Limitations of evidence: Of the included studies, only few studies from India compared the slum prevalence estimates with those living on non-slum urban and rural areas, this limits the generalisability of the finding.

Interpretation: The burden of hypertension and type 2 diabetes varied widely between countries and regions and, to some degree, also within countries.

Funding: This research was funded by the National Institute for Health Research (NIHR) (16/136/87) using UK aid from the UK Government to support global health research.

PROSPERO registration number: CRD42017077381

For peer review only - http://bmjopen.bmj.com/site/about/guidelines.xhtml

Strengths and limitations of this study

- To reduce the chance of missing relevant studies, no language constraints were applied during the literature search.
- The data was extracted by two independent reviewers, reducing the possibility of bias.
- We analysed trends over time, and between geographical regions.
- The substantial between studies heterogeneity is an important limitation.
- Of the included studies, only few studies from India compared the slum prevalence estimates with those living on non-slum urban and rural areas, this limits the generalisability of the finding.

For peer review only

For peer review only - http://bmjopen.bmj.com/site/about/guidelines.xhtml

INTRODUCTION

Noncommunicable diseases (NCDs) are currently the leading cause of death globally, even in low- and middle-income countries (LMICs) the burden of disease is shifting from infectious diseases to NCDs[1]. NCDs now account for about 41 million deaths annually, corresponding to nearly 7 in 10 of all deaths worldwide. Every year, 15 million people of ages of 30 to 69 years die from these diseases, more than 85% of which are people living in LMICs. Most of the deaths from NCDs are caused by cardiovascular diseases, followed by cancer and respiratory diseases. NCDs affect people in all age groups, countries and geographic regions. The leading causes of these diseases include increased consumption of unhealthy foods, increased physical inactivity and population ageing[2-4]. These factors are mediated through metabolic risk factors for NCDs the most common of which include hypertension and type 2 diabetes[2-4]

Urbanization is a global phenomenon that is occurring at a fast pace in most LMICs[5 6]. For more than 20 years, urban settlements have been increasing in population size because of fast growth in urban births, significant movement of people from rural areas and sustained integration of the global economy [5 6]. The United Nations defines slums as urban areas with overcrowding, poor sanitation infrastructure, limited access to safe water, and/or poor structural quality of housing[7 8]. Slums are now an important component of today's urban settlements and likely continue to be for the foreseeable future [7 8].

For peer review only - http://bmjopen.bmj.com/site/about/guidelines.xhtml

Despite increased global awareness about the presence and persistence of slums, and evidence that their popualtions are affected by different health problems and needs to other urban inhabitants, the health of their inhabitants is under researched[7-10]. The health of the urban poor, people with low socioeconomic status living in urban areas, is usually conflated with that of slum residents. Although there is substantial overlap between these groups, there are also richer residents within slum neighbourhoods, as well as urban poverty occuring in non-slum urban areas. Health outcomes for these two groups may differ depending on whether deprivation is at the individual (urban poverty) or neighbourhood level (slum resident) due to neighbourhood effects [7 8 11 12]. For exampe, with respect to NCD risk-factors, those resident in slums, whatever their personal socio-economic status, may be more exposed to a common physical environmental risk factors (for example: air pollution increasing risk of hypertension), social environmental risk factors (for example: crime rates which may increase stress and drive metabolic risk) or institutional risk-factors (for example: stigma on the basis of their address reducing access to appropriate medical care). Many existing studies of NCDs risk factors done in urban areas do not disaggregate the population's health data by slum and non-slums status to allow for the detection of intra-urban health disparities that are due to neighbourhood effects rather than individual socio-economic status[13-22].

Understanding how the global challenges of hypertension, type 2 diabetes and rapid unplanned urbanisation intersect, by investigating whether the up to 1 billion people residing in slums[23]are succumbing to these important metabolic risk factors for non-communicable disease, will inform priorities for health services and health policy in LMICs. To fill this research gap, we therefore systematically gathered all the publications that relate to the burden of

For peer review only - http://bmjopen.bmj.com/site/about/guidelines.xhtml

hypertension among slum residents to (1) assess the contemporary prevalence esimates of hypertension among slum residents (2) compare the prevalence of hypertension and Type 2 diabetes in slums with those in two other types of settlement i.e. non-slum urban and rural areas; and (3) assess the proportion of those with hypertension who were aware of their hypertensive status, those on treatment and those with blood pressure under control.

For peer review only - http://bmjopen.bmj.com/site/about/guidelines.xhtml

For peer review only

METHODS

Protocol and registration

The study background, rationale, and methods were specified in advance and documented in a protocol that was published in the PROSPERO register (CRD42017077381).

Search and information sources:

We searched Ovid MEDLINE, Cochrane CENTRAL and EMBASE from inception to December 2020 using the following keywords: slum, shanty town, ghetto, hypertension and type 2 diabetes. The search strategy for Medline is shown in **Annex 1**.

Eligibility criteria:

We evaluated each identified study against the following pre-defined selection criteria:

- *Types of studies:* We included all studies (cross-sectional studies, retrospective or prospective cohort studies) that reported prevalence of hypertension and type 2 diabetes mellitus among slum residents as a primary or secondary outcome. No language, publication date or publication status restrictions were imposed.
- *Types of participants:* adult population (18 years and above) living in slum (as defined by the authors of the original studies included).
- *Types of Interventions:* Not applicable.
- *Types of outcomes:* Essential hypertension (also called primary or idiopathic hypertension), defined as persistent (seated) systolic blood pressure (SBP) of 140 mmHg or greater or had diastolic blood pressure 90mmHg or greater regardless of age and sex. We excluded studies that included subjects with pregnancy-induced, pre-

For peer review only - http://bmjopen.bmj.com/site/about/guidelines.xhtml

eclampsia, malignant, portal, pulmonary, renal, intracranial or ocular hypertension. We also excluded studies used only self-reported measure, i.e. deducible from the use of antihypertensive drugs or self-reported physician-diagnosed cases. If data were available, we noted (1) the percentage of those aware of their hypertension status (2) on any anti-hypertensive treatment, and (3) blood pressure controlled to a target level. Awareness of hypertension was defined as self-reporting of any prior diagnosis of hypertension by a healthcare professional. Treatment of hypertension was defined as receiving prescribed antihypertensive medication for management of high BP at some time in the 1 year preceding the survey. Control of hypertension was defined as the proportion of patients reporting antihypertensive therapy with SBP of less than 140 mmHg and DBP of less than 90 mmHg.

Type 2 diabetes was defined based on measured fasting plasma glucose, or oral glucose tolerance test. Type 2 diabetes was diagnosed if the fasting blood glucose was ≥126 mg/dL (≥7.0 mmol/L) after an overnight fast for at least 8 hours, or random capillary blood glucose of >= 11.1 mmol/L or if the participant was taking treatment for type 2 diabetes.

Study selection

Two reviewers (OAU, AAA) independently evaluated the eligibility and methodological quality of the studies obtained from the literature searches. All articles yielded by the database search were initially screened by their titles and abstracts to obtain studies that met inclusion criteria. In cases of discrepancies, agreement was reached by discussion with a third reviewer. Two reviewers (OAU, AAA) independently then independently evaluated the full-text articles

For peer review only - http://bmjopen.bmj.com/site/about/guidelines.xhtml

of all identified citations to establish relevance of the article according to the pre-specified criteria. In cases of discrepancies, agreement was reached by discussion with a third reviewer.

Data collection process and data items

OAU extracted data and AAA and OO checked the extracted data. For each study that met the selection criteria, details extracted included on year of publication, country of origin, study design, sample size, sampling strategy, study period, setting (rural/urban/slum), socio-demographic variables, prevalence estimates; etc.

Risk of bias (quality) assessment

We used the Risk of Bias Assessment tool for Non-randomized Studies (RoBANS)[24] to assessed the risk of bias of included studies (see Box 1). The risk of bias in a study was graded as low, high or unclear on the basis of study features including the selection (selection of participants and confounding variables), performance (measurement of exposure), detection (blinding of outcome assessments), attrition (incomplete outcome data) and reporting (selective outcome reporting).

For each included study, we estimated the precision (C) or margin of error, considering the sample size (SS) and the observed prevalence (p) of hypertension among slum dwellers from the formula:

$$SS = Z^2 * p * (1-p)/C^2 \qquad (1)$$

where Z was the z-value fixed at 1.96 across studies (corresponding to 95% confidence interval). The desirable margin of error is 5% (0.05) or lower.

For peer review only - http://bmjopen.bmj.com/site/about/guidelines.xhtml

Synthesis of results

For the meta-analysis, we used DerSimonian-Laird random effects model[25] due to anticipated variations in study population, health care delivery systems and stage of epidemic transition to pool the hypertension and type 2 diabetes prevalence estimates. We performed leave-one-study-out sensitivity analysis to determine the stability of the results[26]. This analysis evaluated the influence of individual studies by estimating the pooled prevalence estimates in the absence of each study[26]. We assessed heterogeneity among studies by inspecting the forest plots and using the chi-squared test for heterogeneity with a 10% level of statistical significance and using the I^2 statistic where we interpret a value of 50% as representing moderate heterogeneity[27 28]. We assessed the possibility of publication bias by evaluating a funnel plot for asymmetry. Because graphical evaluation can be subjective, we also conducted a Egger's regression asymmetry test as formal statistical tests for publication bias[29].

Following the overall analyses, we performed the following sub-group analyses: place of residence (rural versus urban slum versus non-slum urban); participants risk factors, including socioeconomic position; study design (cross-sectional, cohort); study location (low- and middle income versus high-income countries); and study precision.

We examined time trends in the prevalence estimates using meta-regression regression models with the prevalence estimates as the outcome variable and the calendar year of the publication as the predictor. In order to measure secular patterns in prevalence figures, we use the annual average percentages change (AAPC). We fitted a regression line to the natural logarithm of the prevalence estimates, i.e., $y = \alpha + \beta x + \varepsilon$, where y = ln(Prevalence), and x = calendar year. The AAPC was calculated as $100 \times (\exp(\beta)-1)$. The 95% confidence interval (CI) of the AAPC was also computed from the regression model. [30] The prevalence calculations indicated an

For peer review only - http://bmjopen.bmj.com/site/about/guidelines.xhtml

upward trend when both the AAPC estimate and the lower limit of its 95% CI were > 0. However, they indicated a downward trend when both the AAPC and its upper limits were less than 0. The prevalence estimates were otherwise considered stable over time[30]. This systematic review was reported according to the Preferred Reporting Items for Systematic Reviews and Meta-analyses (PRISMA) guideline (**Annex 2**)[31].

Patient and public involvement

No patient was involved.

Results

Study selection and characteristics

The literature search yielded 1490 articles. **eFigure 1** shows the study selection flow diagram. After review, 135 articles were selected for critical reading. Seventy-two studies did not meet the inclusion criteria and were excluded (see **eTable 1** for list of excluded studies). The other 62 studies involving 108,110 participants met the inclusion criteria and were included in the meta-analysis[13-22 32-80]. Forty-three studies reported only hypertension prevalence estimates, 29 studies reported only type 2 diabetes prevalence estimates and eight reported both. **Table 1 and eTable 2** presents the characteristics of the included studies. The studies were reported between 1989 and 2019. Studies were reported as full-text journal articles (n=61, **98%);** except for one which was reported as a conference abstract. The number of participants included in the studies ranged from 100 to 15,763. When reported, the mean age of participants ranged from 32 years to 47 years. Most of the studies were carried out in South Asia: India (n=30); Bangladesh (n=8) and Nepal (n=1) and Pakistan (n=1); followed by sub-Saharan Africa: Kenya (n=9) and Nigeria (n=4); Latin America and Caribbean: Brazil (n=5) and Peru (n=1) and East Asia and Pacific: Thailand (n=1). Most of the studies were conducted in

For peer review only - http://bmjopen.bmj.com/site/about/guidelines.xhtml

the following urban slums: Kibera (n=4), Delhi (n=3), Hyderabad (n=3), Ajegunle (n=2), Chandigarh (n=2), Chennai (n=2), Dhaka (n=2), Haryana (n=2), and Maceio (n=2).

Risk of bias of included studies

Summary of risk of bias assessment for each study is shown in **eTable 3.** The risk of bias in the selection of participants was low in most studies (n=56, 90%), high in three studies (5%) and unclear in three studies (5%). Risk of bias due to confounding variables was low in most studies (n=39, 63%), high in 22 studies (36%) and unclear in one study. Risk of bias due to measurement of exposure, blinding of outcome assessments and selective outcome reporting was low in all the 62 studies as we included all studies that used objective measure of hypertension and type 2 diabetes. Risk of bias due to incomplete outcome data was low in most studies (n=54, 87%), high in 2 studies (3%) and unclear in six studies (10%).

Variations in prevalence of hypertension and type 2 diabetes by geographical regions

Prevalence of hypertension and type 2 diabetes from individuals are shown in **Figure 1 and Figure 2** respectively.

East Asia and Pacific

Thailand: One study from Klong-Toey slum found that 77 of the 976 respondents had type 2 diabetes in 1989 (7.9%, 95% CI 6.3 to 9.8).

Latin America and Caribbean

Brazil: Four studies reported the prevalence of hypertension from three different slums: Maceio (n=2), Rio de Janeiro (n=1) and Salvador (n=1). Florencio et al. found that almost one

For peer review only

For peer review only - http://bmjopen.bmj.com/site/about/guidelines.xhtml

third of the Maceio slum dweller were hypertensive in 2004 (29.8%, 95% CI 24.8 to 35.2), while Ferriera et al estimated prevalence of hypertenssion among Maceio slum residents to be 14.8% (95% 10.4 to 20.2) in 2005. The reported prevalence of hypertension in other slums was 11.3% (95% CI10.2 to 12.4) in Rio de Janerio in 2007 and 20.6% (95% CI 19.5 to 21.7) in Salvador in 2015. The pooled prevalence ('annualised year average') of hypertension for the four studies yielded an estimate of 18.4% (95% CI 12.0% to 26.2%). One study from Brazil found that one in ten had type 2 diabetes in 2017.

Peru: One study from a Lima slum conducted in 2014 found that 21 of the 142 respondents were hypertensive (14.8%, 95% CI 9.4 to 21.7).

South Asia

Bangladesh: Four studies from Dhakan slum reported prevalence of hypertension. The reported prevalence of hypertension ranged from 11.6% (95% CI 9.7 to 13.8) in 2012 to 19.56% (95% CI 17.85 to 21.37) in 2018. Fivestudies from Dhakan slum reported prevalence of type 2 diabetes. The pooled prevalence ('annualised year average') of hypertension for the three studies yielded an estimate of 16.1% (95% CI 12.2% to 20.3%). The reported prevalence of type 2 diabetes in these slums ranged from 8.1% (95% CI 6.8 to 9.6) in 2004 to 18.12% (95% CI 16.46 to 19.87) in 2019.

India: Twenty-two studies from India reported prevalence of hypertension from more than 15 difference slums. The reported prevalence varied across and within the slums. For example, Kar and colleagues estimated the prevalence of hypertension of 27.6% (95% 21.4 to 34.4) among 196 Chandigarh and Haryana slum residents in 2008; however they estimated

For peer review only - http://bmjopen.bmj.com/site/about/guidelines.xhtml

the prevalence of hypertension of 16.5% (95% CI 15.1 to 18.0) among 2,562 196 Chandigarh and Haryana slum residents in 2010. Prevalence of type diabetes also varied across slums in India. The pooled prevalence ('annualised year average') of hypertension for the 22 studies yielded an estimate of 26.8% (95% CI 22.5% to 31.3%). In Delhi, the reported prevalence of type 2 diabetes ranged from 12.7% (95% CI 11.3 to 14.2) in 2007 to 31.5% (95% CI 27.8 to 35.4) in 2012. The pooled prevalence ('annualised year average') of type 2 for the 13studies yielded an estimate of 12.2% (95% CI 9.2% to 15.6%).

Nepal: One study from a Kathmandu slum conducted in 2013 found that 193 of the 689 respondents were hypertensive (28.0%, 95% CI 24.7 to 31.5).

Pakistan: One study from a Lahore slum found that 22 of the 695 respondents had type 2 diabetes in 2008 (3.2%, 95% CI 2.0 to 4.8).

Sub-Saharan Africa. *Kenya:* Six studies reported the prevalence of hypertension from three different slums: Kibera (n=4) and Viwandani and Korogocho (n=2). The reported prevalence among Kibera slum residents ranged from 13.0% (95% CI9.9 to 16.7) in 2013 to 27.8% (95% CI 25.9 to 29.7) in 2015. van de Vijver found that 640 of the 5,190 respondents from Viwandani and Korogocho slum residents were hypertensive (12.3%, 95% CI 11.5 to 13.3). The pooled prevalence ('annualised year average') of hypertension for the six studies yielded an estimate of 19.2% (95% CI 13.2% to 26.0%). The reported prevalence of type 2 diabetes ranged from 0.9% (95% CI 0.7 to 1.2 in Nairobi slum in 2016 to 4.4% (95% CI 3.8 to 5.0) in Viwandani and Korogocho in 2013. The pooled prevalence ('annualised year average') of type 2 diabetes for the six studies yielded an estimate of 4.5% (95% CI 2.0% to 7.9%).

For peer review only - http://bmjopen.bmj.com/site/about/guidelines.xhtml

Nigeria: Four studies from five different slums reported prevalence of hypertension. The reported prevalence varied across and within the slums. Ezeala-Adikaibe found that half of the respondents from Enugu slum were hypertensive in 2016 (52.5%, 95% CI 48.9 to 56.0). While Daniel et al. and Sowemimo et al. found that almost one-third of the Ajegule (38.2%, 95% CI 35.1 to 41.3, 2013) and Yemetu (33.1%, 95% CI 30.0 to 36.5, 2015) slum residents were hypertensive. However, Akinwale found that only 12.8% of the respondents from Ijora Oloye, Ajegunle and Makoko were hypertensive in 2013. The pooled prevalence ('annualised year average') of hypertension for the four studies yielded an estimate of 33.2% (95% CI 15.6% to 53.5%). Akinwale found that only 3.3% of the respondents from Ijora Oloye, Ajegunle and Makoko had type 2 diabetes in 2013.

Secular trends in hypertension and Type 2 diabetes prevalence estimates

Secular trends in hypertension, in 5 countries for which there were data across multiple time points, and type 2 diabetes, in 3 countries in which we had data across multiple time points, among slum residents are shown in **Figures 3 and 4**. We observed a continuous increase in prevalence of hypertension among slum residents in four out of five countries. The increase is more pronounced in India, followed by Kenya and Bangladesh. The prevalence of hypertension increased by 204.6% from 11.7% in 2001 to 35.5% in 2019 in India. The prevalence of hypertension increased by 98.8% from 12.3% in 2013 to 24.5% in 2019 in Kenya. However, the results of the trend analysis showed statistically significant upward trends only in India, such that the prevalence of hypertension increased +6.9% (95% CI +2.0% to +12.0%) per year between 2001 and 2019. There was no statistically significant trend was observed in Brazil using trend analyses (trend =-0.0%, 95% CI −22.7% to +29.2%). We also observed a

For peer review only - http://bmjopen.bmj.com/site/about/guidelines.xhtml

continuous increase in prevalence of type 2 diabetes among slum residents in India and Bangladesh. The prevalence of type 2 diabetes increased by 123.6% from 8.1% in 2004 to 18.1% in 2019 in Bangladesh. The prevalence of type 2 diabetes increased by 95.8% from 10.3% in 2001 to 20.2% in 2019 in India. However, the results of the trend analysis showed statistically significant upward trends only in Bangladesh such that the prevalence of type 2 diabetes increased +5.9% (95% CI +1.1% to +10.8%) per year between 2004 and 2019. A non-statistically significant downward trends in type 2 diabetes prevalence was also observed in Kenya (trend =-11.1%, 95% CI −45.7% to +45.6%).

For peer review only

Prevalence of hypertension by different hypertension and type 2 diabetes subgroups

Study characteristics: As shown in **Table 1**, the pooled prevalence of hypertension was highest in studies conducted in lower-middle income countries (23.2%, 95% CI 21.5 to 29.0, 36 studies) than those from upper-middle income countries (17.9%, 95% CI 12.1 to 24.6, 5 studies). The pooled prevalence of hypertension tended to be higher among studies from South Asia (25.3%, 95% CI 21.3 to 29.6, 26 studies) and sub-Saharan Africa (24.4%, 95% CI 17.7 to 31.9, 10 studies) than those from Latin America and Carribean (18.3%, 95% CI 13.4 to 23.9, 6 studies). The pooled prevalence tended to higher among imprecise studies (33.4%, 95% CI 25.7 to 41.7, 8 studies) than those from precise studies (22.4%, 95% CI 18.9 to 26.1%, 35 studies). The pattern was similar for type 2 diabetes prevalence estimates.

Socio-demographic characteristics: As shown in **Table 1**, the pooled prevalence of hypertension was similar among males (22.5%, 95% CI 16.0 to 29.7, 24 studies) and females (23.5%, 95% CI 18.6 to 28.1, 24 studies). The pooled prevalence of hypertension tended to

For peer review only - http://bmjopen.bmj.com/site/about/guidelines.xhtml

be higher among older adults (49.6%, 95% CI 36.7 to 62.6, 9 studies) than middle-age (35.0%, 95% CI 45.6, 9 studies) and young adults (15.7%, 95% CI 10.1 to 22.1, 8 studies). Similarly, the pooled prevalence of hypertension tended to be higher obese (45.4%, 95% CI 34.5 to 56.5, 6 studies) and overweight (32.9%, 95% CI 21.2 to 45.8, 6 studies) participants than participants with normal (21.9%, 95% CI 11.8 to 34.2, 6 studies) and under-weight (21.8%, 95% CI 11.4 to 34.4, 5 studies). The pooled prevalence of hypertension tended to be higher among those never studied (39.1%, 95% CI 27.5 to 51.3) than those with less than primary (18.3%, 95% CI 13.9 to 23.1, 4 studies), primary (24.8%, 95% CI 12.0 to 40.4, 6 studies) or secondary/higher education attainment (22.4%, 95% CI 11.2 to 36.2, 7 studies). The pooled prevalence of hypertension tended to be higher among least poor (29.2%, 95% CI 13.1 to 48.5, 5 studies) than those with middle- (25.3%, 10.6 to 43.8, 5 studies) and poorest-income (20.9%, 95% CI 10.4 to 33.8, 5 studies). The pattern was similar for type 2 diabetes prevalence estimates.

Lifestyle factors: The pooled prevalence of hypertension tended to be higher among smokers (38.0%, 95% CI 19.1 to 59.0, 5 studies) than those not smoking (30.5%, 95% CI 17.6 to 45.2, 5 studies). We found that the pooled prevalence of hypertension tended to be higher those not physically active (30.8%, 95% CI 7.7 to 60.9, 3 studies) than those physical active (28.8%, 95% CI 11.1 to 50.8); tended to be higher among with no history of alcohol consumption (29.1%, 95% CI 9.3 to 54.3, 3 studies) than those reported alcohol consumption (26.5%, 95% CI 18.0 to 35.9, 3 studies).

Comparative prevalence by place of residence

For peer review only

Six studies from India included non-slum populations alongside data from the slum population, and reported prevalence of hypertension by place of residence[36 38 46 48 49 51]. As shown in **Figure 5**, the pooled prevalence of hypertension was highest among those residing in non-slum urban areas (33.5%, 95% CI 26.0 to 42.0, 6 studies), followed by urban slum residents (28.8%, 95% CI 23.7 to 34.4%, 6 studies) and was lowest among rural residents (24.4%, 95% 18.4 to 31.5, 5 studies). Slum residents were 35% more likely to be hypertensive than those living in rural areas (OR = 1.35, 95% 1.29 to 1.42) and 30% less likely to be hypertensive than those living in other urban areas (OR = 0.70, 95% CI 0.51 to 0.96).

Four studies from India (n=3) and Bangladesh reported prevalence of Type 2 diabetes by place of residence[46 51 59 71]. As shown in **Figure 6**, the pooled prevalence of type 2 diabetes was highest among those residing in non-slum urban areas (13.06%, 95% CI 6.53 to 24.43, 4 studies; 2813 participants), followed by urban slum residents (7.88%, 95% CI 3.32 to 17.55; 4 studies; 1811 participants) and was lowest among rural residents (1.64%; 95% CI 0.06 to 32.21; 3 studies; 405 participants). Such that prevalence of type 2 diabetes tended to be higher among urban slum residents than those living in rural areas (OR = 3.78, 95% 0.75 to 18.93). Urban slum residents were 46% less likely to be diabetic than those from other urban areas (OR = 0.54, 95% CI 0.44 to 0.66).

Treatment cascade

Among those diagnosed with hypertension, only one-third were aware of their hypertensive status (33.6%, 95% CI 19.1 to 50.0%, 12 studies) (**Table 1)**. Among those aware of their high blood pressure, half of them were on antihypertensive medications (51.9%, 95% CI 35.2 to 68.3, 9 studies). Among those on treatment, only one-quarter had good blood pressure

For peer review only - http://bmjopen.bmj.com/site/about/guidelines.xhtml

control (25.2, 95% CI 18.4 to 34.3, 8 studies). Among those diagnosed with type 2 diabetes, 57.4% were aware of their type 2 diabetes status (95% CI 18.2 to 91.8%, 2 studies).

Discussion

Main Findings

This systematic review and meta-analysis summarises available evidence on the global prevalence of hypertension and type 2 diabetes among slum residents. There were several key findings: firstly, the burden of hypertension and type 2 diabetes among slum dweller is high and may be rising globally, with wide variation between countries and regions and, to some degree, also within countries. Using data from within study comparator populations when presented, the pooled prevalence of hypertension and Type 2 diabetes was highest among those residing in non-slum urban areas, followed by slum residents and was lowest among rural residents. This finding corroborates those of previous reviews that observed higher prevalence of hypertension among urban residents than those living in rural areas[81 82]. This high prevalence may be due to rapid urbanization, lifestyle changes, dietary changes and increased life expectancy[83 84] or a combination of these factors[85 86]. In addition, the observed difference could be due to other factors including but not limited to lack of access to testing and care of NCDs risk factors in rural areas and urban areas.

The observed gradient in burden of hypertension and Type 2 diabetes among rural, slum and urban residents is consistent with the effects of urbanization and wealth, as residents

For peer review only - http://bmjopen.bmj.com/site/about/guidelines.xhtml

experience an economic transition when moving from one area to the next[87-92]. LMICs are now undergoing epidemiological transition, the change from a burden of infectious diseases to chronic diseases [93]. In addition, it could be due to increase in awareness in (non-slum) urban areas and recent availability of testing in some places. Recent systematic reviews of dietary risk-behaviour in Sub-Saharan Africa have found that urban populations tended to consume more salt than rural populations [94] and consume fewer portions of vegetables[12]. The rapid pace of urbanisation and economic growth is accelerating the rate of this epidemiologic transition; as such LMICs are at great risk for an explosive growth in the burden of NCDs, including hypertension and type 2 diabetes [87 88].

We found evidence of significant unmet need for hypertension care among urban slum residents. Significant proportion of the urban slum residents were unscreened, undiagnosed, untreated or uncontrolled. This huge unmet need has been documented in previous studies from low- and middle-income settings[95-101]. We also found that control of hypertension among slum residents was poor, such that only one in four slum residents on treatment, had their blood pressure controlled. The poor control of BP noted in our study, despite the fact the one half of those that were unaware of high blood pressure being on antihypertensive medications, needs further exploration. One possible explanation is availability and affordability of the medications and there could be minimal additional contact with a health professional[15]. It has been documented that the control of BP was related to the frequency of follow-up visits[96]. Another possible explanation could be low adherence to prescribed medications, as they may not be able to afford the medications.

As expected, we found that the burden of hypertension increased with the particpants' age, which may be attributed to age-related structural changes in blood vessels which potentially

For peer review only - http://bmjopen.bmj.com/site/about/guidelines.xhtml

cause narrowing of the vascular lumen, and consequently increasing blood pressure, as have been reported in previous studies[102 103]. The association between combined overweight/obesity and hypertension shown in our results exemplify the role of excess body weight in hypertension prevalence, which has been long recognized and consistent across numerous observational and trial data[104-106]. We found evidence of significantly high prevalence of hypertension among smokers compared to the non-smokers. Direct relation of chronic tobacco consumption with hypertension however is not yet well established[107 108] although tobacco consumption has been shown to cause an acute elevation of BP[109].

Study Limitations and Strengths

To the best of our knowledge, this paper is the first systematic reviews that summarises data about prevalence of hypertension and type 2 diabetes among slum residents. Strengths of this study include the use of a predefined and published protocol, a comprehensive search strategy, and involvement of two independent reviewers in the review process. Nevertheless, the findings of this study should be interpreted with caution. Prevalence estimates from different regions and published over the course of 11 years were pooled in this meta-analysis, and as expected, high heterogeneity between studies was found in the meta-analyses. Nonetheless, as affirmed by previous evidence, meta-analyses are the preferred options to narrative syntheses for interpreting the results in a review, even in spite of the presence of a considerable amount of heterogeneity[110]. Heterogeneity appeared to be the norm rather than exception in published meta-analyses of observational studies[111].

In conclusion, the burden of hypertension and type 2 diabetes varied widely between countries and regions and, to some degree, also within countries. In addition, many

For peer review only - http://bmjopen.bmj.com/site/about/guidelines.xhtml

For peer review only

hypertensive individuals are not aware of their condition, not on treatment and control of hypertension is poor. The burden of hypertension and type 2 diabetes was higher among urban residents than their counterparts living in urban slums and rural areas. There is a need for public health strategies to improve the awareness, control and overall management of hypertension and type 2 diabetes in urban areas.

Funding

This research was funded by the National Institute for Health Research (NIHR) Global Health Research Unit on Improving Health in Slums using UK aid from the UK Government to support global health research (Award ID: 16/136/87). The views expressed in this publication are those of the author(s) and not necessarily those of the NIHR or the UK Department of Health and Social Care.

Ethics approval and consent to participate

Not applicable.

Consent for publication: Not applicable.

Data sharing statement: All data relevant to the study are included in the article or uploaded as supplementary information

Competing interests

The authors declare that they have no competing interests.

Authors' contribution

For peer review only - http://bmjopen.bmj.com/site/about/guidelines.xhtml

497 OAU, AAA, OO and RL conceived the study. OAU, AAA and OO collected and analysed initial data. OAU, AAA, OO, JS, PG and RL participated contributed in refining the data analysis. OAU wrote the first manuscript. OAU, AAA, OO, JS, PG and RL contributed to further analysis, interpreting and shaping of the argument of the manuscript and participated in writing the final draft of the manuscript. All the authors read and approved the final manuscript.

For peer review only

For peer review only - http://bmjopen.bmj.com/site/about/guidelines.xhtml

References

1. Global, regional, and national comparative risk assessment of 84 behavioural, environmental and occupational, and metabolic risks or clusters of risks, 1990-2016: a systematic analysis for the Global Burden of Disease Study 2016. *Lancet (London, England)* 2017;390(10100):1345-422. doi: 10.1016/s0140-6736(17)32366-8 [published Online First: 2017/09/19]

2. Bickler SW, Wang A, Amin S, et al. Urbanization in Sub-Saharan Africa: Declining Rates of Chronic and Recurrent Infection and Their Possible Role in the Origins of Non-communicable Diseases. *World journal of surgery* 2018;42(6):1617-28. doi: 10.1007/s00268-017-4389-5 [published Online First: 2017/12/14]

3. Goryakin Y, Rocco L, Suhrcke M. The contribution of urbanization to non-communicable diseases: Evidence from 173 countries from 1980 to 2008. *Economics and human biology* 2017;26:151-63. doi: 10.1016/j.ehb.2017.03.004 [published Online First: 2017/04/15]

4. Khorrami Z, Etemad K, Yarahmadi S, et al. Urbanization and noncommunicable disease (NCD) risk factors: WHO STEPwise Iranian NCD risk factors surveillance in 2011. *Eastern Mediterranean health journal = La revue de sante de la Mediterranee orientale = al-Majallah al-sihhiyah li-sharq al-mutawassit* 2017;23(7):469-79. [published Online First: 2017/08/31]

5. Cohen B. Urban Growth in Developing Countries: A Review of Current Trends and a Caution Regarding Existing Forecasts. *World Development* 2004;32(1):23-51. doi: https://doi.org/10.1016/j.worlddev.2003.04.008

6. Cohen B. Urbanization in developing countries: Current trends, future projections, and key challenges for sustainability. *Technology in Society* 2006;28(1):63-80. doi: https://doi.org/10.1016/j.techsoc.2005.10.005

7. Ezeh A, Oyebode O, Satterthwaite D, et al. The history, geography, and sociology of slums and the health problems of people who live in slums. *Lancet (London, England)* 2017;389(10068):547-58. doi: 10.1016/s0140-6736(16)31650-6 [published Online First: 2016/10/21]

8. Lilford RJ, Oyebode O, Satterthwaite D, et al. Improving the health and welfare of people who live in slums. *Lancet (London, England)* 2017;389(10068):559-70. doi: 10.1016/s0140-6736(16)31848-7 [published Online First: 2016/10/21]

9. Riley LW, Ko AI, Unger A, et al. Slum health: diseases of neglected populations. *BMC Int Health Hum Rights* 2007;7:2. doi: 10.1186/1472-698x-7-2 [published Online First: 2007/03/09]

10. Unger A, Riley LW. Slum health: from understanding to action. *PLoS medicine* 2007;4(10):1561-6. doi: 10.1371/journal.pmed.0040295 [published Online First: 2007/10/26]

11. Lilford R, Kyobutungi C, Ndugwa R, et al. Because space matters: conceptual framework to help distinguish slum from non-slum urban areas. *BMJ Glob Health* 2019;4(2):e001267. doi: 10.1136/bmjgh-2018-001267 [published Online First: 2019/05/30]

For peer review only

For peer review only - http://bmjopen.bmj.com/site/about/guidelines.xhtml

12. Mensah DO, Nunes AR, Bockarie T, et al. Meat, fruit, and vegetable consumption in sub-Saharan Africa: a systematic review and meta-regression analysis. *Nutr Rev* 2020 doi: 10.1093/nutrit/nuaa032 [published Online First: 2020/06/20]

13. Ahmad S, Goel K, Parashar P, et al. A community based cross sectional study on life style & morbidity status of elderly in urban slums of meerut. *Indian Journal of Public Health Research and Development* 2014;5(1):153-57.

14. Anand K, Shah B, Yadav K, et al. Are the urban poor vulnerable to non-communicable diseases? A survey of risk factors for non-communicable diseases in urban slums of Faridabad. *National Medical Journal of India* 2007;20(3):115-20.

15. Banerjee S, Mukherjee TK, Basu S. Prevalence, awareness, and control of hypertension in the slums of Kolkata. *Indian Heart Journal* 2016;68(3):286-94. doi: http://dx.doi.org/10.1016/j.ihj.2015.09.029

16. Daniel OJ, Adejumo OA, Adejumo EN, et al. Prevalence of hypertension among urban slum dwellers in Lagos, Nigeria. *Journal of urban health : bulletin of the New York Academy of Medicine* 2013;90(6):1016-25. doi: 10.1007/s11524-013-9795-x [published Online First: 2013/02/27]

17. Heitzinger K, Montano SM, Hawes SE, et al. A community-based cluster randomized survey of noncommunicable disease and risk factors in a peri-urban shantytown in Lima, Peru. *BMC International Health and Human Rights* 2014;14(1) doi: http://dx.doi.org/10.1186/1472-698X-14-19

18. Nirmala DB, Vijay KM, Sreedhar M. Prevalence of risk factors for Non Communicable Diseases in urban slums of Hyderabad, Telangana *Indian Journal of Basic and Applied Medical Research* 2014;4(1):487-93.

19. Oli N, Vaidya A, Thapa G. Behavioural risk factors of noncommunicable diseases among nepalese urban poor: A descriptive study from a slum area of Kathmandu. *Epidemiology Research International* 2013(pagination) doi: http://dx.doi.org/10.1155/2013/329156

20. Rawal LB, Biswas T, Khandker NN, et al. Non-communicable disease (NCD) risk factors and diabetes among adults living in slum areas of Dhaka, Bangladesh. *PLoS ONE* 2017;12(10) doi: http://dx.doi.org/10.1371/journal.pone.0184967

21. Singh R, Mukherjee M, Kumar R, et al. Study of Risk factors of Coronary Heart Disease in Urban Slums of Patna. *2012* 2012;2(3):-192. doi: 10.3126/nje.v2i3.6902 [published Online First: 2012-10-02]

22. Vigneswari A, Manikandan R, Satyavani K, et al. Prevalence of Risk Factors of Diabetes Among Urban Poor South Indian Population. *The Journal of the Association of Physicians of India* 2015;63(10):32-4. [published Online First: 2016/09/10]

23. UN-Habitat. UN-Habitat Urbanization and development: emerging futures. world cities report 2016. Nairobi Kenya, 2016.

24. Kim SY, Park JE, Lee YJ, et al. Testing a tool for assessing the risk of bias for nonrandomized studies showed moderate reliability and promising validity. *Journal of*

For peer review only - http://bmjopen.bmj.com/site/about/guidelines.xhtml

clinical epidemiology 2013;66(4):408-14. doi: 10.1016/j.jclinepi.2012.09.016 [published Online First: 2013/01/23]

25. DerSimonian R, Laird N. Meta-analysis in clinical trials. *Controlled clinical trials* 1986;7(3):177-88. [published Online First: 1986/09/01]

26. Normand SL. Meta-analysis: formulating, evaluating, combining, and reporting. *Statistics in medicine* 1999;18(3):321-59. [published Online First: 1999/03/10]

27. Higgins JP, Thompson SG. Quantifying heterogeneity in a meta-analysis. *Statistics in medicine* 2002;21(11):1539-58. doi: 10.1002/sim.1186 [published Online First: 2002/07/12]

28. Higgins JP, Thompson SG, Deeks JJ, et al. Measuring inconsistency in meta-analyses. *BMJ (Clinical research ed)* 2003;327(7414):557-60. doi: 10.1136/bmj.327.7414.557 [published Online First: 2003/09/06]

29. Egger M, Davey Smith G, Schneider M, et al. Bias in meta-analysis detected by a simple, graphical test. *BMJ (Clinical research ed)* 1997;315(7109):629-34. [published Online First: 1997/10/06]

30. Clegg LX, Hankey BF, Tiwari R, et al. Estimating average annual per cent change in trend analysis. *Statistics in medicine* 2009;28(29):3670-82. doi: 10.1002/sim.3733 [published Online First: 2009/10/27]

31. Liberati A, Altman DG, Tetzlaff J, et al. The PRISMA statement for reporting systematic reviews and meta-analyses of studies that evaluate healthcare interventions: explanation and elaboration. *BMJ (Clinical research ed)* 2009;339:b2700. doi: 10.1136/bmj.b2700 [published Online First: 2009/07/23]

32. Acharyya T, Kaur P, Murhekar MV. Prevalence of behavioral risk factors, overweight and hypertension in the urban slums of North 24 Parganas District, West Bengal, India, 2010. *Indian journal of public health* 2014;58(3):195-98.

33. Akinwale O, Oyefara J, Adejoh P, et al. The benefits of using a community-engaged research approach to promote a healthy lifestyle in three Nigerian urban slums. *Southern African Journal of Epidemiology and Infection* 2014;29(1):48-50.

34. Ayah R, Joshi MD, Wanjiru R, et al. A population-based survey of prevalence of diabetes and correlates in an urban slum community in Nairobi, Kenya. *BMC public health* 2013;13(371):20. doi: https://dx.doi.org/10.1186/1471-2458-13-371

35. Chakraborty R, Bose K. Comparison of body adiposity indices in predicting blood pressure and hypertension among slum-dwelling men in Kolkata, India. *Malaysian Journal of Nutrition* 2012;18(3):319-28.

36. Chaturvedi S, Pant M, Yadav G. Hypertension in Delhi: prevalence, awareness, treatment and control. *Tropical doctor* 2007;37(3):142-5. doi: 10.1258/004947507781524593 [published Online First: 2007/08/25]

37. Dasappa H, Fathima FN, Prabhakar R, et al. Prevalence of diabetes and pre-diabetes and assessments of their risk factors in urban slums of Bangalore. *Journal of family medicine and*

For peer review only - http://bmjopen.bmj.com/site/about/guidelines.xhtml

primary care 2015;4(3):399-404. doi: 10.4103/2249-4863.161336 [published Online First: 2015/08/20]

38. Deepa M, Pradeepa R, Anjana R, et al. Noncommunicable diseases risk factor surveillance: experience and challenge from India. *Indian journal of community medicine : official publication of Indian Association of Preventive & Social Medicine* 2011;36(Suppl 1):S50-6. doi: 10.4103/0970-0218.94709 [published Online First: 2012/05/26]

39. Edwards JK, Bygrave H, Van den Bergh R, et al. HIV with non-communicable diseases in primary care in Kibera, Nairobi, Kenya: characteristics and outcomes 2010-2013. *Trans R Soc Trop Med Hyg* 2015;109(7):440-6. doi: https://dx.doi.org/10.1093/trstmh/trv038

40. Ezeala-Adikaibe BA, Orjioke C, Ekenze OS, et al. Population-based prevalence of high blood pressure among adults in an urban slum in Enugu, South East Nigeria. *Journal of Human Hypertension* 2016;30(4):285-91. doi: http://dx.doi.org/10.1038/jhh.2015.49

41. Ferreira HDS, Florencio TMTDM, Fragoso MDAC, et al. Hypertension, abdominal obesity and short stature: Aspects of nutritional transition within a shantytown in the city of Maceio (Northeastern Brazil). *Revista de Nutricao* 2005;18(2):209-18.

42. Florencio TT, Ferreira HS, Cavalcante JC, et al. Short stature, obesity and arterial hypertension in a very low income population in North-eastern Brazil. *Nutrition, Metabolism and Cardiovascular Diseases* 2004;14(1):26-33. doi: http://dx.doi.org/10.1016/S0939-4753%2804%2980044-9

43. Haregu TN, Oti S, Ngomi N, et al. Interlinkage among cardio-metabolic disease markers in an urban poor setting in Nairobi, Kenya. *Global health action* 2016;9(pp 30626) doi: http://dx.doi.org/10.3402/gha.v9.30626

44. Huda MN, Alam KS, Harun Ur R. Prevalence of chronic kidney disease and its association with risk factors in disadvantageous population. *International journal of nephrology* 2012;2012:267329. doi: 10.1155/2012/267329 [published Online First: 2012/08/01]

45. Jalil F, Moore SE, Butt NS, et al. Early-life risk factors for adult chronic disease: Follow-up of a cohort born during 1964-1978 in an urban slum of Lahore, Pakistan. *Journal of Health, Population and Nutrition* 2008;26(1):12-21.

46. Joshi A, Puricelli Perin DM, Arora M. Using Portable Health Information Kiosk to assess chronic disease burden in remote settings. *Rural and remote health* 2013;13(2):2279. [published Online First: 2013/03/29]

47. Joshi MD, Ayah R, Njau EK, et al. Prevalence of hypertension and associated cardiovascular risk factors in an urban slum in Nairobi, Kenya: a population-based survey. *BMC public health* 2014;14:1177. doi: 10.1186/1471-2458-14-1177 [published Online First: 2014/11/20]

48. Kar SS, Thakur JS, Jain S, et al. Cardiovascular disease risk management in a primary health care setting of North India. *Indian Heart Journal* 2008;60(1):19-25.

49. Kar SS, Thakur JS, Virdi NK, et al. Risk factors for cardiovascular diseases: Is the social gradient reversing in northern India? *National Medical Journal of India* 2010;23(4):206-09.

For peer review only - http://bmjopen.bmj.com/site/about/guidelines.xhtml

50. Kumari SMV, Humaira B, Sreedhar M. A study on prevalence of hypertension in urban slum field practice area of osmania medical college – Hyderabad *Indian Journal of Basic and Applied Medical Research* 2014;4(1):462-70.

51. Lubree HG, Rege SS, Bhat DS, et al. Body fat and cardiovascular risk factors in Indian men in three geographical locations. *Food and Nutrition Bulletin* 2002;23(3 SUPP):146-49.

52. Marins VM, Almeida RM, Pereira RA, et al. The association between socioeconomic indicators and cardiovascular disease risk factors in Rio de Janeiro, Brazil. *J Biosoc Sci* 2007;39(2):221-9. doi: https://dx.doi.org/10.1017/S0021932006001246

53. Misra A, Pandey RM, Devi JR, et al. High prevalence of diabetes, obesity and dyslipidaemia in urban slum population in northern India. *International journal of obesity and related metabolic disorders : journal of the International Association for the Study of Obesity* 2001;25(11):1722-9. doi: 10.1038/sj.ijo.0801748 [published Online First: 2001/12/26]

54. Olack B, Wabwire-Mangen F, Smeeth L, et al. Risk factors of hypertension among adults aged 35-64 years living in an urban slum Nairobi, Kenya. *BMC public health* 2015;15:1251. doi: 10.1186/s12889-015-2610-8 [published Online First: 2015/12/19]

55. Ongeti K, Ogeng'o J, Pulei A, et al. Blood pressure characteristics among slum dwellers in Kenya. *Global Advanced Research* 2013;2(4):80-85.

56. Oti SO, van de Vijver SJ, Agyemang C, et al. The magnitude of diabetes and its association with obesity in the slums of Nairobi, Kenya: results from a cross-sectional survey. *Tropical medicine & international health : TM & IH* 2013;18(12):1520-30. doi: 10.1111/tmi.12200 [published Online First: 2013/10/15]

57. Patil RS, Gothankar JS. Assessment of risk of type 2 diabetes using the Indian Diabetes Risk Score in an urban slum of Pune, Maharashtra, India: a cross-sectional study. *WHO South-East Asia journal of public health* 2016;5(1):53-61. doi: 10.4103/2224-3151.206555 [published Online First: 2016/04/01]

58. Rahim MA, Vaaler S, Keramat Ali SM, et al. Prevalence of type 2 diabetes in urban slums of Dhaka, Bangladesh. *Bangladesh Medical Research Council Bulletin* 2004;30(2):60-70.

59. Sayeed MA, Mahtab H, Khanam PA, et al. Prevalence of diabetes and impaired fasting glucose in urban population of Bangladesh. *Bangladesh Medical Research Council Bulletin* 2007;33(1):1-12.

60. Singh AK, Mani K, Krishnan A, et al. Prevalence, awareness, treatment and control of diabetes among elderly persons in an urban slum of delhi. *Indian journal of community medicine : official publication of Indian Association of Preventive & Social Medicine* 2012;37(4):236-9. doi: 10.4103/0970-0218.103472 [published Online First: 2013/01/08]

61. Sinha P, Taneja DK, Singh NP, et al. Seasonal variation in prevalence of hypertension: Implications for interpretation. *Indian journal of public health* 2010;54(1):7-10.

For peer review only - http://bmjopen.bmj.com/site/about/guidelines.xhtml

62. Sitthi-Amorn C, Chandraprasert S, Bunnag SC, et al. The prevalence and risk factors of hypertension in Klong Toey Slum and Klong Toey government apartment houses. *International Journal of Epidemiology* 1989;18(1):89-94.

63. Snyder RE, Lopes LA, Tavares LCC, et al. O Dia de Dona Maria-Using technology and community based participatory research to improve healthcare delivery in a Brazilian urban slum. *Annals of Global Health* 2016;Conference:7th Annual CUGH Conference: Bridging to a Sustainable Future in Global Health. United States. 82 (3) (pp 599).

64. Sowemimo I, Ajayi I, Akpa O, et al. Prevalence of hypertension and associated factors among residents of Ibadan-north local government area of Oyo State, Nigeria. *Journal of Hypertension* 2015;Conference:25th European Meeting on Hypertension and Cardiovascular Protection. doi: http://dx.doi.org/10.1097/01.hjh.0000467432.10548.8c

65. Sunita M, Singh AK, Rogye A, et al. Prevalence of Diabetic Retinopathy in Urban Slums: The Aditya Jyot Diabetic Retinopathy in Urban Mumbai Slums Study-Report 2. *Ophthalmic Epidemiology* 2017;24(5):303-10. doi: http://dx.doi.org/10.1080/09286586.2017.1290258

66. Unger A, Felzemburgh RD, Snyder RE, et al. Hypertension in a Brazilian urban slum population. *J Urban Health* 2015;92(3):446-59. doi: https://dx.doi.org/10.1007/s11524-015-9956-1

67. Uthakalla VK, Kishore Kumar KJ, Jena SK, et al. Prevalence study of overweight/obesity among adults (20-60yrs) of urban field practice area of osmania medical college, Hyderabad. *Indian Journal of Public Health Research and Development* 2012;3(3):250-53.

68. van de Vijver S, Oti S, Tervaert TC, et al. Introducing a model of cardiovascular prevention in Nairobi's slums by integrating a public health and private-sector approach: the SCALE-UP study. *Global health action* 2013;6(pp 22510)

69. Vikram NK, Pandey RM, Misra A, et al. Non-obese (body mass index < 25 kg/m²) Asian Indians with normal waist circumference have high cardiovascular risk. *Nutrition* 2003;19(6):503-09. doi: http://dx.doi.org/10.1016/S0899-9007%2802%2901083-3

70. Wasir JS, Misra A, Vikram NK, et al. C-reactive protein, obesity, and insulin resistance in postmenopausal women in urban slums of North India. *Diabetes and Metabolic Syndrome: Clinical Research and Reviews* 2007;1(2):83-89. doi: http://dx.doi.org/10.1016/j.dsx.2007.02.001

71. Yajnik CS, Joglekar CV, Lubree HG, et al. Adiposity, inflammation and hyperglycaemia in rural and urban Indian men: Coronary Risk of Insulin Sensitivity in Indian Subjects (CRISIS) Study. *Diabetologia* 2008;51(1):39-46. doi: https://dx.doi.org/10.1007/s00125-007-0847-1

72. Hypertension detection, treatment and control rates in urban slum population in bangladesh. *Journal of Hypertension* 2018;Conference:27th Scientific Meeting of the International Society of Hypertension, ISH 2018. China. 36 (Supplement 3) (pp e337-e338).

73. Assessment of risk factors of hypertension among adults residing in urban slum of Delhi. *Asian Journal of Pharmaceutical and Clinical Research* 2018;11(1):405-07. doi: http://dx.doi.org/10.22159/ajpcr.2018.v11i1.23755

For peer review only - http://bmjopen.bmj.com/site/about/guidelines.xhtml

74. Prevalence of hypertension among elderly residing in slums of west Delhi. *Asian Journal of Pharmaceutical and Clinical Research* 2018;11(4):337-39. doi: http://dx.doi.org/10.22159/ajpcr.2018.v11i4.23414

75. Abhinav Jain BKAMPSMSKSACSJ. A Study of Prevalence of Diabetes Mellitus and its Risk Factors in the Urban Slum Population of Gurugram. *Indian Journal of Public Health Research & Development* 2019;10(4):141-45. doi: 10.37506/ijphrd.v10i4.6613

76. Bawah AT, Abaka-Yawson A, Seini MM, et al. Prevalence of diabetes among homeless and slum dwellers in Accra, Ghana: a survey study. *BMC Res Notes* 2019;12(1):572. doi: 10.1186/s13104-019-4613-5 [published Online First: 2019/09/13]

77. Gadallah M, Megid SA, Mohsen A, et al. Hypertension and associated cardiovascular risk factors among urban slum dwellers in Egypt: a population-based survey. *Eastern Mediterranean health journal = La revue de sante de la Mediterranee orientale = al-Majallah al-sihhiyah li-sharq al-mutawassit* 2018;24(5):435-42. doi: 10.26719/2018.24.5.435 [published Online First: 2018/07/26]

78. George CE, Norman G, Wadugodapitya A, et al. Health issues in a Bangalore slum: findings from a household survey using a mobile screening toolkit in Devarajeevanahalli. *BMC public health* 2019;19(1):456. doi: 10.1186/s12889-019-6756-7 [published Online First: 2019/05/01]

79. Tymejczyk O, McNairy ML, Petion JS, et al. Hypertension prevalence and risk factors among residents of four slum communities: population-representative findings from Port-au-Prince, Haiti. *Journal of hypertension* 2019;37(4):685-95. doi: 10.1097/hjh.0000000000001966 [published Online First: 2019/03/01]

80. Vusirikala A, Wekesah F, Kyobutungi C, et al. Assessment of cardiovascular risk in a slum population in Kenya: use of World Health Organisation/International Society of Hypertension (WHO/ISH) risk prediction charts - secondary analyses of a household survey. *BMJ open* 2019;9(9):e029304. doi: 10.1136/bmjopen-2019-029304 [published Online First: 2019/09/07]

81. Addo J, Smeeth L, Leon DA. Hypertension in sub-saharan Africa: a systematic review. *Hypertension (Dallas, Tex : 1979)* 2007;50(6):1012-8. doi: 10.1161/hypertensionaha.107.093336 [published Online First: 2007/10/24]

82. Pereira M, Lunet N, Azevedo A, et al. Differences in prevalence, awareness, treatment and control of hypertension between developing and developed countries. *Journal of hypertension* 2009;27(5):963-75. [published Online First: 2009/04/30]

83. Gupta R, al-Odat NA, Gupta VP. Hypertension epidemiology in India: meta-analysis of 50 year prevalence rates and blood pressure trends. *J Hum Hypertens* 1996;10(7):465-72. [published Online First: 1996/07/01]

84. Mahmood SE, Prakash D, Srivastava JP, et al. Prevalence of Hypertension Amongst Adult Patients Attending Out Patient Department of Urban Health Training Centre, Department of Community Medicine, Era's Lucknow Medical College and Hospital, Lucknow. *Journal of clinical and diagnostic research : JCDR* 2013;7(4):652-6. doi: 10.7860/jcdr/2013/4707.2874 [published Online First: 2013/06/05]

For peer review only

For peer review only - http://bmjopen.bmj.com/site/about/guidelines.xhtml

85. Amuna P, Zotor FB. Epidemiological and nutrition transition in developing countries: impact on human health and development. *The Proceedings of the Nutrition Society* 2008;67(1):82-90. doi: 10.1017/s0029665108006058 [published Online First: 2008/02/01]

86. Kroll M, Bharucha E, Kraas F. Does rapid urbanization aggravate health disparities? Reflections on the epidemiological transition in Pune, India. *Glob Health Action* 2014;7:23447. doi: 10.3402/gha.v7.23447 [published Online First: 2014/09/13]

87. Angkurawaranon C, Jiraporncharoen W, Chenthanakij B, et al. Urbanization and non-communicable disease in Southeast Asia: a review of current evidence. *Public health* 2014;128(10):886-95. doi: 10.1016/j.puhe.2014.08.003 [published Online First: 2014/11/05]

88. Cheema A, Adeloye D, Sidhu S, et al. Urbanization and prevalence of type 2 diabetes in Southern Asia: A systematic analysis. *Journal of global health* 2014;4(1):010404. doi: 10.7189/jogh.04.010404 [published Online First: 2014/07/01]

89. Low WY, Lee YK, Samy AL. Non-communicable diseases in the Asia-Pacific region: Prevalence, risk factors and community-based prevention. *International journal of occupational medicine and environmental health* 2015;28(1):20-6. doi: 10.2478/s13382-014-0326-0 [published Online First: 2015/07/15]

90. Phipps ME, Chan KK, Naidu R, et al. Cardio-metabolic health risks in indigenous populations of Southeast Asia and the influence of urbanization. *BMC public health* 2015;15:47. doi: 10.1186/s12889-015-1384-3 [published Online First: 2015/02/01]

91. Siegel KR, Patel SA, Ali MK. Non-communicable diseases in South Asia: contemporary perspectives. *British medical bulletin* 2014;111(1):31-44. doi: 10.1093/bmb/ldu018 [published Online First: 2014/09/06]

92. Streatfield PK, Khan WA, Bhuiya A, et al. Adult non-communicable disease mortality in Africa and Asia: evidence from INDEPTH Health and Demographic Surveillance System sites. *Glob Health Action* 2014;7:25365. doi: 10.3402/gha.v7.25365 [published Online First: 2014/11/08]

93. Gaziano TA, Bitton A, Anand S, et al. Growing epidemic of coronary heart disease in low- and middle-income countries. *Current problems in cardiology* 2010;35(2):72-115. doi: 10.1016/j.cpcardiol.2009.10.002 [published Online First: 2010/01/30]

94. Oyebode O, Oti S, Chen YF, et al. Salt intakes in sub-Saharan Africa: a systematic review and meta-regression. *Population health metrics* 2016;14:1. doi: 10.1186/s12963-015-0068-7 [published Online First: 2016/01/14]

95. Adeloye D, Basquill C. Estimating the prevalence and awareness rates of hypertension in Africa: a systematic analysis. *PLoS One* 2014;9(8):e104300. doi: 10.1371/journal.pone.0104300 [published Online First: 2014/08/05]

96. Macia E, Duboz P, Gueye L. Prevalence, awareness, treatment and control of hypertension among adults 50 years and older in Dakar, Senegal. *Cardiovascular journal of Africa* 2012;23(5):265-9. doi: 10.5830/cvja-2011-039 [published Online First: 2011/10/18]

For peer review only - http://bmjopen.bmj.com/site/about/guidelines.xhtml

97. Mohan V, Deepa M, Farooq S, et al. Prevalence, awareness and control of hypertension in Chennai--The Chennai Urban Rural Epidemiology Study (CURES-52). *The Journal of the Association of Physicians of India* 2007;55:326-32. [published Online First: 2007/09/12]

98. Pilav A, Doder V, Brankovic S. Awareness, Treatment, and control of Hypertension among Adult Population in the Federation of Bosnia and Herzegovina over the Past Decade. *Journal of public health research* 2014;3(3):323. doi: 10.4081/jphr.2014.323 [published Online First: 2015/01/02]

99. Supiyev A, Kossumov A, Utepova L, et al. Prevalence, awareness, treatment and control of arterial hypertension in Astana, Kazakhstan. A cross-sectional study. *Public health* 2015;129(7):948-53. doi: 10.1016/j.puhe.2015.02.020 [published Online First: 2015/03/31]

100. Tailakh A, Evangelista LS, Mentes JC, et al. Hypertension prevalence, awareness, and control in Arab countries: a systematic review. *Nursing & health sciences* 2014;16(1):126-30. doi: 10.1111/nhs.12060 [published Online First: 2013/10/15]

101. Yazdanpanah L, Shahbazian H, Shahbazian H, et al. Prevalence, awareness and risk factors of hypertension in southwest of Iran. *Journal of renal injury prevention* 2015;4(2):51-6. doi: 10.12861/jrip.2015.11 [published Online First: 2015/06/11]

102. Landahl S, Bengtsson C, Sigurdsson JA, et al. Age-related changes in blood pressure. *Hypertension (Dallas, Tex : 1979)* 1986;8(11):1044-9. [published Online First: 1986/11/01]

103. Pinto E. Blood pressure and ageing. *Postgraduate medical journal* 2007;83(976):109-14. doi: 10.1136/pgmj.2006.048371 [published Online First: 2007/02/20]

104. Dyer AR, Elliott P, Shipley M. Body mass index versus height and weight in relation to blood pressure. Findings for the 10,079 persons in the INTERSALT Study. *American journal of epidemiology* 1990;131(4):589-96. [published Online First: 1990/04/01]

105. Folsom AR, Kushi LH, Anderson KE, et al. Associations of general and abdominal obesity with multiple health outcomes in older women: the Iowa Women's Health Study. *Archives of internal medicine* 2000;160(14):2117-28. [published Online First: 2000/07/25]

106. Hu G, Barengo NC, Tuomilehto J, et al. Relationship of physical activity and body mass index to the risk of hypertension: a prospective study in Finland. *Hypertension (Dallas, Tex : 1979)* 2004;43(1):25-30. doi: 10.1161/01.Hyp.0000107400.72456.19 [published Online First: 2003/12/06]

107. Abtahi F, Kianpour Z, Zibaeenezhad MJ. Correlation between cigarette smoking and blood pressure and pulse pressure among teachers residing in Shiraz, Southern Iran. *Iran Cardiovasc Res J* 2011;5:97-102.

108. Primatesta P, Falaschetti E, Gupta S, et al. Association between smoking and blood pressure: evidence from the health survey for England. *Hypertension (Dallas, Tex : 1979)* 2001;37(2):187-93. [published Online First: 2001/03/07]

109. Westman EC. Does smokeless tobacco cause hypertension? *Southern medical journal* 1995;88(7):716-20. [published Online First: 1995/07/01]

For peer review only - http://bmjopen.bmj.com/site/about/guidelines.xhtml

110. Ioannidis JP, Patsopoulos NA, Rothstein HR. Reasons or excuses for avoiding meta-analysis in forest plots. *BMJ (Clinical research ed)* 2008;336(7658):1413-5. doi: 10.1136/bmj.a117 [published Online First: 2008/06/21]

111. Higgins JP. Commentary: Heterogeneity in meta-analysis should be expected and appropriately quantified. *Int J Epidemiol* 2008;37(5):1158-60. doi: 10.1093/ije/dyn204 [published Online First: 2008/10/04]

For peer review only

For peer review only - http://bmjopen.bmj.com/site/about/guidelines.xhtml

TABLES

Table 1: Pooled prevalence by difference subgroup

Subgroup		Hypertension				Type 2 Diabetes		
		n	%	I^2		n	%	I^2
Sample size	Smaller studies (<1000)	27	25.9 (21.6 to 30.6)	97.1		15	11.0 (8.2 to 14.2)	93.9
Sample size	Larger studies (1000+)	17	21.4 (17.2 ro 26.1)	99.6		15	7.8 (5.1 to 11.1)	99.4
Study precision	Imprecise studies	8	33.4 (25.7 to 41.7)	91.2		1	25.2 (17.3 to 34.2)	-
Study precision	Precise studies	36	22.3 (18.9 to 25.9)	99.2		29	8.9 (6.9 to 11.2)	98.9
Publication year	2001 to 2005	5	15.6 (9.0 to 23.8)	94.7		4	8.2 (6.7 to 9.8)	53.6
Publication year	2006 to 2010	6	28.6 (18.9 to 39.4)	98.7		4	6.3 (3.3 to 10.3)	90.6
Publication year	2011 to 2020	33	24.7 (21.0 to 28.6)	99.2		22	10.2 (7.4 to 13.4)	99.2
Region	South Asia	27	25.1 (20.7 to 29.8)	98.9		19	11.9 (9.1 to 15.1)	97.6
Region	Sub-Saharan Africa	10	24.4 (17.7 to 31.9)	99.2		8	4.5 (2.4 to 7.2)	98.8
Region	Latin America and Caribbean	6	18.3 (13.4 to 23.9)	97.1		1	10.2 (8.1 to 12.3)	-
Region	Middle East and North Africa	1	31.2 (28.4 to 34.1)	-		1	8.8 (7.1 to 10.6)	-
Region	East Asia and Pacific	-	-	-		1	7.9 (6.3 to 9.7)	
Income category	Lower Middle Income	36	25.2 (21.2 to 29.4)	99.1		28	9.3 (7.0 to 11.92)	98.9
Income category	Upper Middle Income	5	17.9 (12.1 to 24.6)	97.6		2	9.0 (6.9 to 11.3)	62
Income category	Low Income	2	24.0 (16.9 to 32.0)	92.2				
Sex	Male	24	22.5 (16.0 to 29.7)	99.2		11	8.1 (5.1 to 11.6)	97.6
Sex	Female	24	23.2 (18.6 to 28.1)	98.7		11	7.3 (4.6 to 10.6)	97.5
Age	Young adult	8	15.7 (10.1 to 22.1)	97.8		2	2.1 (0.3 to 5.4)	96.7
Age	Middle-age adult	9	35.0 (25.0 to 45.6)	99.2		2	5.6 (4.5 to 6.8)	0
Age	Older adult	9	49.6 (36.7 to 62.6)	98.3		2	9.1 (7.0 to 11.4)	0
Body mass index	Under weight	5	21.8 (11.4 to 34.4)	87.3				
Body mass index	Normal weight	6	21.9 (11.8 to 34.2)	98.6		2	2.3 (1.8 to 2.8)	0
Body mass index	Overweight	6	32.9 (21.2 to 45.8)	97.4		2	4.2 (1.2 to 8.8)	50
Body mass index	Obese	6	45.4 (34.5 to 56.6)	93.3		2	6.4 (4.0 to 9.3)	0
Education Status	Never studied	7	39.1 (27.5 to 51.3)	98		1	5.1 (3.0 to 7.8)	-
Education Status	Less than primary	4	18.3 (13.9 to 23.1)	87.1		1	4.6 (3.4 to 6.1)	-
Education Status	Primary	6	24.8 (12.0 to 40.4)	99.4		1	4.4 (3.6 to 5.2)	-
Education Status	Secondary or higher	7	22.4 (11.1 to 36.2)	99.3		1	4.1 (3.2 to 5.2)	-
Income	Poorest	5	20.9 (10.4 to 33.8)	98.9				
Income	Middle	5	25.3 (10.6 to 43.8)	99.5				
Income	Least poor	5	29.2 (13.1 to 48.5)	98.3				
Smoking status	Yes	5	38.0 (19.1 to 59.0)	99.1				
Smoking status	No	5	30.5 (17.6 to 45.2)	99.6				
Alcohol consumption	Yes	3	26.5 (18.0 to 35.9)	83.4				
Alcohol consumption	No	3	29.1 (9.3 to 54.3)	99.7				
Physically active	Yes	3	28.8 (11.1 to 50.8)	99.6				
Physically active	No	3	30.8 (7.7 to 60.9)	98.4				
Treatment cascade	Aware of HBP	12	33.6 (19.1 to 50.0)	99.7				
Treatment cascade	On treatment	9	51.9 (35.2 to 68.3)	98.6				
Treatment cascade	BP controlled	8	25.9 (18.4 to 34.3)	87.8				

* World Bank Country Income Groups, 2018

For peer review only

For peer review only - http://bmjopen.bmj.com/site/about/guidelines.xhtml

Participants were divided into age groups that, broadly defined, covered young adulthood (18 to 35 years), middle age (36 to 55 years), and older adulthood (56 years and older).

Underweight - BMI under 18.5 kg/m^2

Normal weight - BMI greater than or equal to 18.5 to 24.9 kg/m^2

Overweight – BMI greater than or equal to 25 to 29.9 kg/m^2

Obesity – BMI greater than or equal to 30 kg/m^2

Physical activity as defined by the authors

Alcohol consumption as defined by authors

Smoking status as defined by authors

Income status as reported by authors

For peer review only

For peer review only - http://bmjopen.bmj.com/site/about/guidelines.xhtml

FIGURE LEGENDS

Figure 1: Hypertension prevalence estimates among slum residents and 95% confidence intervals from individual studies and pooled data

Figure 2: Type 2 diabetes mellitus prevalence estimates among slum residents and 95% confidence intervals from individual studies and pooled data

Figure 3: Secular trends in hypertension prevalence estimates among slum residents across different regions

Figure 4: Secular trends in Type 2 diabetes mellitus prevalence estimates among slum residents across different regions

Figure 5: Hypertension prevalence estimates by place of residence: urban versus rural versus slum

Figure 6: Type 2 diabetes mellitus prevalence estimates by place of residence: urban versus rural versus slum

For peer review only - http://bmjopen.bmj.com/site/about/guidelines.xhtml

ONLINE ONLY SUPPLEMENTS

eFigure 1: Study selection and inclusion flow chart

Box 1: Study selection and inclusion flow chart

eTable 1: List of Excluded Studies

eTable 2: Characteristics of included studies

eTable 3: Risk of bias of included studies

Annex 1: MEDLINE Search Strategy

Annex 2: PRISMA Checklist

For peer review only

For peer review only - http://bmjopen.bmj.com/site/about/guidelines.xhtml

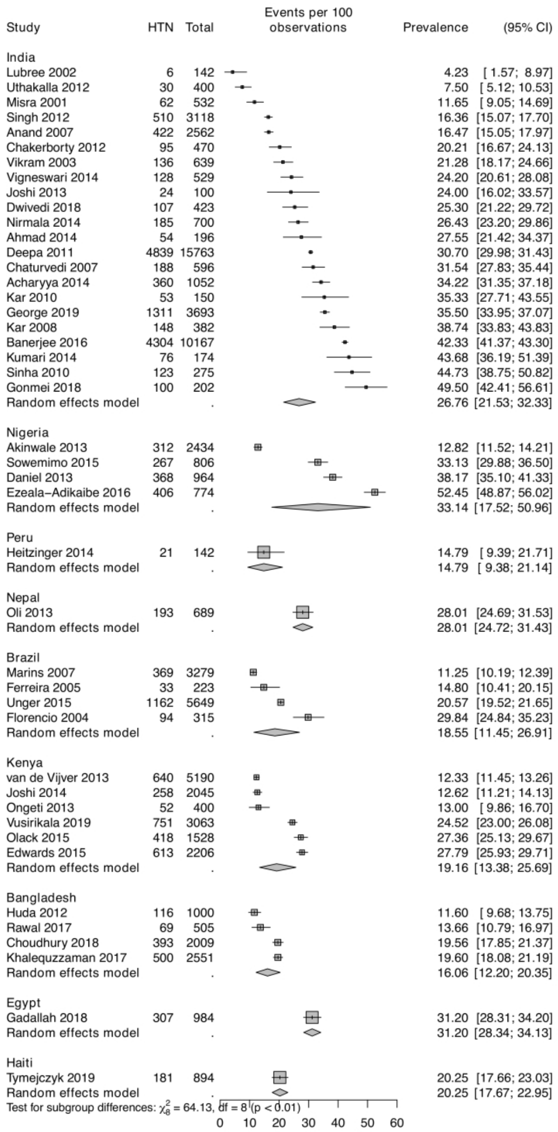

Figure 1

228x406mm (300 x 300 DPI)

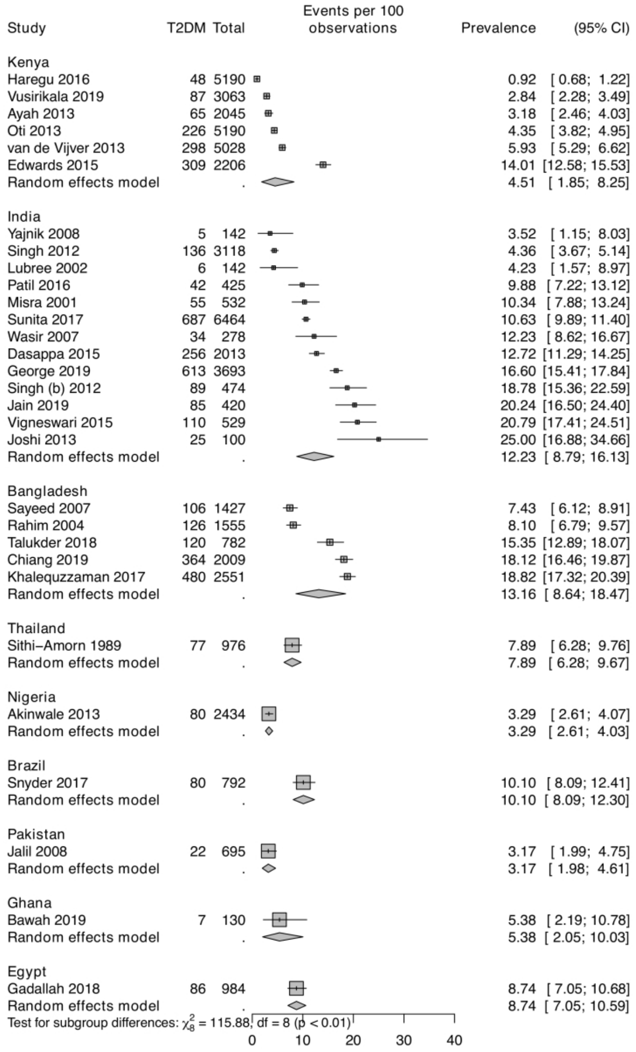

Figure 2

228x355mm (300 x 300 DPI)

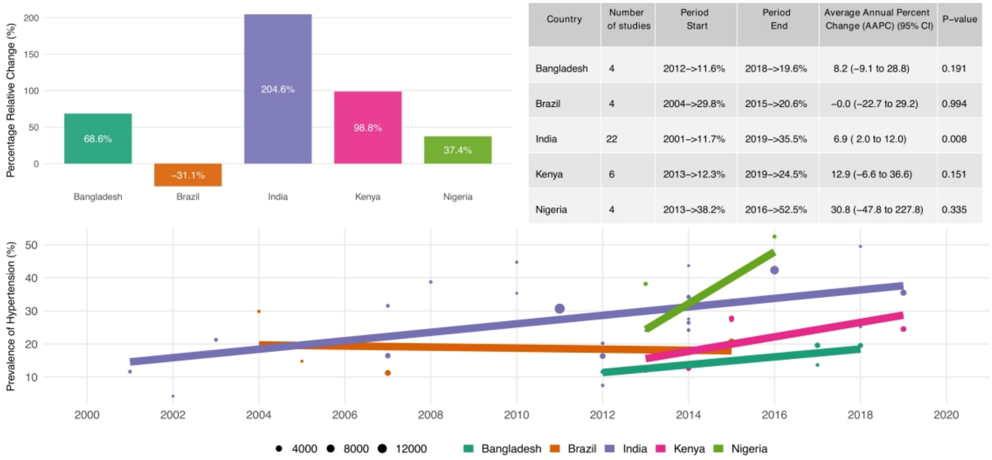

Country	Number of studies	Period Start	Period End	Average Annual Percent Change (AAPC) (95% CI)	P−value
Bangladesh	4	2012−>11.6%	2018−>19.6%	8.2 (−9.1 to 28.8)	0.191
Brazil	4	2004−>29.8%	2015−>20.6%	−0.0 (−22.7 to 29.2)	0.994
India	22	2001−>11.7%	2019−>35.5%	6.9 (2.0 to 12.0)	0.008
Kenya	6	2013−>12.3%	2019−>24.5%	12.9 (−6.6 to 36.6)	0.151
Nigeria	4	2013−>38.2%	2016−>52.5%	30.8 (−47.8 to 227.8)	0.335

Figure 3

496x229mm (300 x 300 DPI)

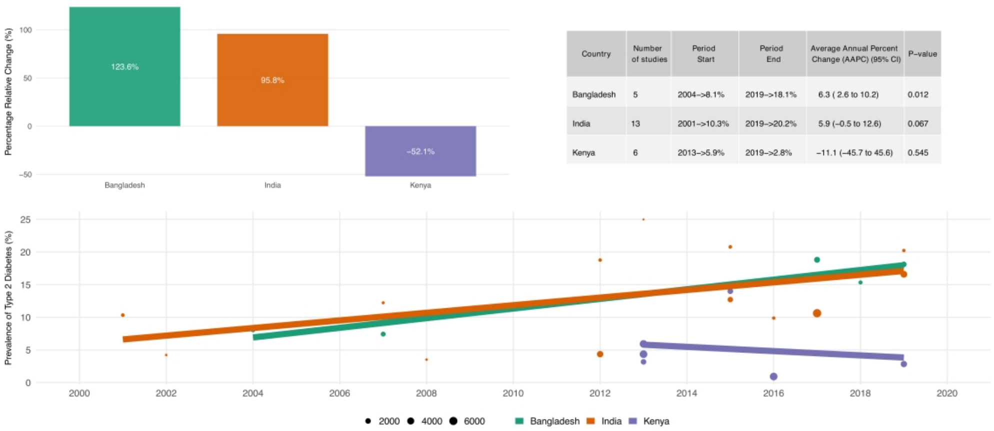

Country	Number of studies	Period Start	Period End	Average Annual Percent Change (AAPC) (95% CI)	P−value
Bangladesh	5	2004−>8.1%	2019−>18.1%	6.3 (2.6 to 10.2)	0.012
India	13	2001−>10.3%	2019−>20.2%	5.9 (−0.5 to 12.6)	0.067
Kenya	6	2013−>5.9%	2019−>2.8%	−11.1 (−45.7 to 45.6)	0.545

Figure 4

602x263mm (300 x 300 DPI)

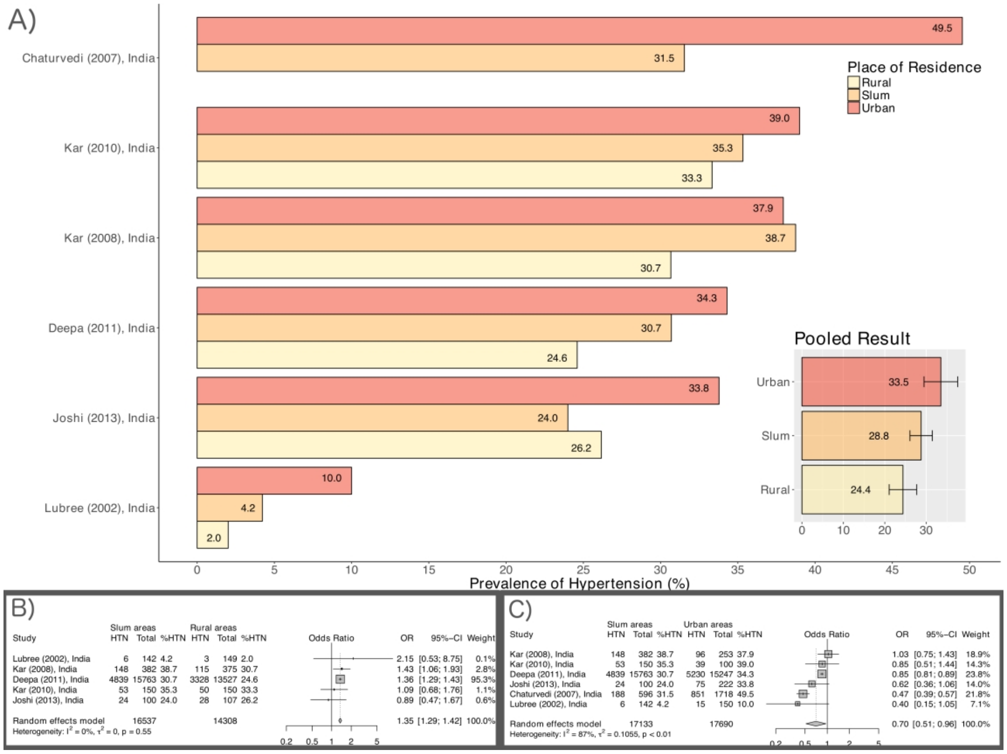

Figure 5

478x357mm (300 x 300 DPI)

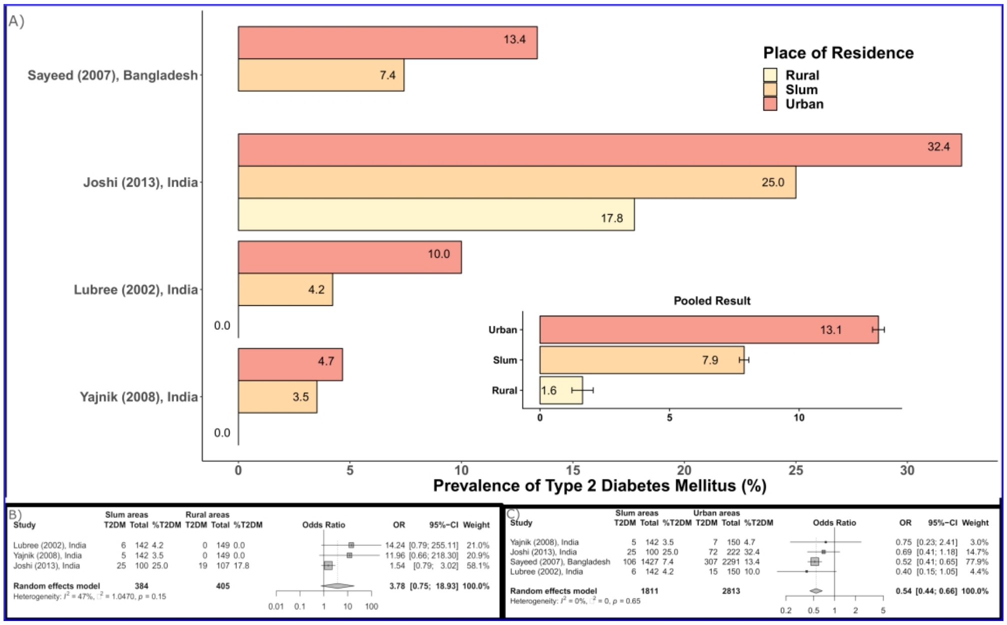

Figure 6

425x261mm (300 x 300 DPI)

Supplementary Digital Content

Table of Contents

eFigure 1: Study selection and inclusion flow chart....1

Box 1: Study selection and inclusion flow chart....2

eTable 1: List of Excluded Studies....3

eTable 2: Characteristics of included studies....11

eTable 3: Risk of bias of included studies....13

Annex 1: MEDLINE Search Strategy....15

For peer review only

eFigure 1: Study selection and inclusion flow chart

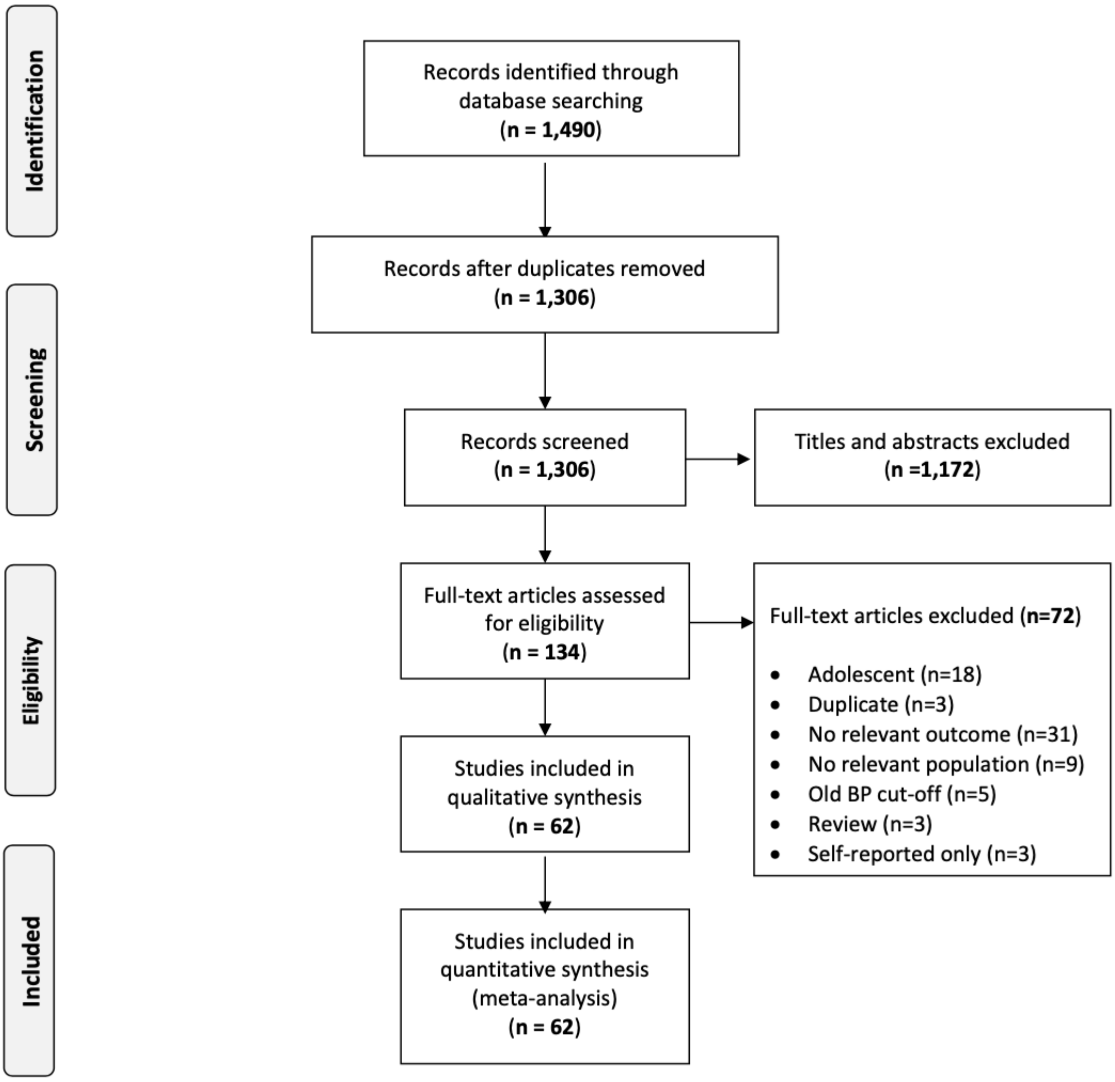

For peer review only - http://bmjopen.bmj.com/site/about/guidelines.xhtml

Box 1: Study selection and inclusion flow chart

Domain	Details	Risk of bias
Selection of participants	Selection bias caused by the inadequate selection of participants	- Low<br>- High<br>- Unclear
Confounding variables	Selection bias caused by the inadequate confirmation and consideration of confounding variable	- Low<br>- High<br>- Unclear
Measurement of exposure	Performance bias caused by the inadequate measurement of exposure	- Low<br>- High<br>- Unclear
Blinding of outcome assessments	Detection bias caused by the inadequate blinding of outcome assessments	- Low<br>- High<br>- Unclear
Incomplete outcome data	Attrition bias caused by the inadequate handling of incomplete outcome data	- Low<br>- High<br>- Unclear
Selective outcome reporting	Reporting bias caused by the selective reporting of outcomes	- Low<br>- High<br>- Unclear

For peer review only

For peer review only - http://bmjopen.bmj.com/site/about/guidelines.xhtml

eTable 1: List of Excluded Studies

s/n	Study	Reason
1	Maiti 2016[1]	Adolescent
2	Khopkar 2015[2]	Adolescent
3	Paul 2013[3]	Adolescent
4	Kamath 2012[4]	Adolescent
5	Simsek 2012[5]	Adolescent
6	Saha 2011[6]	Adolescent
7	Oria 2010[7]	Adolescent
8	Saha 2008[8]	Adolescent
9	Saha 2008[9]	Adolescent
10	Sesso 2004[10]	Adolescent
11	Fernandes 2003[11]	Adolescent
12	Zeelie 2010[12]	Adolescent
13	Soudrassanane 2008[13]	Adolescent
14	Werner 2015[14]	Duplicate
15	van de Vijver 2016[15]	Duplicate
16	Haregu 2016[16]	Duplicate
17	Ezenwaka 1997[17]	Old BP cut-off
18	Suriyawongpaisal 1993[18]	Old BP cut-off
19	Suriyawongpaisal 1991[19]	Old BP cut-off
20	Sitthi-Amornn 1989[20]	Old BP cut-off
21	Bunnag 1990[21]	Old BP cut-off
22	E. Sharmin Trisha 2016[22]	No relevant outcome
23	Bhandari 2015[23]	No relevant outcome
24	Oti 2014[24]	No relevant outcome
25	Hiremath 2014[25]	No relevant outcome
26	Joshi 2013[26]	No relevant outcome
27	van de Vijver 2013[27]	No relevant outcome
28	Itrat 2011[28]	No relevant outcome
29	Ahmed 2011[29]	No relevant outcome
30	Haregu 2015[30]	No relevant outcome
31	van de Vijver 2015[31]	No relevant outcome
32	Kohli 2016[32]	No relevant outcome
33	Mudgapalli 2016[33]	No relevant population
34	Natarajan 2014[34]	No relevant population
35	Kumaramanickavel 2014[35]	No relevant population
36	Kumaramanickavel 2015[36]	No relevant population
37	Hulzebosch 2015[37]	No relevant population
38	Madhu 2016[38]	No relevant population
39	Mugure 2014[39]	No relevant population
40	Mukhopadhyay 2012[40]	No relevant population
41	Khan 2010[41]	No relevant population
42	Etyang 2013[42]	Review
43	Dhar 2014[43]	Review
44	Bhargava 1991[44]	Review
46	Kien 2015[45]	Self-reported only
47	Sur 2007[46]	Self-reported only
48	Thakur 2013[47]	Self-reported only
49	Ahmedani 2019[48]	No relevant outcome
50	Ashe 2019[49]	No relevant outcome
51	Asiki 2018[50]	No relevant outcome
52	Bagdey 2019[51]	No relevant outcome
53	Cope 2020[52]	No relevant outcome
54	De Silva 2018[53]	No relevant outcome
55	Kapwata 2018[54]	No relevant outcome
56	Kawazoe 2018 [55]	No relevant outcome

For peer review only - http://bmjopen.bmj.com/site/about/guidelines.xhtml

57	Khanam 2019[56]	No relevant outcome
58	Kolak 2018[57]	No relevant outcome
59	Korn 2018[58]	No relevant outcome
60	Kotian 2019[59]	No relevant outcome
61	Kumar 2018[60]	No relevant outcome
62	Ma 2018[61]	No relevant outcome
63	Maharana 2019[62]	No relevant outcome
64	Nagarkar 2018[63]	No relevant outcome
65	Narendran 2018[64]	No relevant outcome
66	Rajapakshe 2018[65]	No relevant outcome
67	Sarkar 2019[66]	No relevant outcome
68	Scazufca 2019[67]	No relevant outcome
69	Wang 2018[68]	No relevant outcome
70	Wekasah 2020[69]	No relevant outcome
71	Wilson 2020[70]	No relevant outcome
72	Yadav 2018[71]	No relevant outcome
73	Zhang 2019[72]	No relevant outcome

For peer review only

List of excluded studies

1. Maiti M, Bandyopadhyay L. Variation in blood pressure among adolescent schoolchildren in an urban slum of Kolkata, West Bengal. *Postgraduate Medical Journal (no pagination), 2016* 2016;Date of Publication:July 25. doi: http://dx.doi.org/10.1136/postgradmedj-2016-134227
2. Khopkar SA, Virtanen SM, Kulathinal S. Mental health, anthropometry and blood pressure among adolescents living in slums of Nashik, India. *Tanzania Journal of Health Research* 2015;17(4) doi: http://dx.doi.org/10.4314/thrb.v17i4.6
3. Paul B, Saha I, Mukherjee A. Adolescent Hypertension and Family History. *Pakistan Paediatric Journal* 2013;37(3):177-79.
4. Kamath N, Goud BR, Phadke KD, et al. Use of oscillometric devices for the measurement of blood pressure-comparison with the gold standard. *Indian Journal of Pediatrics* 2012;79(9):1230-32. doi: http://dx.doi.org/10.1007/s12098-011-0600-0
5. Simsek E, Selver B, Dallar Y, et al. Obesity epidemiology in children living in the lower socio-economic status. *Hormone Research in Paediatrics* 2012;Conference:51st Annual Meeting of the European Society for Paediatric Endocrinology. doi: http://dx.doi.org/10.1159/000343184
6. Saha I, Paul B, Mukherjee A, et al. Validity of the WHO criteria for adolescent hypertension. *East African journal of public health* 2011;8(2):135-37.
7. Oria RB, Patrick PD, Oria MOB, et al. ApoE polymorphisms and diarrheal outcomes in Brazilian shanty town children. *Brazilian Journal of Medical and Biological Research* 2010;43(3):249-56.
8. Saha I, Paul B, Dasgupta A. Prevalence of hypertension and variation of blood pressure with age among adolescents in Chetla, India. *Tanzania journal of health research* 2008;10(2):108-11.
9. Saha I, Paul B, Dasgupta A, et al. Variations of adolescent blood pressure by multifactorial analysis in an urban slum of Kolkata. *Journal of the Indian Medical Association* 2008;106(9)
10. Sesso R, Barreto GP, Neves J, et al. Malnutrition is associated with increased blood pressure in childhood. *Nephron Clinical Practice* 2004;97(2):c61-c66. doi: http://dx.doi.org/10.1159/000078402
11. Fernandes MTB, Sesso R, Martins PA, et al. Increased blood pressure in adolescents of low socioeconomic status with short stature. *Pediatric Nephrology* 2003;18(5):435-39.
12. Zeelie A, Moss SJ, Kruger HS. The relationship between body composition and selected metabolic syndrome markers in black adolescents in South Africa: the PLAY study. *Nutrition* 2010;26(11-12):1059-64. doi: 10.1016/j.nut.2010.03.001 [published Online First: 2010/06/15]
13. Soudarssanane M, Mathanraj S, Sumanth M, et al. Tracking of blood pressure among adolescents and young adults in an urban slum of puducherry. *Indian journal of community medicine : official publication of Indian Association of Preventive & Social Medicine* 2008;33(2):107-12. doi: 10.4103/0970-0218.40879 [published Online First: 2008/04/01]
14. Werner ME, van de Vijver S, Adhiambo M, et al. Results of a hypertension and diabetes treatment program in the slums of Nairobi: a retrospective cohort study. *BMC health services research* 2015;15(pp 512) doi: http://dx.doi.org/10.1186/s12913-015-1167-7
15. van de Vijver S, Oti SO, Gomez GB, et al. Impact evaluation of a community-based intervention for prevention of cardiovascular diseases in the slums of Nairobi: the SCALE-UP study. *Glob Health Action* 2016;9(1):30922. doi: 10.3402/gha.v9.30922 [published Online First: 2017/02/06]

For peer review only - http://bmjopen.bmj.com/site/about/guidelines.xhtml

16. Haregu TN, Oti S, Egondi T, et al. Measurement of overweight and obesity an urban slum setting in sub-Saharan Africa: a comparison of four anthropometric indices. *BMC obesity* 2016;3:46. doi: 10.1186/s40608-016-0126-0 [published Online First: 2016/11/12]
17. Ezenwaka CE, Akanji AO, Akanji BO, et al. The prevalence of insulin resistance and other cardiovascular disease risk factors in healthy elderly southwestern Nigerians. *Atherosclerosis* 1997;128(2):201-11. doi: http://dx.doi.org/10.1016/S0021-9150%2896%2905991-6
18. Suriyawongpaisal P, Underwood P. Situation of hypertension in some Bangkok slums. *Journal of the Medical Association of Thailand = Chotmaihet thangphaet* 1993;76(3):123-28.
19. Suriyawongpaisal P, Underwood P, Rouse IL, et al. An investigation of hypertension in a slum of Nakhon Ratchasima. *The Southeast Asian journal of tropical medicine and public health* 1991;22(4):586-94.
20. Sitthi-Amorn C, Chandraprasert S, Bunnag SC, et al. The prevalence and risk factors of hypertension in Klong Toey Slum and Klong Toey government apartment houses. *International Journal of Epidemiology* 1989;18(1):89-94.
21. Bunnag SC, Sitthi-Amorn C, Chandraprasert S. The prevalence of obesity, risk factors and associated diseases in Klong Toey slum and Klong Toey government apartment houses. *Diabetes Res Clin Pract* 1990;10(1)
22. N EST, Jelinek HF, Tarvainen MP, et al. Socioeconomic status, age and heart rate variability in a Bangladeshi community. *Conference proceedings : Annual International Conference of the IEEE Engineering in Medicine and Biology Society IEEE Engineering in Medicine and Biology Society Annual Conference* 2016;01 doi: http://dx.doi.org/10.1109/EMBC.2016.7591919
23. Bhandari S, Sarma PS, Thankappan KR. Adherence to antihypertensive treatment and its determinants among urban slum dwellers in Kolkata, India. *Asia Pacific journal of public health / Asia Pacific Academic Consortium for Public Health* 2015;27(2) doi: http://dx.doi.org/10.1177/1010539511423568
24. Oti SO, van de Vijver S, Kyobutungi C. Trends in non-communicable disease mortality among adult residents in Nairobi's slums, 2003-2011: applying InterVA-4 to verbal autopsy data. *Global health action* 2014;7(pp 25533) doi: http://dx.doi.org/10.3402/gha.v7.25533
25. Hiremath RN, Venkatesh G, Sharvesh, et al. Hypertension status and awareness among geriatric population living in Urban slum. *Nepal Journal of Epidemiology* 2014;Conference:International Conference on Research Methodology and Scientific Writing.
26. Joshi A, Mehta S, Grover A, et al. Knowledge, attitude, and practices of individuals to prevent and manage metabolic syndrome in an Indian setting. *Diabetes Technology and Therapeutics* 2013;15(8):644-53. doi: http://dx.doi.org/10.1089/dia.2012.0309
27. van de Vijver SJ, Oti SO, Agyemang C, et al. Prevalence, awareness, treatment and control of hypertension among slum dwellers in Nairobi, Kenya. *Journal of hypertension* 2013;31(5):1018-24. doi: 10.1097/HJH.0b013e32835e3a56 [published Online First: 2013/02/22]
28. Itrat A, Ahmed B, Khan M, et al. Risk factor profiles of South Asians with cerebrovascular disease. *International Journal of Stroke* 2011;6(4):346-48. doi: http://dx.doi.org/10.1111/j.1747-4949.2011.00622.x
29. Ahmed B, Itrat A, Khan M, et al. Risk factor profiles of south asians with cerebrovascular disease: Findings from a community-based prevalence study in semiurban Pakistan. *Circulation: Cardiovascular Quality and Outcomes* 2011;Conference:Quality of Care

For peer review only - http://bmjopen.bmj.com/site/about/guidelines.xhtml

and Outcomes Research in Cardiovascular Disease and Stroke 2011 Scientific Sessions.

30. Haregu TN, Oti S, Egondi T, et al. Co-occurrence of behavioral risk factors of common non-communicable diseases among urban slum dwellers in Nairobi, Kenya. *Glob Health Action* 2015;8(28697) doi: https://dx.doi.org/10.3402/gha.v8.28697
31. van de Vijver S, Oti S, Moll van Charante E, et al. Cardiovascular prevention model from Kenyan slums to migrants in the Netherlands. *Global health* 2015;11(11):07. doi: https://dx.doi.org/10.1186/s12992-015-0095-y
32. Kohli C, Gupta K. LBOS 03-03 ECONOMIC IMPACT OF HYPERTENSION. *Journal of hypertension* 2016;34 Suppl 1 - ISH 2016 Abstract Book:e551-e52. doi: 10.1097/01.hjh.0000501509.98288.ad [published Online First: 2016/10/19]
33. Mudgapalli V, Sharan S, Amadi C, et al. Perception of receiving SMS based health messages among hypertensive individuals in urban slums. *Technology and Health Care* 2016;24(1):57-65. doi: http://dx.doi.org/10.3233/THC-151097
34. Natarajan S, Mohan S, Satagopan U, et al. Elderly patients with T2DM should be periodically screened for diabetic retinopathy and its complications to reduce visual morbidity - A study from slums of Western India. *Investigative Ophthalmology and Visual Science* 2014;Conference:2014 Annual Meeting of the Association for Research in Vision and Ophthalmology.
35. Kumaramanickavel G, Mohan S, Satagopan U, et al. Diabetic retinopathy in urban slums of Mumbai, India - Social, lifestyle, clinical and genetic risk factors. *Investigative Ophthalmology and Visual Science* 2014;Conference:2014 Annual Meeting of the Association for Research in Vision and Ophthalmology.
36. Kumaramanickavel G, Mohan S, Kumar Singh A, et al. AJDRUMSS-diabetic retinopathy prevalence study in Mumbai slums of India-association of demographic, genetic and medical risk factors. *Investigative Ophthalmology and Visual Science* 2015;Conference:2015 Annual Meeting of the Association for Research in Vision and Ophthalmology.
37. Hulzebosch A, van de Vijver S, Oti SO, et al. Profile of people with hypertension in Nairobi's slums: a descriptive study. *Globalization and health* 2015;11(pp 26) doi: http://dx.doi.org/10.1186/s12992-015-0112-1
38. Madhu B, Srinath KM, Chandresh S, et al. Quality of diabetic care in an urban slum area of Mysore: A community based study. *Diabetes and Metabolic Syndrome: Clinical Research and Reviews* 2016 doi: http://dx.doi.org/10.1016/j.dsx.2016.03.014
39. Mugure G, Karama M, Kyobutungi C, et al. Correlates for cardiovascular diseases among diabetic/hypertensive patients attending outreach clinics in two Nairobi slums, Kenya. *Pan African Medical Journal* 2014;19(no pagination) doi: http://dx.doi.org/10.11604/pamj.2014.19.261.5261
40. Mukhopadhyay A, Sundar U, Adwani S, et al. Prevalence of stroke and post-stroke cognitive impairment in the elderly in Dharavi, Mumbai. *Journal of Association of Physicians of India* 2012;60(10):29-32.
41. Khan RMA, Ahmad M. To assess the public awareness about obesity among adult populace of lahore. *Pakistan Journal of Medical and Health Sciences* 2010;4(4)
42. Etyang A, Harding S, Cruickshank JK. Slum living and hypertension in tropical settings: Neglected issue, statistical artifact or surprisingly slight? Insights amidst adversity. *Journal of Hypertension* 2013;31(5):877-79. doi: http://dx.doi.org/10.1097/HJH.0b013e32836103fb
43. Dhar L. Preventing coronary heart disease risk of slum dwelling residents in India. *Journal of family medicine and primary care* 2014;3(1):58-62. doi: 10.4103/2249-4863.130278 [published Online First: 2014/05/03]

For peer review only - http://bmjopen.bmj.com/site/about/guidelines.xhtml

44. Bhargava SK, Singh KK, Saxena BN. ICMR Task Force National Collaborative Study on Identification of High Risk Families, Mothers and Outcome of their Off-springs with particular reference to the problem of maternal nutrition, low birth weight, perinatal and infant morbidity and mortality in rural and urban slum communities. Summary, conclusions and recommendations. *Indian pediatrics* 1991;28(12):1473-80. [published Online First: 1991/12/01]
45. Kien VD, Van Minh H, Giang KB, et al. Socioeconomic inequalities in self-reported chronic non-communicable diseases in urban Hanoi, Vietnam. *Global Public Health* 2015 doi: http://dx.doi.org/10.1080/17441692.2015.1123282
46. Sur D, Mukhopadhyay SP. A study on smoking habits among slum dwellers and the impact on health and economics. *Journal of the Indian Medical Association* 2007;105(9):492-98.
47. Thakur R, Banerjee A, Nikumb V. Health problems among the elderly: a cross-sectional study. *Annals of medical and health sciences research* 2013;3(1):19-25. doi: 10.4103/2141-9248.109466 [published Online First: 2013/05/02]
48. Ahmedani MY, Fawwad A, Shaheen F, et al. Optimized health care for subjects with type 1 diabetes in a resource constraint society: A three-year follow-up study from Pakistan. *World J Diabetes* 2019;10(3):224-33. doi: 10.4239/wjd.v10.i3.224
49. Ashe S, Routray D. Prevalence, associated risk factors of depression and mental health needs among geriatric population of an urban slum, Cuttack, Odisha. *International Journal of Geriatric Psychiatry* 2019;34(12):1799-807. doi: 10.1002/gps.5195
50. Asiki G, Mohamed SF, Wambui D, et al. Sociodemographic and behavioural factors associated with body mass index among men and women in Nairobi slums: AWI-Gen Project. *Global health action* 2018;11(sup2):1470738-38. doi: 10.1080/16549716.2018.1470738
51. Bagdey PS, Ansari JA, Barnwal RK. Prevalence and epidemiological factors associated with hypertension among post-menopausal women in an urban area of central India. *Clinical Epidemiology and Global Health* 2019;7(1):111-14. doi: 10.1016/j.cegh.2018.02.008
52. Cope AB, Edmonds A, Ludema C, et al. Neighborhood Poverty and Control of HIV, Hypertension, and Diabetes in the Women's Interagency HIV Study. *AIDS Behav* 2020;24(7):2033-44. doi: 10.1007/s10461-019-02757-5
53. De Silva AP, De Silva SHP, Haniffa R, et al. Inequalities in the prevalence of diabetes mellitus and its risk factors in Sri Lanka: a lower middle income country. *Int J Equity Health* 2018;17(1):45-45. doi: 10.1186/s12939-018-0759-3
54. Kapwata T, Manda S. Geographic assessment of access to health care in patients with cardiovascular disease in South Africa. *BMC health services research* 2018;18(1):197-97. doi: 10.1186/s12913-018-3006-0
55. Kawazoe N, Zhang X, Chiang C, et al. Prevalence of hypertension and hypertension control rates among elderly adults during the cold season in rural Northeast China: a cross-sectional study. *J Rural Med* 2018;13(1):64-71. doi: 10.2185/jrm.2959 [published Online First: 2018/05/29]
56. Khanam F, Hossain MB, Mistry SK, et al. Prevalence and Risk Factors of Cardiovascular Diseases among Bangladeshi Adults: Findings from a Cross-sectional Study. *J Epidemiol Glob Health* 2019;9(3):176-84. doi: 10.2991/jegh.k.190531.001
57. Kolak M, Bradley M, Block DR, et al. Urban foodscape trends: Disparities in healthy food access in Chicago, 2007–2014. *Health & Place* 2018;52:231-39. doi: 10.1016/j.healthplace.2018.06.003

For peer review only - http://bmjopen.bmj.com/site/about/guidelines.xhtml

58. Korn A, Bolton SM, Spencer B, et al. Physical and Mental Health Impacts of Household Gardens in an Urban Slum in Lima, Peru. *Int J Environ Res Public Health* 2018;15(8):1751. doi: 10.3390/ijerph15081751
59. Kotian S, Waingankar P, Mahadik V. Assessment of compliance to treatment of hypertension and diabetes among previously diagnosed patients in urban slums of Belapur, Navi Mumbai, India. *Indian Journal of Public Health* 2019;63(4):348. doi: 10.4103/ijph.ijph_422_18
60. Kumar R, Kaur N, Pilania M. Morbidity Pattern of Patients Attending a Primary Healthcare Facility in an Urban Slum of Chandigarh, India. *JOURNAL OF CLINICAL AND DIAGNOSTIC RESEARCH* 2018 doi: 10.7860/jcdr/2018/31331.11297
61. Ma C. The prevalence of depressive symptoms and associated factors in countryside-dwelling older Chinese patients with hypertension. *Journal of Clinical Nursing* 2018;27(15-16):2933-41. doi: 10.1111/jocn.14349
62. Maharana S, Garg S, Dasgupta A, et al. A study on impact of oral health on general health among the elderly residing in a slum of Kolkata: A cross-sectional study. *Indian Journal of Dental Research* 2019;30(2):164. doi: 10.4103/ijdr.ijdr_491_17
63. Nagarkar AM, Kulkarni SS. Obesity and its Effects on Health in Middle-Aged Women from Slums of Pune. *J Midlife Health* 2018;9(2):79-84. doi: 10.4103/jmh.JMH_8_18
64. Narendran M, Rani BBS, Kulkarni P, et al. Interdependence of communicable and Non-Communicable diseases among elderly population in declared slum in Mysuru City, Karnataka. *Indian Journal of Public Health Research & Development* 2018;9(11):62. doi: 10.5958/0976-5506.2018.01426.2
65. Rajapakshe OBW, Sivayogan S, Kulatunga PM. Prevalence and correlates of depression among older urban community-dwelling adults in Sri Lanka. *Psychogeriatrics* 2018;19(3):202-11. doi: 10.1111/psyg.12389
66. Sarkar A, Roy D, Chauhan MM, et al. A lay epidemiological study on coexistent stress in hypertension: Its prevalence, risk factors, and implications in patients' lives. *Journal of family medicine and primary care* 2019;8(3):966-71. doi: 10.4103/jfmpc.jfmpc_60_19
67. Scazufca M, de Paula Couto MCP, Henrique MG, et al. Pilot study of a two-arm non-randomized controlled cluster trial of a psychosocial intervention to improve late life depression in socioeconomically deprived areas of São Paulo, Brazil (PROACTIVE): feasibility study of a psychosocial intervention for late life depression in São Paulo. *BMC public health* 2019;19(1):1152-52. doi: 10.1186/s12889-019-7495-5
68. Wang H, Su M, Fang P-q, et al. Analysis on Medical Expenses of Hypertensive Inpatients in Urban Areas from 2010 to 2013—Evidence from Two Provinces in South of China. *Current Medical Science* 2018;38(4):741-48. doi: 10.1007/s11596-018-1939-5
69. Wekesah FM, Klipstein-Grobusch K, Grobbee DE, et al. Determinants of Mortality from Cardiovascular Disease in the Slums of Nairobi, Kenya. *Glob Heart* 2020;15(1):33-33. doi: 10.5334/gh.787
70. Wilson V, Nittoori S. Risk of type 2 diabetes mellitus among urban slum population using Indian Diabetes Risk Score. *Indian Journal of Medical Research* 2020;152(3):308. doi: 10.4103/ijmr.ijmr_1597_18
71. Yadav S, Saraswat N, Saini AK, et al. A REVIEW ON THE PREVALENCE OF HYPERTENSION IN SIDE-LINED POPULATIONS; SLUM DWELLERS, SHIFT JOB WORKERS AND OCCUPATIONAL NOISE AFFECTED WORKERS: ATTRIBUTABLE TO LIFESTYLE AND ENVIRONMENTAL FACTOR. *Asian Journal of Pharmaceutical and Clinical Research* 2018;11(10):18. doi: 10.22159/ajpcr.2018.v11i10.27007

For peer review only - http://bmjopen.bmj.com/site/about/guidelines.xhtml

72. Zhang X, Chen X, Gong W. Type 2 diabetes mellitus and neighborhood deprivation index: A spatial analysis in Zhejiang, China. *J Diabetes Investig* 2019;10(2):272-82. doi: 10.1111/jdi.12899 [published Online First: 2018/08/28]

For peer review only

For peer review only - http://bmjopen.bmj.com/site/about/guidelines.xhtml

eTable 2: Characteristics of included studies

Study	Country	Slum	Sample size	Age group	% female
Acharyya (2014)	India	North-Parganas	1052	25-64	49.8
Ahmad (2014)	India	Meerut	196	>60	50
Akinwale (2013)	Nigeria	Ijora Oloye, Ajegunle & Makoko	2434		
Anand (2007)	India	Faridabad	2562	15+	50.9
Ayah (2013)	Kenya		2061	18-90	49.1
Banerjee (2016)	India	Kolkata	10167	>20 years	60
Chakerborty (2012)	India	Kolkata	470	18-60	0
Chaturvedi (2007)	India	Delhi	596	>20	
Daniel (2013)	Nigeria	Ajegunle	964	20-81	65.8
Dasappa (2015)	India	Bangalore	2013	35+	50.8
Deepa (2011)	India	Ballabgarh, Delhi, Chennai, Trivandrum , Dibrugarh and Nagpur	15763	15-64	
Edwards (2015)	Kenya	Kibera			
Ezeala-Adikaibe (2016)	Nigeria	Enugu	774	⩾ 20	64.7
Ferreira (2005)	Brazil	Maceio	223	18-65	100
Florencio (2004)	Brazil	Maceio	416	18-60	57
Haregu (2016)	Kenya	Nairobi	5190	18+	46.2
Heitzinger (2014)	Peru	Lima	142	18-81	69.7
Huda (2012)	Bangladesh	Mirpur, Dhaka	1000	15-65	33.4
Jalil (2008)	Pakistan	Lahore	695		43.6
Joshi (2013)	India	Rourkela & Bhubaneswar	100	>18	69
Joshi (2014)	Kenya	Kibera	2045	18-90	49.1
Kar (2008)	India	Chandigarh & Haryana	1010	>30	58.9
Kar (2010)	India	Chandigarh & Haryana	150	>30	62
Khalequzzaman (2017)	Bangladesh	Dhakar	2551	18+	46.7
Kumari (2014)	India	Hyderabad	250		78
Lubree (2002)	India	Pune	150	30-50	100
Marins (2007)	Brazil	Rio-de-Janeiro	3279	>20	56.9
Misra (2001)	India	Gautam-Nagar, Delhi	532		68
Nirmala (2014)	India	Hyderabad, Telangana	700	>20	50.8
Olack (2015)	Kenya	Kibera	1528	35-64	58.1
Oli (2013)	Nepal	Kathmandu	689	15-64	58.9
Ongeti (2013)	Kenya	Kibera	400	14-75	70.3
Oti (2013)	Kenya	Viwandani & Korogocho		18+	46
Patil (2016)	India	Pune, Maharashtra	425	20+	
Rahim (2004)	Bangladesh	Dhakar	1555	20+	52.99
Rawal (2017)	Bangladesh	Dhaka	507		50
Sayeed (2007)	Bangladesh	Dhakar			59.2
Singh (b) (2012)	India	Delhi	474	60+	48
Singh (2012)	India	Patna	3118	>30	56.5
Sinha (2010)	India	Gokulpuri	275	18-40	100
Sithi-Amorn (1989)	Thailand	Klong-Toey	976		54.7

For peer review only - http://bmjopen.bmj.com/site/about/guidelines.xhtml

Snyder (2017)	Brazil		792		64.5
Sowemimo (2015)	Nigeria	Yemetu, Ibadan	806	18-90	
Sunita (2017)	India	Mumbai	6464	>40	
Unger (2015)	Brazil	Salvador	5649	>18	58.3
Uthakalla (2012)	India	Hyderabad		20-60	56
Vigneswari (2014)	India	Chennai	529	18+	77.3
Vigneswari (2015)	India		529	18+	77.3
Vikram (2003)	India	New-Delhi	639		73.4
Wasir (2007)	India	Delhi	278		
Yajnik (2008)	India		142	30-50	0
van de Vijver (2013)	Kenya	Viwandani & Korogocho	5190	>18	46.2
Bawah (2019)	Ghana	Accra	2009		
Chiang (2019)	Bangladesh	Dhaka	423		
Choudhury (2018)	Bangladesh	Dhaka	984	43.4	73
Dwivedi (2018)	India	Bangalore			
Gadallah (2018)	Egypt	West Delhi			
George (2019)	India	Bangalore		57.6	
Gonmei (2018)	India	Delhi			
Jain (2019)	India	Delhi	984	43.4	73
Tymejczyk (2019)	Haiti	Gurugram	420		
Vusirikala (2019)	Kenya	Nairobi		57.6	

For peer review only - http://bmjopen.bmj.com/site/about/guidelines.xhtml

eTable 3: Risk of bias of included studies

Study	Selection of participants	Confounding variables	Measurement of exposure	Blinding of outcome assessments	Incomplete outcome data	Selective outcome reporting
Acharyya (2014)	Low risk	Low risk	Low risk	Low risk	Low risk	Low risk
Ahmad (2014)	Low risk	High risk	Low risk	Low risk	Unclear risk	Low risk
Akinwale (2013)	Low risk	High risk	Low risk	Low risk	Low risk	Low risk
Anand (2007)	Low risk	Low risk	Low risk	Low risk	Low risk	Low risk
Ayah (2013)	Low risk	Low risk	Low risk	Low risk	Low risk	Low risk
Banerjee (2016)	Low risk	Low risk	Low risk	Low risk	Unclear risk	Low risk
Chakerborty (2012)	High risk	High risk	Low risk	Low risk	Low risk	Low risk
Chaturvedi (2007)	Low risk	Low risk	Low risk	Low risk	Low risk	Low risk
Daniel (2013)	Low risk	Low risk	Low risk	Low risk	Low risk	Low risk
Dasappa (2015)	Low risk	High risk	Low risk	Low risk	Low risk	Low risk
Deepa (2011)	Low risk	High risk	Low risk	Low risk	Low risk	Low risk
Edwards (2015)	Low risk	High risk	Low risk	Low risk	Low risk	Low risk
Ezeala-Adikaibe (2016)	High risk	Low risk	Low risk	Low risk	High risk	Low risk
Ferreira (2005)	Low risk	Low risk	Low risk	Low risk	Low risk	Low risk
Florencio (2004)	Low risk	Low risk	Low risk	Low risk	Low risk	Low risk
Haregu (2016)	Unclear risk	Low risk	Low risk	Low risk	Unclear risk	Low risk
Heitzinger (2014)	Low risk	Low risk	Low risk	Low risk	Low risk	Low risk
Huda (2012)	Low risk	High risk	Low risk	Low risk	Low risk	Low risk
Jalil (2008)	Low risk	Low risk	Low risk	Low risk	Low risk	Low risk
Joshi (2013)	High risk	Low risk	Low risk	Low risk	Low risk	Low risk
Joshi (2014)	Low risk	Low risk	Low risk	Low risk	Low risk	Low risk
Kar (2008)	Low risk	Low risk	Low risk	Low risk	Low risk	Low risk
Kar (2010)	Low risk	Low risk	Low risk	Low risk	Low risk	Low risk
Khalequzzaman (2017)	Low risk	Low risk	Low risk	Low risk	Low risk	Low risk
Kumari (2014)	Low risk	High risk	Low risk	Low risk	Low risk	Low risk
Lubree (2002)	Low risk	High risk	Low risk	Low risk	Low risk	Low risk
Marins (2007)	Low risk	High risk	Low risk	Low risk	Low risk	Low risk
Misra (2001)	Low risk	High risk	Low risk	Low risk	Low risk	Low risk
Nirmala (2014)	Low risk	High risk	Low risk	Low risk	Low risk	Low risk
Olack (2015)	Low risk	Low risk	Low risk	Low risk	Low risk	Low risk
Oli (2013)	Low risk	Low risk	Low risk	Low risk	Low risk	Low risk
Ongeti (2013)	Low risk	Low risk	Low risk	Low risk	Low risk	Low risk
Oti (2013)	Low risk	Low risk	Low risk	Low risk	Low risk	Low risk
Patil (2016)	Low risk	High risk	Low risk	Low risk	Low risk	Low risk
Rahim (2004)	Low risk	High risk	Low risk	Low risk	Low risk	Low risk
Rawal (2017)	Low risk	Low risk	Low risk	Low risk	Low risk	Low risk
Sayeed (2007)	Low risk	High risk	Low risk	Low risk	Low risk	Low risk
Singh (b) (2012)	Low risk	Low risk	Low risk	Low risk	Low risk	Low risk
Singh (2012)	Low risk	Low risk	Low risk	Low risk	Low risk	Low risk

For peer review only

Study	Selection of participants	Confounding variables	Measurement of exposure	Blinding of outcome assessments	Incomplete outcome data	Selective outcome reporting
Sinha (2010)	Low risk	Low risk	Low risk	Low risk	Low risk	Low risk
Sithi-Amorn (1989)	Low risk	High risk	Low risk	Low risk	Low risk	Low risk
Snyder (2017)	Low risk	Low risk	Low risk	Low risk	Low risk	Low risk
Sowemimo (2015)	Low risk	Low risk	Low risk	Low risk	Unclear risk	Low risk
Sunita (2017)	Low risk	High risk	Low risk	Low risk	Low risk	Low risk
Unger (2015)	Low risk	Low risk	Low risk	Low risk	Low risk	Low risk
Uthakalla (2012)	Low risk	High risk	Low risk	Low risk	Low risk	Low risk
Vigneswari (2014)	Low risk	High risk	Low risk	Low risk	Low risk	Low risk
Vigneswari (2015)	Low risk	High risk	Low risk	Low risk	Low risk	Low risk
Vikram (2003)	Low risk	Low risk	Low risk	Low risk	Low risk	Low risk
Wasir (2007)	Low risk	High risk	Low risk	Low risk	High risk	Low risk
Yajnik (2008)	Low risk	High risk	Low risk	Low risk	Low risk	Low risk
van de Vijver (2013)	Low risk	Low risk	Low risk	Low risk	Low risk	Low risk
Bawah (2019)	Unclear risk	Low risk	Low risk	Low risk	Unclear risk	Low risk
Chiang (2019)	Low risk	Low risk	Low risk	Low risk	Low risk	Low risk
Choudhury (2018)	Low risk	Low risk	Low risk	Low risk	Low risk	Low risk
Dwivedi (2018)	Low risk	Low risk	Low risk	Low risk	Low risk	Low risk
Gadallah (2018)	Low risk	Low risk	Low risk	Low risk	Low risk	Low risk
George (2019)	Low risk	Low risk	Low risk	Low risk	Low risk	Low risk
Gonmei (2018)	Unclear risk	Unclear risk	Low risk	Low risk	Unclear risk	Low risk
Jain (2019)	Low risk	Low risk	Low risk	Low risk	Low risk	Low risk
Tymejczyk (2019)	Low risk	Low risk	Low risk	Low risk	Low risk	Low risk
Vusirikala (2019)	Low risk	Low risk	Low risk	Low risk	Low risk	Low risk

For peer review only

For peer review only - http://bmjopen.bmj.com/site/about/guidelines.xhtml

Annex 1: MEDLINE Search Strategy

1 exp hypertension/<br>2 hypertens$.mp.<br>3 exp blood pressure/<br>4 (blood pressure or bloodpressure).mp.<br>5 (essential adj3 hypertension).ti,ab.<br>6 (isolat* adj3 hypertension).ti,ab.<br>7 (elevat* adj3 blood adj pressur*).ti,ab.<br>8 (high adj3 blood adj pressur*).ti,ab.<br>9 (increase* adj3 blood pressur*).ti,ab.<br>10 ((systolic or diastolic or arterial) adj3 pressur*).ti,ab.<br>11 essential hypertension.mp.<br>12 isolated hypertension.mp.<br>13 elevated blood pressure.mp.<br>14 high blood pressure.mp.<br>15 increase blood pressure.mp.<br>16 diastolic pressure.mp.<br>17 pre-hypertension.mp.<br>18 pre-hypertensive.mp.<br>19 prehypertension.mp.<br>20 prehypertensive.mp.<br>21 arterial pressure.mp.<br>22 cardiovascular diseases/<br>23 exp coronary disease/<br>24 cardiovascular risk factor$.tw.<br>25 (cardiovascular adj3 disease$).tw.<br>26 (Coronary adj3 disease$).tw.<br>27 heart disease$.tw.<br>28 coronary risk factor$.tw.<br>29 or/1-28
1 exp Diabetes Mellitus, Type 2/<br>2 exp DIABETES MELLITUS/<br>3 T2DM.ti,ab.<br>4 (Type* adj3 ("2" or "II" or two*) adj3 (diabete* or diabetic*)).tw.<br>5 ((Maturit* or adult* or slow*) adj3 onset* adj3 (diabete* or diabetic*)).tw.<br>6 ((Ketosis-resistant* or stable*) adj3 (diabete* or diabetic*)).tw.<br>7 ((Non-insulin* or Non insulin* or Noninsulin*) adj3 depend* adj3 (diabete* or diabetic*)).tw.<br>8 IDDM.ti,ab.<br>9 diabet$.ti.<br>10 PREDIABETIC STATE/<br>11 prediabet$.ti,ab.<br>12 impaired glucose tolerance.ti,ab.<br>13 IGT.ti,ab.<br>14 Impaired fasting glucose.ti,ab.<br>15 IFG.ti,ab.<br>16 Impaired glucose regulation.ti,ab. 1<br>17 IGR.ti,ab.<br>18 GLUCOSE INTOLERANCE/<br>19 (diabet* or glucose or hyperglycaemia or hyperglycaemia or postprandial or post-prandial or insulin or hypoglycemia or hypoglycaemia or IGT or OGTT or CGMS).tw.<br>20 (subclinical diabetes" or "subclinical diabetic" or "sub-clinical diabetes" or "sub-clinical diabetic").tw.<br>21 or/1-20
22 (baladi or bandas de miseria or barraca or barrio marginal or barrio or bidonville or brarek or bustee or chalis or chereka bete or dagatan or estero or favela or galoos or gecekondu or hrushebi).mp.<br>23 (ishash or karyan or katras or looban or loteamento or medina achouaia or morro or mudun safi or musseque or solares or tanake or taudis or township or tugurio or udukku or umjondolo or watta or zopadpattis).mp.<br>24 (slum or slums or ghetto or ghettos or informal settlement$ or shantytown$ or shanty town$).mp.<br>25 slum/<br>26 ghetto/<br>27 or/22-26

For peer review only

Annex 2: PRISMA Checklist

Section/topic	#	Checklist item	Reported on page #
**TITLE**			
Title	1	Identify the report as a systematic review, meta-analysis, or both.	1
**ABSTRACT**			
Structured summary	2	Provide a structured summary including, as applicable: background; objectives; data sources; study eligibility criteria, participants, and interventions; study appraisal and synthesis methods; results; limitations; conclusions and implications of key findings; systematic review registration number.	2 -3
**INTRODUCTION**			
Rationale	3	Describe the rationale for the review in the context of what is already known.	6
Objectives	4	Provide an explicit statement of questions being addressed with reference to participants, interventions, comparisons, outcomes, and study design (PICOS).	6-7
**METHODS**			
Protocol and registration	5	Indicate if a review protocol exists, if and where it can be accessed (e.g., Web address), and, if available, provide registration information including registration number.	8
Eligibility criteria	6	Specify study characteristics (e.g., PICOS, length of follow-up) and report characteristics (e.g., years considered, language, publication status) used as criteria for eligibility, giving rationale.	8-9
Information sources	7	Describe all information sources (e.g., databases with dates of coverage, contact with study authors to identify additional studies) in the search and date last searched.	8
Search	8	Present full electronic search strategy for at least one database, including any limits used, such that it could be repeated.	8
Study selection	9	State the process for selecting studies (i.e., screening, eligibility, included in systematic review, and, if applicable, included in the meta-analysis).	9
Data collection process	10	Describe method of data extraction from reports (e.g., piloted forms, independently, in duplicate) and any processes for obtaining and confirming data from investigators.	9
Data items	11	List and define all variables for which data were sought (e.g., PICOS, funding sources) and any assumptions and simplifications made.	9
Risk of bias in individual studies	12	Describe methods used for assessing risk of bias of individual studies (including specification of whether this was done at the study or outcome level), and how this information is to be used in any data synthesis.	10-11
Summary measures	13	State the principal summary measures (e.g., risk ratio, difference in means).	11-12
Synthesis of results	14	Describe the methods of handling data and combining results of studies, if done, including measures of consistency (e.g., I^2) for each meta-analysis.	11-12

For peer review only - http://bmjopen.bmj.com/site/about/guidelines.xhtml

Section/topic	#	Checklist item	Reported on page #
Risk of bias across studies	15	Specify any assessment of risk of bias that may affect the cumulative evidence (e.g., publication bias, selective reporting within studies).	10-11
Additional analyses	16	Describe methods of additional analyses (e.g., sensitivity or subgroup analyses, meta-regression), if done, indicating which were pre-specified.	12
**RESULTS**			
Study selection	17	Give numbers of studies screened, assessed for eligibility, and included in the review, with reasons for exclusions at each stage, ideally with a flow diagram.	13
Study characteristics	18	For each study, present characteristics for which data were extracted (e.g., study size, PICOS, follow-up period) and provide the citations.	13
Risk of bias within studies	19	Present data on risk of bias of each study and, if available, any outcome level assessment (see item 12).	13-14
Results of individual studies	20	For all outcomes considered (benefits or harms), present, for each study: (a) simple summary data for each intervention group (b) effect estimates and confidence intervals, ideally with a forest plot.	14-18
Synthesis of results	21	Present results of each meta-analysis done, including confidence intervals and measures of consistency.	14-20
Risk of bias across studies	22	Present results of any assessment of risk of bias across studies (see Item 15).	13-14
Additional analysis	23	Give results of additional analyses, if done (e.g., sensitivity or subgroup analyses, meta-regression [see Item 16]).	19-20
**DISCUSSION**			
Summary of evidence	24	Summarize the main findings including the strength of evidence for each main outcome; consider their relevance to key groups (e.g., healthcare providers, users, and policy makers).	21-23
Limitations	25	Discuss limitations at study and outcome level (e.g., risk of bias), and at review-level (e.g., incomplete retrieval of identified research, reporting bias).	23
Conclusions	26	Provide a general interpretation of the results in the context of other evidence, and implications for future research.	23
**FUNDING**			
Funding	27	Describe sources of funding for the systematic review and other support (e.g., supply of data); role of funders for the systematic review.	24

For peer review only
